# Supplementary material for: Dual catalysis for enantioselective convergent synthesis of enantiopure vicinal amino alcohols
Source: Nat Commun. 2018 Jan 29;9:410. doi: 10.1038/s41467-017-02698-4 (PMC5789022; doi:10.1038/s41467-017-02698-4)
Supplement: Supplementary file 1 — Supplementary Information [file 41467_2017_2698_MOESM1_ESM.pdf]

# Supplementary Methods

## General Information:

Melting points (mp) were determined on Buchi M-560 melting point apparatus and were uncorrected. Infrared (IR) spectra were measured with Nicolet FT-380 spectrometer using film KBr pellet techniques. $^{1}H$ and $^{13}C$ NMR spectra were recorded on Bruker Advance III 400 MHz or Bruker Advance III 500 MHz spectrometer. Chemical shifts are expressed in parts per million (δ) referenced to tetramethyl silane (0.0 ppm), chloroform (7.26 ppm or 77.0 ppm), methanol (3.31 ppm or 49.0 ppm) and dimethyl sulfoxide (2.50 ppm or 39.5 ppm), respectively. The NMR data are presented as follows: chemical shift, multiplicity (s = singlet, d = doublet, t = triplet, q = quartet, dd = doublet of doublet, dt = doublet of triplet, dq = doublet of quartet, td = triplet of doublet, qd = quartet of doublet, ddd = doublet of doublet of doublet, m = multiplet, br = broad), coupling constant (Hz) and integration. High resolution mass spectra (HRMS) were recorded on Bruker 7.0 T FT-MS mass spectrometer. Mass spectra (MS) were measured with Bruker amaZon SL mass spectrometer. Optical rotations were measured using Anton Paar MCP-500 polarimeter. Diastereomeric ratios (dr) were determined by $^{1}H$ NMR analysis or HPLC analysis of the crude reaction mixtures. Enantiomeric excesses (ee) were determined by chiral HPLC analysis using Shimadzu LC-10ATVP (or Agilent HPLC 1260) with Lux (5 μm, 250×10 mm) Amylose-1 chiral column and ethanol (or isopropyl alcohol) in hexane.

Chemicals were used as received from commercial suppliers unless otherwise stated. The aromatic aldehydes used in the photo reactions were purified by passing through neutral alumina columns. 1,2-Dichloroethane (DCE), dichloromethane (DCM) and MeCN were both distilled over $CaH_2$ and bubbled with argon for one hour to remove dissolved oxygen. $Ru(bpy)_3(PF_6)_2$ was purchased from Sigma Aldrich. A PHILIPS 65 W (or 45 W) compact fluorescent lamp (CFL) was used as the light source. Flash chromatography was carried out using SiliCycle Silia*Flash* P60 (40−63 μm) silica gel.

Aldonitrones **1a**[1], **1e**[2], **1f**[1], **1g**[1], **1h**[1], **1i**[3], **1j**[3], **1n**[4] and **1o**[1] were prepared by condensation of commercially available *N*-alkyl hydroxylamine hydrochlorides with the corresponding aldehydes according to reported procedure[1]; ketonitrones **1b**[5], **1c**[6] and **1d**[7] were prepared by $Sc(OTf)_3$-promoted condensation of *N*-benzyl hydroxylamine with the corresponding ketones; cyclic nitrone **1k** was purchased from Matrix Scientific, **1l** and **1m** were prepared by $MnO_2$-oxidation of the corresponding cyclic hydroxylamines[8]. The spectroscopic characterization of above-mentioned nitrones matched those reported in the literatures.

NO-Feng-PD*i*PPRa (**L1-a**), NO-Feng-PD*i*PPPy (**L1-c**), NO-Feng-PD*i*PPPi (**L1-d**), NO-Feng-PDMPRa (**L1-e**), (*R*)-*i*Pr-PyBOX (**L2-a**), (*S*)-Ph-PyBOX (**L2-c**), (*S*,*R*)-IN-PyBOX (**L2-e**) and **L3** to **L10** were commercially available chiral ligands. NO-Feng-PT*i*PPRa (**L1-b**) and other Feng's chiral *N*,*N*′-dioxide ligands **L1-f** to **L1-h** were prepared according to the reported procedure[9] (Supplementary Table 1).

***Supplementary Table 1.*** Chiral ligands for optimization

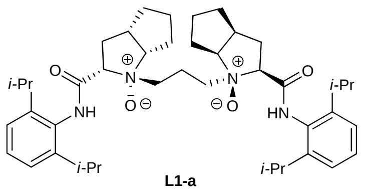
**L1-a**
NO-Feng-PD*i*PPRa

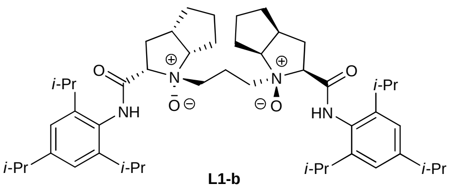
**L1-b**
NO-Feng-PT*i*PPRa

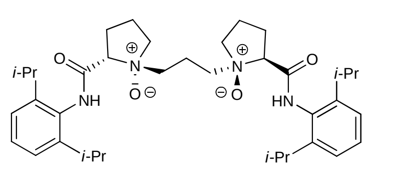
**L1-c**
NO-Feng-PD*i*PPPy

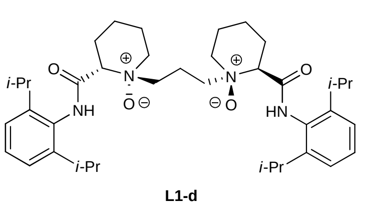
**L1-d**
NO-Feng-PD*i*PPPi

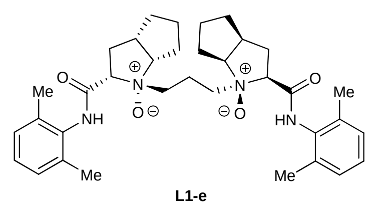
**L1-e**
NO-Feng-PDMPRa

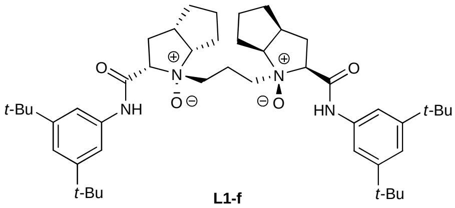
**L1-f**
NO-Feng-PD*t*BPRa

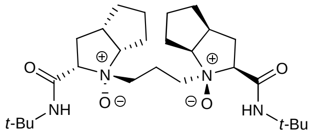
**L1-g**
NO-Feng-P*t*BRa

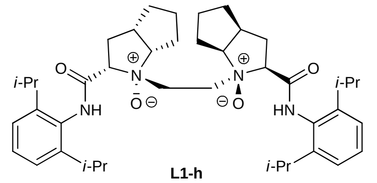
**L1-h**
NO-Feng-ED*i*PPRa

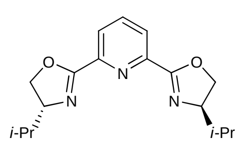
**L2-a**
(*R*)-*i*Pr-PyBOX

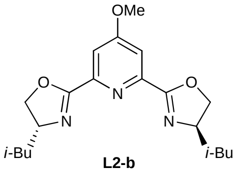
**L2-b**
(*R*)-*i*Bu-4'-OMe-PyBOX

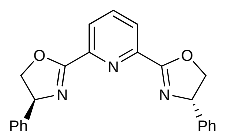
**L2-c**
(*S*)-Ph-PyBOX

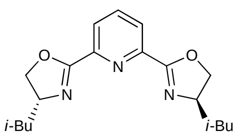
**L2-d**
(*R*)-*i*Bu-PyBOX

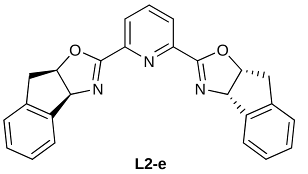
**L2-e**
(*S*,*R*)-IN-PyBOX

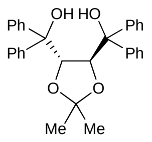
**L3**
(+)-TADDOL

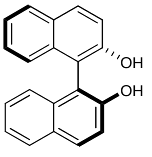
**L4**
(–)-BINOL

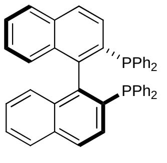
**L5**
(–)-BINAP

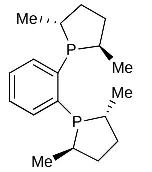
**L6**
(*R*,*R*)-Me-DUPHOS

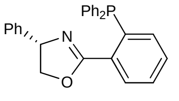
**L7**
(*S*)-Ph-PHOX

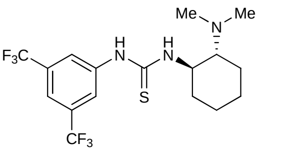
**L8**
(*R*,*R*)-TUC

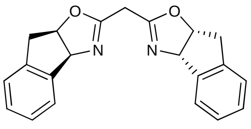
**L9**
(*S*,*R*)-IN-BOX

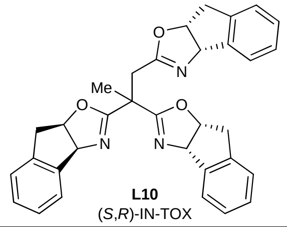
**L10**
(*S*,*R*)-IN-TOX

**L1-b** as a white solid: mp 128.5−129.2 ºC; $[\alpha]_D^{20} = -54.5$ (*c* 1.0, $CHCl_3$); IR (film) $v_{max}$: 3459, 2956, 2876, 1672, 1521 $cm^{-1}$; $^1H$ NMR (400 MHz, $CDCl_3$) δ 1.13−1.31 (m, 36H), 1.56−1.69 (m, 4H), 1.69−1.84 (m, 4H), 1.95−2.10 (m, 2H), 2.32−2.43 (m, 2H), 2.57−2.76 (m, 6H), 2.76−2.93 (m, 4H), 3.02 (heptet, *J* = 6.9 Hz, 4H), 3.28−3.41 (m, 2H), 3.43−3.56 (m, 2H), 3.90 (dd, *J* = 13.0, 5.2 Hz, 2H), 3.97−4.10 (m, 2H), 7.00 (s, 4H), 12.38−12.49 (s, 2H); $^{13}C$ NMR (100 MHz, $CDCl_3$) δ 18.8, 23.2, 23.9, 27.0, 27.6, 28.9, 32.0, 34.1, 34.5, 42.4, 65.8, 80.8, 83.8, 121.1, 128.7, 144.3, 147.7, 166.1; HRMS calcd for $[C_{49}H_{77}N_4O_4]^+$ $(M + H)^+$: 785.5939; found: 785.5947.

(*R*)-*i*Bu-4′-OMe-PyBOX (**L2-b**) was prepared according to following procedures:

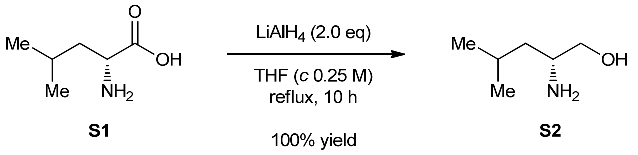


To a stirred 0 ºC suspension of $LiAlH_4$ (5.70 g, 150 mmol, 2.0 equiv.) and THF (300 mL) in a 500 mL two-necked round bottom flask was added **S1** (9.84 g, 75.0 mmol, 1.0 equiv.) portion wise under an argon atmosphere. The mixture was stirred at 0 ºC for 0.5 hour, and then was heated to reflux for 10 hours. To quench the reaction, the mixture was cooled to 0 ºC, slowly added water (16 mL) and stirred for 0.5 hour at 0 ºC. Anhydrous $Na_2SO_4$ was added to absorb the excess water. After filtration, the solvent was evaporated under reduced pressure to afford the desired product **S2** as a colorless oil (8.79 g, 75.0 mmol, quant. yield). The crude product was used for next step without further purification.

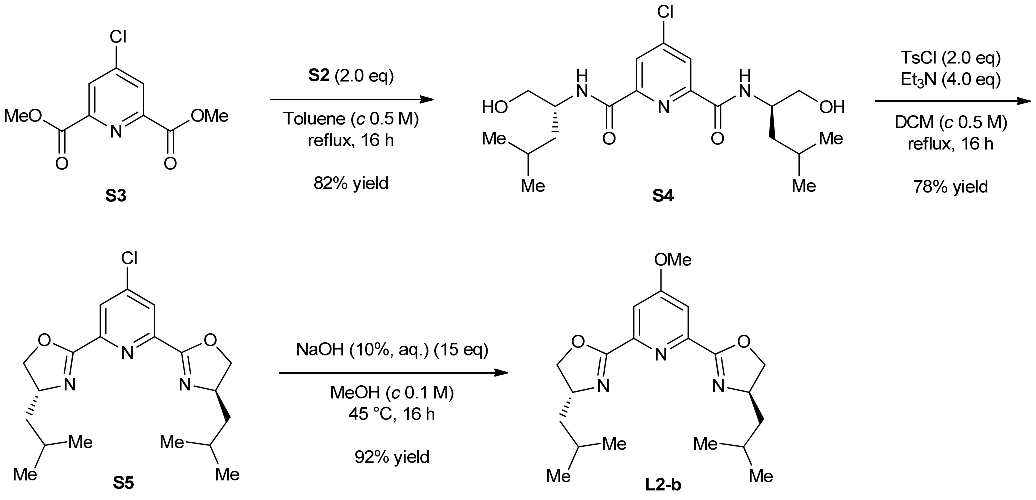


A mixture of **S3** (8.92 g, 38.8 mmol, 1.0 equiv.) and **S2** (9.11 g, 77.7 mmol, 2.0 equiv.) in toluene (78 mL) was heated to reflux for 16 hours. After cooling to room temperature, the solvent was evaporated under reduced pressure, and the residue was purified by flash chromatography on silica gel to afford **S4** as a yellow oil (eluent: EtOAc/PE = 3/1; 12.8 g, 32.0 mmol, 82% yield).

To a stirred solution of **S4** (12.8 g, 32.0 mmol, 1.0 equiv.) in $CH_2Cl_2$ (64 mL) was added $Et_3N$ (17.8 mL, 128 mmol, 4.0 equiv.) and *p*-toluenesulfonyl chloride (12.2 g, 64.0 mmol, 2.0 equiv.). The mixture was heated to reflux for 16 hours. After cooling to room temperature, the mixture was washed with water (50 mL). The organic layer was dried over anhydrous $MgSO_4$, filtered and concentrated under reduced pressure. The residue was purified by flash chromatography on silica gel to afford **S5** as a yellow oil (eluent: EtOAc/PE = 1/3 with 1% $Et_3N$; 9.03 g, 24.8 mmol, 78% yield).

To a stirred solution of **S5** (9.03 g, 24.8 mmol, 1.0 equiv.) in methanol (250 mL) was added aqueous NaOH solution (10% wt, 14.9 g NaOH in 134 mL water; 372 mmol, 15 equiv.). After being stirred at 45 ºC for 16 hours, the mixture was cooled and extracted with $CH_2Cl_2$ (3×150 mL). The combined organic layers were washed with brine, dried over anhydrous $MgSO_4$, filtered and concentrated under reduced pressure. The residue was purified by flash chromatography on silica gel to afford **L2-b** as a white solid (eluent: EtOAc/PE = 1/1 with 1% $Et_3N$; 8.23 g, 22.9 mmol, 92% yield). After being recrystallized in EtOAc, the ligand was stored at 4 ºC under an argon atmosphere: mp 108.6−109.2 ºC; $[\alpha]_D^{20}$ = +102.0 (*c* 1.0, $CHCl_3$); IR (film) $\nu_{max}$: 3446, 2959, 2927, 2869, 1643, 1588, 1467, 1399, 1217, 1085, 1047 $cm^{-1}$; $^1H$ NMR (400 MHz, $CDCl_3$) δ 0.92−1.03 (m, 12H), 1.33−1.44 (m, 2H),

1.67−1.77 (m, 2H), 1.77−1.91 (m, 2H), 3.94 (s, 3H), 4.08 (dd, $J$ = 8.5, 8.2 Hz, 2H), 4.31−4.43 (m, 2H), 4.59 (dd, $J$ = 9.2, 8.5 Hz, 2H), 7.68 (s, 2H); $^{13}$C NMR (100 MHz, $CDCl_3$) δ 22.6, 22.8, 25.4, 45.4, 55.8, 65.3, 73.8, 111.6, 148.4, 162.2, 166.6; HRMS calcd for $[C_{20}H_{30}N_3O_3]^+$ $(M + H)^+$: 360.2282; found: 360.2280.

## Optimization of the Reaction Conditions:

The optimization of the reaction conditions was conducted with the following procedure using nitrone **1a** and 4-fluorobenzaldehyde as the model reaction: an oven-dried 25 mL Schlenk tube equipped with a magnetic stir bar was added nitrone **1a** (53.2 mg, 0.30 mmol, 1.0 equiv.), chiral ligand (18 mol% for *N*,*N'*-dioxides, or 30 mol% for other ligands), $Ru(bpy)_3(PF_6)_2$ (5.2 mg, 0.006 mmol, 2.0 mol%) and Lewis acid (0.045 mmol, 15 mol%) in the glove box. When the tube was sealed and removed from the glove box, solvent (6.0 mL) was added, followed by 4-fluorobenzaldehyde (48 μL, 0.45 mmol, 1.5 equiv.) and the co-reductant (1.2 mmol, 4.0 equiv.). The tube was placed approximately 10 cm away from a 65 W CFL. After being stirred at 0 ºC under an argon atmosphere for 48 to 72 hours, the reaction mixture was filtered through a thin pad of silica gel (100 to 200 mesh), washed with EtOAc and concentrated under reduced pressure. The residue was purified by flash chromatography on silica gel to afford **2a-1**. The yields were the combined yields for two diastereo isomers. The dr values were determined by $^1$H NMR integral area of the crude products [$\delta_H$ 5.53 (*anti*), 4.87 (*syn*) in $CDCl_3$], and the ee values were determined by chiral HPLC analysis of the major diastereo isomers [Lux® Amylose-1 column, Shimadzu HPLC, 30 ºC, hexane/EtOH = 85/15 (v/v), 1.0 mL/min, 220 nm; $t_1$ = 7.9 min, $t_2$ = 16.7 min].

Our investigation commenced with the general photocatalytic reductive condition (Supplementary Table 2 and Supplementary Table 3), and the desired diastereomeric mixture of vicinal hydroxyamino alcohols was obtained while a catalytic amount of Lewis acid was added (Supplementary Table 2, entries 1 and 2). By using a 45 W CFL in the presence of $La(OTf)_3$ (15 mol%) and *N*,*N*-diisopropylethylamine (DIPEA) (4.0 equiv.), the coupling of nitrone **1a** with 4-fluorobenzaldehyde in $CH_3CN$ at 30 ºC for 48 hours offered (±)-**2a-1** in 92% combined yield (Supplementary Table 2, entry 1). Other photocatalysts, such as $Ir(ppy)_3$ and $Ir(ppy)_2(dtbbpy)(PF_6)$ also were used under this condition, but just the latter can give desired product in moderate yield (56%) (Supplementary Table 2, entries 3 and 4). To get enantioselective results, a series of factors such as chiral ligands, Lewis acids, co-reductants, temperature and solvents were studied. Fortunately, by using a 65 W CFL at 0 ºC with Feng's chiral *N*,*N'*-dioxides (**L1-a** to **L1-h**) and PyBOX ligands (**L2-a** and **L2-b**), mild to good ee were obtained (Supplementary Table 2, entries 5 to 8, and Supplementary Table 3). Noteworthily, complexes of $Sc(OTf)_3$ with Feng's chiral *N*,*N'*-dioxides should be used in $CH_2Cl_2$ (Supplementary Table 2, entries 5 and 6, and Supplementary Table 3), while complexes of $La(OTf)_3$ with PyBOX ligands should be used in $CH_3CN$ (Supplementary Table 2, entries 7 and 8). When *N*,*N*,*N'*,*N'*-tetraethylethylenediamine (TEEDA) was used as co-reductant instead of DIPEA, a higher stereoselectivity was obtained in a slightly lower yield (Supplementary Table 2, entry 9 *vs.* entry 5, and entry 10 *vs.* entry 6). The complex of $Sc(OTf)_3$ with Feng's chiral *N*,*N'*-dioxide **L1-b** was the best chiral catalyst (Supplementary Table 2, entries 10 to 13). The best enantioselectivity was obtained at 0 ºC (Supplementary Table 2, entries 10, and 14 to 16). Finally, when DCE was used as solvent, the yield of desired product **2a-1** was increased to 93% without loss of ee (Supplementary Table 2, entries 17 and 18).

***Supplementary Table 2.*** Optimization of Lewis acids, ligands, co-reductants, temperature and solvents

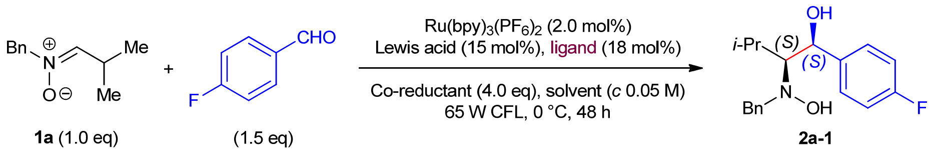


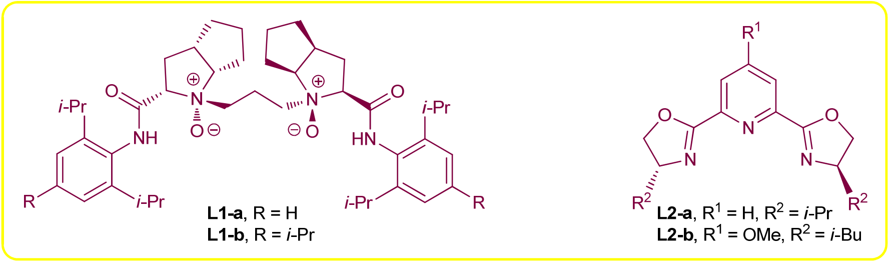


| Entry | Lewis acid | Ligand | Co-reductant | Solvent | Yield (%)[h] | dr | ee (%) |
|---|---|---|---|---|---|---|---|
| 1[a] | $La(OTf)_3$ | _ | $i$Pr$_2$NEt | $CH_3CN$ | 92 | 1/3.4 | _ |
| 2[a] | $Sc(OTf)_3$ | _ | $i$Pr$_2$NEt | $CH_3CN$ | 39 | 1/2.0 | _ |
| 3[b] | $La(OTf)_3$ | _ | $i$Pr$_2$NEt | $CH_3CN$ | trace (<5%) | _ | _ |
| 4[c] | $La(OTf)_3$ | _ | $i$Pr$_2$NEt | $CH_3CN$ | 56 | 1/3.4 | _ |
| 5 | $Sc(OTf)_3$ | **L1-a** | $i$Pr$_2$NEt | DCM | 99 | 1/6.8 | 84 |
| 6 | $Sc(OTf)_3$ | **L1-b** | $i$Pr$_2$NEt | DCM | 99 | 1/6.6 | 82 |
| 7 | $La(OTf)_3$ | **L2-a**[g] | $i$Pr$_2$NEt | $CH_3CN$ | 73 | 1/5.7 | –16 |
| 8 | $La(OTf)_3$ | **L2-b**[g] | $i$Pr$_2$NEt | $CH_3CN$ | 83 | 1/6.9 | –24 |
| 9 | $Sc(OTf)_3$ | **L1-a** | TEEDA | DCM | 81 | 1/10 | 89 |
| 10 | $Sc(OTf)_3$ | **L1-b** | TEEDA | DCM | 73 | 1/11 | 92 |
| 11 | $La(OTf)_3$ | **L1-b** | TEEDA | DCM | 57 | 1/20 | 12 |
| 12 | $Eu(OTf)_3$ | **L1-b** | TEEDA | DCM | 30 | 1/10 | 11 |
| 13 | $Y(OTf)_3$ | **L1-b** | TEEDA | DCM | 31 | 1/12 | –0.6 |
| 14[d] | $Sc(OTf)_3$ | **L1-b** | TEEDA | DCM | 73 | 1/11 | 91 |
| 15[e] | $Sc(OTf)_3$ | **L1-b** | TEEDA | DCM | 71 | 1/11 | 91 |
| 16[f] | $Sc(OTf)_3$ | **L1-b** | TEEDA | DCM | 68 | 1/9.5 | 88 |
| 17 | $Sc(OTf)_3$ | **L1-b** | TEEDA | $CH_3CN$ | 46 | 1/3.7 | 92 |
| 18 | $Sc(OTf)_3$ | **L1-b** | TEEDA | DCE | 93 | 1/12 | 92 |

[a] $T$ = 30 °C, $c$ = 0.1 M, 45 W CFL. [b] $Ir(ppy)_3$ instead of $Ru(bpy)_3(PF_6)_2$. [c] $Ir(ppy)_2(dtbbpy)(PF_6)$ instead of $Ru(bpy)_3(PF_6)_2$. [d] $T$ = 20 °C. [e] $T$ = –10 °C. [f] $T$ = –20 °C. [g] **L2** (30 mol%). [h] Yields were determined by $^1$H NMR analysis using 1,3,5-trimethoxybenzene as an internal standard.

***Supplementary Table 3.*** Optimization of Feng's chiral *N*,*N'*-dioxide ligands

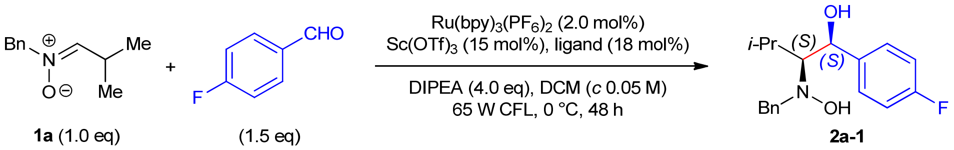


| | | |
|---|---|---|
| 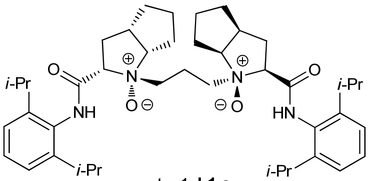 entry 1: **L1-a**<br>99% yield<br>1/6.8 dr<br>84% ee | 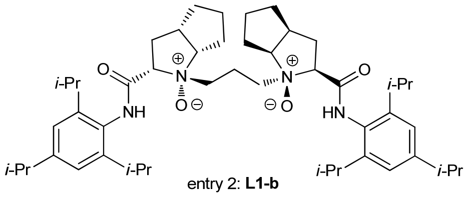 entry 2: **L1-b**<br>99% yield<br>1/6.6 dr<br>82% ee | 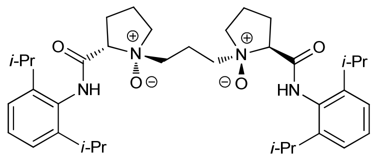 entry 3: **L1-c**<br>74% yield<br>1/11 dr<br>71% ee |
| 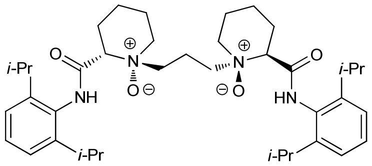 entry 4: **L1-d**<br>79% yield<br>1/6.2 dr<br>47% ee | 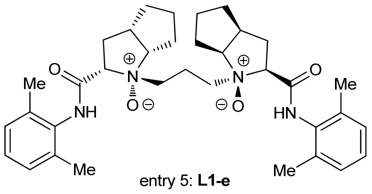 entry 5: **L1-e**<br>37% yield<br>1/20 dr<br>–27% ee | 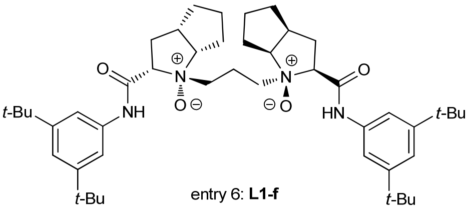 entry 6: **L1-f**<br>15% yield<br>1/10 dr<br>–44% ee |
| 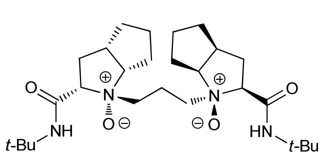 entry 7: **L1-g**<br>8% yield<br>1/3.6 dr<br>–45% ee | 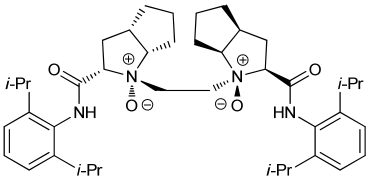 entry 8: **L1-h**<br>57% yield<br>1/12 dr<br>60% ee | 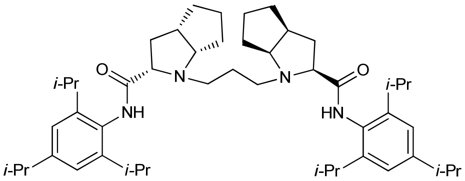 entry 9: **pre-L1-b**<br>29% yield<br>1/1.8 dr<br>0.8% ee |

***Supplementary Note 1.*** Photocatalytic reduction of Feng's ligands promoted by Lewis acid

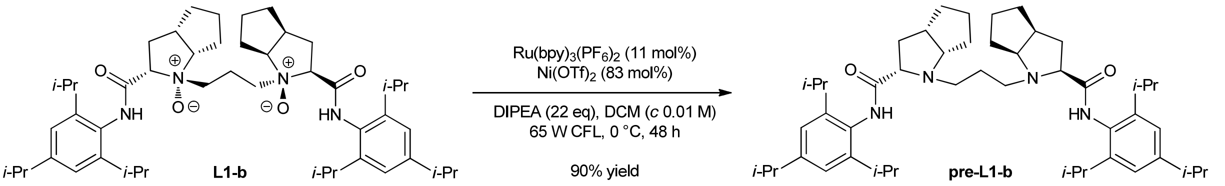


Some Lewis acids can promote the reduction of Feng's ligands under reductive radical conditions. For example, **L1-b** can be reduced to its tertiary diamine precursor under a general photocatalytic reductive condition using $Ni(OTf)_2$ as the Lewis acid. Note that tertiary diamine precursors of Feng's ligands (such as **pre-L1-b**) showed poor catalytic activity in reductive coupling reaction of nitrones with aldehydes (see Supplementary Table 3, entry 9).

## Photocatalytic Enantioselective Reductive Cross-Coupling of Nitrones with Aldehydes:

**General procedure**: An oven-dried 25 mL Schlenk tube equipped with a magnetic stir bar was added nitrone (0.30 mmol, 1.0 equiv.), **L1-b** (42.4 mg, 0.054 mmol, 18 mol%), $Ru(bpy)_3(PF_6)_2$ (5.2 mg, 0.006 mmol, 2.0 mol%) and $Sc(OTf)_3$ (22.1 mg, 0.045 mmol, 15 mol%) in the glove box. When the tube was sealed and removed from the glove box, DCE (or DCM) (6.0 mL) was added, followed by the aldehyde (0.45 mmol, 1.5 equiv.) and TEEDA (0.26 mL, 1.2 mmol, 4.0 equiv.). The tube was placed approximately 10 cm away from a 65 W CFL. After being stirred at 0 ºC under an argon atmosphere for 48 hours, the reaction mixture was filtered through a thin pad of silica gel (100 to 200 mesh), washed with EtOAc and concentrated under reduced pressure. The residue was purified by flash chromatography to afford desired vicinal hydroxyamino alcohols **2a** to **2j**. The yields were the combined yields of two diastereo isomers. The dr values were determined by $^1H$ NMR integral area or chiral HPLC analysis of the crude products, and the ee values were determined by chiral HPLC analysis. Unless otherwise noted, the characterization data and ee values are reported for the major diastereomer.

**Modified method 1:** An oven-dried 25 mL Schlenk tube equipped with a magnetic stir bar was added nitrone (0.30 mmol, 1.0 equiv.), **L1-a** (37.9 mg, 0.054 mmol, 18 mol%), $Ru(bpy)_3(PF_6)_2$ (5.2 mg, 0.006 mmol, 2.0 mol%) and $Sc(OTf)_3$ (22.1 mg, 0.045 mmol, 15 mol%) in the glove box. When the tube was sealed and removed from the glove box, DCM (6.0 mL) was added, followed by the aldehyde (0.45 mmol, 1.5 equiv.) and DIPEA (0.21 mL, 1.2 mmol, 4.0 equiv.). The tube was placed approximately 10 cm away from a 65 W CFL, and the reaction mixture was stirred at −5 ºC under an argon atmosphere for 48 hours to afford desired products **2k** to **2m**.

**Modified method 2:** An oven-dried 25 mL Schlenk tube equipped with a magnetic stir bar was added nitrone (0.30 mmol, 1.0 equiv.), **L2-b** (32.4 mg, 0.09 mmol, 30 mol%), $Ru(bpy)_3(PF_6)_2$ (5.2 mg, 0.006 mmol, 2.0 mol%) and $La(OTf)_3$ (26.4 mg, 0.045 mmol, 15 mol%) in the glove box. When the tube was sealed and removed from the glove box, $CH_3CN$ (6.0 mL) was added, followed by the aldehyde (0.45 mmol, 1.5 equiv.) and TEEDA (0.26 mL, 1.2 mmol, 4.0 equiv.). The tube was placed approximately 10 cm away from a 65 W CFL, and the reaction mixture was stirred at −10 ºC under an argon atmosphere for 72 hours to afford desired products **2k** to **2m**.

### (1*S*,2*S*)-2-(Benzyl(hydroxy)amino)-1-(4-fluorophenyl)-3-methylbutan-1-ol (2a-1)

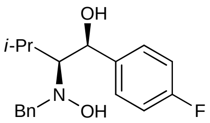


Following the **general procedure**, the asymmetric reductive coupling of nitrone **1a** (53.2 mg, 0.30 mmol, 1.0 equiv.) with 4-fluorobenzaldehyde (48 μL, 0.45 mmol, 1.5 equiv.) gave the desired product **2a-1** as the major diastereomer [dr = 1/12 (*anti*/*syn*), determined by $^1H$ NMR integral area of the crude product, $\delta_H$ 5.53 (*anti*), 4.87 (*syn*)] with 92% ee [Lux® Amylose-1 column, Shimadzu HPLC, 30 ºC, hexane/EtOH = 85/15 (v/v), 1.0 mL/min, 220 nm; $t_1$ = 7.9 min, $t_2$ = 16.7 min]. Characterization data was obtained by chromatography on silica gel (eluent: EtOAc/PE = 1/5; 84.8 mg, 93% combined yield).

Major diastereomer (*syn*, more polar) as a white solid: mp 113.5−114.1 ºC; $[\alpha]_D^{20}$ = +64.5 (*c* 1.0, $CHCl_3$, 92% ee); IR (film) $\nu_{max}$: 3366, 2949, 1505, 1217 $cm^{-1}$; $^1H$ NMR (400 MHz, $CDCl_3$) δ 0.79 (d, *J* = 7.1 Hz, 3H), 0.99 (d, *J* = 7.0 Hz, 3H), 1.98−2.08 (m, 1H), 2.75 (dd, *J* = 8.2, 2.9 Hz, 1H), 3.98 (d, *J* = 13.3 Hz, 1H), 4.03−4.48 (br, 1H), 4.07 (d, *J* = 13.3 Hz, 1H), 4.87 (d, *J* = 8.2 Hz, 1H), 5.01−5.35 (br, 1H), 6.97−7.07 (m, 2H), 7.22−7.39 (m, 7H); $^{13}C$ NMR (100 MHz, $CDCl_3$) δ 20.6, 20.8, 27.0, 63.2, 72.3, 74.9, 115.1 (d, *J* = 21.3 Hz), 127.4, 128.4, 129.1 (d, *J* = 8.1 Hz), 129.2, 138.1, 138.3 (d, *J* = 3.0 Hz), 162.3 (d, *J* = 245.8 Hz); HRMS calcd for $[C_{18}H_{22}FNNaO_2]^+$ $(M + Na)^+$: 326.1527; found: 326.1528.

**(*S*)-2-(Benzyl(hydroxy)amino)-1-(4-fluorophenyl)-2-methylpropan-1-ol (2b-1)**

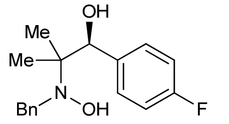


Following the **general procedure**, the asymmetric reductive coupling of nitrone **1b** (49.0 mg, 0.30 mmol, 1.0 equiv.) with 4-fluorobenzaldehyde (48 μL, 0.45 mmol, 1.5 equiv.) gave the desired product **2b-1** as a white solid (chromatography on silica gel, eluent: EtOAc/PE = 1/6; 62.4 mg, 72% yield) with 97% ee [Lux® Amylose-1 column, Shimadzu HPLC, 30 ºC, hexane/EtOH = 60/40 (v/v), 1.0 mL/min, 220 nm; $t_1$ = 17.4 min, $t_2$ = 26.9 min]: mp 148.5−149.2 ºC; $[\alpha]_D^{20}$ = −22.3 (*c* 1.0, $CHCl_3$, 97% ee); IR (film) $\nu_{max}$: 3356, 1505, 1361, 1223, 1041 $cm^{-1}$; $^1H$ NMR (500 MHz, MeOD) δ 1.01 (s, 3H), 1.08 (s, 3H), 3.85 (d, *J* = 13.5 Hz, 1H), 3.99 (d, *J* = 13.5 Hz, 1H), 4.99 (s, 1H), 7.01−7.08 (m, 2H), 7.20−7.25 (m, 1H), 7.27−7.33 (m, 2H), 7.34−7.40 (m, 2H), 7.40−7.47 (m, 2H); $^{13}C$ NMR (125 MHz, MeOD) δ 18.4, 19.0, 57.4, 65.8, 77.8, 115.1 (d, *J* = 21.2 Hz), 127.8, 129.1, 130.3, 131.0 (d, *J* = 8.0 Hz), 138.3 (d, *J* = 2.6 Hz), 140.9, 163.6 (d, *J* = 243.7 Hz); HRMS calcd for $[C_{17}H_{20}FNNaO_2]^+$ $(M + Na)^+$: 312.1370; found: 312.1370.

**(*S*)-(1-(Benzyl(hydroxy)amino)cyclopentyl)(4-fluorophenyl)methanol (2c)**

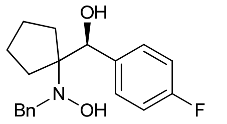


Following the **general procedure**, the asymmetric reductive coupling of nitrone **1c** (56.8 mg, 0.30 mmol, 1.0 equiv.) with 4-fluorobenzaldehyde (48 μL, 0.45 mmol, 1.5 equiv.) gave the desired product **2c** as a white solid (chromatography on silica gel, eluent: EtOAc/PE = 1/6; 47.4 mg, 50% yield) with 94% ee [Lux® Amylose-1 column, Shimadzu HPLC, 30 ºC, hexane/EtOH = 60/40 (v/v), 1.0 mL/min, 220 nm; $t_1$ = 14.8 min, $t_2$ = 18.4 min]: mp 136.9−137.4 ºC; $[\alpha]_D^{20}$ = −21.5 (*c* 1.0, $CHCl_3$, 94% ee); IR (film) $\nu_{max}$: 3388, 2943, 1601, 1511, 1226 $cm^{-1}$; $^1H$ NMR (400 MHz, $CDCl_3$) δ 0.61−0.79 (m, 1H), 1.16−1.27 (m, 1H), 1.37−1.56 (m, 2H), 1.56−1.76 (m, 2H), 1.76−1.98 (m, 2H), 3.85 (d, *J* = 13.4 Hz, 1H), 3.95 (d, *J* = 13.4 Hz, 1H), 4.10−4.66 (br, 1H), 5.10 (s, 1H), 5.19−5.66 (br, 1H), 6.94−7.05 (m, 2H), 7.21−7.43 (m, 7H); $^{13}C$ NMR (100 MHz, $CDCl_3$) δ 25.2, 25.6, 29.4, 30.0, 57.0, 75.8, 77.1, 114.5 (d, *J* = 21.2 Hz), 127.2, 128.3, 129.2, 129.6 (d, *J* = 7.9 Hz), 136.1 (d, *J* = 2.8 Hz), 138.6, 162.1 (d, *J* = 245.5 Hz); HRMS calcd for $[C_{19}H_{22}FNNaO_2]^+$ $(M + Na)^+$: 338.1527; found: 338.1529.

**(*S*)-(1-(Benzyl(hydroxy)amino)cyclohexyl)(4-fluorophenyl)methanol (2d)**

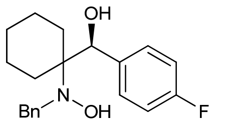


Following the **general procedure**, the asymmetric reductive coupling of nitrone **1d** (61.0 mg, 0.30 mmol, 1.0 equiv.) with 4-fluorobenzaldehyde (48 μL, 0.45 mmol, 1.5 equiv.) gave the desired product **2d** as a white solid (chromatography on silica gel, eluent: EtOAc/PE = 1/6; 64.0 mg, 65% yield) with 92% ee [Lux® Amylose-1 column, Shimadzu HPLC, 30 ºC, hexane/EtOH = 60/40 (v/v), 1.0 mL/min, 225 nm; $t_1$ = 4.4 min, $t_2$ = 5.5 min]: mp 147.4−148.2 ºC; $[\alpha]_D^{20}$ = −37.9 (*c* 1.0, $CHCl_3$, 92% ee); IR (film) $\nu_{max}$: 3356, 2844, 1604, 1450, 1223, 1037 $cm^{-1}$; $^1H$ NMR (400 MHz, $CDCl_3$) δ 0.83−1.08 (m, 2H), 1.08−1.21 (m, 1H), 1.29−1.39 (m, 1H), 1.42−1.51 (m, 1H), 1.56−1.78 (m, 4H), 2.27−2.37 (m, 1H), 4.05 (d, *J* = 13.7 Hz, 1H), 4.20 (d, *J* = 13.7 Hz, 1H), 4.36−4.58 (br, 1H), 5.13 (s, 1H), 5.18−5.28 (br, 1H), 6.95−7.05 (m, 2H), 7.25−7.39 (m, 5H), 7.39−7.46 (m, 2H); $^{13}C$ NMR (100 MHz, $CDCl_3$) δ 20.8 (2C), 26.1, 26.3, 32.6, 54.9, 64.6, 78.2, 114.4 (d, *J* = 21.2 Hz), 127.0, 128.4, 128.8, 129.6 (d, *J* = 8.0 Hz), 137.0 (d, *J* = 2.9 Hz), 139.3, 162.1 (d, *J* = 245.4 Hz); HRMS calcd for $[C_{20}H_{24}FNNaO_2]^+$ $(M + Na)^+$: 352.1683; found: 352.1684.

**(*S*)-2-(Benzyl(hydroxy)amino)-2-methyl-1-phenylpropan-1-ol (2b-2)**

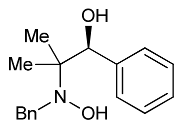

Following the **general procedure**, the asymmetric reductive coupling of nitrone **1b** (49.0 mg, 0.30 mmol, 1.0 equiv.) with benzaldehyde (46 μL, 0.45 mmol, 1.5 equiv.) gave the desired product **2b-2** as a white solid (chromatography on silica gel, eluent: EtOAc/PE = 1/6; 66.7 mg, 82% yield) with 99% ee [Lux® Amylose-1 column, Shimadzu HPLC, 30 ºC, hexane/EtOH = 60/40 (v/v), 1.0 mL/min, 220 nm; $t_1$ = 11.6 min, $t_2$ = 15.3 min]: mp 147.4−148.0 ºC; $[\alpha]_D^{20}$ = –24.1 (*c* 1.0, $CHCl_3$, 99% ee); IR (film) $\nu_{max}$: 3359, 1457, 1358, 1037 $cm^{-1}$; $^1H$ NMR (400 MHz, $CDCl_3$) δ 0.96 (s, 3H), 1.01 (s, 3H), 3.76 (d, *J* = 13.1 Hz, 1H), 3.93 (d, *J* = 13.1 Hz, 1H), 4.03–4.42 (br, 1H), 4.83 (s, 1H), 5.30–5.49 (br, 1H), 7.20–7.38 (m, 10H); $^{13}C$ NMR (100 MHz, $CDCl_3$) δ 16.3, 18.5, 56.1, 65.2, 76.8, 127.2, 127.3, 127.5, 128.1, 128.3, 129.4, 138.5, 140.0; HRMS calcd for $[C_{17}H_{21}NNaO_2]^+$ $(M + Na)^+$: 294.1465; found: 294.1466.

**(*S*)-2-(Benzyl(hydroxy)amino)-2-methyl-1-(4-(methylthio)phenyl)propan-1-ol (2b-3)**

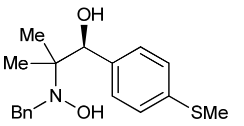

Following the **general procedure**, the asymmetric reductive coupling of nitrone **1b** (49.0 mg, 0.30 mmol, 1.0 equiv.) with 4-(methylthio)benzaldehyde (60 μL, 0.45 mmol, 1.5 equiv.) gave the desired product **2b-3** as a white solid (chromatography on silica gel, eluent: EtOAc/PE = 1/5; 65.0 mg, 68% yield) with 94% ee [Lux® Amylose-1 column, Shimadzu HPLC, 30 ºC, hexane/*i*-PrOH = 85/15 (v/v), 1.0 mL/min, 259 nm; $t_1$ = 9.9 min, $t_2$ = 13.0 min]: mp 160.2−160.8 ºC; $[\alpha]_D^{20}$ = –25.4 (*c* 0.5, $CHCl_3$, 94% ee); IR (film) $\nu_{max}$: 3347, 2917, 1434, 1370, 1041 $cm^{-1}$; $^1H$ NMR (400 MHz, $CDCl_3$) δ 1.00 (s, 3H), 1.05 (s, 3H), 2.49 (s, 3H), 3.80 (d, *J* = 13.1 Hz, 1H), 3.97 (d, *J* = 13.1 Hz, 1H), 4.06–4.21 (br, 1H), 4.78–4.86 (br, 1H), 4.87 (s, 1H), 7.19–7.25 (m, 2H), 7.26–7.41 (m, 7H); $^{13}C$ NMR (100 MHz, $CDCl_3$) δ 15.9, 16.3, 18.6, 56.2, 65.3, 76.6, 125.9, 127.4, 128.4, 128.6, 129.4, 137.1, 137.3, 138.4; HRMS calcd for $[C_{18}H_{23}NNaO_2S]^+$ $(M + Na)^+$: 340.1342; found: 340.1343.

**(1*S*,2*S*)-2-(Benzyl(hydroxy)amino)-1-(4-fluorophenyl)pentan-1-ol (2e)**

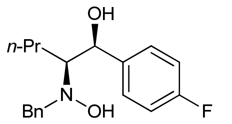

Following the **general procedure**, the asymmetric reductive coupling of nitrone **1e** (53.2 mg, 0.30 mmol, 1.0 equiv.) with 4-fluorobenzaldehyde (48 μL, 0.45 mmol, 1.5 equiv.) gave the desired product **2e** as the minor diastereomer [dr = 1.1/1 (*anti*/*syn*), determined by $^1H$ NMR integral area of crude product, $\delta_H$ 5.44 (*anti*), 4.43 (*syn*)]; major diastereomer (*anti*): 82% ee [Lux® Amylose-1 column, Shimadzu HPLC, 30 ºC, hexane/EtOH = 80/20 (v/v), 1.0 mL/min, 265 nm; $t_1$ = 5.7 min, $t_2$ = 6.0 min]; minor diastereomer (*syn*): 83% ee [Lux® Amylose-1 column, Shimadzu HPLC, 30 ºC, hexane/EtOH = 60/40 (v/v), 1.0 mL/min, 265 nm; $t_1$ = 5.1 min, $t_2$ = 12.7 min]. Characterization data was obtained by chromatography on silica gel (eluent: EtOAc/PE = 1/5; 62.8 mg, 69% combined yield).

Major diastereomer (*anti*, less polar) as a white solid: mp 117.4−118.2 ºC; $[\alpha]_D^{20}$ = +3.5 (*c* 1.0, $CHCl_3$, 82% ee); IR (film) $\nu_{max}$: 3366, 2956, 1508, 1220 $cm^{-1}$; $^1H$ NMR (400 MHz, $CDCl_3$) δ 0.69 (t, *J* = 7.0 Hz, 3H), 0.95−1.08 (m, 1H), 1.28−1.45 (m, 2H), 1.57−1.70 (m, 1H), 2.65−2.76 (m, 1H), 3.54−3.95 (br, 1H), 4.00 (d, *J* = 13.0 Hz, 1H), 4.07 (d, *J* = 13.0 Hz, 1H), 5.44 (s, 1H), 5.64−6.24 (br, 1H), 6.94−7.05 (m, 2H), 7.20−7.40 (m, 7H); $^{13}C$ NMR (100 MHz, $CDCl_3$) δ 13.9, 20.4, 26.0, 61.6, 70.2, 71.3, 114.9 (d, *J* = 21.3 Hz), 127.2 (d, *J* = 7.9 Hz), 127.6, 128.5, 129.4, 137.4, 137.8 (d, *J* = 3.0 Hz), 161.7 (d, *J* = 244.6 Hz); HRMS calcd for $[C_{18}H_{22}FNNaO_2]^+$ $(M + Na)^+$: 326.1527; found: 326.1525.

Minor diastereomer (**2e**, *syn*, more polar) as a white solid: mp 113.5−114.1 ºC; $[\alpha]_D^{20}$ = +23.0 (*c* 1.0, $CHCl_3$, 83% ee); IR (film) $\nu_{max}$: 3366, 2950, 1505, 1217 $cm^{-1}$; $^1H$ NMR (400 MHz, $CDCl_3$) δ 0.68 (t, *J* = 7.0 Hz, 3H), 0.72−0.85

(m, 1H), 0.95−1.19 (m, 2H), 1.68−1.81 (m, 1H), 2.57−2.69 (m, 1H), 3.81 (d, *J* = 13.2 Hz, 1H), 4.05 (d, *J* = 13.2 Hz, 1H), 4.43 (d, *J* = 9.1 Hz, 1H), 5.03−6.09 (br, 1H), 6.93−7.06 (m, 2H), 7.21−7.40 (m, 7H); $^{13}$C NMR (100 MHz, $CDCl_3$) δ 14.1, 21.4, 26.6, 61.0, 71.5, 74.6, 115.1 (d, *J* = 21.4 Hz), 127.4, 128.4, 129.1 (d, *J* = 8.1 Hz), 129.2, 136.9 (d, *J* = 2.8 Hz), 137.6, 162.4 (d, *J* = 245.9 Hz); HRMS calcd for $[C_{18}H_{22}FNNaO_2]^+$ $(M + Na)^+$: 326.1527; found: 326.1527.

### (1*S*,2*S*)-2-(Benzyl(hydroxy)amino)-1-(4-fluorophenyl)propan-1-ol (2f-1)

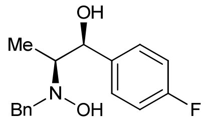


Following the **general procedure**, the asymmetric reductive coupling of nitrone **1f** (44.8 mg, 0.30 mmol, 1.0 equiv.) with 4-fluorobenzaldehyde (48 μL, 0.45 mmol, 1.5 equiv.) gave the desired product **2f-1** as the minor diastereomer [dr = 4.1/1 (*anti*/*syn*), determined by chiral HPLC analysis] of an inseparable diastereomeric mixture; major diastereomer (*anti*): 92% ee; minor diastereomer (*syn*): 42% ee [Lux® Amylose-1 column, Agilent HPLC, 30 ºC, hexane/EtOH = 90/10 (v/v), 1.0 mL/min, 220 nm; *anti*: $t_1$ = 13.9 min, $t_2$ = 17.8 min; *syn*: $t_1$ = 14.5 min, $t_2$ = 26.1 min]. Characterization data was obtained by chromatography on silica gel (eluent: EtOAc/PE = 1/6; 59.7 mg, 72 % combined yield): IR (film) $\nu_{max}$: 3426, 2913, 1604, 1510, 1222 cm$^{-1}$; HRMS calcd for $[C_{16}H_{18}FNNaO_2]^+$ $(M + Na)^+$: 298.1214; found: 298.1218.

Major diastereomer (*anti*, data read from spectrum of the diastereomeric mixture): $^1$H NMR (500 MHz, MeOD) δ 1.12 (d, *J* = 6.6 Hz, 3H), 2.95 (qd, *J* = 6.6, 4.7 Hz, 1H), 3.81 (d, *J* = 13.4 Hz, 1H), 4.01 (d, *J* = 13.4 Hz, 1H), 5.09 (d, *J* = 4.7 Hz, 1H), 6.99−7.08 (m, 2H), 7.17−7.44 (m, 7H); $^{13}$C NMR (125 MHz, MeOD) δ 8.8, 62.0, 67.6, 74.8, 115.4 (d, *J* = 21.5 Hz), 127.9, 129.0, 129.1 (d, *J* = 8.1 Hz), 130.2, 139.7, 140.5 (d, *J* = 3.0 Hz), 163.1 (d, *J* = 243.1 Hz).

Minor diastereomer (**2f-1**, *syn*, data read from spectrum of the diastereomeric mixture): $^1$H NMR (500 MHz, MeOD) δ 0.88 (d, *J* = 6.5 Hz, 3H), 2.88 (dq, *J* = 9.0, 6.5 Hz, 1H), 3.82 (d, *J* = 13.3 Hz, 1H), 4.04 (d, *J* = 13.3 Hz, 1H), 4.52 (d, *J* = 9.0 Hz, 1H), 6.99−7.08 (m, 2H), 7.17−7.44 (m, 7H); $^{13}$C NMR (125 MHz, MeOD) δ 7.9, 61.9, 67.8, 76.0, 115.9 (d, *J* = 21.3 Hz), 128.2, 129.2, 130.30, 130.32 (d, *J* = 8.1 Hz), 138.7 (d, *J* = 3.3 Hz), 139.5, 163.7 (d, *J* = 244.6 Hz).

### (1*S*,2*S*)-2-(Benzyl(hydroxy)amino)-2-cyclohexyl-1-(4-fluorophenyl)ethanol (2g)

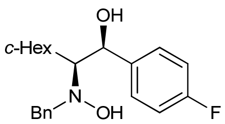


Following the **general procedure**, the asymmetric reductive coupling of nitrone **1g** (65.2 mg, 0.30 mmol, 1.0 equiv.) with 4-fluorobenzaldehyde (48 μL, 0.45 mmol, 1.5 equiv.) gave the desired product **2g** as the major diastereomer [dr = 1/3.0 (*anti*/*syn*), determined by $^1$H NMR integral area of crude product, $\delta_H$ 5.53 (*anti*), 4.88 (*syn*)]; major diastereomer (*syn*): 74% ee [Lux® Amylose-1 column, Shimadzu HPLC, 30 ºC, hexane/EtOH = 80/20 (v/v), 1.0 mL/min, 225 nm; $t_1$ = 6.7 min, $t_2$ = 13.3 min]; minor diastereomer (*anti*): 89% ee [Lux® Amylose-1 column, Agilent HPLC, 30 ºC, hexane/EtOH = 85/15 (v/v), 1.0 mL/min, 225 nm; $t_1$ = 6.7 min, $t_2$ = 7.6 min]. Characterization data was obtained by chromatography on silica gel (eluent: EtOAc/PE = 1/5; 89.6 mg, 87% combined yield).

Major diastereomer (**2g**, *syn*, more polar) as a white solid: mp 136.6−137.2 ºC; $[\alpha]_D^{20}$ = +42.7 (*c* 1.0, $CHCl_3$, 74% ee); IR (film) $\nu_{max}$: 3379, 2927, 2853, 1515, 1441, 1220 cm$^{-1}$; $^1$H NMR (400 MHz, $CDCl_3$) δ 0.76−0.89 (m, 1H), 0.92−1.05 (m, 1H), 1.07−1.21 (m, 2H), 1.25−1.35 (m, 1H), 1.53−1.81 (m, 6H), 2.71 (dd, *J* = 8.2, 2.6 Hz, 1H), 3.95 (d, *J* = 13.3 Hz, 1H), 4.07 (d, *J* = 13.3 Hz, 1H), 4.13−4.71 (br, 1H), 4.88 (d, *J* = 8.2 Hz, 1H), 4.95−5.32 (br, 1H), 6.95−7.12 (m, 2H), 7.21−7.41 (m, 7H); $^{13}$C NMR (100 MHz, $CDCl_3$) δ 26.4, 26.8, 27.0, 31.1, 31.6, 37.5, 62.9, 71.9, 75.1, 115.1 (d, *J* = 21.4 Hz), 127.4, 128.4, 129.1 (d, *J* = 8.2 Hz), 129.2, 138.1, 138.4 (d, *J* = 3.0 Hz), 162.3 (d, *J* =

245.9 Hz); HRMS calcd for $[C_{21}H_{26}FNNaO_2]^+$ $(M + Na)^+$: 366.1840; found: 366.1839.

Minor diastereomer (*anti*, less polar) as a white solid: mp 140.1−140.6 ºC; $[\alpha]_D^{25}$ = +5.0 (*c* 1.0, $CHCl_3$, 89% ee); IR (film) $\nu_{max}$: 3379, 2930, 2853, 1604, 1508, 1457, 1217 $cm^{-1}$; $^1H$ NMR (400 MHz, $CDCl_3$) δ 0.75−0.91 (m, 1H), 1.00−1.24 (m, 4H), 1.34−1.44 (m, 1H), 1.49−1.62 (m, 2H), 1.66−1.77 (m, 1H), 1.77−1.90 (m, 1H), 2.10−2.23 (m, 1H), 2.66 (dd, *J* = 6.4, 1.7 Hz, 1H), 3.36−3.56 (br, 1H), 3.93 (d, *J* = 13.1 Hz, 1H), 4.18 (d, *J* = 13.1 Hz, 1H), 4.90−5.09 (br, 1H), 5.48−5.58 (m, 1H), 6.96−7.06 (m, 2H), 7.25−7.40 (m, 7H); $^{13}C$ NMR (100 MHz, $CDCl_3$) δ 26.40, 26.43, 26.6, 31.2, 31.9, 36.9, 62.6, 70.5, 74.6, 114.8 (d, *J* = 21.3 Hz), 127.0 (d, *J* = 7.9 Hz), 127.5, 128.4, 129.2, 137.9, 139.1 (d, *J* = 3.0 Hz), 161.5 (d, *J* = 244.5 Hz); HRMS calcd for $[C_{21}H_{26}FNNaO_2]^+$ $(M + Na)^+$: 366.1840; found: 366.1840.

### (1*S*,2*S*)-2-(Benzyl(hydroxy)amino)-1-(4-fluorophenyl)-3,3-dimethylbutan-1-ol (2h)

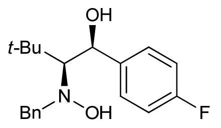


Following the **general procedure**, the asymmetric reductive coupling of nitrone **1h** (57.4 mg, 0.30 mmol, 1.0 equiv.) with 4-fluorobenzaldehyde (48 μL, 0.45 mmol, 1.5 equiv.) gave the desired product **2h** as the major diastereomer [dr = 1/5.0 (*anti*/*syn*), determined by $^1H$ NMR integral area of crude product, $\delta_H$ 5.57 (*anti*), 5.19 (*syn*)] with 38% ee [Lux® Amylose-1 column, Shimadzu HPLC, 30 ºC, hexane/EtOH = 80/20 (v/v), 1.0 mL/min, 230 nm; $t_1$ = 4.2 min, $t_2$ = 4.9 min]. Characterization data was obtained by chromatography on silica gel (eluent: EtOAc/PE = 1/6; 24.9 mg, 26% combined yield).

Major diastereomer (*syn*, more polar) as a white solid: mp 126.1−127.0 ºC; $[\alpha]_D^{20}$ = +93.8 (*c* 0.5, $CHCl_3$, 38% ee); IR (film) $\nu_{max}$: 3369, 2953, 1518, 1226 $cm^{-1}$; $^1H$ NMR (400 MHz, $CDCl_3$) δ 1.09 (s, 9H), 2.73 (d, *J* = 4.0 Hz, 1H), 3.61 (d, *J* = 13.5 Hz, 1H), 3.74 (d, *J* = 13.5 Hz, 1H), 4.45−4.76 (br, 1H), 5.03−5.29 (br, 1H), 5.19 (d, *J* = 4.0 Hz, 1H), 7.00−7.09 (m, 2H), 7.10−7.17 (m, 2H), 7.17−7.30 (m, 3H), 7.33−7.44 (m, 2H); $^{13}C$ NMR (100 MHz, $CDCl_3$) δ 28.9, 36.5, 65.2, 74.6, 76.8, 114.9 (d, *J* = 21.3 Hz), 127.2, 128.18 (d, *J* = 7.8 Hz), 128.19, 128.9, 138.7, 140.6 (d, *J* = 3.0 Hz), 161.9 (d, *J* = 245.0 Hz); HRMS calcd for $[C_{19}H_{24}FNNaO_2]^+$ $(M + Na)^+$: 340.1683; found: 340.1683.

### (1*S*,2*S*)-1-(4-Fluorophenyl)-2-(hydroxy(methyl)amino)-3-methylbutan-1-ol (2i)

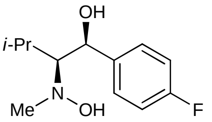


Following the **general procedure**, the asymmetric reductive coupling of nitrone **1i** (30.3 mg, 0.30 mmol, 1.0 equiv.) with 4-fluorobenzaldehyde (48 μL, 0.45 mmol, 1.5 equiv.) gave the desired product **2i** as the major diastereomer [dr = 1/5.5 (*anti*/*syn*), determined by $^1H$ NMR integral area of crude product, $\delta_H$ 5.48 (*anti*), 4.79 (*syn*)] with 81% ee [Lux® Amylose-1 column, Shimadzu HPLC, 30 ºC, hexane/EtOH = 90/10 (v/v), 1.0 mL/min, 220 nm; $t_1$ = 6.7 min, $t_2$ = 8.2 min]. Characterization data was obtained by chromatography on silica gel (eluent: EtOAc/PE = 1/2; 28.0 mg, 41% combined yield).

Major diastereomer (*syn*, more polar) as a colorless oil: $[\alpha]_D^{20}$ = +56.8 (*c* 0.5, $CHCl_3$, 81% ee); IR (film) $\nu_{max}$: 3375, 2966, 2917, 1611, 1505, 1217 $cm^{-1}$; $^1H$ NMR (400 MHz, $CDCl_3$) δ 0.72 (d, *J* = 7.2 Hz, 3H), 0.96 (d, *J* = 7.0 Hz, 3H), 1.90−2.03 (m, 1H), 2.61 (dd, *J* = 8.4, 2.5 Hz, 1H), 2.78 (s, 3H), 4.79 (d, *J* = 8.4 Hz, 1H), 4.93−5.78 (br, 2H), 6.98−7.09 (m, 2H), 7.29−7.40 (m, 2H); $^{13}C$ NMR (100 MHz, $CDCl_3$) δ 20.0, 21.1, 26.1, 46.3, 71.8, 76.2, 115.1 (d, *J* = 21.4 Hz), 129.1 (d, *J* = 8.1 Hz), 138.2 (d, *J* = 2.8 Hz), 162.3 (d, *J* = 245.7 Hz); HRMS calcd for $[C_{12}H_{18}FNNaO_2]^+$ $(M + Na)^+$: 250.1214; found: 250.1214.

**(1*S*,2*S*)-1-(4-Fluorophenyl)-2-(hydroxy(isopropyl)amino)-3-methylbutan-1-ol (2j)**

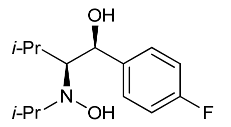


Following the **general procedure**, the asymmetric reductive coupling of nitrone **1j** (38.8 mg, 0.30 mmol, 1.0 equiv.) with 4-fluorobenzaldehyde (48 μL, 0.45 mmol, 1.5 equiv.) gave the desired product **2j** as the major diastereomer [dr = 1/8.0 (*anti*/*syn*), determined by $^{1}$H NMR integral area of crude product, $\delta_H$ 5.40 (*anti*), 4.75 (*syn*)] with 79% ee [Lux® Amylose-1 column, Shimadzu HPLC, 30 ºC, hexane/EtOH = 80/20 (v/v), 1.0 mL/min, 265 nm; $t_l$ = 4.4 min, $t_2$ = 5.2 min]. Characterization data was obtained by chromatography on silica gel (eluent: EtOAc/PE = 1/3; 64.6 mg, 84% combined yield).

Major diastereomer (*syn*, more polar) as a white solid: mp 70.5−71.3 ºC; $[\alpha]_D^{20}$ = +39.4 (*c* 1.0, $CHCl_3$, 79% ee); IR (film) $\nu_{max}$: 3372, 2962, 1614, 1505, 1210 $cm^{-1}$; $^{1}$H NMR (400 MHz, $CDCl_3$) δ 0.62 (d, *J* = 7.2 Hz, 3H), 0.99 (d, *J* = 7.0 Hz, 3H), 1.05 (d, *J* = 6.2 Hz, 3H), 1.11 (d, *J* = 6.0 Hz, 3H), 1.99−2.13 (m, 1H), 2.78−2.92 (m, 1H), 3.12−3.27 (m, 1H), 4.75 (d, *J* = 8.2 Hz, 1H), 4.85−5.57 (br, 1H), 5.57−6.37 (br, 1H), 6.95−7.10 (m, 2H), 7.29−7.44 (m, 2H); $^{13}$C NMR (100 MHz, $CDCl_3$) δ 19.8, 19.9, 21.2, 22.0, 25.7, 54.2, 70.1, 70.6, 114.9 (d, *J* = 21.4 Hz), 129.3 (d, *J* = 8.1 Hz), 138.3 (d, *J* = 2.6 Hz), 162.2 (d, *J* = 245.4 Hz); HRMS calcd for $[C_{14}H_{22}FNNaO_2]^+$ $(M + Na)^+$: 278.1527; found: 278.1527.

**(1*S*,2*S*)-2-(Benzyl(hydroxy)amino)-1-(3-fluorophenyl)-3-methylbutan-1-ol** (**2a-2**)

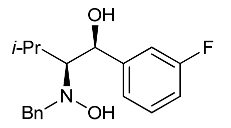


Following the **general procedure**, the asymmetric reductive coupling of nitrone **1a** (53.2 mg, 0.30 mmol, 1.0 equiv.) with 3-fluorobenzaldehyde (48 μL, 0.45 mmol, 1.5 equiv.) gave the desired product **2a-2** as the major diastereomer [dr = 1/7.2 (*anti*/*syn*), determined by $^{1}$H NMR integral area of crude product, $\delta_H$ 5.56 (*anti*), 4.84 (*syn*)] with 79% ee [Lux® Amylose-1 column, Shimadzu HPLC, 30 ºC, hexane/EtOH = 80/20 (v/v), 1.0 mL/min, 220 nm; $t_l$ = 10.4 min, $t_2$ = 13.5 min]. Characterization data was obtained by chromatography on silica gel (eluent: EtOAc/PE = 1/6; 83.5 mg, 92% combined yield).

Major diastereomer (*syn*, more polar) as a white solid: mp 84.4−85.0 ºC; $[\alpha]_D^{20}$ = +56.9 (*c* 1.0, $CHCl_3$, 79% ee); IR (film) $\nu_{max}$: 3369, 2962, 2911, 1617, 1595, 1457, 1249 $cm^{-1}$; $^{1}$H NMR (400 MHz, $CDCl_3$) δ 0.82 (d, *J* = 7.0 Hz, 3H), 0.99 (d, *J* = 6.9 Hz, 3H), 1.95−2.08 (m, 1H), 2.72 (dd, *J* = 7.6, 3.3 Hz, 1H), 3.91 (d, *J* = 13.3 Hz, 1H), 4.00 (d, *J* = 13.3 Hz, 1H), 4.08−4.68 (br, 1H), 4.84 (d, *J* = 7.6 Hz, 1H), 5.15−5.65 (br, 1H), 6.90−7.00 (m, 1H), 7.02−7.13 (m, 2H), 7.20−7.37 (m, 6H); $^{13}$C NMR (100 MHz, $CDCl_3$) δ 20.5, 20.7, 27.1, 63.1, 72.4, 74.7, 114.2 (d, *J* = 21.3 Hz), 114.4 (d, *J* = 20.9 Hz), 122.9 (d, *J* = 2.4 Hz), 127.4, 128.3, 129.2, 129.6 (d, *J* = 8.1 Hz), 138.1, 145.6 (d, *J* = 6.6 Hz), 162.8 (d, *J* = 245.7 Hz); HRMS calcd for $[C_{18}H_{22}FNNaO_2]^+$ $(M + Na)^+$: 326.1527; found: 326.1527.

**(1*S*,2*S*)-2-(Benzyl(hydroxy)amino)-1-(2-fluorophenyl)-3-methylbutan-1-ol** (**2a-3**)

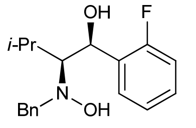


Following the **general procedure**, the asymmetric reductive coupling of nitrone **1a** (53.2 mg, 0.30 mmol, 1.0 equiv.) with 2-fluorobenzaldehyde (47 μL, 0.45 mmol, 1.5 equiv.) gave the desired product **2a-3** as the major diastereomer [dr = 1/12 (*anti*/*syn*), determined by $^{1}$H NMR integral area of crude product, $\delta_H$ 5.83 (*anti*), 5.24 (*syn*)] with 66% ee [Lux® Amylose-1 column, Shimadzu HPLC, 30 ºC, hexane/*i*-PrOH = 85/15 (v/v), 1.0 mL/min, 225 nm; $t_l$ = 5.5 min, $t_2$ = 6.8 min]. Characterization data was obtained by chromatography on silica gel (eluent: EtOAc/PE = 1/6; 39.2 mg, 43% combined yield).

Major diastereomer (*syn*, more polar) as a colorless oil: $[\alpha]_D^{20}$ = +59.8 (*c* 1.0, $CHCl_3$, 66% ee); IR (film) $\nu_{max}$: 3366, 2956, 1492, 1454 $cm^{-1}$; $^1H$ NMR (400 MHz, $CDCl_3$) δ 0.89 (d, *J* = 7.0 Hz, 3H), 1.00 (d, *J* = 6.9 Hz, 3H), 1.97−2.10 (m, 1H), 2.88 (dd, *J* = 7.0, 4.2 Hz, 1H), 3.87 (d, *J* = 13.4 Hz, 1H), 3.95 (d, *J* = 13.4 Hz, 1H), 4.09−4.54 (br, 1H), 5.24 (d, *J* = 7.0 Hz, 1H), 5.27−5.54 (br, 1H), 6.94−7.03 (m, 1H), 7.09−7.17 (m, 1H), 7.17−7.34 (m, 6H), 7.42−7.52 (m, 1H); $^{13}C$ NMR (100 MHz, $CDCl_3$) δ 20.5, 20.7, 27.5, 63.1, 67.5, 73.4, 115.2 (d, *J* = 22.2 Hz), 124.0 (d, *J* = 3.2 Hz), 127.2, 128.2, 128.9 (d, *J* = 8.3 Hz), 129.0, 129.1 (d, *J* = 4.4 Hz), 130.0 (d, *J* = 12.6 Hz), 138.3, 160.1 (d, *J* = 245.7 Hz); HRMS calcd for $[C_{18}H_{22}FNNaO_2]^+$ $(M + Na)^+$: 326.1527; found: 326.1528.

**(1*S*,2*S*)-2-(Benzyl(hydroxy)amino)-1-(4-methoxyphenyl)-3-methylbutan-1-ol (2a-4)**

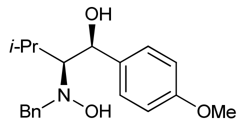


Following the **general procedure**, the asymmetric reductive coupling of nitrone **1a** (53.2 mg, 0.30 mmol, 1.0 equiv.) with 4-methoxybenzaldehyde (55 μL, 0.45 mmol, 1.5 equiv.) gave the desired product **2a-4** as the major diastereomer [dr = 1/6.5 (*anti*/*syn*), determined by $^1H$ NMR integral area of crude product, $\delta_H$ 5.49 (*anti*), 4.87 (*syn*)] with 76% ee [Lux® Amylose-1 column, Shimadzu HPLC, 30 ºC, hexane/EtOH = 80/20 (v/v), 1.0 mL/min, 225 nm; $t_1$ = 9.0 min, $t_2$ = 12.9 min]. Characterization data was obtained by chromatography on silica gel (eluent: EtOAc/PE = 1/4; 82.0 mg, 87% combined yield).

Major diastereomer (*syn*, more polar) as a white solid: mp 101.6−102.3 ºC; $[\alpha]_D^{20}$ = +53.9 (*c* 1.0, $CHCl_3$, 76% ee); IR (film) $\nu_{max}$: 3379, 2956, 2921, 1614, 1515, 1246 $cm^{-1}$; $^1H$ NMR (400 MHz, $CDCl_3$) δ 0.78 (d, *J* = 7.1 Hz, 3H), 0.98 (d, *J* = 6.9 Hz, 3H), 1.89−2.06 (m, 1H), 2.80 (dd, *J* = 8.6, 2.6 Hz, 1H), 3.79 (s, 3H), 4.03 (d, *J* = 13.4 Hz, 1H), 4.10 (d, *J* = 13.4 Hz, 1H), 4.87 (d, *J* = 8.6 Hz, 1H), 4.99−5.27 (br, 1H), 6.81−6.95 (m, 2H), 7.21−7.43 (m, 7H); $^{13}C$ NMR (100 MHz, $CDCl_3$) δ 20.59, 20.65, 27.0, 55.2, 63.2, 72.6, 74.9, 113.7, 127.3, 128.4, 128.7, 129.2, 134.5, 138.4, 159.2; HRMS calcd for $[C_{19}H_{25}NNaO_3]^+$ $(M + Na)^+$: 338.1727; found: 338.1725.

**(1*S*,2*S*)-2-(Benzyl(hydroxy)amino)-1-(3-methoxyphenyl)-3-methylbutan-1-ol (2a-5)**

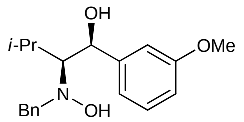


Following the **general procedure**, the asymmetric reductive coupling of nitrone **1a** (53.2 mg, 0.30 mmol, 1.0 equiv.) with 3-methoxybenzaldehyde (55 μL, 0.45 mmol, 1.5 equiv.) gave the desired product **2a-5** as the major diastereomer [dr = 1/5.7 (*anti*/*syn*), determined by $^1H$ NMR integral area of crude product, $\delta_H$ 5.54 (*anti*), 4.84 (*syn*)] with 82% ee [Lux® Amylose-1 column, Shimadzu HPLC, 30 ºC, hexane/EtOH = 80/20 (v/v), 1.0 mL/min, 220 nm; $t_1$ = 11.4 min, $t_2$ = 18.2 min]. Characterization data was obtained by chromatography on silica gel (eluent: EtOAc/PE = 1/4; 93.8 mg, 99% combined yield).

Major diastereomer (*syn*, more polar) as a colorless oil: $[\alpha]_D^{20}$ = +42.6 (*c* 1.0, $CHCl_3$, 82% ee); IR (film) $\nu_{max}$: 3366, 2959, 1604, 1454, 1258 $cm^{-1}$; $^1H$ NMR (400 MHz, $CDCl_3$) δ 0.81 (d, *J* = 7.0 Hz, 3H), 0.98 (d, *J* = 6.9 Hz, 3H), 1.90−2.06 (m, 1H), 2.77 (dd, *J* = 8.0, 3.0 Hz, 1H), 3.75 (s, 3H), 3.97 (d, *J* = 13.4 Hz, 1H), 4.03 (d, *J* = 13.4 Hz, 1H), 4.11−4.62 (br, 1H), 4.84 (d, *J* = 8.0 Hz, 1H), 5.27−5.71 (br, 1H), 6.76−6.85 (m, 1H), 6.87−6.98 (m, 2H), 7.17−7.38 (m, 6H); $^{13}C$ NMR (100 MHz, $CDCl_3$) δ 20.5, 20.6, 27.1, 55.1, 63.1, 72.9, 74.5, 112.99, 113.00, 119.8, 127.2, 128.2, 129.1 (2C), 138.3, 144.3, 159.5; HRMS calcd for $[C_{19}H_{25}NNaO_3]^+$ $(M + Na)^+$: 338.1727; found: 338.1725.

**(1*S*,2*S*)-2-(Benzyl(hydroxy)amino)-1-(2-methoxyphenyl)-3-methylbutan-1-ol (2a-6)**

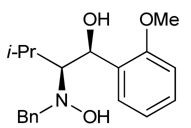

Following the **general procedure**, the asymmetric reductive coupling of nitrone **1a** (53.2 mg, 0.30 mmol, 1.0 equiv.) with 2-methoxybenzaldehyde (54 μL, 0.45 mmol, 1.5 equiv.) gave the desired product **2a-6** as the major diastereomer [dr = 1/15 (*anti*/*syn*), determined by $^1$H NMR integral area of crude product, $\delta_H$ 5.80 (*anti*), 5.34 (*syn*)] with 97% ee [Lux® Amylose-1 column, Shimadzu HPLC, 30 ºC, hexane/EtOH = 85/15 (v/v), 1.0 mL/min, 230 nm; $t_1$ = 6.4 min, $t_2$ = 9.0 min]. Characterization data was obtained by chromatography on silica gel (eluent: EtOAc/PE = 1/4; 58.7 mg, 62% combined yield).

Major diastereomer (*syn*, more polar) as a colorless oil: $[\alpha]_D^{20}$ = +105.5 (*c* 1.0, $CHCl_3$, 97% ee); IR (film) $\nu_{max}$: 3395, 2966, 1601, 1489, 1460, 1236, 1047 $cm^{-1}$; $^1$H NMR (400 MHz, $CDCl_3$) δ 0.93 (d, *J* = 6.9 Hz, 3H), 1.00 (d, *J* = 6.8 Hz, 3H), 1.92−2.06 (m, 1H), 2.96 (dd, *J* = 6.6, 4.6 Hz, 1H), 3.74 (s, 3H), 3.91 (s, 2H), 3.97−4.34 (br, 1H), 5.34 (d, *J* = 6.6 Hz, 1H), 5.36−5.57 (br, 1H), 6.78−6.85 (m, 1H), 6.93−7.00 (m, 1H), 7.16−7.29 (m, 6H), 7.41−7.49 (m, 1H); $^{13}$C NMR (100 MHz, $CDCl_3$) δ 20.5, 20.7, 27.8, 55.0, 63.2, 69.0, 72.4, 110.3, 120.5, 126.9, 128.1, 128.2, 128.4, 129.0, 131.2, 138.7, 156.4; HRMS calcd for $[C_{19}H_{25}NNaO_3]^+$ $(M + Na)^+$: 338.1727; found: 338.1725.

### (1*S*,2*S*)-2-(Benzyl(hydroxy)amino)-3-methyl-1-phenylbutan-1-ol (2a-7)

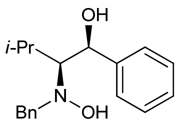

Following the **general procedure**, the asymmetric reductive coupling of nitrone **1a** (53.2 mg, 0.30 mmol, 1.0 equiv.) with benzaldehyde (46 μL, 0.45 mmol, 1.5 equiv.) gave the desired product **2a-7** as the major diastereomer [dr = 1/9.8 (*anti*/*syn*), determined by $^1$H NMR integral area of crude product, $\delta_H$ 5.56 (*anti*), 4.80 (*syn*)] with 92% ee [Lux® Amylose-1 column, Shimadzu HPLC, 30 ºC, hexane/EtOH = 80/20 (v/v), 1.0 mL/min, 220 nm; $t_1$ = 8.0 min, $t_2$ = 15.5 min]. Characterization data was obtained by chromatography on silica gel (eluent: EtOAc/PE = 1/5; 73.4 mg, 86% combined yield).

Major diastereomer (*syn*, more polar) as a white solid: mp 88.0−88.6 ºC; $[\alpha]_D^{20}$ = +83.5 (*c* 1.0, $CHCl_3$, 92% ee); IR (film) $\nu_{max}$: 3369, 2969, 2917, 1450, 1031 $cm^{-1}$; $^1$H NMR (400 MHz, $CDCl_3$) δ 0.74 (d, *J* = 7.1 Hz, 3H), 0.95 (d, *J* = 7.0 Hz, 3H), 1.88−2.04 (m, 1H), 2.74 (dd, *J* = 8.2, 2.9 Hz, 1H), 3.91 (d, *J* = 13.3 Hz, 1H), 4.00 (d, *J* = 13.3 Hz, 1H), 4.14−4.72 (br, 1H), 4.80 (d, *J* = 8.2 Hz, 1H), 5.46−6.02 (br, 1H), 7.16−7.41 (m, 10H); $^{13}$C NMR (100 MHz, $CDCl_3$) δ 20.5, 20.7, 26.8, 62.8, 72.8, 74.4, 127.1, 127.4, 127.5, 128.1, 128.2, 129.1, 138.2, 142.4; HRMS calcd for $[C_{18}H_{23}NNaO_2]^+$ $(M + Na)^+$: 308.1621; found: 308.1618.

### (1*S*,2*S*)-2-(Benzyl(hydroxy)amino)-1-(4-bromophenyl)-3-methylbutan-1-ol (2a-8)

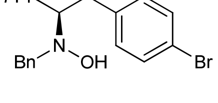

Following the **general procedure**, the asymmetric reductive coupling of nitrone **1a** (53.2 mg, 0.30 mmol, 1.0 equiv.) with 4-bromobenzaldehyde (83.2 mg, 0.45 mmol, 1.5 equiv.) gave the desired product **2a-8** as the major diastereomer [dr = 1/7.7 (*anti*/*syn*), determined by $^1$H NMR integral area of crude product, $\delta_H$ 5.42 (*anti*), 4.83 (*syn*)] with 91% ee [Lux® Amylose-1 column, Agilent HPLC, 30 ºC, hexane/EtOH = 85/15 (v/v), 1.0 mL/min, 220 nm; $t_1$ = 9.5 min, $t_2$ = 19.6 min]. Characterization data was obtained by chromatography on silica gel (eluent: EtOAc/PE = 1/6; 85.7 mg, 78% combined yield).

Major diastereomer (*syn*, more polar) as a white solid: mp 104.4−105.1 ºC; $[\alpha]_D^{25}$ = +78.5 (*c* 2.0, $CHCl_3$, 91% ee); IR (film) $\nu_{max}$: 3341, 2960, 2917, 1488, 1385, 1071, 1010 $cm^{-1}$; $^1$H NMR (500 MHz, $CDCl_3$) δ 0.81 (d, *J* = 7.1 Hz, 3H), 0.99 (d, *J* = 6.9 Hz, 3H), 1.96−2.06 (m, 1H), 2.73 (dd, *J* = 8.0, 3.2 Hz, 1H), 3.84−4.62 (br, 1H), 3.95 (d, *J* =

13.3 Hz, 1H), 4.04 (d, $J$ = 13.3 Hz, 1H), 4.83 (d, $J$ = 8.0 Hz, 1H), 5.04−5.39 (br, 1H), 7.19−7.23 (m, 2H), 7.24−7.35 (m, 5H), 7.42−7.47 (m, 2H); $^{13}$C NMR (125 MHz, $CDCl_3$) δ 20.6, 20.8, 27.0, 63.1, 72.3, 74.8, 121.4, 127.4, 128.4, 129.1, 129.2, 131.4, 138.1, 141.8; HRMS calcd for $[C_{18}H_{22}BrNNaO_2]^+$ $(M + Na)^+$: 386.0726; found: 386.0728.

**(1*S*,2*S*)-2-(Benzyl(hydroxy)amino)-3-methyl-1-(4-(methylthio)phenyl)butan-1-ol** (**2a-9**)

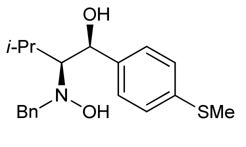


Following the **general procedure**, the asymmetric reductive coupling of nitrone **1a** (53.2 mg, 0.30 mmol, 1.0 equiv.) with 4-(methylthio)benzaldehyde (60 μL, 0.45 mmol, 1.5 equiv.) gave the desired product **2a-9** as the major diastereomer [dr = 1/8.5 (*anti*/*syn*), determined by $^1$H NMR integral area of crude product, $\delta_H$ 5.51 (*anti*), 4.79 (*syn*)] with 90% ee [Lux® Amylose-1 column, Shimadzu HPLC, 30 ºC, hexane/EtOH = 80/20 (v/v), 1.0 mL/min, 268 nm; $t_1$ = 10.1 min, $t_2$ = 15.8 min]. Characterization data was obtained by chromatography on silica gel (eluent: EtOAc/PE = 1/5; 79.7 mg, 80% combined yield).

Major diastereomer (*syn*, more polar) as a white solid: mp 105.7−106.2 ºC; $[\alpha]_D^{20}$ = +85.2 ($c$ 1.0, $CHCl_3$, 90% ee); IR (film) $\nu_{max}$: 3369, 2962, 2917, 1499, 1085 cm$^{-1}$; $^1$H NMR (400 MHz, $CDCl_3$) δ 0.76 (d, $J$ = 7.1 Hz, 3H), 0.96 (d, $J$ = 6.9 Hz, 3H), 1.91−2.03 (m, 1H), 2.44 (s, 3H), 2.73 (dd, $J$ = 8.4, 2.8 Hz, 1H), 3.96 (d, $J$ = 13.3 Hz, 1H), 4.04 (d, $J$ = 13.3 Hz, 1H), 4.14−4.63 (br, 1H), 4.79 (d, $J$ = 8.4 Hz, 1H), 5.32−5.72 (br, 1H), 7.16−7.36 (m, 9H); $^{13}$C NMR (100 MHz, $CDCl_3$) δ 15.8, 20.5, 20.7, 26.8, 63.0, 72.4, 74.5, 126.4, 127.2, 128.0, 128.3, 129.1, 137.6, 138.2, 139.3; HRMS calcd for $[C_{19}H_{25}NNaO_2S]^+$ $(M + Na)^+$: 354.1498; found: 354.1497.

***N*-(4-((1*S*,2*S*)-2-(Benzyl(hydroxy)amino)-1-hydroxy-3-methylbutyl)phenyl)acetamide** (**2a-10**)

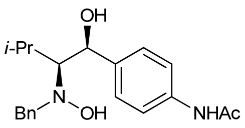


Following the **general procedure**, the asymmetric reductive coupling of nitrone **1a** (53.2 mg, 0.30 mmol, 1.0 equiv.) with *N*-(4-formylphenyl)acetamide (73.4 mg, 0.45 mmol, 1.5 equiv.) gave the desired product **2a-10** as the major diastereomer [dr = 1/5.3 (*anti*/*syn*), determined by $^1$H NMR integral area of crude product, $\delta_H$ 5.53 (*anti*), 4.97 (*syn*)] with 82% ee [Lux® Amylose-1 column, Shimadzu HPLC, 30 ºC, hexane/EtOH = 80/20 (v/v), 1.0 mL/min, 240 nm; $t_1$ = 7.8 min, $t_2$ = 10.9 min]. Characterization data was obtained by chromatography on silica gel (eluent: EtOAc/PE = 2/1; 102.1 mg, 99% combined yield).

Major diastereomer (*syn*, more polar) as a white solid: mp 89.9−90.6 ºC; $[\alpha]_D^{20}$ = +72.8 ($c$ 1.0, $CHCl_3$, 82% ee); IR (film) $\nu_{max}$: 3321, 2953, 2924, 1665, 1515, 1402 cm$^{-1}$; $^1$H NMR (400 MHz, MeOD) δ 0.82 (d, $J$ = 7.0 Hz, 3H), 0.98 (d, $J$ = 6.9 Hz, 3H), 1.86−1.98 (m, 1H), 2.10 (s, 3H), 2.82 (dd, $J$ = 8.2, 3.2 Hz, 1H), 4.08 (s, 2H), 4.97 (d, $J$ = 8.2 Hz, 1H), 7.19−7.26 (m, 1H), 7.26−7.41 (m, 6H), 7.51−7.59 (m, 2H); $^{13}$C NMR (100 MHz, MeOD) δ 20.8, 21.3, 23.8, 28.4, 64.4, 74.1, 75.2, 120.8, 128.0, 129.1 (2C), 130.3, 139.3, 139.9, 140.4, 171.5; HRMS calcd for $[C_{20}H_{26}N_2NaO_3]^+$ $(M + Na)^+$: 365.1836; found: 365.1834.

**(1*S*,2*S*)-2-(Benzyl(hydroxy)amino)-3-methyl-1-(thiophen-3-yl)butan-1-ol** (**2a-11**)

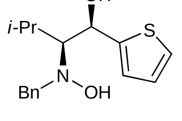


Following the **general procedure**, the asymmetric reductive coupling of nitrone **1a** (53.2 mg, 0.30 mmol, 1.0 equiv.) with thiophene-2-carbaldehyde (41 μL, 0.45 mmol, 1.5 equiv.) gave the desired product **2a-11** as the major diastereomer [dr = 1/6.2 (*anti*/*syn*), determined by $^1$H NMR integral area of crude product, $\delta_H$ 5.63 (*anti*), 5.13 (*syn*)] with 73% ee [Lux® Amylose-1 column,

Shimadzu HPLC, 30 ºC, hexane/EtOH = 80/20 (v/v), 1.0 mL/min, 235 nm; $t_1$ = 11.3 min, $t_2$ = 17.1 min]. Characterization data was obtained by chromatography on silica gel (eluent: EtOAc/PE = 1/6; 63.8 mg, 73% combined yield).

Major diastereomer (*syn*, more polar) as a white solid: mp 85.9−86.5 ºC; $[\alpha]_D^{20}$ = +35.1 (*c* 1.0, $CHCl_3$, 73% ee); IR (film) $\nu_{max}$: 3369, 2965, 2917, 1460, 1364 $cm^{-1}$; $^1H$ NMR (400 MHz, $CDCl_3$) δ 0.82 (d, *J* = 7.1 Hz, 3H), 1.01 (d, *J* = 6.9 Hz, 3H), 1.98−2.12 (m, 1H), 2.79 (dd, *J* = 8.2, 2.8 Hz, 1H), 3.98 (d, *J* = 13.3 Hz, 1H), 4.07 (d, *J* = 13.3 Hz, 1H), 4.21−4.79 (br, 1H), 5.13 (d, *J* = 8.2 Hz, 1H), 5.19−5.56 (br, 1H), 6.89−6.95 (m, 1H), 6.95−7.01 (m, 1H), 7.17−7.40 (m, 6H); $^{13}C$ NMR (100 MHz, $CDCl_3$) δ 20.3, 20.6, 27.0, 63.0, 68.7, 75.3, 125.0, 125.2, 126.2, 127.3, 128.3, 129.2, 138.1, 146.6; HRMS calcd for $[C_{16}H_{21}NNaO_2S]^+$ $(M + Na)^+$: 314.1185; found: 314.1184.

**(*S*)-5-((*S*)-(4-Fluorophenyl)(hydroxy)methyl)-2,2-dimethylpyrrolidin-1-ol (2k-1)**

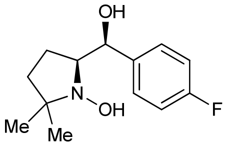


Following the **modified method 1**, the asymmetric reductive coupling of nitrone **1k** (33 μL, 0.30 mmol, 1.0 equiv.) with 4-fluorobenzaldehyde (48 μL, 0.45 mmol, 1.5 equiv.) gave the desired product **2k-1** as the major diastereomer [dr = 1/1.9 (*anti*/*syn*), determined by $^1H$ NMR integral area of crude product, $\delta_H$ 5.02 (*anti*), 4.70 (*syn*)]; major diastereomer (*syn*): 94% ee [Lux® Amylose-1 column, Shimadzu HPLC, 30 ºC, hexane/EtOH = 80/20 (v/v), 1.0 mL/min, 270 nm; $t_1$ = 7.7 min, $t_2$ = 8.5 min]; minor diastereomer (*anti*): 41% ee [Lux® Amylose-1 column, Agilent HPLC, 30 ºC, hexane/EtOH = 85/15 (v/v), 1.0 mL/min, 270 nm; $t_1$ = 7.7 min, $t_2$ = 9.5 min]. Characterization data was obtained by chromatography on silica gel (eluent: EtOAc/PE = 1/2; 51.6 mg, 72% combined yield).

Following the **modified method 2**, the desired product **2k-1** was obtained as the major diastereomer [dr < 1/20 (*anti*/*syn*)] in 84% combined yield (60.3 mg) with 92% ee.

Major diastereomer (**2k-1**, *syn*, more polar) as a colorless oil: $[\alpha]_D^{20}$ = +27.1 (*c* 1.0, $CHCl_3$, 94% ee); IR (film) $\nu_{max}$: 3324, 2972, 2933, 2873, 1604, 1511, 1220, 1153, 1149 $cm^{-1}$; $^1H$ NMR (400 MHz, $CDCl_3$) δ 1.09 (s, 3H), 1.24 (s, 3H), 1.28−1.37 (m, 1H), 1.47−1.64 (m, 3H), 3.26−3.36 (m, 1H), 4.70 (d, *J* = 8.2 Hz, 1H), 4.79−6.48 (br, 2H), 6.98−7.06 (m, 2H), 7.31−7.39 (m, 2H); $^{13}C$ NMR (100 MHz, $CDCl_3$) δ 19.8, 23.0, 26.4, 34.2, 65.4, 69.5, 77.5, 115.2 (d, *J* = 21.3 Hz), 128.5 (d, *J* = 8.0 Hz), 138.1 (d, *J* = 3.0 Hz), 162.3 (d, *J* = 245.5 Hz); HRMS calcd for $[C_{13}H_{19}FNO_2]^+$ $(M + H)^+$: 240.1394; found: 240.1396.

Minor diastereomer (*anti*, less polar) as a white solid: mp 87.2−87.5 ºC; $[\alpha]_D^{25}$ = +16.5 (*c* 1.0, $CHCl_3$, 41% ee); IR (film) $\nu_{max}$: 3369, 2930, 2869, 2873, 1604, 1220, 1149 $cm^{-1}$; $^1H$ NMR (400 MHz, $CDCl_3$) δ 1.06 (s, 3H), 1.22 (s, 3H), 1.30−1.39 (m, 1H), 1.44−1.70 (m, 3H), 3.22 (ddd, *J* = 9.7, 7.0, 2.5 Hz, 1H), 3.48−4.60 (br, 2H), 5.02 (d, *J* = 1.6 Hz, 1H), 6.98−7.08 (m, 2H), 7.31−7.42 (m, 2H); $^{13}C$ NMR (100 MHz, $CDCl_3$) δ 16.6, 18.0, 26.7, 34.3, 63.9, 69.5, 70.1, 115.0 (d, *J* = 21.2 Hz), 127.2 (d, *J* = 7.8 Hz), 136.8 (d, *J* = 2.9 Hz), 161.9 (d, *J* = 245.0 Hz); HRMS calcd for $[C_{13}H_{19}FNO_2]^+$ $(M + H)^+$: 240.1394; found: 240.1397.

**(*S*)-5-((*S*)-Hydroxy(phenyl)methyl)-2,2-dimethylpyrrolidin-1-ol (2k-2)**

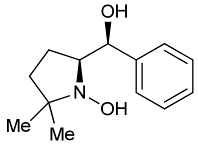


Following the **modified method 1**, the asymmetric reductive coupling of nitrone **1k** (33 μL, 0.30 mmol, 1.0 equiv.) with benzaldehyde (46 μL, 0.45 mmol, 1.5 equiv.) gave the desired product **2k-2** as the major diastereomer [dr = 1/1.9 (*anti*/*syn*), determined by $^1H$ NMR integral area of crude product, $\delta_H$ 5.05 (*anti*), 4.70 (*syn*)]; major diastereomer (*syn*): 92% ee [Lux®

Amylose-1 column, Shimadzu HPLC, 30 ºC, hexane/EtOH = 80/20 (v/v), 1.0 mL/min, 220 nm; $t_1$ = 8.1 min, $t_2$ = 8.8 min]; minor diastereomer (*anti*): 36% ee [Lux® Amylose-1 column, Agilent HPLC, 30 ºC, hexane/EtOH = 95/5 (v/v), 1.0 mL/min, 220 nm; $t_1$ = 17.8 min, $t_2$ = 19.2 min]. Characterization data was obtained by chromatography on silica gel (eluent: EtOAc/PE = 1/2; 48.5 mg, 73% combined yield).

Following the **modified method 2**, the desired product **2k-2** was obtained as the major diastereomer [dr < 1/20 (*anti*/*syn*)] in 76% combined yield (50.3 mg) with 87% ee.

Major diastereomer (**2k-2**, *syn*, more polar) as a colorless oil: $[\alpha]_D^{20}$ = +32.8 (*c* 1.0, $CHCl_3$, 92% ee); IR (film) $\nu_{max}$: 3334, 2972, 2927, 2869, 1454, 1079 $cm^{-1}$; $^1H$ NMR (400 MHz, $CDCl_3$) δ 1.08 (s, 3H), 1.24 (s, 3H), 1.38−1.48 (m, 1H), 1.48−1.68 (m, 3H), 3.29−3.39 (m, 1H), 4.70 (d, *J* = 7.2 Hz, 1H), 7.24−7.42 (m, 5H); $^{13}C$ NMR (100 MHz, $CDCl_3$) δ 19.8, 23.0, 26.4, 34.3, 65.3, 69.6, 78.1, 126.9, 127.7, 128.4, 142.3; HRMS calcd for $[C_{13}H_{20}NO_2]^+$ $(M + H)^+$: 222.1489; found: 222.1488.

Minor diastereomer (*anti*, less polar) as a white solid: mp 65.6−66.6 ºC; $[\alpha]_D^{25}$ = +17.7 (*c* 1.0, $CHCl_3$, 36% ee); IR (film) $\nu_{max}$: 3401, 2965, 2869, 1450, 1361, 1194 $cm^{-1}$; $^1H$ NMR (400 MHz, $CDCl_3$) δ 1.06 (s, 3H), 1.23 (s, 3H), 1.32−1.60 (m, 3H), 1.64−1.76 (m, 1H), 3.26 (ddd, *J* = 9.8, 7.1, 2.5 Hz, 1H), 3.37−4.74 (br, 2H), 5.05 (d, *J* = 1.8 Hz, 1H), 7.20−7.28 (m, 1H), 7.28−7.36 (m, 2H), 7.36−7.44 (m, 2H); $^{13}C$ NMR (100 MHz, $CDCl_3$) δ 16.7, 18.0, 26.8, 34.4, 63.8, 69.5, 70.6, 125.6, 126.9, 128.1, 141.2; HRMS calcd for $[C_{13}H_{20}NO_2]^+$ $(M + H)^+$: 222.1489; found: 222.1488.

**(*S*)-5-((*S*)-Hydroxy(4-(methylthio)phenyl)methyl)-2,2-dimethylpyrrolidin-1-ol** (**2k-3**)

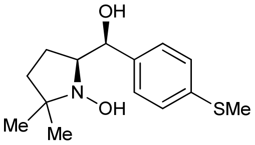


Following the **modified method 1**, the asymmetric reductive coupling of nitrone **1k** (33 µL, 0.30 mmol, 1.0 equiv.) with 4-(methylthio)benzaldehyde (60 µL, 0.45 mmol, 1.5 equiv.) gave the desired product **2k-3** as the major diastereomer [dr = 1/2.0 (*anti*/*syn*), determined by $^1H$ NMR integral area of crude product, $\delta_H$ 5.01 (*anti*), 4.67 (*syn*)]; major diastereomer (*syn*): 95% ee [Lux® Amylose-1 column, Shimadzu HPLC, 30 ºC, hexane/EtOH = 80/20 (v/v), 1.0 mL/min, 270 nm; $t_1$ = 12.0 min, $t_2$ = 16.3 min]; minor diastereomer (*anti*): 49% ee [Lux® Amylose-1 column, Agilent HPLC, 30 ºC, hexane/EtOH = 85/15 (v/v), 1.0 mL/min, 270 nm; $t_1$ = 13.5 min, $t_2$ = 21.3 min]. Characterization data was obtained by chromatography on silica gel (eluent: EtOAc/PE = 1/2; 75.6 mg, 94% combined yield).

Following the **modified method 2**, the desired product **2k-3** was obtained as the major diastereomer [dr < 1/20 (*anti*/*syn*)] in 94% combined yield (75.2 mg) with 89% ee.

Major diastereomer (**2k-3**, *syn*, more polar) as a colorless oil: $[\alpha]_D^{20}$ = +33.6 (*c* 1.0, $CHCl_3$, 95% ee); IR (film) $\nu_{max}$: 3403, 2965, 2834, 1514, 1248, 1035, 1001 $cm^{-1}$; $^1H$ NMR (400 MHz, $CDCl_3$) δ 1.08 (s, 3H), 1.24 (s, 3H), 1.28−1.37 (m, 1H), 1.45−1.63 (m, 3H), 2.46 (s, 3H), 3.25−3.41 (m, 1H), 4.67 (d, *J* = 8.5 Hz, 1H), 5.01−7.01 (br, 2H), 7.16−7.25 (m, 2H), 7.25−7.34 (m, 2H); $^{13}C$ NMR (100 MHz, $CDCl_3$) δ 15.8, 20.0, 23.1, 26.2, 34.2, 65.5, 69.5, 77.6, 126.5, 127.4, 137.6, 139.2; HRMS calcd for $[C_{14}H_{22}NO_2S]^+$ $(M + H)^+$: 268.1366; found: 268.1366.

Minor diastereomer (*anti*, less polar) as a white solid: mp 110.5−111.1 ºC; $[\alpha]_D^{25}$ = +21.5 (*c* 1.0, $CHCl_3$, 49% ee); IR (film) $\nu_{max}$: 3403, 2965, 2834, 1514, 1248, 1172, 1035 $cm^{-1}$; $^1H$ NMR (400 MHz, $CDCl_3$) δ 1.05 (s, 3H), 1.22 (s, 3H), 1.30−1.41 (m, 1H), 1.41−1.63 (m, 2H), 1.63−1.76 (m, 1H), 2.47 (s, 3H), 3.22 (ddd, *J* = 9.7, 7.2, 2.3 Hz, 1H), 3.68−4.97 (br, 2H), 5.01 (d, *J* = 1.8 Hz, 1H), 7.19−7.28 (m, 2H), 7.28−7.37 (m, 2H); $^{13}C$ NMR (100 MHz, $CDCl_3$) δ 16.0, 16.6, 18.0, 26.8, 34.3, 63.8, 69.5, 70.3, 126.2, 126.6, 136.7, 138.3; HRMS calcd for $[C_{14}H_{22}NO_2S]^+$ (M +

H)$^+$: 268.1366; found: 268.1367.

**(*S*)-2-((*S*)-(4-Fluorophenyl)(hydroxy)methyl)pyrrolidin-1-ol (2l)**

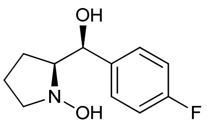


Following the **modified method 1**, the asymmetric reductive coupling of nitrone **1l** (0.30 mmol, 1.0 equiv.) with 4-fluorobenzaldehyde (48 µL, 0.45 mmol, 1.5 equiv.) gave the desired product **2l** as the major diastereomer [dr = 1/2.1 (*anti*/*syn*), determined by chiral HPLC analysis]; major diastereomer (*syn*): 97% ee [Lux® Amylose-1 column, Agilent HPLC, 30 ºC, hexane/EtOH = 70/30 (v/v), 1.0 mL/min, 270 nm; $t_1$ = 10.4 min, $t_2$ = 13.2 min]; minor diastereomer (*anti*): 4% ee [Lux® Amylose-1 column, Agilent HPLC, 30 ºC, hexane/EtOH = 85/15 (v/v), 1.0 mL/min, 270 nm; $t_1$ = 10.6 min, $t_2$ = 18.1 min]. Characterization data was obtained by chromatography on silica gel (eluent: EtOAc; 45.6 mg, 72% combined yield).

Following the **modified method 2**, the desired product **2l** was obtained as the major diastereomer [dr = 1/12 (*anti*/*syn*)] in 64% combined yield (40.6 mg) with 72% ee.

Major diastereomer (**2l**, *syn*, more polar) as a colorless oil: $[\alpha]_D^{20}$ = +6.7 (*c* 1.0, MeOH, 97% ee); IR (film) $\nu_{max}$: 3350, 2966, 2873, 1611, 1505, 1223 cm$^{-1}$; $^1$H NMR (400 MHz, $CDCl_3$) δ 1.22−1.34 (m, 1H), 1.38−1.56 (m, 1H), 1.61−1.89 (m, 2H), 2.95 (ddd, apparent dt, *J* = 11.0, 8.6 Hz, 1H), 3.09−3.36 (m, 2H), 4.30−4.90 (m, 1H), 6.59−7.23 (br, 2H), 6.94−7.07 (m, 2H), 7.26−7.40 (m, 2H); $^{13}$C NMR (100 MHz, $CDCl_3$) δ 21.1, 25.7, 58.6 (2C), 74.5, 115.2 (d, *J* = 21.3 Hz), 128.6 (d, *J* = 8.0 Hz), 137.7 (d, *J* = 2.9 Hz), 162.3 (d, *J* = 246.1 Hz); HRMS calcd for $[C_{11}H_{14}FNNaO_2]^+$ (M + Na)$^+$: 234.0901; found: 234.0902.

Minor diastereomer (*anti*, less polar) as a colorless oil: $[\alpha]_D^{25}$ = +1.6 (*c* 1.0, $CHCl_3$, 4% ee); IR (film) $\nu_{max}$: 3372, 2972, 2860, 1611, 1511, 1226 cm$^{-1}$; $^1$H NMR (400 MHz, $CDCl_3$) δ 1.35−1.48 (m, 1H), 1.57−1.93 (m, 3H), 2.83 (ddd, apparent q, *J* = 9.5 Hz, 1H), 2.99 (ddd, apparent td, *J* = 8.9, 1.6 Hz, 1H), 3.30 (ddd, *J* = 9.5, 7.3, 2.0 Hz, 1H), 5.08−5.23 (m, 1H), 5.51−6.54 (br, 2H), 6.94−7.07 (m, 2H), 7.27−7.40 (m, 2H); $^{13}$C NMR (100 MHz, $CDCl_3$) δ 19.3, 19.4, 57.8, 69.9, 73.8, 115.0 (d, *J* = 21.3 Hz), 127.2 (d, *J* = 7.9 Hz), 137.1 (d, *J* = 3.0 Hz), 161.9 (d, *J* = 245.1 Hz); HRMS calcd for $[C_{11}H_{14}FNNaO_2]^+$ (M + Na)$^+$: 234.0901; found: 234.0902.

**(*S*)-2-((*S*)-(4-Fluorophenyl)(hydroxy)methyl)piperidin-1-ol (2m)**

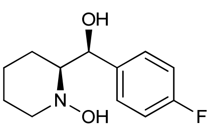


Following the **modified method 1**, the asymmetric reductive coupling of nitrone **1m** (0.30 mmol, 1.0 equiv.) with 4-fluorobenzaldehyde (48 µL, 0.45 mmol, 1.5 equiv.) gave the desired product **2m** as the major diastereomer [dr = 1/9.9 (*anti*/*syn*), determined by chiral HPLC analysis] with 97% ee [Lux® Amylose-1 column, Shimadzu HPLC, 30 ºC, hexane/EtOH = 80/20 (v/v), 1.0 mL/min, 270 nm; $t_1$ = 8.2 min, $t_2$ = 8.6 min]. Characterization data was obtained by chromatography on silica gel (eluent: EtOAc; 39.0 mg, 58% combined yield).

Following the **modified method 2**, the desired product **2m** was obtained as the major diastereomer [dr = 1/20 (*anti*/*syn*)] in 47% combined yield (31.8 mg) with 57% ee.

Major diastereomer (*syn*, more polar) as a white solid: mp 122.9−123.6 ºC; $[\alpha]_D^{20}$ = −15.6 (*c* 1.0, MeOH, 97% ee); IR (film) $\nu_{max}$: 3366, 2921, 2850, 1601, 1511, 1223 cm$^{-1}$; $^1$H NMR (400 MHz, DMSO-d6, 25 ºC) δ 0.31−0.77 (m, 1H), 0.91−1.22 (m, 2H), 1.39−1.67 (m, 3H), 2.34−2.60 (m, 2H), 3.08−3.25 (m, 1H), 4.88−5.52 (m, 2H), 7.02−7.19 (m, 2H), 7.38−7.52 (m, 2H), 7.82−8.17 (br, 1H); $^{13}$C NMR (100 MHz, DMSO-d6, 25 ºC) δ 23.0, 24.7, 25.1, 60.0,

70.4, 72.0, 113.6 (d, $J$ = 20.0 Hz), 128.9 (d, $J$ = 7.6 Hz), 138.6 (d, $J$ = 2.3 Hz), 161.0 (d, $J$ = 241.4 Hz); $^1$H NMR (400 MHz, DMSO-d6, 75 ºC) δ 0.78−0.96 (m, 1H), 1.05−1.17 (m, 1H), 1.34−1.56 (m, 4H), 2.49−2.60 (m, 2H), 3.13−3.24 (m, 1H), 4.68−5.07 (br, 1H), 4.95 (d, $J$ = 4.9 Hz, 1H), 6.99−7.13 (m, 2H), 7.38−7.48 (m, 2H), 7.48−7.80 (br, 1H); $^{13}$C NMR (100 MHz, DMSO-d6, 75 ºC) δ 22.5, 23.3, 23.9, 58.6, 71.0, 71.8, 113.2 (d, $J$ = 20.9 Hz), 128.4 (d, $J$ = 7.8 Hz), 138.3 (d, $J$ = 3.0 Hz), 160.7 (d, $J$ = 241.9 Hz); HRMS calcd for $[C_{12}H_{16}FNNaO_2]^+$ (M + Na)$^+$: 248.1057; found: 248.1059.

# Supplementary Discussion:

## Determination of the Absolute Configuration of Vicinal Amino Alcohols:

In order to determinate the relative stereochemistry of major diastereomers in Table 2, under a general chiral ligand free condition (as demonstrated in Supplementary Table 2, entry 1), reductive coupling of nitrone **1n** with benzaldehyde provided desired vicinal hydroxyamino alcohol as a separable mixture of diastereomers in 66% combined yield with 2.1/1 dr. The stereochemistry of major diastereomer (±)-**2n** was assigned as 1,2-*anti* according to the observed vicinal coupling constant[10] ($J_{1,2}$ = 0 Hz, as to minor diastereomer $J_{1,2}$ = 9.0 Hz). Indium-mediated reduction[11] of (±)-**2n** afforded racemic (±)-ephedrine [(±)-**6n**] in 90% yield and it also confirmed the 1,2-*anti* configuration of (±)-**2n** (Supplementary Fig. 1). However, most of the observed vicinal coupling constants ($J_{1,2}$) of major diastereomers in Table 2 were higher than 7.0 Hz. So, we speculated that the relative stereochemistry of the major diastereomers in Table 2 should be 1,2-*syn* configuration.

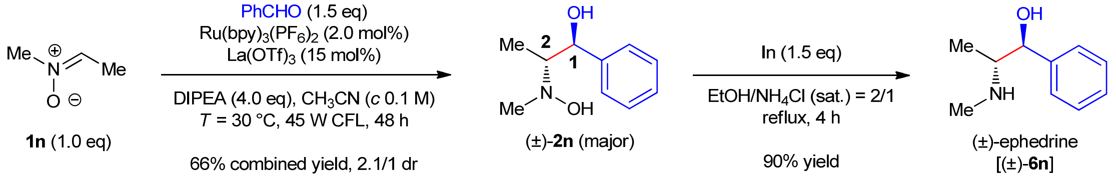


***Supplementary Figure 1*. Concise synthesis of (±)-ephedrine [(±)-6n].** Reductive coupling of nitrone **1n** with benzaldehyde under a general chiral ligand free condition gave (±)-**2n** as the major diastereomer (2.1/1 dr). Indium-mediated reduction of (±)-**2n** gave (±)-**6n** in 40% yield for two steps, which also confirmed the 1,2-*anti* configuration of (±)-**2n**.

Furthermore, indium-mediated reduction of vicinal hydroxyamino alcohols **2a-9**, **2b-3** and **2k-3** followed by acidification afforded hydrochlorides of vicinal amino alcohols **6a-9**, **6b-3** and **6k-3**. Their absolute configurations were determined to be (1*S*,2*S*)-**6a-9**, (*S*)-**6b-3** and (1*S*,2*S*)-**6k-3** by single-crystal X-ray diffraction analysis (Supplementary Fig. 2).

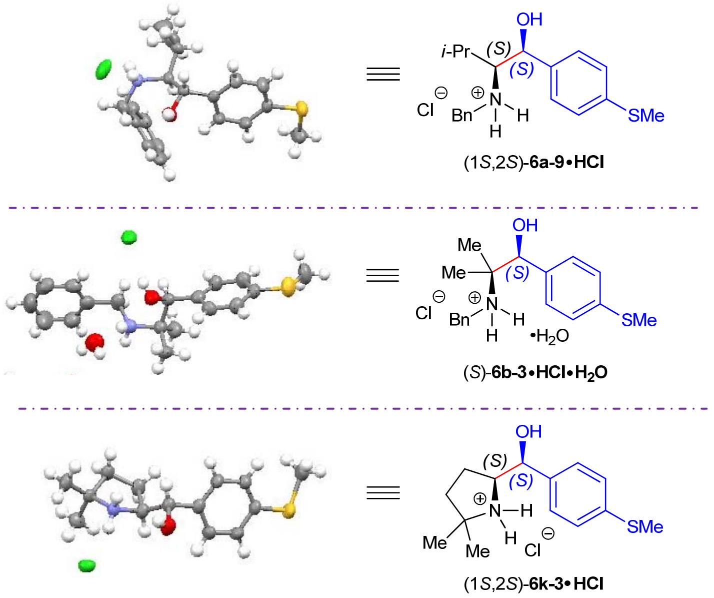


***Supplementary Figure 2.*** **Single X-ray structures of 6a-9•HCl, 6b-3•HCl and 6k-3•HCl.** The absolute configurations of (1*S*,2*S*)-**6a-9•HCl** (CCDC: 1537335), (*S*)-**6b-3•HCl•$H_2O$** (CCDC: 1537337) and (1*S*,2*S*)-**6k-3•HCl** (CCDC: 1537338) were determined by single-crystal X-ray diffraction analysis.

**General procedure for vicinal amino alcohol hydrochlorides**[11]: To a solution of vicinal hydroxyamino alcohol **2** in EtOH/$NH_4Cl$ (sat.) = 2/1 (v/v) (*c* 0.05 M) was added indium powder (1.5 equiv.). The mixture was heated to reflux for 4 hours. Upon completion, the reaction mixture was cooled to room temperature, filtered through a thin pad of Celite and washed with EtOAc. Saturated $Na_2CO_3$ (aq.) was added to the filtrate, the organic layer was separated and the aqueous layer was extracted with EtOAc for three times. The combined organic layers were washed with brine, dried over anhydrous $Na_2SO_4$, filtered and concentrated under reduced pressure. The residue was purified by flash chromatography to afford the vicinal amino alcohol **6**. The amino alcohol **6** was dissolved in HCl solution (2.0 M in EtOAc). The solvent was evaporated under reduced pressure to form the hydrochloride **6•HCl**. Recrystallization of **6•HCl** from ethanol/cyclohexane afforded X-ray quality crystals.

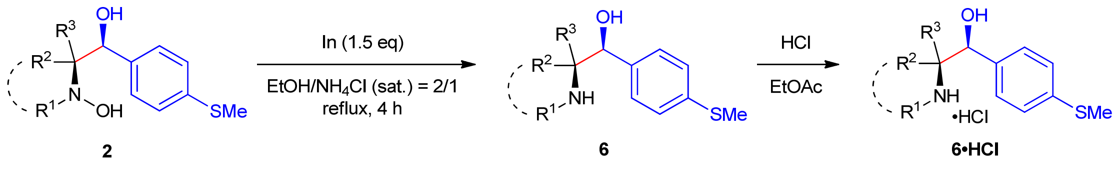


### (1*S*,2*S*)-2-(Benzylamino)-3-methyl-1-(4-(methylthio)phenyl)butan-1-ol (6a-9)

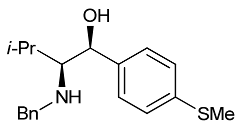

Following the **general procedure**, the reduction of **2a-9** (200 mg, 0.60 mmol, 90% ee) gave the desired product **6a-9** as a colorless oil (chromatography on silica gel, eluent: MeOH/$CH_2Cl_2$ = 1/30; 152 mg, 80% yield): $[\alpha]_D^{20}$ = +41.9 (*c* 1.0, $CHCl_3$, 90% ee); IR (film) $\nu_{max}$: 3375, 2959, 2911, 1499, 1450, 1092 $cm^{-1}$; $^1H$ NMR (400 MHz, $CDCl_3$) δ 0.89 (d, *J* = 6.9 Hz, 3H), 0.97 (d, *J* = 6.9 Hz, 3H), 1.69−1.84 (m, 1H), 2.43 (s, 3H), 2.57 (dd, *J* = 5.3, 4.9 Hz, 1H), 3.56 (d, *J* = 12.8 Hz, 1H), 3.68 (d, *J* = 12.8 Hz, 1H), 4.39 (d, *J* = 5.3 Hz, 1H), 7.11−7.37 (m, 9H); $^{13}C$ NMR (100 MHz, $CDCl_3$)

δ 15.8, 17.7, 20.4, 30.0, 54.4, 69.0, 71.8, 126.5, 126.6, 127.0, 128.0, 128.3, 136.6, 139.9, 141.4; HRMS calcd for $[C_{19}H_{26}NOS]^+$ $(M + H)^+$: 316.1730; found: 316.1729.

**(1*S*,2*S*)-2-(Benzylamino)-3-methyl-1-(4-(methylthio)phenyl)butan-1-ol hydrochloride (6a-9•HCl)**

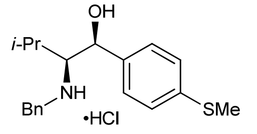


The product **6a-9•HCl** as a white solid: mp 169.0−169.7 ºC; $[\alpha]_D^{20}$ = +14.0 (*c* 1.0, MeOH, 90% ee); IR (film) $\nu_{max}$: 3369, 2959, 2921, 1454, 1335, 1089 $cm^{-1}$; $^1H$ NMR (400 MHz, MeOD) δ 0.89 (d, *J* = 7.0 Hz, 3H), 0.93 (d, *J* = 7.0 Hz, 3H), 1.75−1.90 (m, 1H), 2.49 (s, 3H), 3.20 (dd, *J* = 7.6, 3.0 Hz, 1H), 4.31 (d, *J* = 13.0 Hz, 1H), 4.43 (d, *J* = 13.0 Hz, 1H), 4.93 (d, *J* = 7.6 Hz, 1H), 7.26−7.37 (m, 4H), 7.40−7.50 (m, 5H); $^{13}C$ NMR (100 MHz, MeOD) δ 15.5, 17.6, 18.8, 29.0, 53.3, 68.6, 72.0, 127.7, 128.3, 130.2, 130.8, 131.7, 132.3, 139.5, 140.8; HRMS calcd for $[C_{19}H_{26}NOS]^+$ $(M)^+$: 316.1730; found: 316.1729.

**(*S*)-2-(Benzylamino)-2-methyl-1-(4-(methylthio)phenyl)propan-1-ol (6b-3)**

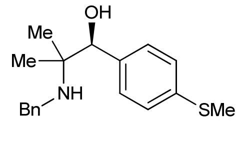


Following the **general procedure**, the reduction of **2b-3** (210 mg, 0.66 mmol, 94% ee) gave the desired product **6b-3** as a colorless oil (chromatography on silica gel, eluent: EtOAc/PE = 1/1; 175 mg, 88% yield): $[\alpha]_D^{20}$ = +10.6 (*c* 0.5, $CHCl_3$, 94% ee); IR (film) $\nu_{max}$: 3356, 2966, 2917, 2853, 1777, 1668, 1457, 1265, 1098 $cm^{-1}$; $^1H$ NMR (400 MHz, $CDCl_3$) δ 0.90 (s, 3H), 1.12 (s, 3H), 2.42 (s, 3H), 3.73 (s, 2H), 4.43 (s, 1H), 7.14−7.19 (m, 2H), 7.20−7.35 (m, 7H); $^{13}C$ NMR (100 MHz, $CDCl_3$) δ 15.6, 21.4, 23.1, 46.1, 57.1, 77.2, 125.6, 126.9, 128.00, 128.04, 128.3, 136.9, 137.3, 140.3; HRMS calcd for $[C_{18}H_{24}NOS]^+$ $(M + H)^+$: 302.1573; found: 302.1576.

**(*S*)-2-(Benzylamino)-2-methyl-1-(4-(methylthio)phenyl)propan-1-ol hydrochloride (6b-3•HCl)**

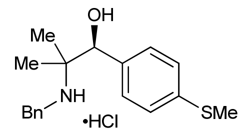


The product **6b-3•HCl** as a white solid: mp 137.6−138.5 ºC; $[\alpha]_D^{20}$ = +31.4 (*c* 1.0, MeOH, 94% ee); IR (film) $\nu_{max}$: 3369, 2917, 1438, 1338, 1092 $cm^{-1}$; $^1H$ NMR (400 MHz, MeOD) δ 1.27 (s, 3H), 1.37 (s, 3H), 2.49 (s, 3H), 4.31 (d, *J* = 12.6 Hz, 1H), 4.39 (d, *J* = 12.6 Hz, 1H), 5.06 (s, 1H), 7.26−7.32 (m, 2H), 7.39−7.52 (m, 5H), 7.58−7.67 (m, 2H); $^{13}C$ NMR (100 MHz, MeOD) δ 15.6, 19.4, 21.1, 46.4, 64.6, 74.5, 126.9, 129.5, 130.1, 130.4, 131.4, 133.2, 137.1, 140.4; HRMS calcd for $[C_{18}H_{24}NOS]^+$ $(M)^+$: 302.1573; found: 302.1576.

**(*S*)-((*S*)-5,5-Dimethylpyrrolidin-2-yl)(4-(methylthio)phenyl)methanol (6k-3)**

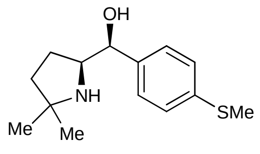


Following the **general procedure**, the reduction of **2k-3** (181 mg, 0.68 mmol, 95% ee) gave the desired product **6k-3** as a colorless oil (chromatography on silica gel, eluent: MeOH/$CH_2Cl_2$ = 1/6; 89.0 mg, 52% yield): $[\alpha]_D^{20}$ = +22.6 (*c* 0.5, $CHCl_3$, 95% ee); IR (film) $\nu_{max}$: 3324, 2956, 2917, 2853, 1265, 1092, 1008 $cm^{-1}$; $^1H$ NMR (500 MHz, MeOD) δ 1.16 (s, 3H), 1.23 (s, 3H), 1.55−1.65 (m, 4H), 2.46 (s, 3H), 3.38−3.45 (m, 1H), 4.41 (d, *J* = 7.4 Hz, 1H), 7.22−7.26 (m, 2H), 7.27−7.32 (m, 2H); $^{13}C$ NMR (125 MHz, MeOD) δ 15.9, 28.6, 29.4, 29.8, 40.0, 60.5, 65.4, 78.0, 127.6, 128.3, 139.2, 141.4; HRMS calcd for $[C_{14}H_{22}NOS]^+$ $(M + H)^+$: 252.1417; found: 252.1418.

**(*S*)-((*S*)-5,5-Dimethylpyrrolidin-2-yl)(4-(methylthio)phenyl)methanol hydrochloride (6k-3•HCl)**

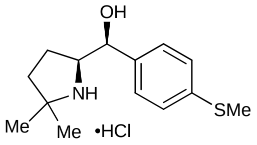


The product **6k-3•HCl** as a white solid: mp > 230 ºC (decomposed); $[\alpha]_D^{20}$ = +46.4 (*c* 1.0, MeOH, 95% ee); IR (film) $\nu_{max}$: 3369, 2972, 2914, 1438, 1380, 1082 $cm^{-1}$; $^{1}H$ NMR (400 MHz, MeOD) δ 1.44 (s, 3H), 1.53 (s, 3H), 1.83−2.00 (m, 4H), 2.47 (s, 3H), 3.85−3.96 (m, 1H), 4.75 (d, *J* = 8.5 Hz, 1H), 7.25−7.31 (m, 2H), 7.38−7.44 (m, 2H); $^{13}C$ NMR (100 MHz, MeOD) δ 15.6, 25.5, 25.9, 27.0, 38.4, 66.1, 66.2, 74.6, 127.6, 128.4, 139.0, 140.5; HRMS calcd for $[C_{14}H_{22}NOS]^+$ $(M)^+$: 252.1417; found: 252.1418.

## Control Experiments for the Photocatalytic Cross-Coupling of Nitrones with Aldehydes:

A series of experimental studies were carried out to gain a better comprehension of this reductive coupling reaction. Control experiments established that photocatalyst, Lewis acid, co-reductant (DIPEA) and light are both essential for this reaction (Supplementary Table 4). Additionally, when 1.0 equivalent of TEMPO (2,2,6,6-tetramethylpiperidine-1-oxyl) was added to the reaction system, the reductive coupling of nitrones with aldehydes did not occur. All of these results implied a free radical process in this reductive coupling reaction.

***Supplementary Table 4.*** Control experiments for the mechanism study

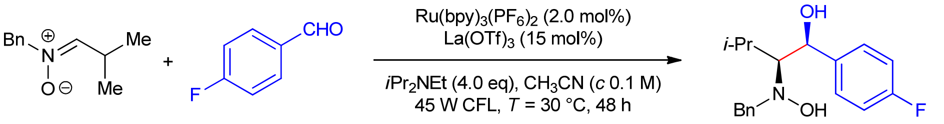


**1a** (1.0 eq) (1.5 eq) (±)-**2a-1**
92% yield, 1/3.4 dr (*anti*/*syn*)

| Entry | Variation from standard condition | Yield (%)[a] |
|---|---|---|
| 1 | None | 92 |
| 2 | No $Ru(bpy)_3(PF_6)_2$ | 0 |
| 3 | No $La(OTf)_3$ | 0 |
| 4 | No DIPEA | 0 |
| 5 | No light | 0 |
| 6 | Added TEMPO (1 equiv.) | 0 |

[a] Yields were determined by $^{1}H$ NMR analysis using an internal standard.

To compare our photocatalytic reductive coupling reaction with previous $SmI_2$-mediated reductive coupling of nitrones with aldehydes/ketones[12], some experiments were conducted to examine the role of nitrones under the photocatalytic conditions. Surprisingly, nitrone **1a** reacted with neither aliphatic aldehydes (*e.g.*, isobutyraldehyde, Supplementary Fig. 3-a) nor α,β-unsaturated compounds (*e.g.*, methyl acrylate, Supplementary Fig. 3-b) under the standard condition (as demonstrated in Supplementary Table 4). Moreover, homocoupling product **S6** was not observed when nitrone **1o** was treated under the standard condition in the absence of aldehyde, which could be obtained under $SmI_2$-mediated condition[12] (Supplementary Fig. 3-c). Combined these results with radical clock reactions (see Fig. 2), we speculated this photocatalytic reductive coupling is initialed by visible-light-exited single electron transfer (SET) reduction of aldehydes rather than that of nitrones.

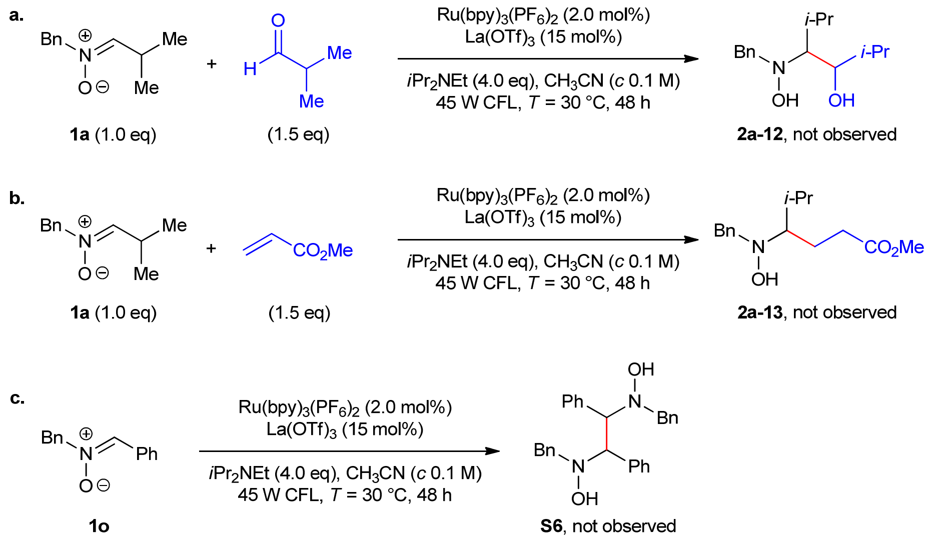


***Supplementary Figure 3.*** **Examinations of the role of nitrones under the photocatalytic conditions. a,** cross-coupling product **2a-12** was not observed when nitrone **1a** and isobutyraldehyde were treated under the standard condition. **b**, cross-coupling product **2a-13** was not observed when nitrone **1a** and methyl acrylate were treated under the standard condition. **c**, homocoupling product **S6** was not observed when nitrone **1o** was treated under the standard condition in the absence of aldehyde.

## Radical Clock Reactions:

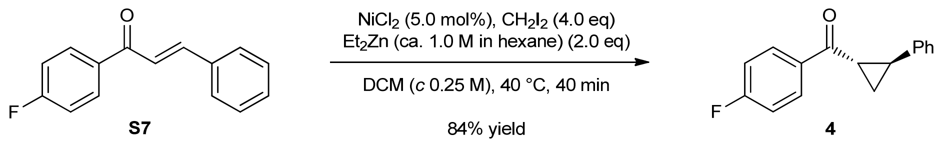


Based on a literature procedure[13], an oven-dried 100 mL round bottom flask equipped with a magnetic stir bar was added **S7** (1.13 g, 5.0 mmol, 1.0 equiv.) and $NiCl_2$ (32.4 mg, 0.25 mmol, 5.0 mol%) in the glove box. When the flask was sealed and removed from the glove box, DCM (20.0 mL) was added, followed by $CH_2I_2$ (1.61 mL, 20 mmol, 4.0 equiv.). The mixture was heated to 40 ºC, and $Et_2Zn$ (ca. 1.0 M in hexane) (10.0 mL, 10 mmol, 2.0 equiv.) was added slowly (over 30 min). After being stirred at 40 ºC for additional 10 min, the reaction mixture was diluted with 20 mL EtOAc, and quenched with saturated $NH_4Cl$ (aq.) (10 mL). The organic layer was separated, and the aqueous layer was extracted with $CH_2Cl_2$ (3×10 mL). The combined organic layers were dried over anhydrous $Na_2SO_4$, filtered and concentrated under reduced pressure. The residue was purified by flash chromatography on silica gel to afford **4** as a white solid (eluent: EtOAc/PE = 1/30; 1.01 g, 84% yield): mp 60.1−60.7 ºC; IR (film) $\nu_{max}$: 3062, 3026, 1668, 1595, 1418, 1220, 1159, 1034 $cm^{-1}$; $^1$H NMR (400 MHz, $CDCl_3$) δ 1.54 (ddd, $J$ = 7.6, 6.8, 4.2 Hz, 1H), 1.84−1.97 (m, 1H), 2.62−2.76 (m, 1H), 2.77−2.90 (m, 1H), 7.04−7.26 (m, 5H), 7.26−7.38 (m, 2H), 7.95−8.09 (m, 2H); $^{13}$C NMR (100 MHz, $CDCl_3$) δ 19.1, 29.1, 29.9, 115.6 (d, $J$ = 21.8 Hz), 126.1, 126.6, 128.5, 130.6 (d, $J$ = 9.3 Hz), 134.1 (d, $J$ = 2.9 Hz), 140.3, 165.6 (d, $J$ = 254.5 Hz), 196.8; HRMS calcd for $[C_{16}H_{13}FNaO]^+$ $(M + Na)^+$: 263.0843; found: 263.0843.

**General procedure for the radical clock reaction**: An oven-dried 25 mL Schlenk tube equipped with a

magnetic stir bar was added nitrone **1f** (74.6 mg, 0.50 mmol, 1.0 equiv.), ketone **3** or **4** (0.50 mmol, 1.0 equiv.), $Ru(bpy)_3(PF_6)_2$ (8.6 mg, 0.01 mmol, 2.0 mol%) and $La(OTf)_3$ (44.0 mg, 0.075 mmol, 15 mol%) in the glove box. When the tube was sealed and removed from the glove box, $CH_3CN$ (5.0 mL, *c* 0.1 M) was added, followed by DIPEA (0.35 mL, 2.0 mmol, 4.0 equiv.). The tube was placed approximately 10 cm away from a 45 W CFL. After being stirred at 30 ºC under an argon atmosphere for 48 hours, the reaction mixture was filtered through a thin pad of silica gel (100 to 200 mesh), washed with EtOAc and concentrated under reduced pressure. The residue was purified by flash chromatography to afford the product.

**2-(Benzyl(hydroxy)amino)-1-(4-fluorophenyl)propan-1-ol (2f-2)**

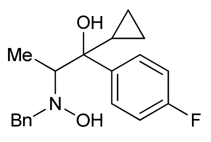


Following the **general procedure**, the reaction of nitrone **1f** and ketone **3** afforded **2f-2** as a 1/1.6 (dr, minor/major) mixture of inseparable diastereomers (chromatography on silica gel, eluent: EtOAc/PE = 1/8; 42.7 mg, 27% combined yield; recovered 59.0 mg ketone **3**, 72%): IR (film) $\nu_{max}$: 3427, 3004, 2942, 1604, 1502, 1223 $cm^{-1}$; HRMS calcd for $[C_{19}H_{22}FNNaO_2]^+$ $(M + Na)^+$: 338.1527; found: 338.1525.

Major diastereomer (data read from spectrum of the diastereomeric mixture): $^1$H NMR (400 MHz, $CDCl_3$) δ 0.14−0.32 (m, 2H), 0.36−0.58 (m, 2H), 1.11 (d, *J* = 6.8 Hz, 3H), 1.42−1.53 (m, 1H), 3.16 (q, *J* = 6.8 Hz, 1H), 3.76 (d, *J* = 13.3 Hz, 1H), 3.86−4.02 (br, 1H), 3.94 (d, *J* = 13.3 Hz, 1H), 4.28−4.48 (br, 1H), 6.91−7.03 (m, 2H), 7.19−7.45 (m, 7H); $^{13}$C NMR (100 MHz, $CDCl_3$) δ 0.6, 1.9, 6.4, 20.3, 62.0, 68.8, 76.2, 114.4 (d, *J* = 20.9 Hz), 127.2 (d, *J* = 7.7 Hz), 127.5, 128.4, 129.0, 137.7, 141.6 (d, *J* = 3.1 Hz), 161.4 (d, *J* = 244.4 Hz).
Minor diastereomer (data read from spectrum of the diastereomeric mixture): $^1$H NMR (400 MHz, $CDCl_3$) δ 0.14−0.32 (m, 1H), 0.36−0.58 (m, 2H), 0.60−0.71 (m, 1H), 1.18 (d, *J* = 6.8 Hz, 3H), 1.30−1.37 (m, 1H), 3.03 (q, *J* = 6.8 Hz, 1H), 3.72 (d, *J* = 13.3 Hz, 1H), 3.86−4.02 (br, 1H), 4.02 (d, *J* = 13.3 Hz, 1H), 4.53−4.68 (br, 1H), 6.91−7.03 (m, 2H), 7.19−7.45 (m, 7H); $^{13}$C NMR (100 MHz, $CDCl_3$) δ 1.1, 2.5, 5.8, 16.4, 62.0, 69.1, 75.4, 114.3 (d, *J* = 21.0 Hz), 127.5, 128.0 (d, *J* = 7.9 Hz), 128.4, 129.1, 137.4, 142.4 (d, *J* = 2.9 Hz), 161.5 (d, *J* = 244.7 Hz).

**5-(Benzyl(hydroxy)amino)-1-(4-fluorophenyl)-4-phenylhexan-1-one (5)**

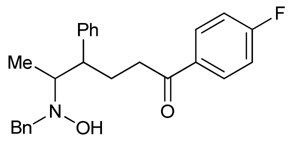


Following the **general procedure**, the reaction of nitrone **1f** and ketone **4** afforded **5** as a 1/2.0 (dr, minor/major) mixture of diastereomers (chromatography on silica gel, eluent: EtOAc/PE = 1/8; 104 mg, 53% combined yield; recovered 55.3 mg ketone **4**, 46%).

Major diastereomer (more polar) as a white solid: mp 85.0−85.7 ºC; IR (film) $\nu_{max}$: 3462, 3030, 2917, 1688, 1598, 1223, 1150 $cm^{-1}$; $^1$H NMR (400 MHz, $CDCl_3$) δ 1.18 (d, *J* = 5.9 Hz, 3H), 1.86−2.00 (m, 1H), 2.34−2.47 (m, 1H), 2.68−2.81 (m, 2H), 2.95−3.08 (m, 2H), 3.64 (d, *J* = 13.4 Hz, 1H), 3.95 (d, *J* = 13.4 Hz, 1H), 4.78−5.05 (br, 1H), 6.97−7.06 (m, 2H), 7.08−7.26 (m, 8H), 7.26−7.36 (m, 2H), 7.73−7.85 (m, 2H); $^{13}$C NMR (100 MHz, $CDCl_3$) δ 10.5, 24.5, 36.4, 48.5, 60.0, 65.8, 115.4 (d, *J* = 21.8 Hz), 126.2, 126.8, 128.0, 128.3, 128.4, 128.9, 130.5 (d, *J* = 9.4 Hz), 133.3 (d, *J* = 2.8 Hz), 138.6, 142.9, 165.5 (d, *J* = 254.3 Hz), 198.8; HRMS calcd for $[C_{25}H_{26}FNNaO_2]^+$ $(M + Na)^+$: 414.1840; found: 414.1843.

Minor diastereomer (less polar) as a white solid: mp 123.1−124.0 ºC; IR (film) $\nu_{max}$: 3346, 3026, 2975, 2930, 1681, 1595, 1233, 1156 $cm^{-1}$; $^1$H NMR (400 MHz, $CDCl_3$) δ 0.86 (d, *J* = 6.3 Hz, 3H), 1.66−1.80 (m, 1H), 2.66−2.81 (m, 3H), 2.93−3.13 (m, 2H), 3.78 (d, *J* = 13.6 Hz, 1H), 4.08 (d, *J* = 13.6 Hz, 1H), 5.44−5.57 (br, 1H), 6.99−7.08 (m,

2H), 7.08−7.14 (m, 2H), 7.17−7.37 (m, 6H), 7.40−7.49 (m, 2H), 7.77−7.89 (m, 2H); $^{13}$C NMR (100 MHz, $CDCl_3$) δ 9.5, 27.1, 35.9, 50.0, 60.3, 64.7, 115.5 (d, $J$ = 21.7 Hz), 126.5, 126.9, 128.2, 128.5, 128.6, 128.9, 130.6 (d, $J$ = 9.3 Hz), 133.4 (d, $J$ = 2.9 Hz), 139.0, 143.4, 165.6 (d, $J$ = 254.6 Hz), 199.4; HRMS calcd for $[C_{25}H_{26}FNNaO_2]^+$ (M + Na)$^+$: 414.1840; found: 414.1840.

## Cyclic Voltammetry Experiments:

Cyclic voltammetry was performed on a CHI660E electrochemical analyzer. All data were obtained at 10 ºC and 50 mV/s sweep rate using a three electrode setup: Pt wire counter electrode, glassy carbon working electrode and saturated calomel reference electrode (SCE). A 0.01 M $CH_3CN$ solution of nitrone **1a** or 4-fluorobenzaldehyde [with or without $Sc(OTf)_3$] was prepared with 0.1 M tetrabutylammonium hexafluorophosphate as the supporting electrolyte. The solutions were sparged with nitrogen for 10 minutes prior to acquiring all CV data.

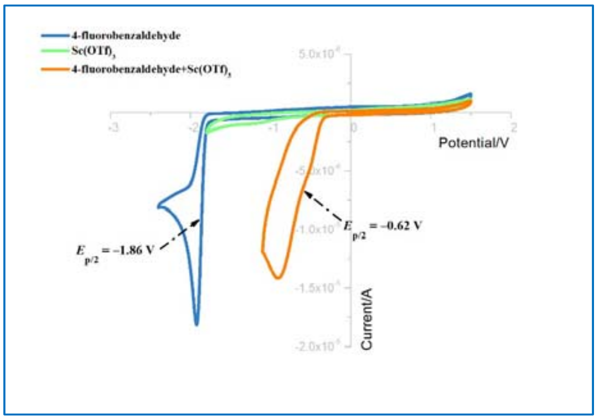


***Supplementary Figure 4.*** **CV of 4-fluorobenzaldehyde and $Sc(OTf)_3$.** Addition of $Sc(OTf)_3$ resulted in a significant reduction in 4-fluorobenzaldehyde’s half-wave potential which shifted from −1.86 V to −0.62 V (*vs.* SCE) in $CH_3CN$.

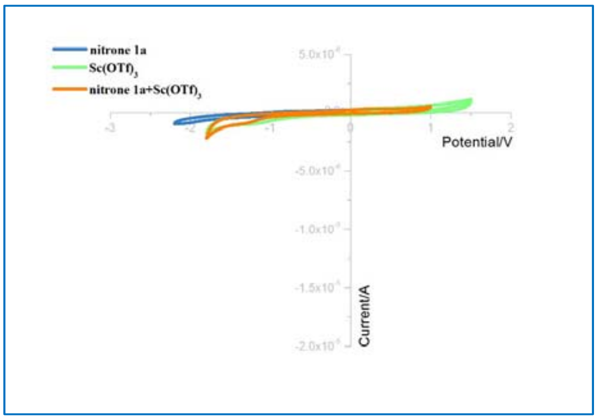


***Supplementary Figure 5.*** **CV of nitrone 1a and $Sc(OTf)_3$.** Addition of $Sc(OTf)_3$ has no significant influence on nitrone **1a**’s half-wave potential in $CH_3CN$.

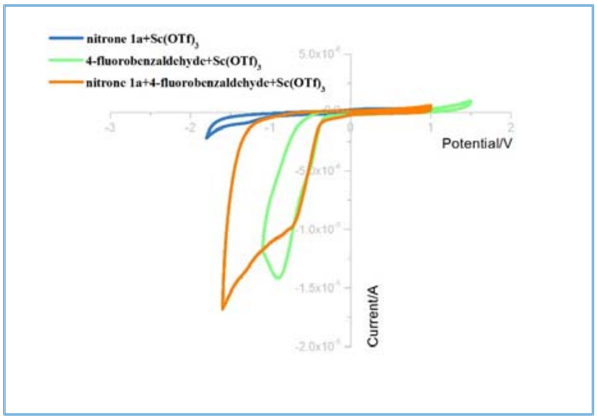


***Supplementary Figure 6.*** **CV of nitrone 1a, 4-fluorobenzaldehyde and Sc(OTf)$_3$.** A mixture of nitrone **1a**, 4-fluorobenzaldehyde and Sc(OTf)$_3$ with an onset potential $E_{op}$ > –0.5 V (*vs.* SCE) in $CH_3CN$.

## Solubility of Nitrones, Aldehydes and Lewis Acids in Acetonitrile or 1,2-Dichloroethane:

The solubility of nitrone **1a**, 4-fluorobenzaldehyde and Lewis acids with the same stoichiometry as the standard photocatalytic condition demonstrated in Supplementary Table 4 was investigated (Supplementary Fig. 7). Lewis acids, such as La(OTf)$_3$, showed poor solubility in $CH_3CN$ (Supplementary Fig. 7-A, entry I). Addition of 4-fluorobenzaldehyde has no significant influence on La(OTf)$_3$'s solubility (Supplementary Fig. 7-A, entry II). However, nitrones (such as **1a**) can significantly improve the solubility of La(OTf)$_3$ in $CH_3CN$ to give a homogeneous and transparent solution (Supplementary Fig. 7-A, entries III and IV). Similar solubility of nitrone **1a**, 4-fluorobenzaldehyde and Sc(OTf)$_3$ in DCE was observed (Supplementary Fig. 7-B). Taking full account of control experiments, CV experiments and reactants' solubility studies, a complex of nitrone, aldehyde and Lewis acid is a plausible starting point of the photocatalytic reaction.

**A:**

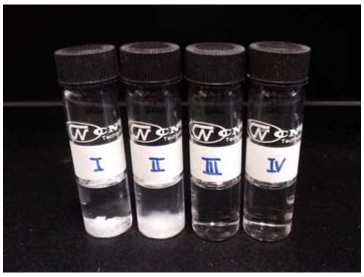


(**I**) La(OTf)$_3$ (26 mg, 0.045 mmol) in $CH_3CN$ (3.0 mL). (**II**) La(OTf)$_3$ (26 mg, 0.045 mmol) and 4-fluorobenzaldehyde (48 μL, 0.45 mmol) in $CH_3CN$ (3.0 mL). (**III**) La(OTf)$_3$ (26 mg, 0.045 mmol) and nitrone **1a** (53 mg, 0.30 mmol) in $CH_3CN$ (3.0 mL). (**IV**) La(OTf)$_3$ (26 mg, 0.045 mmol), nitrone **1a** (53 mg, 0.30 mmol) and 4-fluorobenzaldehyde (48 μL, 0.45 mmol) in $CH_3CN$ (3.0 mL).

**B:**

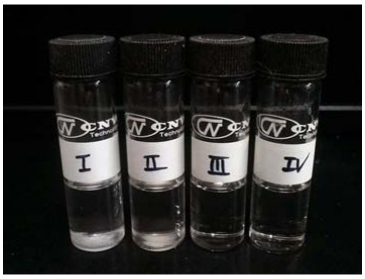


(**I**) Sc(OTf)$_3$ (22 mg, 0.045 mmol) in DCE (3.0 mL). (**II**) Sc(OTf)$_3$ (22 mg, 0.045 mmol) and 4-fluorobenzaldehyde (48 μL, 0.45 mmol) in DCE (3.0 mL). (**III**) Sc(OTf)$_3$ (22 mg, 0.045 mmol) and nitrone **1a** (53 mg, 0.30 mmol) in DCE (3.0 mL). (**IV**) Sc(OTf)$_3$ (22 mg, 0.045 mmol), nitrone **1a** (53 mg, 0.30 mmol) and 4-fluorobenzaldehyde (48 μL, 0.45 mmol) in DCE (3.0 mL).

***Supplementary Figure 7.*** **The solubility of nitrones, aldehydes and Lewis acids in acetonitrile or 1,2-dichloroethane.** Nitrones can improve the solubility of Lewis acids while aldehydes cannot. Thus, Lewis acids have a significantly higher affinity with nitrones than that with aldehydes.

## Concise Synthesis of (+)-Ephedrine and (−)-Selegiline:

**(1*S*,2*R*)-2-(Hydroxy(methyl)amino)-1-phenylpropan-1-ol (2n)**

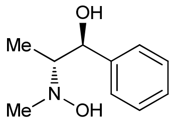


Following the **modified method 1**, the asymmetric reductive coupling of nitrone **1n** (110 mg, 1.5 mmol, 5.0 equiv.) with benzaldehyde (31 μL, 0.30 mmol, 1.0 equiv.) gave the desired product **2n** as the major diastereomer [dr = 11/1 (*anti*/*syn*), determined by chiral HPLC analysis] with 94% ee [Lux® Amylose-1 column, Shimadzu HPLC, 30 ºC, hexane/EtOH = 80/20 (v/v), 1.0 mL/min, 225 nm; $t_1$ = 7.2 min, $t_2$ = 8.1 min]. Characterization data was obtained by chromatography on silica gel (eluent: EtOAc; 46.2 mg, 85% combined yield).

Major diastereomer (*anti*, more polar) as a white solid: mp 69.8−70.4 ºC; $[\alpha]_D^{20}$ = +12.6 (*c* 1.0, MeOH, 94% ee); IR (film) $\nu_{max}$: 3369, 2988, 2850, 1460 $cm^{-1}$; $^1H$ NMR (400 MHz, MeOD) δ 0.91 (d, *J* = 6.7 Hz, 3H), 2.68 (s, 3H), 2.73−2.82 (m, 1H), 5.24 (s, 1H), 7.17−7.25 (m, 1H), 7.27−7.42 (m, 4H); $^{13}C$ NMR (100 MHz, MeOD) δ 9.0, 45.8, 70.5, 73.8, 127.0, 127.8, 129.0, 144.2; HRMS calcd for $[C_{10}H_{15}NNaO_2]^+$ $(M + Na)^+$: 204.0995; found: 204.0995.

**(1*S*,2*R*)-Ephedrine (6n)**

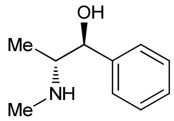


Following the **general procedure**, the reduction of **2n** (29.8 mg, 0.16 mmol, 94% ee) gave the desired product **6n** as a colorless oil [chromatography on silica gel, eluent: MeOH/$CH_2Cl_2$ = 1/5 with 1% aqueous $NH_3$ (sat.); 24.5 mg, 90% yield]: $[\alpha]_D^{20}$ = +30.6 (for **6n•HCl**, *c* 2.0, $H_2O$, 94% ee), [lit.[14] for (−)-ephedrine•HCl: $[\alpha]_D^{20}$ = −34.7 (*c* 5.0, $H_2O$)]; IR (film) $\nu_{max}$: 3315, 2927, 2853, 1777, 1668, 1451 $cm^{-1}$; $^1H$ NMR (400 MHz, $CDCl_3$) δ 0.87 (d, *J* = 6.5 Hz, 3H), 2.47 (s, 3H), 2.76−2.87 (m, 1H), 3.20−3.46 (br, 2H), 4.82 (d, *J* = 3.5 Hz, 1H), 7.21−7.27 (m, 1H), 7.27−7.38 (m, 4H); $^{13}C$ NMR (100 MHz, $CDCl_3$) δ 13.5, 33.7, 60.5, 72.9, 126.0, 127.0, 128.0, 141.5; MS (ESI): *m/z* 166 $(M + H)^+$.

**(*R*)-Selegiline (8)**

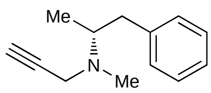


To a 10 mL round bottom flask equipped with a magnetic stir bar was added 10% Pd/C (27.2 mg, 30% wt) and a solution of **2n** (90.6 mg, 0.50 mmol, 1.0 equiv.) in 1 N aqueous HCl (5.0 mL, *c* 0.1 M). The reaction mixture was stirred under hydrogen (1 atmosphere) at 60 ºC for 48 hours. Upon completion, the reaction mixture was cooled to room temperature, filtered through a thin pad of Celite and washed with methanol. The filtrate was concentrated under reduce pressure and then was dried under vacuum to afford crude (*R*)-methamphetamine hydrochloride in quantitative yield, which can be used in next step without further purification.

To a mixture of crude (*R*)-methamphetamine hydrochloride (92.5 mg, ca. 0.50 mmol, 1.0 equiv.) and $K_2CO_3$ (207 mg, 1.5 mmol, 3.0 equiv.) was added $CH_3CN$ (10.0 mL, *c* 0.05 M) and propargyl bromide (59 μL, ca. 80% wt in toluene, 0.55 mmol, 1.1 equiv.) under an argon atmosphere. The reaction mixture was stirred at 80 ºC for 16 hours. Upon completion, the reaction mixture was cooled to room temperature, filtered, washed with $CH_2Cl_2$ and concentrated under reduce pressure. The residue was purified by flash chromatography on silica gel to afford the desired product **8** as a yellow oil (eluent: EtOAc/PE = 1/4; 84.4 mg, 90% yield for two steps from **2n**): $[\alpha]_D^{25}$ = −1.02 (*c* 1.0, EtOH, 94% ee), [lit.[15] for **8**: $[\alpha]_D^{20}$ = −1.29 (*c* 6.43, EtOH, > 99% ee)]; IR (film) $\nu_{max}$: 3295, 3026, 2959, 2930, 2786, 1454 $cm^{-1}$; $^1H$ NMR (400 MHz, $CDCl_3$) δ 0.96 (d, *J* = 6.5 Hz, 3H), 2.23 (t, *J* = 2.3 Hz, 1H), 2.38

(dd, $J$ = 12.8, 9.5 Hz, 1H), 2.41 (s, 3H), 2.92−3.02 (m, 1H), 3.03 (dd, $J$ = 12.8, 4.0 Hz, 1H), 3.41 (d, $J$ = 2.3 Hz, 2H), 7.14−7.21 (m, 3H), 7.23−7.30 (m, 2H); $^{13}$C NMR (100 MHz, $CDCl_3$) δ 15.0, 37.3, 39.7, 43.0, 59.3, 72.4, 80.3, 125.8, 128.2, 129.2, 140.2; MS (ESI): $m/z$ 188 (M + H)$^+$.

## DFT Calculations for the Proposed Mechanism Cycle:

To determinate the proposed mechanism of our photocatalytic reductive cross-coupling reaction of nitrones with aldehydes, the detailed density functional theory (DFT) calculations were performed (Supplementary Fig. 8).

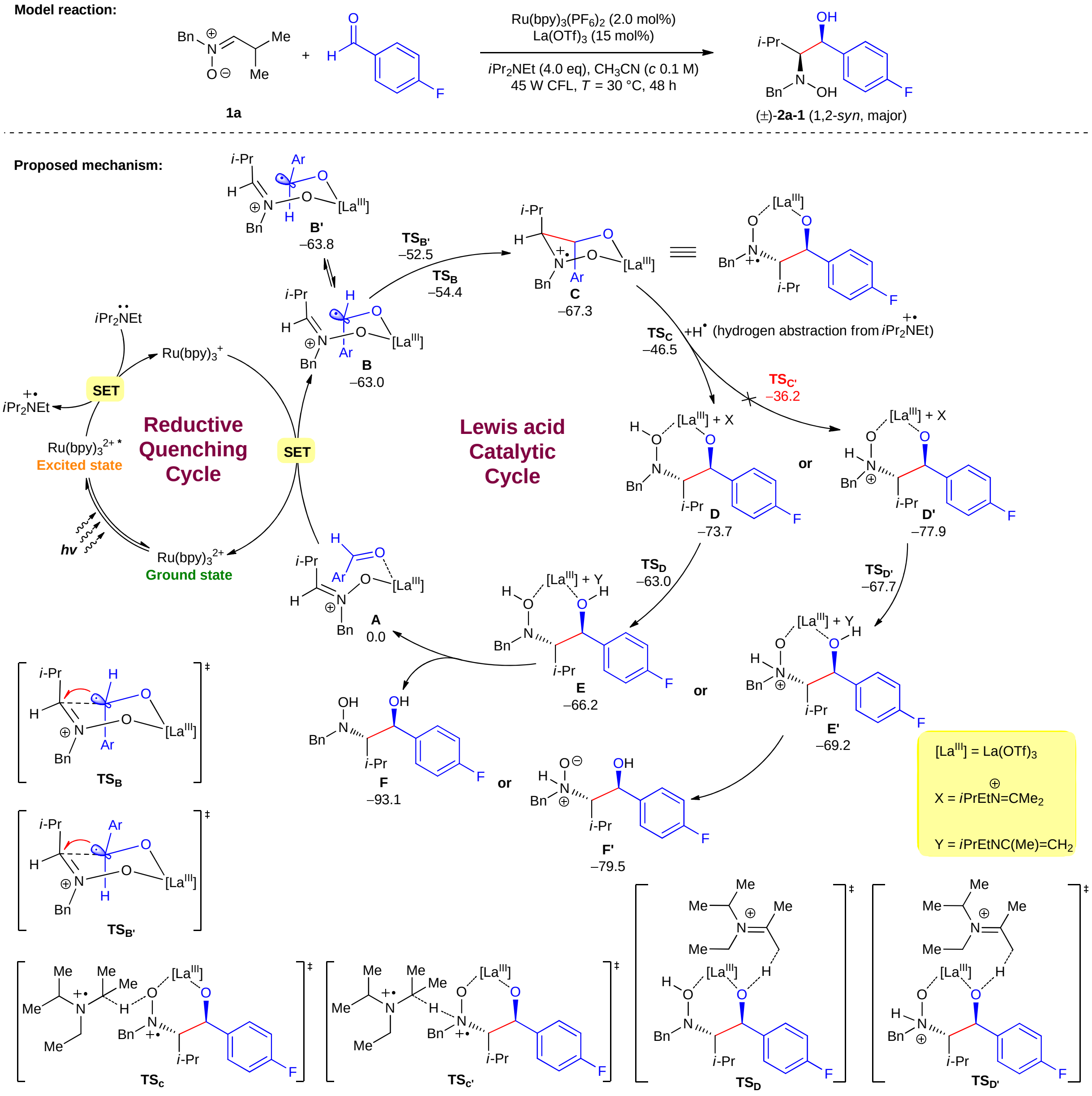


***Supplementary Figure 8*. DFT calculations of La(OTf)$_3$-promoted photocatalytic reductive cross-coupling reaction of nitrones with aldehydes (ΔG in kcal/mol at 298 K).** The detailed DFT calculations were performed. Note that $i$Pr$_2$NEt (DIPEA) was used as sacrificed reductant and hydrogen-source. Thus, [$i$Pr$_2$(Et)N·]$^+$ (formed by donating an electron from DIPEA to the photo-excited state of [Ru(bpy)$_3$]$^{2+}$) and [$i$Pr(Et)N=C(Me)$_2$]$^+$ were used as hydrogen- and proton-sources in the hydrogen-abstraction and proton-transfer steps, respectively.

**Computational methods**: All calculations were carried out with the Gaussian 09 programs[16]. The geometries of all the species were fully optimized by using DFT of the B3LYP method[17,18]. This method was proven to be reliable for description of geometrical parameters and electronic structures as well as for studying the substrates and reproducing the ground state geometry of La(III) based systems[19–22]. It has been successfully applied to the mechanistic studies of various catalytic reactions. The 6-31G (d)[23] basis set was used for all atoms except for lanthanum. For all systems the core electrons of lanthanum are described with quasi-relativistic effective core potential (ECP) which were optimized by the Stuttgard-Dresden group[24–26]. The large core ECP (MWB46) that considers the 4f electrons in the core and the 5s, 5p, 5d and 6s electrons in the valence shell (11 valence electrons) is applied to La(III). As it is stated in the literature, the 4f electrons do not take part in the metal-ligand bond because of their contraction in the core[22]. The large core ECP is used in combination with the optimized basis set. All stationary points were verified as minima or first-order saddle points by vibrational frequency analysis. IRC[27] calculations were used to confirm the transition state (TS). The intermediates were characterized by all real frequencies. All discussed energies are Gibbs free energies at 298 K and the solvent effects of acetonitrile solvent ($\varepsilon = 37.5$) for lanthanum catalysis were studied by performing the self-consistent reaction field (SCRF) of the SMD method of Truhlar and Cramer[28] at the same computational level.

## Complexes Formed from Nitrones, Aldehydes, La(OTf)$_3$ and Their Anions:

The electron affinity of nitrone **1a**, 4-fluorobenzaldehyde (**FBA**) in acetonitrile solvent is predicted to be 23.4 and 45.1 kcal/mol (in free energy), respectively (see Supplementary Equations 1 and 2).

**1a** + e → [**1a**]$^-$ ΔG(298K) = –23.4, ΔE = –23.0 (1)

**FBA** + e → [**FBA**]$^-$ ΔG(298K) = –45.1, ΔE = –44.7 (2)

Ligation of nitrone **1a** to the Lewis acid La(OTf)$_3$ is slightly stronger than that of **FBA**. Thus, both complexes La(OTf)$_3$(**1a**)$_2$ and **A** [**A** = La(OTf)$_3$(**1a**)(**FBA**)] can be formed within the solution (see Supplementary Equations 3 and 4).

La(OTf)$_3$ + 2 (**1a**) → La(OTf)$_3$(**1a**)$_2$ ΔG(298K) = 15.7, ΔE = –10.9 (3)

La(OTf)$_3$(**1a**)$_2$ + **FBA** → **A** + **1a** ΔG(298K) = 3.8, ΔE = 6.0 (4)

However, complex **A** is found to be much more electron-withdrawing than La(OTf)$_3$(**1a**)$_2$ and, upon SET from the photo-generated Ru(bpy)$_3^+$, the formation of cross-coupling precursor **B** (**B** = [La(OTf)$_3$(**1a**)(**FBA**)]$^-$) is overwhelmingly favored over the formation of homocoupling precursors [La(OTf)$_3$(**1a**)$_2$]$^-$ and [La(OTf)$_3$(**FBA**)$_2$]$^-$ (see Supplementary Equations 5 to 7).

La(OTf)$_3$(**1a**)$_2$ + e → [La(OTf)$_3$(**1a**)$_2$]$^-$ ΔG(298K) = –42.9, ΔE = –41.2 (5)

**A** + e → **B** ΔG(298K) = –63.0, ΔE = –63.6 (6)

**B** + **FBA** → [La(OTf)$_3$(**FBA**)$_2$]$^-$ + **1a** ΔG(298K) = 3.6, ΔE = 5.0 (7)

It is also clear that complexation to the Lewis acid significantly enhances the electron affinity of **FBA** (by ~18 kcal/mol) and, as a result, promotes the rate of SET from photo-generated Ru(bpy)$_3^+$ to complex **A**. The attached electron in the as-formed cross-coupling precursor **B** is delocalized within the **FBA** moiety (Supplementary Fig. 9). After the cross-coupling carbon-carbon bond formation (via transition state **TS$_B$**), the as-formed intermediate **C** has the spin-unpaired electron mostly localized within the *N-O* π*-orbital. This accounts for the preferential addition of

hydrogen (abstracted from the $[(iPr)_2EtN\cdot]^+$ radical cation via $\mathbf{TS_C}$) to the nitroxyl *O* atom in the following elementary step.

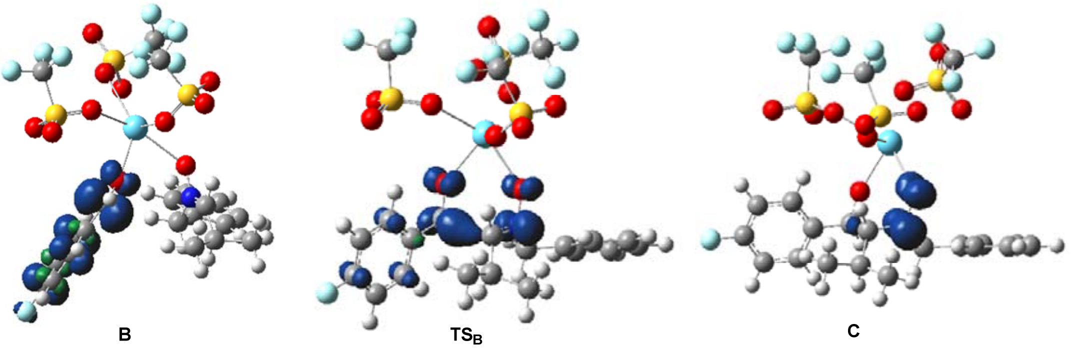


***Supplementary Figure 9.* The spin density distribution within intermediate B, transition State $\mathbf{TS_B}$ and intermediate C.** As shown in the figure, the attached electron in **B** is delocalized within the **FBA** moiety, which agrees well with the mechanism that this photocatalytic reaction is initiated by SET reduction of aldehydes. The as-formed intermediate **C** (via $\mathbf{TS_B}$) has the spin-unpaired electron mostly localized within the *N-O* π*-orbital, so the addition of hydrogen to the nitroxyl *O* atom in the following elementary step is preferential.

## Understanding the Stereoselectivity of Photocatalytic Cross-Coupling of Nitrone 1n with FBA:

For the photocatalytic reductive cross-coupling of nitrone **1n** with **FBA** catalyzed by $La(OTf)_3$, the *anti*-coupling pathway (via transition state $\mathbf{TS_{B1'}}$, Supplementary Fig. 10) has a smaller barrier height than the *syn*-coupling pathway (via $\mathbf{TS_{B1}}$), which accounts for the stereoselectivity towards *anti*-configuration product. Moreover, the enantioselectivity of cross-coupling product of nitrone **1n** with benzaldehyde is revealed by chiral scandium complex **II**[29] involving a *Re*-to-*Si*-facial attack of the ketyl radical to nitrone **1n**.

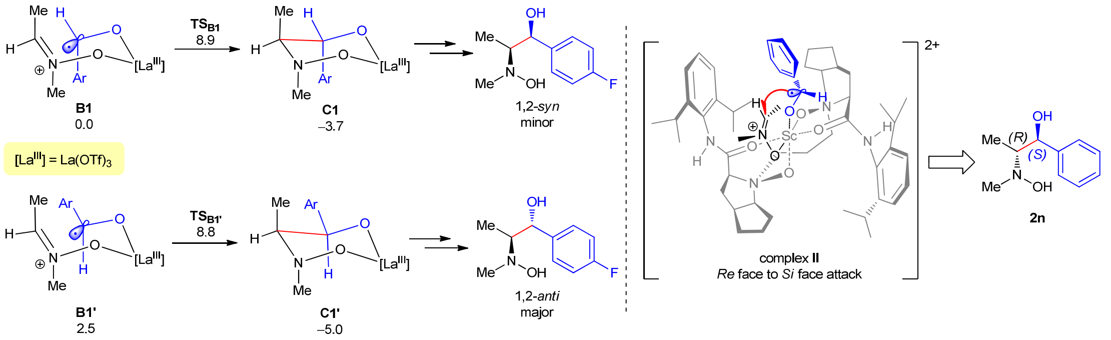


***Supplementary Figure 10.* DFT-computed relative free energies for precursors, transition states and products of the cross-coupling step between nitrone 1n and FBA ligated to $La(OTf)_3$ (ΔG in kcal/mol at 298 K).** DFT calculations revealed the *anti*-coupling pathway is more favored than *syn*-coupling pathway when nitrone **1n** cross-couples with **FBA**. Moreover, the enantioselectivity is revealed by chiral scandium complex **II** reasonably.

Compare with **2a-1**, **2e~2j** and **2n**, these result suggest *anti*- or *syn*-selectivity of this radical coupling reaction may be determined by interactions between substituent $R^1$ or $R^3$ of the aldimine oxide ($R^2$ = H) and Ar group of the

aldehyde. As shown in Supplementary Fig. 11, when $R^3$ is a bulky group, the interaction between $R^3$ and Ar will be greater than that between $R^1$ and Ar, *syn*-vicinal hydroxyamino alcohol will be obtained as a major diastereomer. While $R^3$ is a small group, the interaction between $R^1$ and Ar will be greater than that between $R^3$ and Ar of aldehyde, *anti*-vicinal hydroxyamino alcohol will be obtained as a major diastereomer.

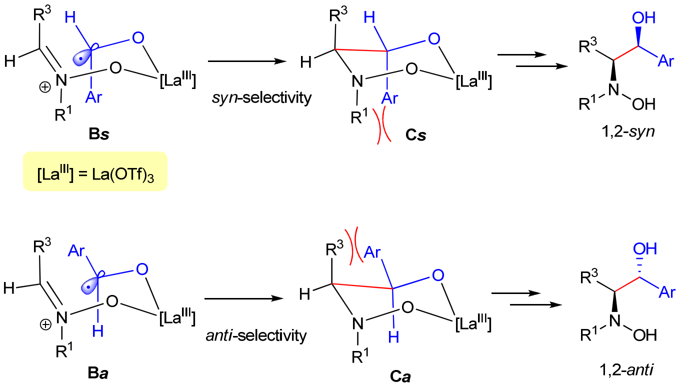


***Supplementary Figure 11.*** **Understanding the *syn*- or *anti*-stereoselectivity of photocatalytic reductive cross-coupling of nitrones with aromatic aldehydes.** A plausible understanding of diastereomeric selectivity by comparing the relative size of $R^3$ and $R^1$.

## Molecular Geometries and Energies:

B3LYP/6-31G*/ MWB46 cartesian coordinates and energies in Hartree

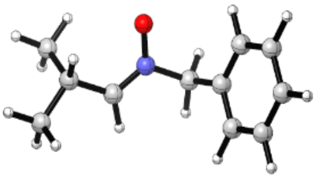

**1a**

Number of imaginary frequencies = 0

0 1

| | | | |
|---|---|---|---|
| C | 1.33339500 | 3.41403900 | -0.36650600 |
| C | 2.70969200 | 5.37688600 | 0.05712400 |
| H | 1.91241800 | 5.66913400 | 0.73069400 |
| N | 2.55279100 | 4.25467300 | -0.59231500 |
| O | 3.39873700 | 3.77800900 | -1.43762300 |
| C | 3.84854500 | 6.99837400 | -1.47526600 |
| H | 3.02007700 | 7.71723500 | -1.46129200 |
| H | 4.77739900 | 7.55466400 | -1.65130700 |
| H | 3.69723500 | 6.30791500 | -2.31112200 |
| C | 4.14616000 | 7.16244000 | 1.04870200 |
| H | 5.05807100 | 7.75470500 | 0.91050500 |
| H | 3.30631500 | 7.86058600 | 1.15781500 |
| H | 4.24466600 | 6.60503900 | 1.98794300 |

| | | | |
|---|---|---|---|
| H | 0.61831200 | 3.99554400 | 0.21776000 |
| H | 0.92415700 | 3.23715900 | -1.36425300 |
| C | 1.67873000 | 2.11723500 | 0.32682900 |
| C | 1.44506400 | 1.96873100 | 1.70060000 |
| C | 2.25008600 | 1.05347400 | -0.38647400 |
| C | 1.77291900 | 0.77714100 | 2.35247300 |
| H | 1.00051400 | 2.78860500 | 2.26005800 |
| C | 2.58134200 | -0.13657800 | 0.26437600 |
| H | 2.43626100 | 1.16476200 | -1.45006600 |
| C | 2.34342700 | -0.27764900 | 1.63516700 |
| H | 1.58127000 | 0.67319700 | 3.41729100 |
| H | 3.02199300 | -0.95523000 | -0.29884700 |
| H | 2.59677000 | -1.20630000 | 2.14004600 |
| C | 3.93189900 | 6.22129200 | -0.14426900 |
| H | 4.78400200 | 5.53225500 | -0.22378800 |

Energies (0K) = -558.122421

Energies (0K) + ZPE = -557.881501

Enthalpies (298K) = -557.867816

Free Energies (298K) = -557.922002

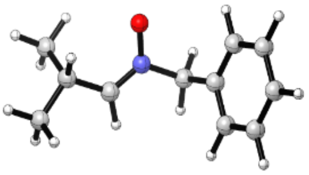

**[1a]**$^{-}$

Number of imaginary frequencies = 1

-1 2

| | | | |
|---|---|---|---|
| C | 1.18933700 | 3.39703600 | -0.58413700 |
| C | 2.68818000 | 5.19094900 | 0.21263500 |
| H | 1.80353700 | 5.76072300 | 0.54008600 |
| N | 2.33612600 | 4.29349300 | -0.81725700 |
| O | 3.25461100 | 3.79417800 | -1.64901200 |
| C | 3.80825200 | 6.91695400 | -1.32546500 |
| H | 2.98971200 | 7.64645400 | -1.23829700 |
| H | 4.73455700 | 7.47968300 | -1.52199900 |
| H | 3.60883500 | 6.27148600 | -2.18708100 |
| C | 4.23561200 | 6.91090000 | 1.17137600 |
| H | 5.14918600 | 7.50229300 | 1.02222500 |
| H | 3.42014200 | 7.61877100 | 1.38005900 |
| H | 4.37052400 | 6.29569500 | 2.07033600 |
| H | 0.40618200 | 3.96238700 | -0.06298500 |
| H | 0.81714500 | 3.10995200 | -1.57603300 |
| C | 1.57538900 | 2.17180500 | 0.20898100 |

| | | | |
|---|---|---|---|
| C | 1.44836700 | 2.14299600 | 1.60902600 |
| C | 2.20301500 | 1.08601200 | -0.42643000 |
| C | 1.91757800 | 1.05584700 | 2.35077400 |
| H | 0.96871100 | 2.97672400 | 2.11762800 |
| C | 2.67456800 | -0.00152500 | 0.31259400 |
| H | 2.32259000 | 1.10585600 | -1.50575700 |
| C | 2.53371400 | -0.02210800 | 1.70537600 |
| H | 1.79937400 | 1.04741400 | 3.43213200 |
| H | 3.15110600 | -0.83626900 | -0.19697000 |
| H | 2.89692300 | -0.87028700 | 2.28066200 |
| C | 3.91127300 | 6.04161300 | -0.05384400 |
| H | 4.75769900 | 5.35830500 | -0.22436800 |

Energies (0K) = -558.1540562

Energies (0K) + ZPE = -557.918175

Enthalpies (298K) = -557.904127

Free Energies (298K) = -557.959253

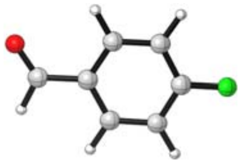

**4-fluorobenzaldehyde = FBA**

Number of imaginary frequencies = 0

0 1

| | | | |
|---|---|---|---|
| C | -4.28961000 | 8.78035000 | 0.34959800 |
| C | -2.88593100 | 8.76880200 | 0.34994300 |
| C | -2.16870000 | 9.96288800 | 0.34930800 |
| C | -2.88369100 | 11.15671700 | 0.34834600 |
| C | -4.27778100 | 11.20564900 | 0.34800100 |
| C | -4.97872800 | 10.00621900 | 0.34863500 |
| H | -2.35632700 | 7.81929600 | 0.35070000 |
| H | -1.08380400 | 9.98113100 | 0.34954900 |
| H | -4.78287100 | 12.16614700 | 0.34725300 |
| H | -6.06439800 | 10.00310500 | 0.34839200 |
| C | -5.02657100 | 7.50295200 | 0.35026100 |
| H | -4.38433800 | 6.59807600 | 0.35081300 |
| O | -6.24435400 | 7.40124800 | 0.34997000 |
| F | -2.19995400 | 12.32021300 | 0.34771800 |

Energies (0K) = -444.8102625

Energies (0K) + ZPE = -444.708281

Enthalpies (298K) = -444.700222

Free Energies (298K) = -444.740139

**[FBA]$^-$**

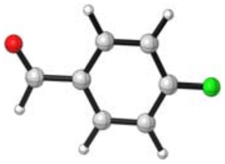

Number of imaginary frequencies = 1

-1 2

| | | | |
|---|---|---|---|
| C | -4.30496900 | 8.73288500 | 0.34957300 |
| C | -2.86845300 | 8.76988700 | 0.34991900 |
| C | -2.16634700 | 9.96392900 | 0.34934400 |
| C | -2.87580100 | 11.17572400 | 0.34839800 |
| C | -4.27465300 | 11.20156900 | 0.34798700 |
| C | -4.97935300 | 10.00662200 | 0.34855000 |
| H | -2.31896600 | 7.82890600 | 0.35067500 |
| H | -1.07893500 | 9.97999900 | 0.34960900 |
| H | -4.78964300 | 12.16006300 | 0.34721200 |
| H | -6.06592100 | 10.00978100 | 0.34824300 |
| C | -5.03347500 | 7.50729000 | 0.35024700 |
| H | -4.39325700 | 6.59169100 | 0.35093400 |
| O | -6.29903100 | 7.38353900 | 0.34999900 |
| F | -2.17825600 | 12.36090900 | 0.34779800 |

Energies (0K) = -444.8779447

Energies (0K) + ZPE = -444.779533

Enthalpies (298K) = -444.771255

Free Energies (298K) = -444.812089

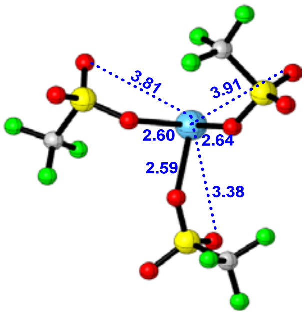


**La(OTf)$_3$**

Number of imaginary frequencies = 0

0 1

| | | | |
|---|---|---|---|
| O | 2.40212200 | 4.24699600 | 0.95787100 |
| S | 1.37545400 | 5.04051300 | 1.64659400 |
| O | 1.66535500 | 6.46761600 | 1.88530600 |
| O | -0.02088900 | 4.79432100 | 1.12319200 |
| C | 1.28418000 | 4.31332600 | 3.35628000 |
| F | 0.99082900 | 3.00943300 | 3.31053300 |
| F | 2.45479700 | 4.46749600 | 3.98824200 |
| F | 0.33121600 | 4.93781000 | 4.08208300 |

| | | | |
|---|---|---|---|
| O | -2.95493000 | 1.74114900 | 4.22830000 |
| S | -3.20293900 | 3.14090500 | 3.86209200 |
| O | -3.24905300 | 4.13390500 | 4.95293800 |
| O | -2.40177300 | 3.60739500 | 2.66776200 |
| C | -4.92420400 | 3.16690900 | 3.16230300 |
| F | -5.06082900 | 2.26764300 | 2.18197100 |
| F | -5.18750800 | 4.39011900 | 2.64914100 |
| F | -5.82959200 | 2.91006200 | 4.11322000 |
| O | -4.46794100 | 3.77565200 | -1.79728800 |
| S | -3.35489400 | 4.67461100 | -1.45849400 |
| O | -3.53078800 | 6.10899000 | -1.75996400 |
| O | -2.77279500 | 4.42018900 | -0.08713400 |
| C | -1.95748200 | 4.13240600 | -2.55454600 |
| F | -1.61523400 | 2.86471900 | -2.29243300 |
| F | -0.88240900 | 4.91315100 | -2.36542800 |
| F | -2.32322100 | 4.22070100 | -3.84118000 |
| La | -2.22038500 | 6.06637100 | 1.83647800 |

Energies (0K) = -2916.21933

Energies (0K) + ZPE = -2916.133254

Enthalpies (298K) = -2916.106048

Free Energies (298K) = -2916.19435

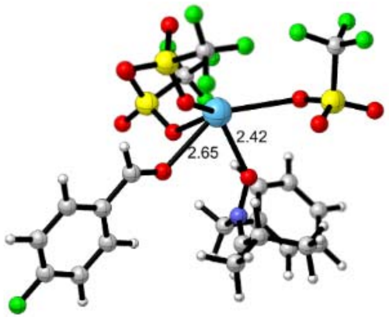


**A = La(OTf)$_3$(1a)(FBA)**

Number of imaginary frequencies = 0

0 1

| | | | |
|---|---|---|---|
| O | -4.12668400 | 2.18959200 | 1.67919400 |
| O | 1.37309300 | 2.18460500 | 0.58944000 |
| S | 0.28956300 | 3.12855900 | 0.29046500 |
| O | 0.33034400 | 3.83417300 | -1.00459700 |
| O | -1.08521400 | 2.59325600 | 0.63084100 |
| C | 0.46405500 | 4.49058300 | 1.54351800 |
| F | 0.43498600 | 4.00299400 | 2.79152600 |
| F | 1.61178000 | 5.15249600 | 1.36876700 |
| F | -0.55622900 | 5.36530900 | 1.41072400 |
| O | -7.04753000 | 4.63457400 | 0.83397400 |
| S | -6.28892300 | 5.49528700 | 1.76293300 |

| | | | |
|---|---|---|---|
| O | -6.88191800 | 5.74217900 | 3.08539700 |
| O | -4.81579800 | 5.17544100 | 1.81267300 |
| C | -6.27022500 | 7.15788200 | 0.93529500 |
| F | -5.69660100 | 7.06577700 | -0.27877900 |
| F | -5.57154900 | 8.04159300 | 1.66193200 |
| F | -7.51626700 | 7.62350300 | 0.77860200 |
| O | -3.38210500 | 5.69937700 | -3.36843000 |
| S | -4.27760600 | 4.56709700 | -3.67717500 |
| O | -4.03745900 | 3.84744300 | -4.93730400 |
| O | -4.51152500 | 3.64656300 | -2.50129400 |
| C | -5.95566400 | 5.33665800 | -3.88111600 |
| F | -6.32032400 | 5.97011900 | -2.75578200 |
| F | -6.87273500 | 4.39960300 | -4.15668300 |
| F | -5.93596600 | 6.22604000 | -4.88284800 |
| O | -3.19692300 | 1.31094800 | -1.06325700 |
| C | -3.13319700 | 0.89460200 | -2.23073900 |
| H | -3.27623600 | 1.59777600 | -3.06365500 |
| C | -2.88159100 | -0.49165200 | -2.59079600 |
| C | -2.66889600 | -1.48633100 | -1.61408000 |
| C | -2.85904900 | -0.83192500 | -3.95693100 |
| C | -2.43803500 | -2.79929300 | -1.99645600 |
| H | -2.68537300 | -1.21866900 | -0.56284800 |
| C | -2.63008300 | -2.14482100 | -4.35199400 |
| H | -3.02349800 | -0.06071900 | -4.70448800 |
| C | -2.42509600 | -3.10030500 | -3.36008100 |
| H | -2.26997500 | -3.58765000 | -1.27038000 |
| H | -2.60820200 | -2.43501200 | -5.39687500 |
| La | -3.38124600 | 3.74303800 | -0.02638100 |
| C | -2.94712300 | 0.38212500 | 2.65204200 |
| C | -5.14261200 | 0.12980200 | 1.60812400 |
| H | -5.04822100 | -0.91064400 | 1.90244000 |
| N | -4.14201000 | 0.88848900 | 1.91830200 |
| C | -6.88875900 | -0.43856700 | -0.06798500 |
| H | -7.11929500 | -1.38825700 | 0.42981500 |
| H | -7.80316900 | -0.07907800 | -0.55231800 |
| H | -6.14486300 | -0.63391100 | -0.84834100 |
| C | -7.45678700 | 0.90690400 | 2.01847200 |
| H | -8.36938300 | 1.27310900 | 1.53520700 |
| H | -7.70870200 | 0.00240600 | 2.58428300 |
| H | -7.11080700 | 1.67058100 | 2.72275700 |
| H | -3.01776300 | -0.70721600 | 2.66018300 |
| H | -2.08478700 | 0.68764000 | 2.05660600 |
| C | -2.87844800 | 0.94919800 | 4.05293100 |
| C | -3.72123400 | 0.45334700 | 5.05772700 |

| | | | |
|---|---|---|---|
| C | -1.97280100 | 1.97162900 | 4.36136900 |
| C | -3.66290500 | 0.97712700 | 6.35013500 |
| H | -4.42220600 | -0.34571000 | 4.82757000 |
| C | -1.91117400 | 2.49399800 | 5.65625600 |
| H | -1.31158500 | 2.35318800 | 3.58918800 |
| C | -2.75739900 | 1.99950100 | 6.65172300 |
| H | -4.31938400 | 0.58476500 | 7.12219900 |
| H | -1.20185600 | 3.28502200 | 5.88496400 |
| H | -2.70984200 | 2.40488400 | 7.65893700 |
| C | -6.38991900 | 0.60907100 | 0.93965000 |
| H | -6.16846000 | 1.54368700 | 0.41624100 |
| F | -2.20277600 | -4.37404400 | -3.73028500 |

Energies (0K) = -3919.1630476

Energies (0K) + ZPE = -3918.730808

Enthalpies (298K) = -3918.67988

Free Energies (298K) = -3918.82543

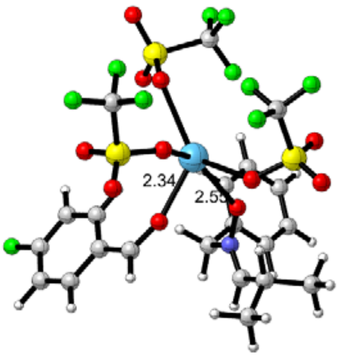


**B = [La(OTf)$_3$(1a)(FBA)]$^-$**

Number of imaginary frequencies = 0

-1 2

| | | | |
|---|---|---|---|
| C | -1.73786000 | -3.81472900 | 1.30938100 |
| C | -3.64494600 | -2.55024000 | 0.47576300 |
| N | -2.45688600 | -2.54809500 | 0.99075700 |
| O | -1.80756400 | -1.44885300 | 1.31374300 |
| O | 4.77538700 | 1.17491200 | 0.89524200 |
| S | 3.47290700 | 0.52846200 | 1.10854700 |
| O | 2.39086300 | 1.01772200 | 0.18197800 |
| O | 3.46642200 | -0.93677100 | 1.29008800 |
| C | 2.88822400 | 1.18073100 | 2.74906100 |
| F | 3.72947100 | 0.82977400 | 3.72834100 |
| F | 2.78561000 | 2.51619700 | 2.72759300 |
| F | 1.66829100 | 0.67496000 | 3.04292100 |
| O | -3.84872600 | 3.18066900 | 0.76219600 |
| S | -2.53781100 | 2.58322500 | 1.05156500 |
| O | -2.23965100 | 2.24642000 | 2.45557300 |

| | | | |
|---|---|---|---|
| O | -2.15031700 | 1.49771400 | 0.07454100 |
| C | -1.30065000 | 3.90904800 | 0.64773200 |
| F | -1.43000700 | 4.33448700 | -0.61395500 |
| F | -0.05085100 | 3.42442500 | 0.80647100 |
| F | -1.45282100 | 4.95476300 | 1.47029800 |
| O | 0.07033400 | 1.49965000 | -1.94737400 |
| S | 0.68419100 | 1.33229800 | -3.31501100 |
| O | 1.66550800 | 0.23573400 | -3.40032100 |
| O | -0.28923300 | 1.44591700 | -4.41349100 |
| C | 1.69980900 | 2.88268400 | -3.43491800 |
| F | 0.92037200 | 3.96724800 | -3.30508800 |
| F | 2.31347500 | 2.94198900 | -4.62723200 |
| F | 2.63522700 | 2.91755100 | -2.47628600 |
| O | -1.01131000 | -1.40252200 | -1.59273400 |
| C | -1.09424900 | -2.36630400 | -2.47754100 |
| C | -0.19775400 | -3.46231200 | -2.56718000 |
| C | -0.38897400 | -4.47120300 | -3.56319800 |
| C | 0.91880200 | -3.62220000 | -1.68644400 |
| C | 0.46484200 | -5.55692600 | -3.67139100 |
| H | -1.22691700 | -4.37959600 | -4.25071100 |
| C | 1.77568700 | -4.71274000 | -1.79504200 |
| H | 1.10583600 | -2.87853500 | -0.91693000 |
| C | 1.54041700 | -5.66617300 | -2.78320500 |
| H | 0.31531500 | -6.32037400 | -4.42959600 |
| H | 2.62306200 | -4.83223700 | -1.12548800 |
| La | -0.07638100 | -0.05070700 | 0.07819300 |
| H | -4.07629900 | -3.52956700 | 0.29447000 |
| H | -1.92223300 | -2.31493800 | -3.19353500 |
| C | -5.40015000 | -1.03485100 | 1.34986200 |
| H | -6.11305700 | -1.85745200 | 1.48286100 |
| H | -4.85714600 | -0.89511600 | 2.29104300 |
| H | -5.96915900 | -0.12070100 | 1.14483800 |
| C | -5.19191500 | -1.47837100 | -1.14833000 |
| H | -5.90235700 | -2.31319100 | -1.10167700 |
| H | -5.75730700 | -0.56553800 | -1.36715700 |
| H | -4.50595900 | -1.66025000 | -1.98345100 |
| C | -4.43192800 | -1.31549500 | 0.17808700 |
| H | -3.73768300 | -0.47525600 | 0.09978900 |
| H | -2.34853300 | -4.63668600 | 0.93123500 |
| H | -0.80291300 | -3.77730100 | 0.74533700 |
| C | -1.48005700 | -3.94867900 | 2.79249900 |
| C | -2.50352600 | -4.37355900 | 3.65131300 |
| C | -0.22062100 | -3.64949900 | 3.32702500 |
| C | -2.27306700 | -4.48931900 | 5.02326200 |

| | | | |
|---|---|---|---|
| H | -3.48223800 | -4.61548700 | 3.24333800 |
| C | 0.01297100 | -3.76766200 | 4.69970900 |
| H | 0.58193100 | -3.33430500 | 2.66453200 |
| C | -1.01400300 | -4.18519300 | 5.55024300 |
| H | -3.07340000 | -4.82115800 | 5.67934600 |
| H | 0.99559500 | -3.53674500 | 5.10246700 |
| H | -0.83331600 | -4.27824400 | 6.61783200 |
| F | 2.37959300 | -6.73583600 | -2.88979100 |

Energies (0K) = -3919.2608022

Energies (0K) + ZPE = -3918.832089

Enthalpies (298K) = -3918.780946

Free Energies (298K) = -3918.925805

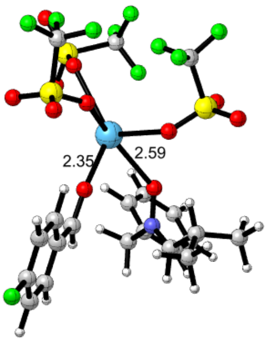


**B′**

**(another isomer of [La(OTf)$_3$(1a)(FBA)]$^-$)**

Number of imaginary frequencies = 0

-1 2

| | | | |
|---|---|---|---|
| C | -4.28471900 | -2.08593300 | 2.59075700 |
| C | -6.29156100 | -0.87496700 | 1.92897900 |
| N | -5.05292000 | -0.83991900 | 2.30764100 |
| O | -4.38876500 | 0.27685600 | 2.52108600 |
| O | 2.09611000 | 3.33878900 | 1.90877900 |
| S | 0.84152200 | 2.60460200 | 2.13454300 |
| O | -0.23757900 | 2.91340400 | 1.13206500 |
| O | 0.95709400 | 1.16836500 | 2.46332000 |
| C | 0.13371700 | 3.35019400 | 3.68182000 |
| F | 0.94196400 | 3.14584600 | 4.72920300 |
| F | -0.06058300 | 4.66860900 | 3.54113100 |
| F | -1.06296000 | 2.78288300 | 3.95967700 |
| O | -6.55681200 | 4.65963100 | 1.96033800 |
| S | -5.17002200 | 4.19702900 | 2.10939200 |
| O | -4.68752800 | 3.92690700 | 3.47641500 |
| O | -4.79104000 | 3.12424400 | 1.11484600 |
| C | -4.12207500 | 5.62178300 | 1.53813000 |
| F | -4.44782900 | 5.99628700 | 0.29556700 |
| F | -2.82200800 | 5.26908700 | 1.54993700 |

| | | | |
|---|---|---|---|
| F | -4.28109600 | 6.67112100 | 2.35631300 |
| O | -2.59363200 | 3.15028700 | -0.92028300 |
| S | -1.99695400 | 3.34169500 | -2.28939000 |
| O | -0.84355800 | 2.47028700 | -2.57551300 |
| O | -3.00946100 | 3.47604800 | -3.35120500 |
| C | -1.28064900 | 5.04767400 | -2.12378800 |
| F | -2.23941400 | 5.92983100 | -1.80191800 |
| F | -0.72616100 | 5.42896900 | -3.28590500 |
| F | -0.33759700 | 5.08358200 | -1.17415200 |
| O | -3.64895200 | 0.23155300 | -0.53925500 |
| C | -4.08987700 | -0.88808100 | -1.06720300 |
| C | -4.61335500 | -0.98920400 | -2.38269200 |
| C | -5.08587100 | -2.24402800 | -2.87840800 |
| C | -4.69375300 | 0.13617400 | -3.26139500 |
| C | -5.60120500 | -2.36942700 | -4.15979400 |
| H | -5.03862600 | -3.11876600 | -2.23329100 |
| C | -5.20902100 | 0.00817100 | -4.54548000 |
| H | -4.33951000 | 1.10241700 | -2.91735400 |
| C | -5.65672900 | -1.23915000 | -4.98013000 |
| H | -5.96000100 | -3.32402900 | -4.53428200 |
| H | -5.26681600 | 0.86162500 | -5.21560600 |
| La | -2.69792900 | 1.61903700 | 1.09810900 |
| H | -6.72593800 | -1.86489400 | 1.83198600 |
| H | -4.04626600 | -1.79733200 | -0.45635300 |
| C | -8.05362700 | 0.60207000 | 2.86082300 |
| H | -8.74280900 | -0.23610600 | 3.01927600 |
| H | -7.48429400 | 0.75768400 | 3.78385400 |
| H | -8.64944400 | 1.50234100 | 2.67151000 |
| C | -7.91792500 | 0.15484900 | 0.35842700 |
| H | -8.60822100 | -0.69481900 | 0.43151000 |
| H | -8.50966700 | 1.05482100 | 0.15643900 |
| H | -7.25548600 | -0.01592500 | -0.49743500 |
| C | -7.11834200 | 0.33992500 | 1.65958300 |
| H | -6.44463400 | 1.19347400 | 1.55266900 |
| H | -4.90714400 | -2.92758200 | 2.28108500 |
| H | -3.40030600 | -2.04093900 | 1.95052500 |
| C | -3.90090900 | -2.18399900 | 4.04935800 |
| C | -4.85153900 | -2.57530700 | 5.00285000 |
| C | -2.59651400 | -1.89048500 | 4.46561700 |
| C | -4.50438500 | -2.66389800 | 6.35186800 |
| H | -5.86522600 | -2.81105800 | 4.68720900 |
| C | -2.24629400 | -1.98197200 | 5.81556100 |
| H | -1.85072500 | -1.59995800 | 3.72940400 |
| C | -3.20035900 | -2.36695900 | 6.76083000 |

| | | | |
|---|---|---|---|
| H | -5.24886600 | -2.96967600 | 7.08214900 |
| H | -1.22983200 | -1.75543400 | 6.12624900 |
| H | -2.92925500 | -2.43925400 | 7.81077900 |
| F | -6.16204300 | -1.36189600 | -6.24127200 |

Energies (0K) = -3919.2597517

Energies (0K) + ZPE = -3918.831408

Enthalpies (298K) = -3918.779906

Free Energies (298K) = -3918.927138

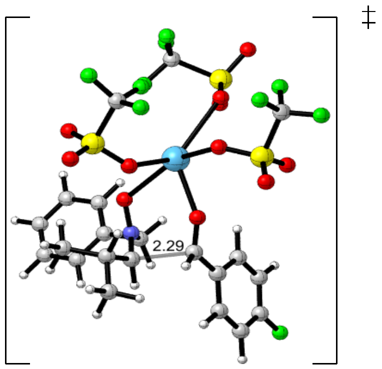


**$TS_B$**

Number of imaginary frequencies = 1

-1 2

| | | | |
|---|---|---|---|
| C | -3.76672600 | -1.95735700 | 2.65242000 |
| C | -5.49890600 | -0.95503300 | 1.19719300 |
| N | -4.54376100 | -0.82225700 | 2.12538100 |
| O | -4.18493000 | 0.37948100 | 2.59237600 |
| O | 2.15582400 | 3.68305100 | 2.27611000 |
| S | 0.89934300 | 2.94536000 | 2.48056200 |
| O | -0.15908900 | 3.24024100 | 1.45326200 |
| O | 1.01449400 | 1.51221000 | 2.82327600 |
| C | 0.16069100 | 3.70152900 | 4.00923500 |
| F | 0.95536600 | 3.51726500 | 5.07066400 |
| F | -0.04348200 | 5.01649000 | 3.84994800 |
| F | -1.03504900 | 3.12769100 | 4.27833800 |
| O | -6.84081700 | 4.63077800 | 1.75596100 |
| S | -5.47934400 | 4.23860700 | 2.14642900 |
| O | -5.23248400 | 3.97380200 | 3.57617400 |
| O | -4.86719700 | 3.20898500 | 1.22717200 |
| C | -4.42693200 | 5.72767900 | 1.78887000 |
| F | -4.55790100 | 6.12095500 | 0.51636900 |
| F | -3.12804200 | 5.43268200 | 2.00888900 |
| F | -4.76526600 | 6.74709600 | 2.58867700 |
| O | -2.48394900 | 3.43592900 | -0.65898400 |
| S | -1.88615000 | 3.24718000 | -2.03023200 |
| O | -0.86418300 | 2.18658600 | -2.09908300 |

| | | | |
|---|---|---|---|
| O | -2.87978700 | 3.28702900 | -3.11624300 |
| C | -0.93150500 | 4.82852800 | -2.22651400 |
| F | -1.75008800 | 5.88784000 | -2.12939400 |
| F | -0.34094900 | 4.86024400 | -3.43214200 |
| F | 0.01842700 | 4.94049300 | -1.28908400 |
| O | -3.54224700 | 0.51718900 | -0.25836000 |
| C | -4.29932900 | -0.45455900 | -0.68242900 |
| C | -3.72497700 | -1.75161900 | -1.05350700 |
| C | -4.50641900 | -2.70422900 | -1.74379500 |
| C | -2.39371500 | -2.08814400 | -0.72825400 |
| C | -3.98749900 | -3.94771400 | -2.09029900 |
| H | -5.53163600 | -2.46040000 | -2.01162200 |
| C | -1.86223800 | -3.33101100 | -1.06895200 |
| H | -1.77318800 | -1.36213500 | -0.21198600 |
| C | -2.67010300 | -4.24074000 | -1.74283600 |
| H | -4.58133100 | -4.68309300 | -2.62449200 |
| H | -0.83789100 | -3.59672600 | -0.82545000 |
| La | -2.62827000 | 1.92347800 | 1.41446600 |
| H | -5.73893200 | -1.99017500 | 0.97346700 |
| H | -5.21413600 | -0.17660100 | -1.21442900 |
| C | -7.31026800 | 0.23669100 | 2.46441600 |
| H | -7.77777800 | -0.70041200 | 2.79189800 |
| H | -6.59522100 | 0.55454600 | 3.22775500 |
| H | -8.09655400 | 0.99822600 | 2.39266900 |
| C | -7.65667600 | -0.36416800 | 0.04257000 |
| H | -8.11050600 | -1.32708400 | 0.31097900 |
| H | -8.46099000 | 0.37805000 | -0.01601200 |
| H | -7.22755100 | -0.46488100 | -0.95958800 |
| C | -6.62091900 | 0.06367900 | 1.09325800 |
| H | -6.19524900 | 1.03268300 | 0.81201800 |
| H | -3.99159300 | -2.82745200 | 2.03213600 |
| H | -2.70679100 | -1.71003100 | 2.54040100 |
| C | -4.09989400 | -2.23192400 | 4.10666500 |
| C | -5.26564900 | -2.93601800 | 4.44234400 |
| C | -3.26245200 | -1.77656100 | 5.13248200 |
| C | -5.59120000 | -3.17366500 | 5.77879000 |
| H | -5.91910700 | -3.30133600 | 3.65341900 |
| C | -3.58552300 | -2.01430500 | 6.47179700 |
| H | -2.34985400 | -1.24082200 | 4.88117300 |
| C | -4.75138100 | -2.71173200 | 6.79738300 |
| H | -6.49615500 | -3.72318300 | 6.02492000 |
| H | -2.92616600 | -1.65655100 | 7.25831400 |
| H | -5.00337700 | -2.89823700 | 7.83803900 |
| F | -2.15817100 | -5.45253700 | -2.07859200 |

Energies (0K) = -3919.2488226
Energies (0K) + ZPE = -3918.820054
Enthalpies (298K) = -3918.770045
Free Energies (298K) = -3918.912131

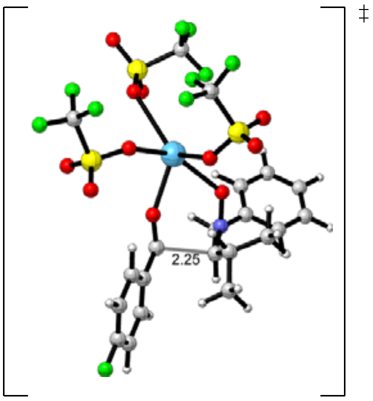


**TS$_{B'}$**
Number of imaginary frequencies = 1
-1 2

| | | | |
|---|---|---|---|
| C | -3.90130700 | -2.04322800 | 2.45586800 |
| C | -5.57533500 | -0.93308200 | 1.02455500 |
| N | -4.66532800 | -0.86712800 | 2.01558300 |
| O | -4.17137000 | 0.30816600 | 2.42729600 |
| O | 2.17610400 | 3.55119000 | 2.06512200 |
| S | 0.92164000 | 2.81389200 | 2.28246600 |
| O | -0.15272900 | 3.12288000 | 1.27529200 |
| O | 1.03794100 | 1.37675800 | 2.60677900 |
| C | 0.20803200 | 3.55251200 | 3.83086400 |
| F | 1.01718200 | 3.34674800 | 4.87774400 |
| F | 0.00982000 | 4.87037300 | 3.69549100 |
| F | -0.98606200 | 2.97929400 | 4.10722900 |
| O | -6.44998600 | 5.00269800 | 1.91986800 |
| S | -5.14330300 | 4.41195400 | 2.24105500 |
| O | -4.88269200 | 4.06990600 | 3.65137500 |
| O | -4.72089900 | 3.33614600 | 1.26865700 |
| C | -3.90628300 | 5.74883200 | 1.87594700 |
| F | -4.02850900 | 6.20017700 | 0.62210400 |
| F | -2.65593300 | 5.26515600 | 2.03142000 |
| F | -4.06661200 | 6.77730100 | 2.71858900 |
| O | -2.52630200 | 3.32984600 | -0.80591300 |
| S | -2.15076300 | 3.28360600 | -2.26426500 |
| O | -1.07779500 | 2.32348900 | -2.57968600 |
| O | -3.30971900 | 3.31448500 | -3.17329700 |
| C | -1.36435000 | 4.95482500 | -2.47285200 |
| F | -2.22172400 | 5.92709400 | -2.12750900 |

| | | | |
|---|---|---|---|
| F | -1.00981700 | 5.13799900 | -3.75454500 |
| F | -0.26672500 | 5.06856800 | -1.71142700 |
| O | -3.58251300 | 0.42901100 | -0.44255300 |
| C | -4.13193900 | -0.72827800 | -0.69125200 |
| C | -4.89960900 | -0.92600300 | -1.92540200 |
| C | -5.29596800 | -2.22364600 | -2.31374600 |
| C | -5.25198800 | 0.16329900 | -2.74986000 |
| C | -6.03557900 | -2.43199400 | -3.47383800 |
| H | -5.01913500 | -3.07513200 | -1.69632800 |
| C | -5.99106600 | -0.03380700 | -3.91472500 |
| H | -4.92419300 | 1.16145500 | -2.47897900 |
| C | -6.37522800 | -1.32759400 | -4.25272800 |
| H | -6.34397900 | -3.42612500 | -3.78254400 |
| H | -6.26367000 | 0.79550700 | -4.56061900 |
| La | -2.62421800 | 1.81878700 | 1.24704200 |
| H | -5.90690100 | -1.94903100 | 0.82867800 |
| H | -3.62130500 | -1.62676700 | -0.32396600 |
| C | -7.18375400 | 0.65338200 | 2.17133300 |
| H | -7.74672700 | -0.15656000 | 2.65278700 |
| H | -6.40604600 | 0.99280300 | 2.85792000 |
| H | -7.87702800 | 1.48502500 | 1.99561700 |
| C | -7.72366100 | -0.28260200 | -0.09596100 |
| H | -8.29205700 | -1.09620300 | 0.37342300 |
| H | -8.41900900 | 0.54439600 | -0.28150000 |
| H | -7.36194700 | -0.63891000 | -1.06292600 |
| C | -6.58642300 | 0.18303200 | 0.82801100 |
| H | -6.07713300 | 1.04069900 | 0.37100000 |
| H | -4.35679600 | -2.91911200 | 1.98657300 |
| H | -2.87748300 | -1.94032500 | 2.07541600 |
| C | -3.88818100 | -2.19095200 | 3.96400900 |
| C | -5.07416100 | -2.47321000 | 4.65772400 |
| C | -2.69269300 | -2.07078300 | 4.68169300 |
| C | -5.06329700 | -2.62786400 | 6.04447400 |
| H | -6.00758600 | -2.57117800 | 4.10814400 |
| C | -2.67843300 | -2.22985800 | 6.07115700 |
| H | -1.76750500 | -1.85965700 | 4.14977500 |
| C | -3.86379100 | -2.50797200 | 6.75493200 |
| H | -5.98863800 | -2.84693200 | 6.57101100 |
| H | -1.74250200 | -2.13614500 | 6.61573400 |
| H | -3.85518600 | -2.63250900 | 7.83455400 |
| F | -7.09543900 | -1.52423800 | -5.38620600 |

Energies (0K) = -3919.247013

Energies (0K) + ZPE = -3918.817904

Enthalpies (298K) = -3918.768102

Free Energies (298K) = -3918.909155

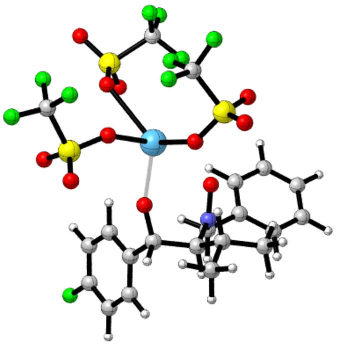

**C**

Number of imaginary frequencies = 0

-1 2

| | | | |
|---|---|---|---|
| C | -1.38061900 | -3.97521900 | 1.32233900 |
| C | -2.68335900 | -2.61574900 | -0.45000200 |
| N | -1.93205800 | -2.70233500 | 0.81129700 |
| O | -1.48471300 | -1.61240300 | 1.34400800 |
| O | 5.02772200 | 1.48830100 | 0.91703000 |
| S | 3.71274300 | 0.87876800 | 1.16282400 |
| O | 2.67926100 | 1.20774000 | 0.11689300 |
| O | 3.68896400 | -0.53738200 | 1.58298400 |
| C | 3.04174100 | 1.77421700 | 2.64499500 |
| F | 3.83522100 | 1.60185900 | 3.70889700 |
| F | 2.91963400 | 3.08633400 | 2.40797500 |
| F | 1.81738200 | 1.28772700 | 2.95195100 |
| O | -3.61346800 | 3.28590200 | 0.82606900 |
| S | -2.30568800 | 2.68536300 | 1.12377100 |
| O | -2.03082000 | 2.32357300 | 2.52690700 |
| O | -1.89967700 | 1.61835700 | 0.13474900 |
| C | -1.07020800 | 4.02545400 | 0.76012300 |
| F | -1.20627500 | 4.48951100 | -0.48769400 |
| F | 0.18338000 | 3.54384100 | 0.89775600 |
| F | -1.22393700 | 5.04498800 | 1.61477300 |
| O | 0.34532400 | 1.66054600 | -1.91897400 |
| S | 0.73638800 | 1.55895700 | -3.37089900 |
| O | 1.79885900 | 0.57221800 | -3.63848900 |
| O | -0.40805300 | 1.57421300 | -4.29705800 |
| C | 1.54975800 | 3.21086400 | -3.61904200 |
| F | 0.70507200 | 4.20334300 | -3.30068300 |
| F | 1.91370500 | 3.35911800 | -4.90232400 |
| F | 2.64506900 | 3.32585400 | -2.85378500 |
| O | -0.96347700 | -1.16501900 | -1.40606200 |
| C | -1.67738300 | -2.31595300 | -1.63619000 |
| C | -0.78988300 | -3.53259400 | -1.94162300 |
| C | -1.35202300 | -4.69176800 | -2.50078600 |

| | | | |
|---|---|---|---|
| C | 0.58994400 | -3.51218600 | -1.70824000 |
| C | -0.56932000 | -5.80634800 | -2.80300700 |
| H | -2.41847300 | -4.72433600 | -2.71265600 |
| C | 1.39485600 | -4.61718500 | -2.00380100 |
| H | 1.05020000 | -2.61637600 | -1.30313700 |
| C | 0.79612400 | -5.74650800 | -2.54453000 |
| H | -0.99954500 | -6.70276100 | -3.23894000 |
| H | 2.46613600 | -4.60336400 | -1.82704800 |
| La | 0.20338000 | 0.09750900 | 0.09190200 |
| H | -3.12360300 | -3.60509100 | -0.60908100 |
| H | -2.33022000 | -2.20055000 | -2.51741300 |
| C | -4.75296300 | -1.93833900 | 0.85375800 |
| H | -5.22986900 | -2.91569700 | 0.69804700 |
| H | -4.21311200 | -1.97337700 | 1.80571200 |
| H | -5.55115500 | -1.19289500 | 0.95175100 |
| C | -4.64747700 | -1.47613000 | -1.61693400 |
| H | -5.02117900 | -2.45808200 | -1.93721300 |
| H | -5.51771600 | -0.82971500 | -1.45186800 |
| H | -4.07554700 | -1.04375300 | -2.44417800 |
| C | -3.82800100 | -1.57923000 | -0.32113000 |
| H | -3.37421200 | -0.60336600 | -0.12211000 |
| H | -1.92066700 | -4.77910100 | 0.81817900 |
| H | -0.32886100 | -4.02599300 | 1.01577400 |
| C | -1.49288400 | -4.10289000 | 2.82576000 |
| C | -2.69049200 | -4.53184600 | 3.41547900 |
| C | -0.39650800 | -3.81128800 | 3.64754100 |
| C | -2.79093300 | -4.66119400 | 4.80228400 |
| H | -3.54363600 | -4.77109300 | 2.78505500 |
| C | -0.49422300 | -3.94037000 | 5.03556000 |
| H | 0.54095000 | -3.49365800 | 3.19686300 |
| C | -1.69245300 | -4.36521100 | 5.61538700 |
| H | -3.72294900 | -4.99993600 | 5.24701600 |
| H | 0.36559900 | -3.71499600 | 5.66105600 |
| H | -1.76857000 | -4.47120400 | 6.69435600 |
| F | 1.56732600 | -6.82407400 | -2.83991400 |

Energies (0K) = -3919.2726993

Energies (0K) + ZPE = -3918.840519

Enthalpies (298K) = -3918.790774

Free Energies (298K) = -3918.932745

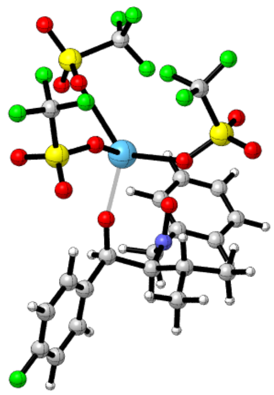

**C′**

Number of imaginary frequencies = 0

-1 2

| | | | |
|---|---|---|---|
| C | -4.48102300 | -2.23154900 | 2.57018600 |
| C | -5.60208600 | -0.80368000 | 0.75973600 |
| N | -4.90828800 | -0.90879400 | 2.05826200 |
| O | -4.30399700 | 0.13478600 | 2.51732400 |
| O | 2.25219600 | 3.44034100 | 1.61604400 |
| S | 1.01272000 | 2.70847900 | 1.91985600 |
| O | -0.15735800 | 3.09924400 | 1.05979200 |
| O | 1.13096000 | 1.25111700 | 2.13543000 |
| C | 0.48767200 | 3.35528400 | 3.58183700 |
| F | 1.39445400 | 3.05877200 | 4.52096200 |
| F | 0.32093300 | 4.68473200 | 3.55228700 |
| F | -0.69052600 | 2.79894000 | 3.94910600 |
| O | -6.39131300 | 4.95924400 | 2.42787700 |
| S | -5.04228100 | 4.39172300 | 2.56018800 |
| O | -4.59209400 | 4.02011000 | 3.91493400 |
| O | -4.72694200 | 3.34891400 | 1.51439600 |
| C | -3.89390500 | 5.77103300 | 2.07517100 |
| F | -4.19752500 | 6.25180000 | 0.86367200 |
| F | -2.62135600 | 5.32409600 | 2.04565000 |
| F | -3.96678000 | 6.77059500 | 2.96375600 |
| O | -2.70350300 | 3.44191100 | -0.72946600 |
| S | -2.40456200 | 3.42926300 | -2.20710100 |
| O | -1.31131700 | 2.51941100 | -2.59198000 |
| O | -3.61198600 | 3.41794800 | -3.05127800 |
| C | -1.70396700 | 5.13539800 | -2.43239900 |
| F | -2.58421600 | 6.06372600 | -2.02986700 |
| F | -1.42424400 | 5.34764100 | -3.72760000 |
| F | -0.57452400 | 5.28659600 | -1.72634000 |
| O | -3.85003600 | 0.57902600 | -0.26054300 |
| C | -4.46206000 | -0.65379200 | -0.33360100 |
| C | -4.94411200 | -0.99912600 | -1.74831500 |
| C | -5.40733100 | -2.28856400 | -2.05208300 |
| C | -4.86870900 | -0.05453800 | -2.77923200 |

| | | | |
|---|---|---|---|
| C | -5.80957400 | -2.62750700 | -3.34511500 |
| H | -5.45117700 | -3.04734600 | -1.27399600 |
| C | -5.26541800 | -0.37470900 | -4.08092500 |
| H | -4.49815100 | 0.94010000 | -2.55592900 |
| C | -5.73198100 | -1.65706600 | -4.33787300 |
| H | -6.16891900 | -3.62310200 | -3.58694300 |
| H | -5.21161900 | 0.35337000 | -4.88499600 |
| La | -2.63454100 | 1.83982000 | 1.21896800 |
| H | -6.09791000 | -1.76944200 | 0.61832200 |
| H | -3.74802100 | -1.46070400 | -0.07566400 |
| C | -7.54257200 | 0.17469400 | 2.08441400 |
| H | -8.06586000 | -0.79109000 | 2.10325700 |
| H | -6.95564200 | 0.25998400 | 3.00413700 |
| H | -8.30419600 | 0.96354000 | 2.10119700 |
| C | -7.57895900 | 0.31939200 | -0.41822100 |
| H | -8.06579200 | -0.65417700 | -0.56275600 |
| H | -8.37050000 | 1.06795500 | -0.29027900 |
| H | -7.03797500 | 0.56506600 | -1.33434400 |
| C | -6.67235000 | 0.31458600 | 0.82258000 |
| H | -6.14812200 | 1.27368100 | 0.87280900 |
| H | -5.29615200 | -2.92622300 | 2.35188900 |
| H | -3.60313400 | -2.54754300 | 1.98925200 |
| C | -4.15402200 | -2.21271400 | 4.04396600 |
| C | -5.17549200 | -2.29134600 | 5.00084800 |
| C | -2.82359300 | -2.13034600 | 4.47484900 |
| C | -4.87192800 | -2.27765400 | 6.36384100 |
| H | -6.21087100 | -2.36493100 | 4.67660900 |
| C | -2.51677100 | -2.11807600 | 5.83806300 |
| H | -2.02442500 | -2.08471700 | 3.73834300 |
| C | -3.54157400 | -2.19035600 | 6.78534700 |
| H | -5.67246900 | -2.34069900 | 7.09629900 |
| H | -1.47991000 | -2.05753600 | 6.15836100 |
| H | -3.30510700 | -2.18402900 | 7.84614000 |
| F | -6.11863000 | -1.97840500 | -5.60030400 |

Energies (0K) = -3919.2694203

Energies (0K) + ZPE = -3918.837488

Enthalpies (298K) = -3918.787476

Free Energies (298K) = -3918.930594

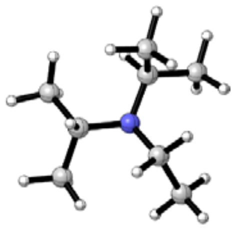

***i*Pr$_2$NEt (*i.e.*, DIPEA)**

Number of imaginary frequencies = 0

0 1

| | | | |
|---|---|---|---|
| C | 1.36283600 | -0.23304000 | 0.27129300 |
| H | 1.36971900 | -0.30922000 | 1.37654000 |
| N | 0.12243400 | 0.43565300 | -0.18989800 |
| C | 2.59729900 | 0.58782400 | -0.13720200 |
| H | 2.58126700 | 1.60440500 | 0.26689500 |
| H | 3.50726800 | 0.10223000 | 0.23496400 |
| H | 2.66770800 | 0.65629700 | -1.23014800 |
| C | -0.06245900 | 1.77028300 | 0.42095300 |
| H | 0.63570500 | 1.90316800 | 1.25896800 |
| H | -1.06181900 | 1.84391400 | 0.86337000 |
| C | -1.10016800 | -0.40260900 | -0.16247800 |
| H | -0.84633000 | -1.32268800 | -0.69564400 |
| C | -1.58198800 | -0.80876000 | 1.24548500 |
| H | -2.41719200 | -1.51670400 | 1.17355500 |
| H | -0.78354900 | -1.29659100 | 1.81692100 |
| H | -1.93164100 | 0.05472800 | 1.82446600 |
| C | 1.51923000 | -1.65438100 | -0.28836400 |
| H | 1.43169300 | -1.65769100 | -1.38231900 |
| H | 2.51452300 | -2.03354400 | -0.03037000 |
| H | 0.78955700 | -2.36089400 | 0.11879000 |
| C | -2.23343900 | 0.24761800 | -0.96913900 |
| H | -1.89430600 | 0.50022800 | -1.98044500 |
| H | -3.07525700 | -0.44979500 | -1.05720200 |
| H | -2.61499800 | 1.16097500 | -0.49797700 |
| C | 0.10858100 | 2.92294100 | -0.57457700 |
| H | 1.10607500 | 2.92070300 | -1.02742000 |
| H | -0.62617300 | 2.85233300 | -1.38499200 |
| H | -0.03359100 | 3.88969200 | -0.07296300 |

Energies (0K) = -371.0378683

Energies (0K) + ZPE = -370.775079

Enthalpies (298K) = -370.76227

Free Energies (298K) = -370.811463

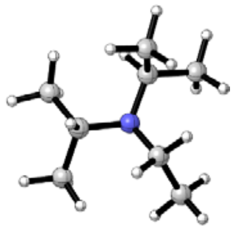

*i*$Pr_2$NEt radical cation, **[*i*$Pr_2$(Et)N·]$^+$**
Number of imaginary frequencies = 1
1 2

| | | | |
|---|---|---|---|
| C | 1.46025700 | -0.20940500 | 0.32733300 |
| H | 1.67090900 | -0.09048500 | 1.40122500 |
| N | 0.12402300 | 0.41238700 | 0.16635000 |
| C | 2.52692800 | 0.59436600 | -0.44701000 |
| H | 2.61699800 | 1.62316500 | -0.09266100 |
| H | 3.48972300 | 0.09989100 | -0.28726900 |
| H | 2.30961400 | 0.59572700 | -1.51959500 |
| C | -0.03834000 | 1.79372900 | 0.62879600 |
| H | 0.75009000 | 1.98300800 | 1.36024600 |
| H | -1.00561300 | 1.85524900 | 1.13373700 |
| C | -1.06266500 | -0.38213800 | -0.18911200 |
| H | -0.69872300 | -1.23882500 | -0.75496400 |
| C | -1.70889300 | -0.90184100 | 1.12146800 |
| H | -2.51823100 | -1.58292800 | 0.84025200 |
| H | -0.99089700 | -1.44753300 | 1.73940000 |
| H | -2.12997200 | -0.07688700 | 1.70392800 |
| C | 1.52574200 | -1.69180000 | -0.03094100 |
| H | 1.37642800 | -1.86170100 | -1.10248000 |
| H | 2.53094400 | -2.04131600 | 0.22393100 |
| H | 0.81329000 | -2.29790600 | 0.53434000 |
| C | -2.07693900 | 0.37699300 | -1.04961800 |
| H | -1.62770900 | 0.72581400 | -1.98345600 |
| H | -2.87782400 | -0.32649300 | -1.29900200 |
| H | -2.52751200 | 1.22322700 | -0.52354500 |
| C | 0.02083300 | 2.85756500 | -0.48612800 |
| H | 0.97434200 | 2.83966800 | -1.01732100 |
| H | -0.78969500 | 2.74292100 | -1.20734500 |
| H | -0.08612300 | 3.83262200 | 0.00050400 |

Energies (0K) = -370.8656712
Energies (0K) + ZPE = -370.601661
Enthalpies (298K) = -370.588808
Free Energies (298K) = -370.639223

**[$i$Pr(Et)N=C(Me)$_2$]$^+$**

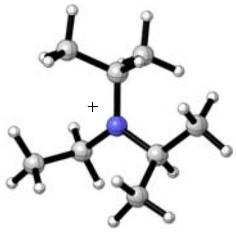


Number of imaginary frequencies = 0

1 1

| | | | |
|---|---|---|---|
| C | 0.96828700 | -0.74542400 | -0.28211700 |
| N | -0.01692200 | 0.02136500 | 0.08953900 |
| C | 2.38684300 | -0.27903200 | -0.32981500 |
| H | 2.54590200 | 0.77433800 | -0.11272100 |
| H | 2.96897700 | -0.88431300 | 0.37720700 |
| H | 2.78107700 | -0.50825200 | -1.32730200 |
| C | 0.19956200 | 1.43539600 | 0.52517200 |
| H | 0.85801600 | 1.91709100 | -0.19898700 |
| H | -0.76591000 | 1.93441400 | 0.45720500 |
| C | -1.44652500 | -0.45265700 | 0.06290400 |
| H | -1.40607800 | -1.51589700 | -0.15694400 |
| C | -2.11974700 | -0.28254300 | 1.42613400 |
| H | -3.11578700 | -0.73396400 | 1.36781200 |
| H | -1.56017400 | -0.79666200 | 2.21397600 |
| H | -2.24759200 | 0.76762900 | 1.70559600 |
| C | 0.75135400 | -2.17319700 | -0.69943900 |
| H | 0.11865200 | -2.22893200 | -1.59261800 |
| H | 1.71190700 | -2.63696000 | -0.92891300 |
| H | 0.26707800 | -2.75033900 | 0.09523600 |
| C | -2.20352900 | 0.23923400 | -1.07593500 |
| H | -1.69394200 | 0.09451600 | -2.03433900 |
| H | -3.20133200 | -0.20602600 | -1.14802800 |
| H | -2.32871700 | 1.31273100 | -0.90209800 |
| C | 0.74899000 | 1.54636400 | 1.94653700 |
| H | 0.06285000 | 1.10709400 | 2.67598400 |
| H | 1.72364100 | 1.06080200 | 2.05142400 |
| H | 0.87043200 | 2.60807200 | 2.18781900 |

Energies (0K) = -370.3107569

Energies (0K) + ZPE = -370.057366

Enthalpies (298K) = -370.04458

Free Energies (298K) = -370.094016

***i*Pr(Et)N-C(Me)=$CH_2$**

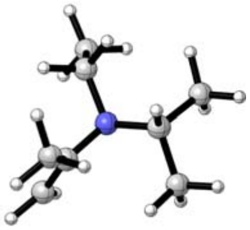

Number of imaginary frequencies = 0

0 1

| | | | |
|---|---|---|---|
| C | 1.14983900 | -0.03273100 | 0.10342800 |
| H | 1.28283000 | -0.15497500 | 1.19521200 |
| N | -0.15979400 | 0.59940200 | -0.19238700 |
| C | 2.30215000 | 0.84227000 | -0.40845800 |
| H | 2.32062700 | 1.83155500 | 0.05890400 |
| H | 3.25976300 | 0.35733100 | -0.18508400 |
| H | 2.23198200 | 0.97696900 | -1.49487200 |
| C | -0.33645100 | 1.94998200 | 0.40035200 |
| H | 0.43580700 | 2.13912400 | 1.15888700 |
| H | -1.29385300 | 1.98395500 | 0.93364100 |
| C | -1.31028900 | -0.23902800 | 0.06437300 |
| C | -1.50930900 | -0.77988700 | 1.46375200 |
| H | -2.44743500 | -1.33797200 | 1.54521600 |
| H | -0.69062500 | -1.44965600 | 1.75565600 |
| H | -1.52832500 | 0.03312900 | 2.20160700 |
| C | 1.24265000 | -1.41689100 | -0.55020800 |
| H | 1.12131600 | -1.33701900 | -1.63765400 |
| H | 2.22623600 | -1.85556600 | -0.34731500 |
| H | 0.48526100 | -2.11279600 | -0.17670200 |
| C | -2.18039400 | -0.46968000 | -0.92561300 |
| H | -3.08186600 | -1.05645100 | -0.76422600 |
| H | -2.01080100 | -0.07315400 | -1.92316300 |
| C | -0.32841200 | 3.06131300 | -0.65092200 |
| H | 0.62093300 | 3.09688300 | -1.19607900 |
| H | -1.13068800 | 2.90455300 | -1.38207500 |
| H | -0.48618600 | 4.03963700 | -0.17809400 |

Energies (0K) = -369.8096337

Energies (0K) + ZPE = -369.570964

Enthalpies (298K) = -369.558477

Free Energies (298K) = -369.607466

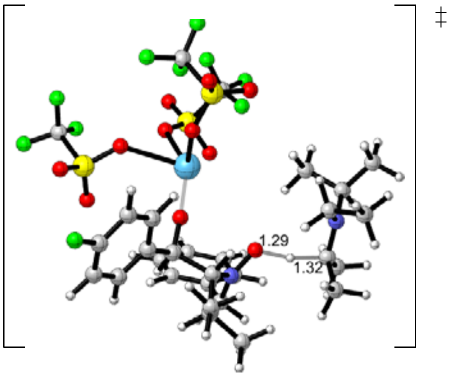


**$TS_C$**

Number of imaginary frequencies = 1

0 1

| | | | |
|---|---|---|---|
| C | -2.04057600 | -4.53632100 | 1.88223900 |
| C | -2.69729800 | -2.82572000 | 0.02655300 |
| N | -2.38043600 | -3.17931700 | 1.41866800 |
| O | -2.26512000 | -2.22525300 | 2.27557900 |
| O | 5.48589700 | -0.62305100 | 3.79605800 |
| S | 4.03357000 | -0.66133600 | 3.56668300 |
| O | 3.65328400 | -0.76551100 | 2.11445600 |
| O | 3.23511800 | -1.52692800 | 4.45635400 |
| C | 3.44902100 | 1.04797500 | 3.99860200 |
| F | 3.68407800 | 1.32156700 | 5.28748400 |
| F | 4.05167800 | 1.97772200 | 3.24676700 |
| F | 2.11447100 | 1.14193200 | 3.78933600 |
| O | -0.27409000 | 3.53922800 | -1.24479800 |
| S | 0.30921500 | 2.73186100 | -0.16499300 |
| O | -0.06845800 | 3.07576700 | 1.21913900 |
| O | 0.27350400 | 1.24713800 | -0.44074200 |
| C | 2.13403500 | 3.08610200 | -0.24070900 |
| F | 2.61556400 | 2.84976000 | -1.46673700 |
| F | 2.80257200 | 2.30129200 | 0.63225800 |
| F | 2.37480800 | 4.36363600 | 0.07678500 |
| O | 2.61508300 | -0.77406000 | -1.01665100 |
| S | 3.16582300 | -1.91756900 | -1.84051200 |
| O | 2.98657100 | -3.23498300 | -1.20187700 |
| O | 2.84523100 | -1.81178000 | -3.27125200 |
| C | 4.99231600 | -1.59011700 | -1.74185400 |
| F | 5.27183000 | -0.34927400 | -2.16368200 |
| F | 5.65347900 | -2.46404200 | -2.51478300 |
| F | 5.42935700 | -1.72193600 | -0.48268200 |
| O | -0.46961900 | -1.88515100 | -0.11611700 |
| C | -1.29270900 | -2.79893400 | -0.73552000 |
| C | -1.41819000 | -2.51886700 | -2.22991600 |
| C | -1.44116400 | -3.57155500 | -3.15485100 |
| C | -1.48001100 | -1.20109800 | -2.70718300 |
| C | -1.55491300 | -3.32736500 | -4.52594100 |

| | | | |
|---|---|---|---|
| H | -1.36555700 | -4.59808700 | -2.80497100 |
| C | -1.59377300 | -0.93615300 | -4.07245000 |
| H | -1.42277600 | -0.37677900 | -2.00324500 |
| C | -1.63540500 | -2.00946800 | -4.95661200 |
| H | -1.56989300 | -4.13675700 | -5.24932800 |
| H | -1.64215900 | 0.07969800 | -4.45272500 |
| La | 1.11282700 | -0.74771700 | 1.03438300 |
| H | -3.04300400 | -1.78996400 | 0.09006100 |
| H | -0.90011900 | -3.82493300 | -0.63712500 |
| C | -4.14293100 | -1.40504700 | 3.85902000 |
| H | -3.29724900 | -1.93273000 | 2.99830100 |
| N | -3.90986800 | -0.04236600 | 3.79801000 |
| C | -5.46729100 | -1.91099300 | 3.28557700 |
| H | -5.69565000 | -1.51645900 | 2.29470800 |
| H | -5.44213200 | -3.00142100 | 3.22629800 |
| H | -6.27957400 | -1.62719500 | 3.96812500 |
| C | -4.33339000 | 0.71295300 | 2.60242000 |
| H | -4.32092300 | 0.03515100 | 1.74312000 |
| H | -3.57644600 | 1.47593700 | 2.40628400 |
| C | -2.93096100 | 0.61367300 | 4.70063300 |
| H | -2.91349700 | 0.02203300 | 5.61469400 |
| C | -1.51144700 | 0.61123100 | 4.10490800 |
| H | -0.81489300 | 1.05199000 | 4.82738000 |
| H | -1.18531100 | -0.41139000 | 3.88846700 |
| H | -1.45883600 | 1.20321300 | 3.18343300 |
| C | -3.75017800 | -2.13066600 | 5.14258300 |
| H | -4.33035300 | -1.74336100 | 5.99046600 |
| H | -3.98382200 | -3.19246300 | 5.03256800 |
| H | -2.68791400 | -2.04019000 | 5.37999200 |
| C | -3.37827800 | 2.02675500 | 5.09762800 |
| H | -4.37597800 | 2.01392400 | 5.54755400 |
| H | -2.67502600 | 2.41448800 | 5.84258900 |
| H | -3.38082500 | 2.72311300 | 4.25300500 |
| C | -5.70812200 | 1.38017700 | 2.72437200 |
| H | -6.50193000 | 0.65016200 | 2.90619600 |
| H | -5.72602900 | 2.11641200 | 3.53271000 |
| H | -5.93283300 | 1.90045000 | 1.78568900 |
| C | -4.58005200 | -2.88351100 | -1.68078600 |
| H | -5.16350900 | -2.09677500 | -1.18491800 |
| H | -3.93634000 | -2.40581200 | -2.42065700 |
| H | -5.28319100 | -3.53277500 | -2.21553800 |
| C | -4.83352900 | -4.28885500 | 0.34903700 |
| H | -5.32295000 | -3.49515400 | 0.92328200 |
| H | -5.61380000 | -4.81115200 | -0.21731800 |

| | | | |
|---|---|---|---|
| H | -4.41261300 | -5.01214200 | 1.05153400 |
| C | -3.80271200 | -3.70760800 | -0.63502600 |
| H | -3.31014100 | -4.54603800 | -1.14526500 |
| H | -2.10827400 | -5.19065500 | 1.01179200 |
| H | -2.81660300 | -4.83838400 | 2.59644600 |
| C | -0.67690400 | -4.65706000 | 2.53775400 |
| C | -0.53905300 | -4.47004000 | 3.92017200 |
| C | 0.45152100 | -5.01126500 | 1.78422600 |
| C | 0.70484000 | -4.62439600 | 4.53814700 |
| H | -1.41244100 | -4.22145000 | 4.51729900 |
| C | 1.69640200 | -5.16800200 | 2.40041400 |
| H | 0.35238400 | -5.18409700 | 0.71560500 |
| C | 1.82608200 | -4.97270100 | 3.77907700 |
| H | 0.79575200 | -4.48425700 | 5.61208800 |
| H | 2.55990400 | -5.45561300 | 1.80619300 |
| H | 2.79211500 | -5.09989100 | 4.25987400 |
| F | -1.74408700 | -1.75954400 | -6.28607600 |

Energies (0K) = -4290.1291412

Energies (0K) + ZPE = -4289.435606

Enthalpies (298K) = -4289.373066

Free Energies (298K) = -4289.538757

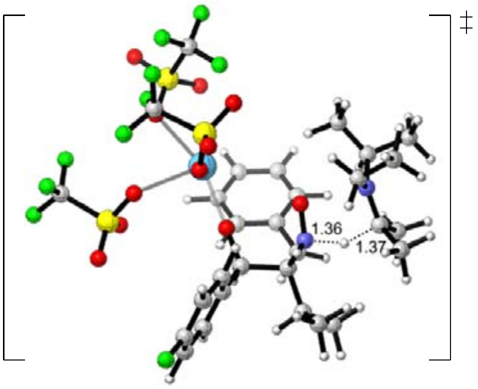


**$TS_{C'}$**

Number of imaginary frequencies = 1

0 1

| | | | |
|---|---|---|---|
| C | -2.26877100 | -3.95442000 | 2.18041200 |
| C | -2.83782600 | -2.44864300 | 0.11592100 |
| N | -2.63459300 | -2.55097800 | 1.63415900 |
| O | -1.97041400 | -1.57758900 | 2.13726100 |
| O | 5.78925900 | -0.53923200 | 3.38782200 |
| S | 4.32556900 | -0.54451000 | 3.23707900 |
| O | 3.86385200 | -0.85742800 | 1.84066300 |
| O | 3.54645000 | -1.21909200 | 4.29342000 |
| C | 3.83746800 | 1.23882600 | 3.41726400 |

| | | | |
|---|---|---|---|
| F | 4.15506400 | 1.70122900 | 4.63245700 |
| F | 4.43912700 | 2.00558000 | 2.49749400 |
| F | 2.49994200 | 1.37137900 | 3.25673600 |
| O | 0.04030600 | 3.36289000 | -1.86821100 |
| S | 0.71498900 | 2.58444200 | -0.82087400 |
| O | 0.53664600 | 3.02830400 | 0.57618700 |
| O | 0.57494100 | 1.08927300 | -0.99591700 |
| C | 2.52994100 | 2.81492700 | -1.13577500 |
| F | 2.85627600 | 2.42697200 | -2.37466300 |
| F | 3.23781300 | 2.07075800 | -0.26045600 |
| F | 2.88181500 | 4.09637300 | -0.97983700 |
| O | 2.63744600 | -1.26384700 | -1.34093000 |
| S | 3.14672600 | -2.57128000 | -1.90564600 |
| O | 3.16549800 | -3.66266300 | -0.91381400 |
| O | 2.61024200 | -2.88281200 | -3.23852800 |
| C | 4.93442700 | -2.16168100 | -2.20453000 |
| F | 5.04290100 | -1.12176200 | -3.04252000 |
| F | 5.55769500 | -3.21693900 | -2.74821300 |
| F | 5.54988100 | -1.84912000 | -1.05615800 |
| O | -0.43273300 | -1.92697900 | -0.12182600 |
| C | -1.45446600 | -2.70836000 | -0.61157200 |
| C | -1.60439000 | -2.49263400 | -2.12351300 |
| C | -1.57987400 | -3.57451900 | -3.01230900 |
| C | -1.70455700 | -1.19476700 | -2.64876600 |
| C | -1.67433500 | -3.38155200 | -4.39409900 |
| H | -1.47957600 | -4.58589700 | -2.62625100 |
| C | -1.80165800 | -0.97999500 | -4.02384600 |
| H | -1.68812800 | -0.34144000 | -1.97829600 |
| C | -1.78839200 | -2.08348700 | -4.87153700 |
| H | -1.65227000 | -4.21619400 | -5.08786100 |
| H | -1.88102200 | 0.01994900 | -4.43925200 |
| La | 1.27534600 | -0.75139400 | 0.75613700 |
| H | -3.07780900 | -1.38956400 | 0.00327400 |
| H | -1.23288500 | -3.78010800 | -0.46814700 |
| C | -4.62226700 | -1.56202600 | 3.04311300 |
| H | -3.86351700 | -2.29254200 | 2.16667200 |
| N | -3.72207700 | -0.56202400 | 3.33063000 |
| C | -5.86448100 | -1.20243600 | 2.22514000 |
| H | -5.65967600 | -0.66065100 | 1.30176500 |
| H | -6.40800500 | -2.11415800 | 1.97188200 |
| H | -6.52701300 | -0.58590100 | 2.84510000 |
| C | -3.66051000 | 0.64376600 | 2.46726200 |
| H | -3.93851100 | 0.33959700 | 1.45981900 |
| H | -2.61736300 | 0.95243400 | 2.41713200 |

| | | | |
|---|---|---|---|
| C | -2.99156800 | -0.51499900 | 4.64316200 |
| H | -2.73225600 | -1.54737700 | 4.87017100 |
| C | -1.68145700 | 0.27295400 | 4.58098000 |
| H | -1.15883200 | 0.12488100 | 5.53245100 |
| H | -1.03204700 | -0.08457200 | 3.77864200 |
| H | -1.84277300 | 1.34943400 | 4.46299500 |
| C | -4.94292800 | -2.61565100 | 4.09963300 |
| H | -5.68058600 | -2.22409200 | 4.81013200 |
| H | -5.39809000 | -3.47671700 | 3.60292400 |
| H | -4.07709900 | -2.96779600 | 4.66211100 |
| C | -3.89036600 | 0.02456100 | 5.77122000 |
| H | -4.85174900 | -0.49168700 | 5.82752500 |
| H | -3.37458800 | -0.12481100 | 6.72677100 |
| H | -4.08122500 | 1.09532900 | 5.65717700 |
| C | -4.53221200 | 1.81876700 | 2.92282500 |
| H | -5.57468700 | 1.52719600 | 3.08009000 |
| H | -4.15642000 | 2.27661300 | 3.84172200 |
| H | -4.50943400 | 2.58326400 | 2.13709100 |
| C | -4.92436500 | -2.43254600 | -1.32855100 |
| H | -5.52393500 | -1.78643800 | -0.67699400 |
| H | -4.37449400 | -1.79041800 | -2.02353600 |
| H | -5.61516400 | -3.04870300 | -1.91613900 |
| C | -4.81711200 | -4.25051900 | 0.40154600 |
| H | -5.32465800 | -3.72000400 | 1.20970400 |
| H | -5.59681100 | -4.71150000 | -0.21616900 |
| H | -4.23802600 | -5.06776800 | 0.83705700 |
| C | -3.97886400 | -3.32995700 | -0.50746100 |
| H | -3.48474600 | -4.00461800 | -1.21461000 |
| H | -2.24288800 | -4.61659700 | 1.31706000 |
| H | -3.09348000 | -4.25964100 | 2.82310500 |
| C | -0.96171200 | -4.01783500 | 2.92630000 |
| C | -0.92824500 | -3.86479600 | 4.31920400 |
| C | 0.22933300 | -4.32979000 | 2.25273500 |
| C | 0.27068700 | -3.99759500 | 5.02455300 |
| H | -1.84977700 | -3.65872000 | 4.85683700 |
| C | 1.42803900 | -4.47316000 | 2.95596300 |
| H | 0.21419500 | -4.48150200 | 1.17719200 |
| C | 1.45270500 | -4.30164400 | 4.34396000 |
| H | 0.27798400 | -3.87871300 | 6.10475500 |
| H | 2.33884800 | -4.73289000 | 2.42239800 |
| H | 2.38475800 | -4.41520500 | 4.89042600 |
| F | -1.88144800 | -1.88167100 | -6.21024900 |

Energies (0K) = -4290.1169505

Energies (0K) + ZPE = -4289.420000

Enthalpies (298K) = -4289.358138

Free Energies (298K) = -4289.522328

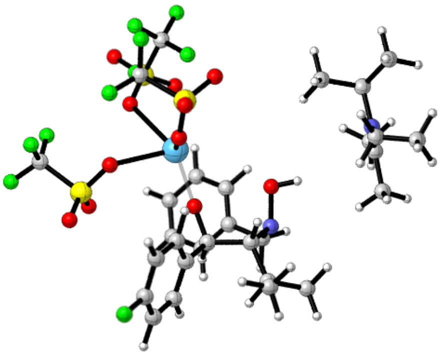

**D**

Number of imaginary frequencies = 0

0 1

| | | | |
|---|---|---|---|
| C | -2.52706300 | -3.60185800 | 1.03768500 |
| C | -3.04796700 | -2.00738400 | -0.91019500 |
| N | -3.07476100 | -2.33385400 | 0.53398000 |
| O | -2.55583900 | -1.24285100 | 1.31966700 |
| O | 5.28580900 | 0.01542400 | 3.03770700 |
| S | 3.84159300 | -0.00157800 | 2.75431300 |
| O | 3.50472700 | -0.22818800 | 1.30925300 |
| O | 2.98890500 | -0.75650300 | 3.69723100 |
| C | 3.29821100 | 1.75760300 | 3.00448200 |
| F | 3.44080500 | 2.12542900 | 4.28528000 |
| F | 4.00636600 | 2.59887700 | 2.24036000 |
| F | 1.99241900 | 1.89096300 | 2.67648100 |
| O | -0.57705900 | 3.98995800 | -2.00255700 |
| S | 0.01155500 | 3.16535700 | -0.93744600 |
| O | -0.36617000 | 3.48749600 | 0.45236600 |
| O | -0.01336000 | 1.68711900 | -1.23883500 |
| C | 1.83270800 | 3.53276700 | -1.00660600 |
| F | 2.31590500 | 3.32675100 | -2.23864600 |
| F | 2.50437000 | 2.73209000 | -0.15257300 |
| F | 2.06935400 | 4.80466200 | -0.66120300 |
| O | 2.38502600 | -0.33238300 | -1.86834700 |
| S | 3.07938500 | -1.42184500 | -2.65125100 |
| O | 3.17391400 | -2.69733000 | -1.91772100 |
| O | 2.66575400 | -1.48632900 | -4.06097500 |
| C | 4.81866100 | -0.77206100 | -2.70927100 |
| F | 4.84703800 | 0.43644700 | -3.28881300 |
| F | 5.59268800 | -1.60509000 | -3.42093200 |
| F | 5.33249100 | -0.66425500 | -1.47680000 |
| O | -0.68308800 | -1.44383000 | -0.96663700 |

| | | | |
|---|---|---|---|
| C | -1.64701300 | -2.22768600 | -1.58874400 |
| C | -1.63102000 | -1.98198900 | -3.09835300 |
| C | -1.73505000 | -3.04527600 | -4.00590200 |
| C | -1.48027400 | -0.68264300 | -3.60933500 |
| C | -1.72228700 | -2.82862300 | -5.38713400 |
| H | -1.82258500 | -4.06317800 | -3.63358100 |
| C | -1.46530600 | -0.44367600 | -4.98445400 |
| H | -1.35888100 | 0.14818600 | -2.92106000 |
| C | -1.59281000 | -1.52586000 | -5.84904200 |
| H | -1.79908400 | -3.64972000 | -6.09340300 |
| H | -1.35041000 | 0.55856300 | -5.38661800 |
| La | 0.88244600 | -0.35926200 | 0.19815500 |
| H | -3.24882700 | -0.93058300 | -0.93027500 |
| H | -1.41664000 | -3.29674400 | -1.45147100 |
| C | -5.32943400 | -0.03096000 | 3.88689500 |
| H | -3.34204000 | -0.94233200 | 1.81014600 |
| N | -5.19500400 | 1.24358600 | 3.65378700 |
| C | -6.19118800 | -0.92552000 | 3.05612900 |
| H | -6.74002600 | -0.44445200 | 2.25061600 |
| H | -5.55097300 | -1.71433200 | 2.63946200 |
| H | -6.90133200 | -1.42847400 | 3.72438800 |
| C | -5.88621500 | 1.91958900 | 2.51439000 |
| H | -5.79974400 | 1.27669200 | 1.63794000 |
| H | -5.31763100 | 2.82368600 | 2.30094800 |
| C | -4.27068900 | 2.10552000 | 4.47414400 |
| H | -4.02051900 | 1.52349600 | 5.35794200 |
| C | -2.98362100 | 2.37084700 | 3.68525900 |
| H | -2.28953800 | 2.92651800 | 4.32446600 |
| H | -2.50118100 | 1.43240000 | 3.38986800 |
| H | -3.16498200 | 2.97004700 | 2.78659200 |
| C | -4.59792200 | -0.71346600 | 5.00822300 |
| H | -4.92525700 | -0.32490900 | 5.97957600 |
| H | -4.80370200 | -1.78453300 | 4.98110900 |
| H | -3.51627500 | -0.56213000 | 4.93145900 |
| C | -4.95826800 | 3.38677600 | 4.95027300 |
| H | -5.86856000 | 3.16391600 | 5.51530000 |
| H | -4.26437300 | 3.90685800 | 5.61895200 |
| H | -5.20347000 | 4.06933600 | 4.13103700 |
| C | -7.34448600 | 2.26834200 | 2.80972800 |
| H | -7.94375900 | 1.37783800 | 3.02172700 |
| H | -7.43246000 | 2.95342500 | 3.65696200 |
| H | -7.76627300 | 2.76071400 | 1.92667100 |
| C | -4.84352100 | -1.81109400 | -2.72676900 |
| H | -5.35037700 | -0.95919000 | -2.25303000 |

| | | | |
|---|---|---|---|
| H | -4.10215100 | -1.41034600 | -3.42111400 |
| H | -5.59320200 | -2.35530400 | -3.31488600 |
| C | -5.37132900 | -3.20057700 | -0.73487900 |
| H | -5.81207300 | -2.36237800 | -0.18171100 |
| H | -6.16534000 | -3.64610100 | -1.34831800 |
| H | -5.05807400 | -3.95326800 | -0.00647600 |
| C | -4.22950000 | -2.72898100 | -1.65229000 |
| H | -3.82518300 | -3.61974200 | -2.15347300 |
| H | -2.91630500 | -4.38024800 | 0.37374600 |
| H | -2.99750700 | -3.76024700 | 2.01786500 |
| C | -1.02394200 | -3.80761500 | 1.20270500 |
| C | -0.28987500 | -3.10270400 | 2.17156800 |
| C | -0.35798800 | -4.78801900 | 0.45331700 |
| C | 1.07906400 | -3.33083800 | 2.34377000 |
| H | -0.79625100 | -2.36555400 | 2.78725800 |
| C | 1.00694200 | -5.03464100 | 0.63273000 |
| H | -0.91423900 | -5.37055300 | -0.27779500 |
| C | 1.73314200 | -4.29876900 | 1.57242900 |
| H | 1.63425000 | -2.76168500 | 3.08466600 |
| H | 1.49884700 | -5.80411600 | 0.04292100 |
| H | 2.79401200 | -4.48758000 | 1.71648800 |
| F | -1.57614700 | -1.30166100 | -7.18914600 |

Energies (0K) = -4290.1767186

Energies (0K) + ZPE = -4289.477017

Enthalpies (298K) = -4289.414022

Free Energies (298K) = -4289.58207

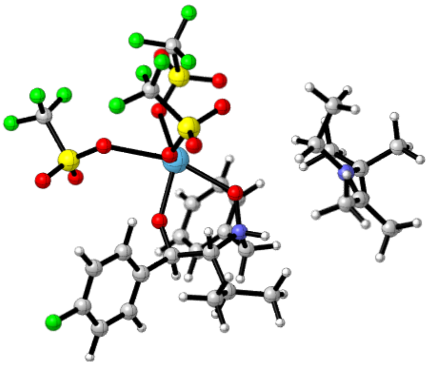

**D′**

Number of imaginary frequencies = 0

0 1

| | | | |
|---|---|---|---|
| C | -1.71590100 | -4.25331900 | 1.64908000 |
| C | -2.43137500 | -2.77165200 | -0.37197700 |
| N | -2.12970800 | -2.84109700 | 1.14336800 |
| O | -1.30032500 | -1.82643600 | 1.57716500 |
| O | 5.09531200 | -0.30172400 | 3.40317800 |

| | | | |
|---|---|---|---|
| S | 3.64379000 | -0.30422500 | 3.16042500 |
| O | 3.26486400 | -0.66257500 | 1.75072100 |
| O | 2.79560900 | -0.92676800 | 4.19662100 |
| C | 3.15216900 | 1.48482000 | 3.23296100 |
| F | 3.42204200 | 2.01094800 | 4.43394400 |
| F | 3.78796200 | 2.20562800 | 2.29865300 |
| F | 1.82115600 | 1.60269700 | 3.01400000 |
| O | -1.62948400 | 3.25267200 | -1.39057000 |
| S | -0.64437400 | 2.59819000 | -0.51625600 |
| O | -0.76496700 | 2.84754600 | 0.93447200 |
| O | -0.40341500 | 1.15090500 | -0.86022800 |
| C | 0.98073300 | 3.36552900 | -0.98192800 |
| F | 1.22372300 | 3.20606300 | -2.29004900 |
| F | 1.98224800 | 2.78672100 | -0.29500000 |
| F | 0.98033600 | 4.67641200 | -0.70431000 |
| O | 2.43840300 | -0.33860500 | -1.20107100 |
| S | 3.24099700 | -1.12501500 | -2.20249600 |
| O | 3.54892500 | -2.50417700 | -1.78005800 |
| O | 2.80256300 | -0.93682400 | -3.59573500 |
| C | 4.86939500 | -0.23518000 | -2.11329700 |
| F | 4.69870100 | 1.07886100 | -2.32873700 |
| F | 5.71085300 | -0.71083700 | -3.04516200 |
| F | 5.44154700 | -0.39514800 | -0.91121400 |
| O | -0.22242900 | -1.99883200 | -1.01827300 |
| C | -1.10098900 | -3.05865900 | -1.15918600 |
| C | -1.31068600 | -3.39655600 | -2.63962100 |
| C | -1.52497200 | -4.71833400 | -3.05448300 |
| C | -1.22456200 | -2.39394900 | -3.61506700 |
| C | -1.69016300 | -5.03687100 | -4.40504100 |
| H | -1.55449300 | -5.51805300 | -2.31823600 |
| C | -1.38609900 | -2.68978500 | -4.96979000 |
| H | -1.02199100 | -1.37470700 | -3.30327100 |
| C | -1.62230300 | -4.00975500 | -5.33780800 |
| H | -1.85315600 | -6.05895800 | -4.73312900 |
| H | -1.32359300 | -1.91943000 | -5.73250600 |
| La | 0.71694700 | -0.73609000 | 0.64893800 |
| H | -2.67040500 | -1.71167500 | -0.50356700 |
| H | -0.68417000 | -3.98372700 | -0.72564700 |
| C | -4.50104300 | -0.71026400 | 3.74062500 |
| H | -3.03064000 | -2.66000400 | 1.60045200 |
| N | -3.34952700 | -0.21729800 | 4.10697500 |
| C | -5.28055700 | -0.13368300 | 2.59308700 |
| H | -4.67302300 | -0.00258900 | 1.69357000 |
| H | -6.12277200 | -0.78620200 | 2.35718500 |

| | | | |
|---|---|---|---|
| H | -5.67727900 | 0.85077300 | 2.87142100 |
| C | -2.77444400 | 0.96990200 | 3.41090100 |
| H | -3.10356100 | 0.94313600 | 2.37519300 |
| H | -1.69384900 | 0.84737800 | 3.40254900 |
| C | -2.58207900 | -0.79752900 | 5.28177700 |
| H | -2.78919900 | -1.86678800 | 5.25433200 |
| C | -1.07009000 | -0.63792400 | 5.13323500 |
| H | -0.59920600 | -1.23980300 | 5.91721600 |
| H | -0.72436900 | -1.01524400 | 4.16586500 |
| H | -0.73650000 | 0.39628700 | 5.26604400 |
| C | -5.16813600 | -1.86155600 | 4.42357100 |
| H | -6.18281800 | -1.54408900 | 4.69537700 |
| H | -5.28670200 | -2.66723200 | 3.68724400 |
| H | -4.67475700 | -2.25003600 | 5.31005300 |
| C | -3.10139200 | -0.21202600 | 6.59851100 |
| H | -4.17514400 | -0.38389800 | 6.72267600 |
| H | -2.57891300 | -0.69897600 | 7.42894600 |
| H | -2.90994000 | 0.86307000 | 6.66163300 |
| C | -3.16895600 | 2.29671400 | 4.06010100 |
| H | -4.25633400 | 2.40405900 | 4.12476700 |
| H | -2.74443500 | 2.40540900 | 5.06162700 |
| H | -2.78133200 | 3.10963900 | 3.43668800 |
| C | -4.39869500 | -3.03177600 | -1.95290900 |
| H | -4.83814800 | -2.05373400 | -1.71755700 |
| H | -3.72803000 | -2.90014500 | -2.80206900 |
| H | -5.21262500 | -3.69708300 | -2.26329900 |
| C | -4.75361300 | -3.63407400 | 0.42242500 |
| H | -5.04435300 | -2.61605900 | 0.71663500 |
| H | -5.65711500 | -4.13436900 | 0.05662800 |
| H | -4.43636700 | -4.18432200 | 1.31480900 |
| C | -3.70045600 | -3.60894600 | -0.70529800 |
| H | -3.39913200 | -4.64376600 | -0.91137400 |
| H | -1.64113200 | -4.89756100 | 0.77456700 |
| H | -2.55800400 | -4.59360100 | 2.25284100 |
| C | -0.45128000 | -4.30787900 | 2.46299400 |
| C | -0.53045100 | -4.41693600 | 3.85830300 |
| C | 0.81226700 | -4.36143200 | 1.85294900 |
| C | 0.62444900 | -4.56700000 | 4.63100700 |
| H | -1.50499300 | -4.40331800 | 4.34091100 |
| C | 1.96786400 | -4.51204600 | 2.62255900 |
| H | 0.89508200 | -4.30132700 | 0.77095500 |
| C | 1.87684900 | -4.61557700 | 4.01431600 |
| H | 0.54380300 | -4.65723800 | 5.71099500 |
| H | 2.93755000 | -4.56196600 | 2.13419900 |

| | | | |
|---|---|---|---|
| H | 2.77608000 | -4.74042400 | 4.61138400 |
| F | -1.77623100 | -4.30705900 | -6.65401800 |

Energies (0K) = -4290.1832383

Energies (0K) + ZPE = -4289.481635

Enthalpies (298K) = -4289.419209

Free Energies (298K) = -4289.588726

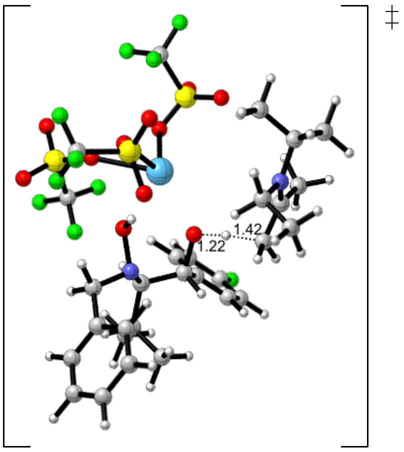


**$TS_D$**

Number of imaginary frequencies = 1

0 1

| | | | |
|---|---|---|---|
| C | -1.65673800 | -4.42229800 | 0.14840600 |
| C | -2.72517100 | -2.85855300 | -1.54200200 |
| N | -1.51118000 | -3.26409100 | -0.77166500 |
| O | -1.23618400 | -2.14094500 | 0.13723500 |
| O | -4.22596600 | 1.21508800 | 3.62508900 |
| S | -3.81955700 | 0.01300300 | 2.88397100 |
| O | -2.64204100 | 0.25003100 | 1.96556600 |
| O | -3.71439900 | -1.24625700 | 3.63511600 |
| C | -5.20086700 | -0.28486300 | 1.67260900 |
| F | -6.36099700 | -0.43551700 | 2.31980500 |
| F | -5.31669400 | 0.74284400 | 0.81623400 |
| F | -4.96551900 | -1.39848500 | 0.95434700 |
| O | -1.41416100 | 4.26145800 | -1.11234800 |
| S | -2.54162900 | 4.18186600 | -0.16049700 |
| O | -3.65803100 | 5.11559400 | -0.35929800 |
| O | -2.98124500 | 2.76503700 | 0.12939500 |
| C | -1.79324200 | 4.66242100 | 1.46940300 |
| F | -0.79338400 | 3.81481600 | 1.78282200 |
| F | -2.70645700 | 4.61363800 | 2.44627300 |
| F | -1.29132400 | 5.90186000 | 1.40917600 |
| O | -1.36770600 | -0.89346700 | -2.23630400 |
| C | -2.29339400 | -1.88892200 | -2.68471800 |
| C | -3.45952100 | -1.19412400 | -3.38488500 |

| | | | |
|---|---|---|---|
| C | -3.54816300 | -1.21299100 | -4.78375700 |
| C | -4.41852600 | -0.45799700 | -2.66867700 |
| C | -4.55887400 | -0.52307400 | -5.45954500 |
| H | -2.81768600 | -1.77482600 | -5.35982600 |
| C | -5.43421300 | 0.24032200 | -3.32245700 |
| H | -4.39948300 | -0.44256700 | -1.58257100 |
| C | -5.48305700 | 0.19415800 | -4.71187800 |
| H | -4.63026000 | -0.53702300 | -6.54259600 |
| H | -6.18208500 | 0.80266200 | -2.77195400 |
| La | -1.49590700 | 0.57531800 | -0.22749600 |
| H | -3.39323900 | -2.33798100 | -0.83940000 |
| H | -1.76134500 | -2.49298700 | -3.42833900 |
| C | 1.14919800 | 0.54272000 | -4.00793200 |
| N | 2.33695600 | 0.59597900 | -3.39029300 |
| C | 0.40314100 | -0.65219400 | -4.17365900 |
| H | -0.52587700 | -0.71784000 | -3.09893100 |
| H | -0.30729800 | -0.60809000 | -4.99947800 |
| H | 0.92199600 | -1.60589000 | -4.11477100 |
| C | 2.98449300 | -0.64742600 | -2.91556500 |
| H | 2.22277700 | -1.28050800 | -2.45382200 |
| H | 3.68025200 | -0.37244600 | -2.12146100 |
| C | 3.02671900 | 1.88169100 | -3.07801600 |
| H | 2.51014500 | 2.65508000 | -3.64264200 |
| C | 2.89816200 | 2.22334200 | -1.58766600 |
| H | 3.34799100 | 3.20408600 | -1.39658200 |
| H | 1.84489200 | 2.27092000 | -1.28645700 |
| H | 3.40739700 | 1.49270400 | -0.94996800 |
| C | 0.50967600 | 1.82282200 | -4.49364900 |
| H | 1.12642800 | 2.30349800 | -5.26202300 |
| H | -0.46579000 | 1.60199100 | -4.93094900 |
| H | 0.36597600 | 2.54704700 | -3.68432800 |
| C | 4.48529000 | 1.88207400 | -3.54990400 |
| H | 4.55550800 | 1.65516700 | -4.61878600 |
| H | 4.90537300 | 2.88070400 | -3.38735500 |
| H | 5.10536500 | 1.16913400 | -2.99659500 |
| C | 3.72822100 | -1.41391900 | -4.01277500 |
| H | 3.06862100 | -1.67329000 | -4.84651500 |
| H | 4.56603200 | -0.83159600 | -4.40714500 |
| H | 4.12777500 | -2.34490400 | -3.59407500 |
| O | 1.47569100 | -1.98095900 | 0.53066000 |
| S | 1.52907700 | -0.94231700 | 1.59794600 |
| O | 2.77177100 | -0.16791900 | 1.69305300 |
| O | 0.26054500 | -0.13325000 | 1.64391500 |
| C | 1.42235000 | -1.90644100 | 3.18256200 |

| | | | |
|---|---|---|---|
| F | 0.31412000 | -2.65938500 | 3.19361800 |
| F | 2.48876200 | -2.70621900 | 3.29519300 |
| F | 1.39059400 | -1.07408500 | 4.22827700 |
| H | -0.25255700 | -2.18412900 | 0.21784800 |
| C | -2.93570400 | -4.75075000 | -3.34010900 |
| H | -3.10257600 | -4.14048100 | -4.23480200 |
| H | -1.86035800 | -4.93474700 | -3.24928500 |
| H | -3.42391300 | -5.71768400 | -3.51111800 |
| C | -5.02381900 | -3.79954700 | -2.27810100 |
| H | -5.21896500 | -3.15335300 | -3.13805900 |
| H | -5.55578800 | -4.74392300 | -2.44750200 |
| H | -5.46120900 | -3.32712500 | -1.39020300 |
| H | -2.66462300 | -4.49236700 | 0.58197700 |
| H | -0.97813600 | -4.20400700 | 0.97775900 |
| C | -1.23090100 | -5.74514900 | -0.46382400 |
| C | -0.01157300 | -5.85489800 | -1.14974000 |
| C | -2.00580300 | -6.89773800 | -0.28167900 |
| C | 0.41666700 | -7.08674300 | -1.64808300 |
| H | 0.60128700 | -4.96904800 | -1.29252200 |
| C | -1.57699700 | -8.13391700 | -0.77494000 |
| H | -2.95154800 | -6.82719400 | 0.25105600 |
| C | -0.36503500 | -8.23172500 | -1.46199700 |
| H | 1.36322500 | -7.15433900 | -2.17853200 |
| H | -2.19193100 | -9.01744500 | -0.62368800 |
| H | -0.03091900 | -9.19080100 | -1.84912600 |
| C | -3.52427600 | -4.09262600 | -2.08105800 |
| H | -3.49284700 | -4.83964900 | -1.28637900 |
| F | -6.46708700 | 0.86929800 | -5.35425100 |

Energies (0K) = -4290.1576008

Energies (0K) + ZPE = -4289.461435

Enthalpies (298K) = -4289.399728

Free Energies (298K) = -4289.564949

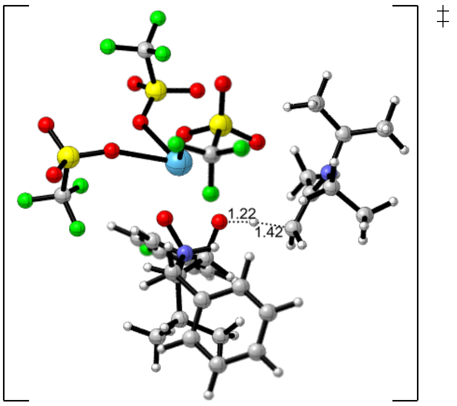


**TS$_{D'}$**

Number of imaginary frequencies = 1

0 1

| | | | |
|---|---|---|---|
| C | -1.81721500 | -4.21662200 | 0.20315200 |
| C | -3.02536200 | -2.85221500 | -1.69833300 |
| N | -1.91069300 | -2.95278500 | -0.64046300 |
| O | -2.07398500 | -1.90464600 | 0.26469400 |
| O | -4.57035800 | 1.30533800 | 3.68959900 |
| S | -4.10871400 | 0.07770700 | 3.02298800 |
| O | -2.87592100 | 0.28186900 | 2.18098200 |
| O | -4.06422700 | -1.14986900 | 3.83334500 |
| C | -5.41194600 | -0.26621300 | 1.73861600 |
| F | -6.62832800 | -0.26501500 | 2.29818700 |
| F | -5.38693800 | 0.67360400 | 0.77464400 |
| F | -5.21506100 | -1.45918600 | 1.15598300 |
| O | -1.45719400 | 4.12671000 | -0.96625200 |
| S | -2.57615200 | 4.08472600 | -0.00271100 |
| O | -3.67112100 | 5.04375700 | -0.20619700 |
| O | -3.04532900 | 2.68637900 | 0.31396600 |
| C | -1.79990000 | 4.57785200 | 1.60975400 |
| F | -0.80450200 | 3.72425600 | 1.91987100 |
| F | -2.69881600 | 4.55122500 | 2.60142000 |
| F | -1.28604200 | 5.81173300 | 1.52758300 |
| O | -1.55168600 | -0.92056100 | -2.22240800 |
| C | -2.58209000 | -1.76256100 | -2.73536300 |
| C | -3.74235400 | -0.93024900 | -3.26982700 |
| C | -3.85281800 | -0.71638400 | -4.65195100 |
| C | -4.66820800 | -0.30335800 | -2.42204900 |
| C | -4.85395300 | 0.09925300 | -5.18347000 |
| H | -3.14799500 | -1.19525700 | -5.32701800 |
| C | -5.67481000 | 0.51859100 | -2.93193400 |
| H | -4.62383300 | -0.45669000 | -1.34867700 |
| C | -5.74698200 | 0.70408400 | -4.30794700 |
| H | -4.94499500 | 0.26497000 | -6.25234100 |
| H | -6.39608000 | 1.00115800 | -2.27977100 |
| La | -1.62699500 | 0.46132700 | -0.00509400 |

| | | | |
|---|---|---|---|
| H | -3.85567900 | -2.49818600 | -1.08700800 |
| H | -2.14730700 | -2.29148000 | -3.59005200 |
| C | 1.12769200 | 0.16676900 | -4.08221100 |
| N | 2.31495300 | 0.18264100 | -3.46427900 |
| C | 0.27444800 | -0.96760500 | -4.11574900 |
| H | -0.66810200 | -0.85393800 | -3.05961600 |
| H | -0.42235400 | -0.95151800 | -4.95467900 |
| H | 0.71251000 | -1.95060100 | -3.95801800 |
| C | 2.81912800 | -1.03417700 | -2.78928400 |
| H | 1.98193600 | -1.51290300 | -2.27649400 |
| H | 3.50637200 | -0.71101300 | -2.00732000 |
| C | 3.12697200 | 1.42774400 | -3.31880700 |
| H | 2.74019100 | 2.13414200 | -4.05096600 |
| C | 2.94595100 | 2.03901200 | -1.92291200 |
| H | 3.51167700 | 2.97536800 | -1.85853000 |
| H | 1.89115000 | 2.26652200 | -1.72857800 |
| H | 3.30468800 | 1.37035100 | -1.13361100 |
| C | 0.60700100 | 1.43680200 | -4.71415500 |
| H | 1.23957500 | 1.74907500 | -5.55364400 |
| H | -0.40163300 | 1.26958700 | -5.09648800 |
| H | 0.57128200 | 2.26628600 | -3.99980600 |
| C | 4.60289500 | 1.19645900 | -3.66118000 |
| H | 4.71821000 | 0.78564100 | -4.66942300 |
| H | 5.12134700 | 2.16083900 | -3.62641800 |
| H | 5.09995500 | 0.52943600 | -2.94934100 |
| C | 3.51773500 | -2.02313600 | -3.72571800 |
| H | 2.86021300 | -2.35388700 | -4.53587000 |
| H | 4.41622800 | -1.58722900 | -4.17194500 |
| H | 3.81931400 | -2.90773600 | -3.15244700 |
| O | 2.04269300 | -0.60266300 | 0.47267000 |
| S | 1.54016400 | -0.45046300 | 1.85349300 |
| O | 2.33085800 | 0.39306600 | 2.76310800 |
| O | 0.05680400 | -0.19703800 | 1.91846300 |
| C | 1.65229600 | -2.15604800 | 2.58137100 |
| F | 1.01065800 | -3.04561800 | 1.80332600 |
| F | 2.93451200 | -2.53329800 | 2.68043700 |
| F | 1.10104900 | -2.18629400 | 3.80094100 |
| C | -2.50190300 | -4.75286100 | -3.42251600 |
| H | -2.59867500 | -4.21444800 | -4.37203300 |
| H | -1.45100700 | -4.71588700 | -3.11789700 |
| H | -2.75198300 | -5.80146400 | -3.61977500 |
| C | -4.88186700 | -4.08059000 | -2.91597600 |
| H | -4.93448200 | -3.39614200 | -3.76889200 |
| H | -5.21562500 | -5.06618900 | -3.26118500 |

| | | | |
|---|---|---|---|
| H | -5.59110000 | -3.73323200 | -2.15563200 |
| H | -2.83663600 | -4.49328700 | 0.47337900 |
| H | -1.31196900 | -3.85368000 | 1.09852900 |
| C | -1.04437000 | -5.36853200 | -0.39592400 |
| C | 0.30743100 | -5.21868900 | -0.74461000 |
| C | -1.63765700 | -6.63210400 | -0.51743100 |
| C | 1.03867800 | -6.30229300 | -1.23431900 |
| H | 0.79677600 | -4.25527800 | -0.61794900 |
| C | -0.90284600 | -7.72115400 | -0.99262800 |
| H | -2.67802200 | -6.76551500 | -0.23183700 |
| C | 0.43475200 | -7.55714800 | -1.35961800 |
| H | 2.08302400 | -6.16993300 | -1.50392700 |
| H | -1.37716600 | -8.69525600 | -1.07648200 |
| H | 1.00641300 | -8.40253300 | -1.73295300 |
| C | -3.45330400 | -4.19349600 | -2.35033800 |
| H | -3.51507700 | -4.92463100 | -1.53945100 |
| F | -6.72351300 | 1.49651400 | -4.81159600 |
| H | -1.01815400 | -2.81322500 | -1.13299400 |

Energies (0K) = -4290.1666532

Energies (0K) + ZPE = -4289.468966

Enthalpies (298K) = -4289.407175

Free Energies (298K) = -4289.572524

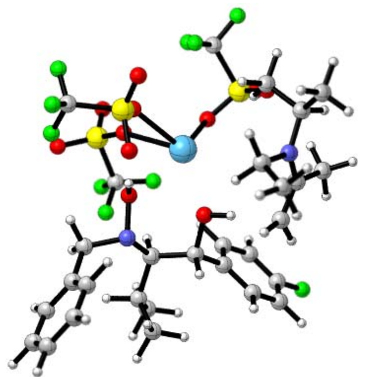

**E**

Number of imaginary frequencies = 0

0 1

| | | | |
|---|---|---|---|
| C | -0.30094700 | -4.12538800 | 0.68627100 |
| C | -1.19065700 | -2.55393700 | -1.11575400 |
| N | -0.04385700 | -3.03045900 | -0.28330300 |
| O | 0.31266200 | -1.88904700 | 0.56988100 |
| O | -2.73854000 | 1.48040400 | 3.99447500 |
| S | -2.26645800 | 0.25301300 | 3.34046000 |
| O | -1.05029300 | 0.47138600 | 2.46630000 |
| O | -2.16852000 | -0.96154600 | 4.16132900 |
| C | -3.56195000 | -0.13954600 | 2.06267800 |

| | | | |
|---|---|---|---|
| F | -4.78493900 | -0.08311500 | 2.59503200 |
| F | -3.49216900 | 0.74260000 | 1.04723500 |
| F | -3.36946000 | -1.36814600 | 1.55528100 |
| O | 0.13099900 | 4.50730400 | -0.59888200 |
| S | -0.99921400 | 4.41244400 | 0.34773000 |
| O | -2.12576900 | 5.33353100 | 0.15024200 |
| O | -1.42619400 | 2.98792000 | 0.62622800 |
| C | -0.26209200 | 4.88974100 | 1.98402700 |
| F | 0.73273800 | 4.03886800 | 2.30439600 |
| F | -1.18292600 | 4.84395700 | 2.95324100 |
| F | 0.24385800 | 6.12742600 | 1.92684500 |
| O | 0.39252000 | -0.83378200 | -1.85977600 |
| C | -0.62222000 | -1.76291400 | -2.32605400 |
| C | -1.65617400 | -0.99783000 | -3.13985200 |
| C | -1.71636900 | -1.16762300 | -4.52992700 |
| C | -2.51889200 | -0.06411500 | -2.54235800 |
| C | -2.61417600 | -0.43599800 | -5.31183300 |
| H | -1.05455700 | -1.88160600 | -5.01264500 |
| C | -3.42012500 | 0.67880600 | -3.30451100 |
| H | -2.51743200 | 0.07568700 | -1.46494800 |
| C | -3.44883500 | 0.47655600 | -4.68067000 |
| H | -2.66630700 | -0.56553800 | -6.38809100 |
| H | -4.09546900 | 1.39525600 | -2.84739200 |
| La | 0.06701900 | 0.81284000 | 0.26969700 |
| H | -1.79063200 | -1.88363600 | -0.48257200 |
| H | -0.11113800 | -2.47515200 | -2.97912200 |
| C | 2.62737600 | 1.16782600 | -3.89581000 |
| N | 3.85688400 | 1.27275300 | -3.28971100 |
| C | 2.03447900 | -0.02517500 | -4.23121900 |
| H | 0.89730700 | -0.48667100 | -2.64929100 |
| H | 1.13143600 | -0.00061400 | -4.83232200 |
| H | 2.53939700 | -0.98159400 | -4.15993900 |
| C | 4.59103000 | 0.04850000 | -2.94517900 |
| H | 3.88666800 | -0.68658700 | -2.53575700 |
| H | 5.28611200 | 0.28687600 | -2.13726900 |
| C | 4.38144900 | 2.55390000 | -2.76230300 |
| H | 3.80015700 | 3.34634100 | -3.23141900 |
| C | 4.18545700 | 2.67062400 | -1.24143700 |
| H | 4.51600200 | 3.65548200 | -0.89084800 |
| H | 3.12737900 | 2.55685500 | -0.97460000 |
| H | 4.76059000 | 1.91367700 | -0.69552700 |
| C | 1.87983100 | 2.44635400 | -4.21907000 |
| H | 2.45825000 | 3.09383000 | -4.88886100 |
| H | 0.93870100 | 2.20462900 | -4.71832000 |

| | | | |
|---|---|---|---|
| H | 1.64540900 | 3.03254300 | -3.32296400 |
| C | 5.84455100 | 2.78644900 | -3.16379100 |
| H | 5.96607900 | 2.73513400 | -4.25123200 |
| H | 6.15671100 | 3.78417900 | -2.83421500 |
| H | 6.52413600 | 2.06100700 | -2.70305900 |
| C | 5.36748300 | -0.56474000 | -4.11633000 |
| H | 4.71217400 | -0.78454300 | -4.96501300 |
| H | 6.15681000 | 0.11076600 | -4.46268300 |
| H | 5.83795100 | -1.50341200 | -3.79931800 |
| O | 3.02234900 | -1.75110400 | 0.99553000 |
| S | 3.08386300 | -0.72035400 | 2.06906100 |
| O | 4.33218800 | 0.04325200 | 2.16924200 |
| O | 1.82314300 | 0.10326100 | 2.12220500 |
| C | 2.96535100 | -1.69392800 | 3.64672500 |
| F | 1.84420300 | -2.42741200 | 3.65572600 |
| F | 4.01788700 | -2.51309000 | 3.74849700 |
| F | 2.95169100 | -0.86934800 | 4.69871800 |
| H | 1.29573600 | -1.97049100 | 0.63788500 |
| C | -1.65639200 | -4.53801100 | -2.76410300 |
| H | -1.74592400 | -3.98238600 | -3.70411400 |
| H | -0.61562800 | -4.85897200 | -2.65552900 |
| H | -2.27300700 | -5.43958900 | -2.85940300 |
| C | -3.59396800 | -3.24181600 | -1.77892300 |
| H | -3.70827900 | -2.62247200 | -2.67259700 |
| H | -4.24128600 | -4.11878500 | -1.90098200 |
| H | -3.96407800 | -2.67368600 | -0.91728200 |
| H | -1.30854500 | -4.07883800 | 1.12257800 |
| H | 0.39998200 | -3.93558100 | 1.50406300 |
| C | -0.00716400 | -5.50635200 | 0.12718700 |
| C | 1.19547800 | -5.76190000 | -0.54944400 |
| C | -0.89748900 | -6.56634800 | 0.34075700 |
| C | 1.49441200 | -7.04573700 | -1.00933700 |
| H | 1.89669700 | -4.94899200 | -0.71741100 |
| C | -0.59826900 | -7.85438800 | -0.11414700 |
| H | -1.83190600 | -6.38262200 | 0.86645900 |
| C | 0.59712400 | -8.09713500 | -0.79409300 |
| H | 2.42914300 | -7.22641300 | -1.53431300 |
| H | -1.30209600 | -8.66444300 | 0.05922000 |
| H | 0.82973900 | -9.09642000 | -1.15286800 |
| C | -2.14358400 | -3.71314300 | -1.56056700 |
| H | -2.19906800 | -4.39263500 | -0.70892300 |
| F | -4.32137000 | 1.19338800 | -5.42715400 |

Energies (0K) = -4290.1654842

Energies (0K) + ZPE = -4289.465462

Enthalpies (298K) = -4289.403203
Free Energies (298K) = -4289.569986

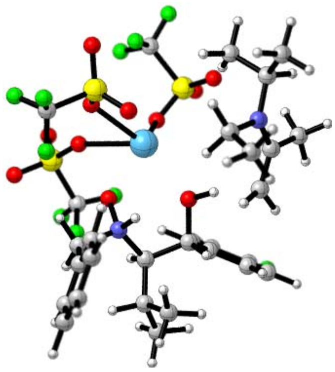

**E′**
Number of imaginary frequencies = 0
0 1

| | | | |
|---|---|---|---|
| C | -0.37861800 | -3.86437800 | 0.58062100 |
| C | -1.47135000 | -2.51979600 | -1.39499400 |
| N | -0.38081800 | -2.62533900 | -0.31241600 |
| O | -0.51632700 | -1.54032900 | 0.54739500 |
| O | -2.97366200 | 1.60574400 | 4.04100400 |
| S | -2.50884800 | 0.36999300 | 3.39320100 |
| O | -1.25707400 | 0.55926700 | 2.57178100 |
| O | -2.48201900 | -0.85165100 | 4.21199800 |
| C | -3.78962200 | 0.02291000 | 2.08452900 |
| F | -5.01920100 | 0.10403900 | 2.60597300 |
| F | -3.69243500 | 0.91929700 | 1.08277900 |
| F | -3.62988100 | -1.20020400 | 1.55887900 |
| O | 0.06696100 | 4.47883600 | -0.57582200 |
| S | -1.04626200 | 4.42658400 | 0.39411800 |
| O | -2.14899400 | 5.37844400 | 0.20141500 |
| O | -1.50638000 | 3.02287800 | 0.70777400 |
| C | -0.26072800 | 4.91703600 | 2.00297200 |
| F | 0.74155000 | 4.06696900 | 2.30033400 |
| F | -1.15113800 | 4.88153500 | 3.00164400 |
| F | 0.24600800 | 6.15365800 | 1.92131700 |
| O | 0.01052600 | -0.59670800 | -1.91377900 |
| C | -1.01730900 | -1.46627700 | -2.45423800 |
| C | -2.15972300 | -0.64029100 | -3.02147900 |
| C | -2.27588500 | -0.49270200 | -4.41138400 |
| C | -3.07085500 | 0.03438100 | -2.19400300 |
| C | -3.27461200 | 0.30497100 | -4.97268700 |
| H | -1.58092700 | -1.01073300 | -5.06701800 |
| C | -4.07262100 | 0.84024100 | -2.73602000 |
| H | -3.01645600 | -0.06281500 | -1.11345900 |

| | | | |
|---|---|---|---|
| C | -4.15451800 | 0.95867500 | -4.11930900 |
| H | -3.37306200 | 0.42054700 | -6.04731700 |
| H | -4.78390900 | 1.36190200 | -2.10349300 |
| La | -0.06028000 | 0.81630000 | 0.37952600 |
| H | -2.30871900 | -2.14409800 | -0.80642800 |
| H | -0.54338700 | -2.00235700 | -3.27800000 |
| C | 2.69630100 | 0.67680500 | -3.94505900 |
| N | 3.90932700 | 0.62756400 | -3.30942800 |
| C | 1.88616200 | -0.41917500 | -4.14464400 |
| H | 0.67030800 | -0.40145700 | -2.64939900 |
| H | 1.03186200 | -0.30642800 | -4.80491800 |
| H | 2.21078700 | -1.43522300 | -3.95155600 |
| C | 4.38550600 | -0.65501100 | -2.77183700 |
| H | 3.54156700 | -1.17738900 | -2.30721900 |
| H | 5.08849600 | -0.44396200 | -1.96398100 |
| C | 4.67942800 | 1.84590900 | -2.96158200 |
| H | 4.29625000 | 2.64834900 | -3.59077400 |
| C | 4.46368200 | 2.25680700 | -1.49663800 |
| H | 5.00485600 | 3.18566800 | -1.28090700 |
| H | 3.40004000 | 2.43269200 | -1.29382300 |
| H | 4.82269400 | 1.49055400 | -0.80063900 |
| C | 2.21117400 | 2.01641600 | -4.46134000 |
| H | 2.89565600 | 2.42876700 | -5.21238800 |
| H | 1.23240600 | 1.89531700 | -4.93097700 |
| H | 2.11360200 | 2.76171500 | -3.66367800 |
| C | 6.17002000 | 1.70073500 | -3.29807400 |
| H | 6.31183300 | 1.43056900 | -4.35019100 |
| H | 6.67338800 | 2.65871800 | -3.12424000 |
| H | 6.66909200 | 0.94990400 | -2.67578100 |
| C | 5.05751200 | -1.55814000 | -3.81254300 |
| H | 4.39352600 | -1.76363600 | -4.65818200 |
| H | 5.97278700 | -1.10077000 | -4.20220900 |
| H | 5.32699000 | -2.51691600 | -3.35281300 |
| O | 3.38628700 | -0.77241500 | 0.66449900 |
| S | 3.10136900 | -0.33142200 | 2.04615500 |
| O | 4.10156900 | 0.53669900 | 2.68699200 |
| O | 1.67797800 | 0.13063400 | 2.23881400 |
| C | 3.12537000 | -1.89058100 | 3.05688100 |
| F | 2.27574600 | -2.80086500 | 2.55286300 |
| F | 4.35740400 | -2.41759000 | 3.05143700 |
| F | 2.77258800 | -1.63541800 | 4.32284000 |
| C | -0.91759200 | -4.46213400 | -3.05678600 |
| H | -0.97130200 | -3.94480300 | -4.02148600 |
| H | 0.12116300 | -4.43059000 | -2.71213200 |

| | | | |
|---|---|---|---|
| H | -1.17330400 | -5.51174200 | -3.23954500 |
| C | -3.31073700 | -3.75658800 | -2.64456600 |
| H | -3.33292800 | -3.10684900 | -3.52507200 |
| H | -3.64697200 | -4.75142200 | -2.95894300 |
| H | -4.03517800 | -3.37172000 | -1.91743500 |
| H | -1.42002400 | -4.10703500 | 0.79320100 |
| H | 0.07669400 | -3.47937200 | 1.49277200 |
| C | 0.40637900 | -5.04737600 | 0.06635200 |
| C | 1.78661700 | -4.93341200 | -0.16403000 |
| C | -0.20716900 | -6.29673900 | -0.09439100 |
| C | 2.52943000 | -6.04009400 | -0.57859700 |
| H | 2.28646100 | -3.98134800 | 0.00013700 |
| C | 0.53826700 | -7.40845500 | -0.49420700 |
| H | -1.27093300 | -6.40185300 | 0.10335800 |
| C | 1.90637900 | -7.28076900 | -0.74495100 |
| H | 3.59645000 | -5.93723900 | -0.75657300 |
| H | 0.04881100 | -8.37178200 | -0.60939000 |
| H | 2.48665700 | -8.14418300 | -1.05901200 |
| C | -1.90085000 | -3.86791200 | -2.03425400 |
| H | -1.99690500 | -4.57633300 | -1.20771500 |
| F | -5.12720500 | 1.73443300 | -4.65155100 |
| H | 0.53458500 | -2.56292800 | -0.77776900 |

Energies (0K) = -4290.1723782

Energies (0K) + ZPE = -4289.470544

Enthalpies (298K) = -4289.408297

Free Energies (298K) = -4289.57493

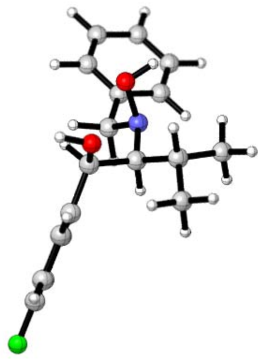

**F**

Number of imaginary frequencies = 0

0 1

| | | | |
|---|---|---|---|
| C | 0.17406400 | 3.73791600 | -0.46835000 |
| C | 1.68033600 | 5.69895700 | -0.07835000 |
| H | 2.51525800 | 5.09205700 | 0.30862200 |
| N | 0.91089500 | 4.87319900 | -1.04013200 |
| O | 1.91618500 | 4.28179000 | -1.90999300 |

| | | | |
|---|---|---|---|
| C | 3.70654400 | 6.72364600 | -1.30279000 |
| H | 3.71685600 | 5.97884400 | -2.10389600 |
| H | 4.13146400 | 7.65743100 | -1.69321100 |
| H | 4.37062200 | 6.36668200 | -0.50508400 |
| C | 1.38237100 | 7.56126300 | -1.86661600 |
| H | 1.79554600 | 8.52122100 | -2.20237600 |
| H | 1.31538200 | 6.90820800 | -2.74456900 |
| H | 0.36838900 | 7.73644900 | -1.49606800 |
| H | 0.83166800 | 3.06828400 | 0.10961500 |
| H | -0.57271200 | 4.15529900 | 0.21357300 |
| C | -0.54604000 | 2.95044900 | -1.54628400 |
| C | -0.34950700 | 1.57128200 | -1.68182800 |
| C | -1.44980800 | 3.59027600 | -2.40842400 |
| C | -1.04277000 | 0.84133300 | -2.65291500 |
| H | 0.35228700 | 1.06457300 | -1.02302500 |
| C | -2.13952100 | 2.86646100 | -3.38243300 |
| H | -1.60679400 | 4.66186200 | -2.31362700 |
| C | -1.93921000 | 1.48724000 | -3.50701100 |
| H | -0.87734000 | -0.22938100 | -2.74336700 |
| H | -2.83668500 | 3.37690500 | -4.04240800 |
| H | -2.47746200 | 0.92291300 | -4.26423300 |
| C | 2.28184700 | 6.96251200 | -0.77485100 |
| H | 2.37330700 | 7.72250500 | 0.00968200 |
| C | 1.61439700 | 6.80331300 | 2.23464000 |
| C | 2.55559100 | 6.12915700 | 3.02760200 |
| C | 3.28468500 | 6.80046300 | 4.00930200 |
| C | 3.05100200 | 8.15869800 | 4.19553000 |
| C | 2.12010700 | 8.85917800 | 3.44034300 |
| C | 1.40489100 | 8.17044900 | 2.45703100 |
| H | 2.72052900 | 5.06425900 | 2.88199300 |
| H | 4.01455900 | 6.28664100 | 4.62706200 |
| H | 1.96171100 | 9.91799900 | 3.62001300 |
| H | 0.67312600 | 8.70263700 | 1.85777200 |
| C | 0.82595700 | 6.03767200 | 1.17419200 |
| H | 0.53790600 | 5.08532500 | 1.63839600 |
| O | -0.34239800 | 6.73658900 | 0.74969300 |
| F | 3.75132500 | 8.81807200 | 5.15231000 |
| H | 1.61085800 | 4.54112500 | -2.79690900 |
| H | -0.89610400 | 6.88274800 | 1.53743200 |

Energies (0K) = -1004.127191

Energies (0K) + ZPE = -1003.758574

Enthalpies (298K) = -1003.73614

Free Energies (298K) = -1003.811034

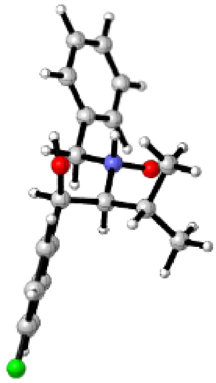

**F′**

Number of imaginary frequencies = 0

0 1

| | | | |
|---|---|---|---|
| C | -1.24137000 | -0.37380000 | -2.79426000 |
| C | -0.43304700 | 0.68696200 | -0.60660000 |
| H | 0.56671600 | 0.67541700 | -1.04788900 |
| N | -1.33562400 | 0.80633300 | -1.83719900 |
| O | -1.06505700 | 1.96432500 | -2.51159200 |
| C | 0.43112900 | 3.03547700 | -0.04479300 |
| H | 0.27153900 | 3.38526200 | -1.06749800 |
| H | 0.33849200 | 3.88318300 | 0.64660600 |
| H | 1.45724400 | 2.65316800 | 0.03376800 |
| C | -2.02101500 | 2.51710900 | 0.34066600 |
| H | -2.06602700 | 3.34551300 | 1.05817600 |
| H | -2.30630100 | 2.90085900 | -0.64457500 |
| H | -2.74775000 | 1.76309800 | 0.66123800 |
| H | -0.20190700 | -0.37509300 | -3.12842700 |
| H | -1.45608900 | -1.30094900 | -2.25906200 |
| C | -2.21083200 | -0.21017700 | -3.93537700 |
| C | -1.90499900 | 0.60689700 | -5.03450200 |
| C | -3.44447600 | -0.87439900 | -3.90632900 |
| C | -2.81583400 | 0.75311800 | -6.08164600 |
| H | -0.95580000 | 1.13062900 | -5.05390000 |
| C | -4.36096100 | -0.72506900 | -4.95174100 |
| H | -3.68857000 | -1.51475000 | -3.06133200 |
| C | -4.04689300 | 0.08898400 | -6.04222800 |
| H | -2.56690300 | 1.38602100 | -6.92976800 |
| H | -5.31325500 | -1.24769800 | -4.91545700 |
| H | -4.75458600 | 0.20335800 | -6.85936800 |
| C | -0.59335400 | 1.94443300 | 0.30116200 |
| H | -0.36366100 | 1.59935900 | 1.31665100 |
| C | 0.48984300 | -0.84466500 | 1.19679600 |
| C | 1.80901900 | -1.03362400 | 0.75646000 |
| C | 2.84886800 | -1.23735500 | 1.66348200 |
| C | 2.55057000 | -1.25324100 | 3.02141400 |

| | | | |
|---|---|---|---|
| C | 1.25766700 | -1.07780500 | 3.49526400 |
| C | 0.22799800 | -0.87509400 | 2.57213300 |
| H | 2.03210300 | -1.02989200 | -0.30747900 |
| H | 3.87107500 | -1.38718800 | 1.33071000 |
| H | 1.06306200 | -1.10306900 | 4.56304700 |
| H | -0.78911200 | -0.74857300 | 2.92468800 |
| C | -0.62653900 | -0.64253200 | 0.17929400 |
| H | -0.56404800 | -1.48259900 | -0.52132400 |
| O | -1.88146900 | -0.68184500 | 0.86101300 |
| F | 3.55642800 | -1.45276200 | 3.90977300 |
| H | -2.31058500 | 0.81185100 | -1.49352900 |
| H | -2.56340200 | -0.95361800 | 0.22318000 |

Energies (0K) = -1004.109395

Energies (0K) + ZPE = -1003.738422

Enthalpies (298K) = -1003.716544

Free Energies (298K) = -1003.78953

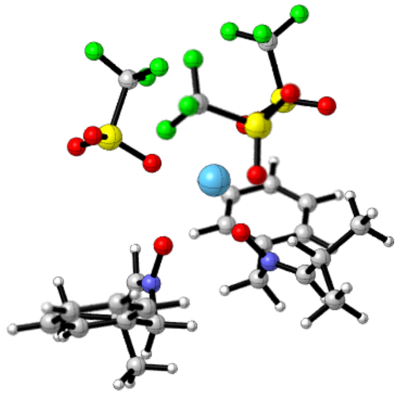

**[La(OTf)$_3$(1a)$_2$]**

Number of imaginary frequencies = 0

0 1

| | | | |
|---|---|---|---|
| O | -2.08217700 | 1.04646900 | 1.26798800 |
| O | 2.02653600 | 3.86349400 | -0.14756300 |
| S | 0.62567600 | 4.19081100 | -0.44863900 |
| O | 0.27763400 | 4.46227100 | -1.85536400 |
| O | -0.35982400 | 3.27232400 | 0.23953100 |
| C | 0.30294500 | 5.79608700 | 0.42665200 |
| F | 0.48952400 | 5.66683000 | 1.74643000 |
| F | 1.12524400 | 6.74723100 | -0.03258700 |
| F | -0.96523800 | 6.18803600 | 0.21306500 |
| O | -5.34574700 | 4.25575100 | 2.03879100 |
| S | -4.05200100 | 4.58387000 | 2.67140700 |
| O | -3.97601500 | 4.48775100 | 4.13654800 |
| O | -2.86846700 | 3.95357800 | 1.97241100 |
| C | -3.79227800 | 6.38398500 | 2.29042900 |

| | | | |
|---|---|---|---|
| F | -3.81818200 | 6.58395800 | 0.96131500 |
| F | -2.60965300 | 6.80056800 | 2.75908100 |
| F | -4.76081200 | 7.12027200 | 2.84947200 |
| O | -4.22005400 | 5.42163100 | -2.70456100 |
| S | -4.84859900 | 4.19415100 | -3.23386500 |
| O | -4.96620700 | 4.07778100 | -4.69471200 |
| O | -4.35916000 | 2.93576800 | -2.56229000 |
| C | -6.60133300 | 4.28271100 | -2.62575600 |
| F | -6.62730900 | 4.33493200 | -1.28191600 |
| F | -7.29716500 | 3.20525000 | -3.01669600 |
| F | -7.20592300 | 5.37829700 | -3.10318700 |
| La | -2.81121600 | 2.77040500 | -0.37423900 |
| C | -2.07236300 | 1.15221900 | 3.62483900 |
| C | -0.02072400 | 1.05330100 | 2.25268400 |
| H | 0.33962000 | 1.03399500 | 1.22953800 |
| N | -1.31034700 | 1.08851100 | 2.35349200 |
| C | 2.07183400 | 0.01580700 | 3.07411100 |
| H | 2.57896200 | 0.20651600 | 2.12066700 |
| H | 2.82481300 | 0.03580500 | 3.86946200 |
| H | 1.64164200 | -0.99133600 | 3.03724300 |
| C | 1.61979600 | 2.49393300 | 3.43121500 |
| H | 2.39123400 | 2.50791500 | 4.20963600 |
| H | 2.08527300 | 2.77394000 | 2.47995100 |
| H | 0.86596900 | 3.24878600 | 3.67919100 |
| H | -1.36055300 | 1.25620700 | 4.44393500 |
| H | -2.66159900 | 2.06757700 | 3.56598200 |
| C | -2.96167000 | -0.05640000 | 3.82756100 |
| C | -2.44014000 | -1.35741500 | 3.78846200 |
| C | -4.31877600 | 0.12480300 | 4.12179500 |
| C | -3.26524000 | -2.45708000 | 4.02969200 |
| H | -1.38710300 | -1.51287000 | 3.56636500 |
| C | -5.14382400 | -0.97559500 | 4.37436100 |
| H | -4.72812700 | 1.13112600 | 4.16466100 |
| C | -4.61966600 | -2.26904200 | 4.32567300 |
| H | -2.84956600 | -3.46060400 | 3.99423700 |
| H | -6.19414000 | -0.81945000 | 4.60558200 |
| H | -5.25944300 | -3.12576100 | 4.51959700 |
| C | 0.99642900 | 1.08218800 | 3.35417200 |
| H | 0.52940400 | 0.85644800 | 4.31631300 |
| C | -4.77179200 | -0.95091700 | 0.18489900 |
| C | -2.63112000 | -1.61150600 | -0.77664100 |
| H | -2.82028800 | -2.56096000 | -0.28628600 |
| N | -3.54445400 | -0.70814500 | -0.62458000 |
| C | -0.22089300 | -2.18512500 | -0.98362400 |

| | | | |
|---|---|---|---|
| H | -0.42344900 | -3.26283400 | -0.95614700 |
| H | 0.68325600 | -2.02629800 | -1.58151000 |
| H | -0.01441600 | -1.85130100 | 0.03971900 |
| C | -1.67152500 | -1.87056600 | -3.05272700 |
| H | -0.77380300 | -1.71286000 | -3.66111400 |
| H | -1.92693600 | -2.93589300 | -3.09063000 |
| H | -2.49394800 | -1.30192700 | -3.49957100 |
| H | -4.64792600 | -1.91854100 | 0.67416100 |
| H | -4.78148900 | -0.16653800 | 0.94455600 |
| C | -6.02128700 | -0.90886800 | -0.66433000 |
| C | -6.38230900 | -2.01566800 | -1.44540000 |
| C | -6.83156300 | 0.23301100 | -0.68204500 |
| C | -7.53105400 | -1.97670900 | -2.23752300 |
| H | -5.76268700 | -2.90936500 | -1.43148300 |
| C | -7.98086900 | 0.27504400 | -1.47555700 |
| H | -6.56494700 | 1.08911100 | -0.06702700 |
| C | -8.33139400 | -0.83002000 | -2.25538600 |
| H | -7.80316100 | -2.84106700 | -2.83744200 |
| H | -8.59555700 | 1.17065300 | -1.48735400 |
| H | -9.22603600 | -0.80009200 | -2.87165300 |
| C | -1.39921600 | -1.41653800 | -1.60085600 |
| H | -1.17024100 | -0.34587800 | -1.61706900 |
| O | -3.49008800 | 0.47578700 | -1.20289900 |

Energies (0K) = -4032.4850664
Energies (0K) + ZPE = -4031.913582
Enthalpies (298K) = -4031.85723
Free Energies (298K) = -4032.013329

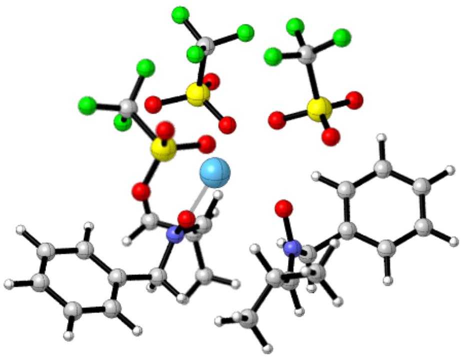

**[La(OTf)$_3$(1a)$_2$]$^-$**
Number of imaginary frequencies = 0
-1 2

| | | | |
|---|---|---|---|
| O | -2.53793100 | 1.30747800 | 1.43716500 |
| O | 1.19979400 | 5.00834600 | 1.37653600 |
| S | -0.23776900 | 5.19902100 | 1.12630000 |
| O | -0.60985900 | 6.01735400 | -0.04474900 |

| | | | |
|---|---|---|---|
| O | -1.04399000 | 3.93231800 | 1.26560500 |
| C | -0.84082900 | 6.17296400 | 2.58910300 |
| F | -0.68149900 | 5.46956400 | 3.72168800 |
| F | -0.15083300 | 7.31805600 | 2.69933000 |
| F | -2.14041100 | 6.47451100 | 2.45811700 |
| O | -6.43381900 | 2.91207300 | 2.02352100 |
| S | -5.70432300 | 4.17965800 | 2.21698200 |
| O | -5.98134000 | 4.93683000 | 3.44590300 |
| O | -4.23521100 | 4.08852300 | 1.88678200 |
| C | -6.32233100 | 5.26324300 | 0.84030700 |
| F | -6.12340500 | 4.64950500 | -0.34354400 |
| F | -5.66729600 | 6.43374600 | 0.81623400 |
| F | -7.63107400 | 5.50845500 | 0.97414800 |
| O | -2.39946000 | 3.92770200 | -3.85424900 |
| S | -3.86688800 | 4.05653100 | -3.72152000 |
| O | -4.65531200 | 3.94905300 | -4.96053100 |
| O | -4.43695300 | 3.29949900 | -2.55919300 |
| C | -4.10040400 | 5.81680200 | -3.17779800 |
| F | -3.49297400 | 6.02016800 | -1.98857900 |
| F | -5.40073000 | 6.10961100 | -3.03279800 |
| F | -3.57029800 | 6.66958100 | -4.06591700 |
| La | -3.01858600 | 2.91060500 | -0.12531500 |
| C | -1.46135600 | 1.38833500 | 3.52331800 |
| C | -0.49625600 | 0.18113800 | 1.56612500 |
| H | -0.79040100 | -0.09836600 | 0.55684300 |
| N | -1.60383800 | 0.68305100 | 2.25218600 |
| C | 1.68083000 | -0.00940700 | 2.95773900 |
| H | 1.34787000 | 0.47565600 | 3.88269700 |
| H | 2.46737500 | -0.72501500 | 3.23127800 |
| H | 2.13104600 | 0.76124900 | 2.32089600 |
| C | -0.07864300 | -1.83628700 | 3.11268000 |
| H | 0.69380400 | -2.54817900 | 3.43232200 |
| H | -0.53560500 | -1.41991500 | 4.01817500 |
| H | -0.85248700 | -2.39867900 | 2.57603500 |
| H | -0.62451800 | 0.93010400 | 4.05719100 |
| H | -1.19891900 | 2.43781300 | 3.33313200 |
| C | -2.70085900 | 1.31654400 | 4.39779100 |
| C | -3.09727100 | 2.43885500 | 5.13699000 |
| C | -3.42970900 | 0.12687700 | 4.54108200 |
| C | -4.19534100 | 2.37751900 | 6.00101900 |
| H | -2.54433900 | 3.36973500 | 5.02884300 |
| C | -4.53031300 | 0.06322400 | 5.39848500 |
| H | -3.13484100 | -0.74945300 | 3.97012200 |
| C | -4.91610200 | 1.18845100 | 6.13442900 |

| | | | |
|---|---|---|---|
| H | -4.49003900 | 3.26070800 | 6.56231000 |
| H | -5.08638700 | -0.86627500 | 5.49457400 |
| H | -5.77315700 | 1.13899500 | 6.80125500 |
| C | 0.53153900 | -0.73176000 | 2.21912900 |
| H | 1.00466100 | -1.24299500 | 1.36861100 |
| C | -4.42483000 | -1.01310000 | 0.07667700 |
| C | -2.51771500 | -1.25985100 | -1.42673100 |
| H | -2.48666500 | -2.28099300 | -1.06188500 |
| N | -3.45714700 | -0.51171600 | -0.94057400 |
| C | -0.23460900 | -1.58558800 | -2.36447400 |
| H | -0.40911700 | -2.64987000 | -2.56538500 |
| H | 0.48641300 | -1.21694200 | -3.10242800 |
| H | 0.22299600 | -1.49770900 | -1.37221700 |
| C | -2.16601200 | -0.88024400 | -3.86657900 |
| H | -1.45857100 | -0.49714800 | -4.61091700 |
| H | -2.40045600 | -1.92035600 | -4.12176300 |
| H | -3.08638200 | -0.29130900 | -3.93150700 |
| H | -4.09535800 | -2.01105100 | 0.37357900 |
| H | -4.31818600 | -0.33027500 | 0.92209400 |
| C | -5.83603100 | -1.03397100 | -0.46447700 |
| C | -6.24747200 | -2.06723700 | -1.31847100 |
| C | -6.74564400 | -0.02430000 | -0.12492900 |
| C | -7.54501500 | -2.08626700 | -1.83289100 |
| H | -5.54852400 | -2.85838100 | -1.58065300 |
| C | -8.04586000 | -0.04375600 | -0.63802100 |
| H | -6.44115900 | 0.77410400 | 0.54764200 |
| C | -8.44677800 | -1.07231900 | -1.49443700 |
| H | -7.85315900 | -2.89248000 | -2.49349000 |
| H | -8.74406200 | 0.74370600 | -0.36640500 |
| H | -9.45775200 | -1.08728400 | -1.89313600 |
| C | -1.54081000 | -0.78368600 | -2.45701900 |
| H | -1.33327700 | 0.27413700 | -2.25423100 |
| O | -3.65431700 | 0.73045800 | -1.33097400 |

Energies (0K) = -4032.5463442

Energies (0K) + ZPE = -4031.979277

Enthalpies (298K) = -4031.922104

Free Energies (298K) = -4032.081724

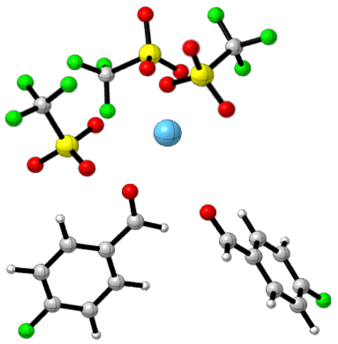

**La(OTf)$_3$(FBA)$_2$**

Number of imaginary frequencies = 0

0 1

| | | | |
|---|---|---|---|
| O | 2.31748100 | 3.45185900 | 0.84494000 |
| S | 1.64727100 | 4.53341500 | 1.57721400 |
| O | 2.32633100 | 5.84097400 | 1.63823800 |
| O | 0.17024200 | 4.64204700 | 1.26886200 |
| C | 1.59976200 | 3.96572800 | 3.34454400 |
| F | 0.99831200 | 2.77656400 | 3.45542700 |
| F | 2.83564500 | 3.87217100 | 3.84726300 |
| F | 0.90730000 | 4.85906800 | 4.08523200 |
| O | -3.37606100 | 2.39770100 | 4.15634300 |
| S | -3.33447000 | 3.82915800 | 3.83551900 |
| O | -3.28339000 | 4.78431500 | 4.95962400 |
| O | -2.35657500 | 4.16618200 | 2.72715900 |
| C | -4.95722500 | 4.19851100 | 3.01099700 |
| F | -5.12622000 | 3.44544700 | 1.91899700 |
| F | -4.99217400 | 5.49513900 | 2.64504800 |
| F | -5.97187300 | 3.96955700 | 3.85332700 |
| O | -3.98140400 | 6.35375600 | -1.87137000 |
| S | -2.68033400 | 5.91451600 | -1.34217800 |
| O | -1.49488300 | 6.71051300 | -1.71107200 |
| O | -2.72024800 | 5.54969100 | 0.12897800 |
| C | -2.40543400 | 4.24485900 | -2.11093500 |
| F | -3.39927000 | 3.41126600 | -1.77822800 |
| F | -1.24657800 | 3.71779400 | -1.69783800 |
| F | -2.37037800 | 4.36207500 | -3.44565500 |
| La | -1.46814700 | 6.48439800 | 2.08524800 |
| C | -4.42139000 | 10.17861800 | 0.45981400 |
| C | -4.77401800 | 11.50424900 | 0.77924300 |
| C | -5.56639300 | 12.24916500 | -0.08608100 |
| C | -5.99545000 | 11.64361600 | -1.26481900 |
| C | -5.66875600 | 10.33098900 | -1.61125500 |
| C | -4.87625700 | 9.59587900 | -0.74238800 |
| H | -4.41994300 | 11.94655700 | 1.70631200 |
| H | -5.85138800 | 13.27288900 | 0.13176700 |
| H | -6.03476600 | 9.91465400 | -2.54384700 |

| | | | |
|---|---|---|---|
| H | -4.60320400 | 8.57296700 | -0.98489900 |
| C | -3.57524200 | 9.44261900 | 1.38310900 |
| H | -3.27508700 | 9.97389300 | 2.29927600 |
| O | -3.18700200 | 8.28103200 | 1.18891700 |
| C | -0.24408400 | 11.53208900 | 3.33448900 |
| C | 0.36958700 | 12.79662300 | 3.31135900 |
| C | 0.18838000 | 13.68844700 | 4.36406500 |
| C | -0.61203800 | 13.28939200 | 5.43061500 |
| C | -1.23498700 | 12.04171100 | 5.48855800 |
| C | -1.04565200 | 11.16197700 | 4.43164100 |
| H | 0.98833100 | 13.07798500 | 2.46348000 |
| H | 0.64951000 | 14.67030700 | 4.37092900 |
| H | -1.84638900 | 11.78514100 | 6.34739200 |
| H | -1.51458600 | 10.18306600 | 4.44573300 |
| C | -0.03393500 | 10.62266800 | 2.20539900 |
| H | 0.62175800 | 11.00578500 | 1.40253300 |
| O | -0.53208200 | 9.50295500 | 2.10308500 |
| F | -0.79432100 | 14.14387200 | 6.45533200 |
| F | -6.76069200 | 12.35539700 | -2.11118300 |

Energies (0K) = -3805.834271

Energies (0K) + ZPE = -3805.540918

Enthalpies (298K) = -3805.496037

Free Energies (298K) = -3805.626039

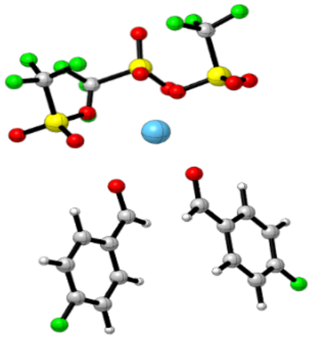

**$[La(OTf)_3(FBA)_2]^-$**

Number of imaginary frequencies = 1

-1 2

| | | | |
|---|---|---|---|
| O | 2.49920200 | 6.03529600 | 1.28532300 |
| S | 1.44907100 | 5.46865400 | 2.14370900 |
| O | 1.51452000 | 5.77299600 | 3.58567200 |
| O | 0.05626700 | 5.61734000 | 1.57045000 |
| C | 1.68412600 | 3.62971300 | 2.02584200 |
| F | 1.51820600 | 3.21925200 | 0.76124000 |
| F | 2.92173400 | 3.30224900 | 2.42439700 |
| F | 0.80323200 | 2.98679400 | 2.80309200 |

| | | | |
|---|---|---|---|
| O | -2.97785300 | 2.33011100 | 4.86150100 |
| S | -3.16005200 | 3.74397800 | 4.50497700 |
| O | -3.13982100 | 4.72739900 | 5.60634800 |
| O | -2.36125900 | 4.18316800 | 3.30519100 |
| C | -4.88552700 | 3.85326900 | 3.82718300 |
| F | -5.07277600 | 2.96426600 | 2.84395400 |
| F | -5.09450500 | 5.08780500 | 3.32081100 |
| F | -5.79504400 | 3.63314400 | 4.78435800 |
| O | -4.56583500 | 5.70978700 | -1.33744900 |
| S | -3.15447800 | 5.65745700 | -0.92727500 |
| O | -2.24322100 | 6.66253800 | -1.50180700 |
| O | -2.97108600 | 5.44395900 | 0.55945400 |
| C | -2.53272100 | 4.03207500 | -1.57764600 |
| F | -3.23899500 | 3.01632100 | -1.06185100 |
| F | -1.24203900 | 3.85646800 | -1.26135200 |
| F | -2.65139700 | 3.99935300 | -2.91318400 |
| La | -2.03256400 | 6.70388500 | 2.52043400 |
| C | -3.90726400 | 10.62504600 | 0.42884300 |
| C | -4.25502400 | 11.95921900 | 0.73847800 |
| C | -4.49792700 | 12.88455300 | -0.27130200 |
| C | -4.39136000 | 12.46380300 | -1.59479700 |
| C | -4.05119500 | 11.15640600 | -1.94205800 |
| C | -3.81118600 | 10.23772400 | -0.92801700 |
| H | -4.33639900 | 12.26342100 | 1.77864300 |
| H | -4.76825600 | 13.91234100 | -0.05062400 |
| H | -3.98084400 | 10.88002800 | -2.98949700 |
| H | -3.53849800 | 9.21510400 | -1.17000100 |
| C | -3.66038800 | 9.68125900 | 1.50333600 |
| H | -3.76522600 | 10.06659500 | 2.52696900 |
| O | -3.47057600 | 8.44294600 | 1.31953800 |
| C | -1.02332200 | 11.02015000 | 2.98979700 |
| C | -1.07391600 | 12.40697200 | 2.70176800 |
| C | -1.14150800 | 13.34902900 | 3.72144600 |
| C | -1.15264300 | 12.90310200 | 5.04224700 |
| C | -1.09529800 | 11.54983700 | 5.37242200 |
| C | -1.02926000 | 10.61328100 | 4.34762400 |
| H | -1.05667100 | 12.73655400 | 1.66628900 |
| H | -1.17905000 | 14.41338500 | 3.51072200 |
| H | -1.09553300 | 11.25095100 | 6.41622400 |
| H | -0.97006900 | 9.55609300 | 4.58898800 |
| C | -0.95418900 | 10.06638500 | 1.90719200 |
| H | -0.86501500 | 10.47134900 | 0.89156400 |
| O | -0.82594200 | 8.80318200 | 2.06692400 |
| F | -1.21419400 | 13.81748500 | 6.04137000 |

| | | | |
|---|---|---|---|
| F | -4.62884900 | 13.35776600 | -2.58319900 |

Energies (0K) = -3805.9408495

Energies (0K) + ZPE = -3805.650977

Enthalpies (298K) = -3805.605715

Free Energies (298K) = -3805.738249

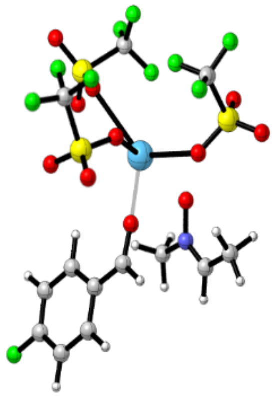

**B1**

Number of imaginary frequencies = 0

-1 2

| | | | |
|---|---|---|---|
| C | -1.22422200 | -4.20628300 | 1.88427000 |
| C | -3.35799200 | -3.27824200 | 1.19650400 |
| N | -2.15551900 | -3.09589700 | 1.63956400 |
| O | -1.68850600 | -1.90019900 | 1.94340500 |
| O | 4.63348500 | 1.37334300 | 1.38639000 |
| S | 3.37988600 | 0.63636400 | 1.60789800 |
| O | 2.30752500 | 0.93437700 | 0.59414200 |
| O | 3.49709100 | -0.79741500 | 1.94632800 |
| C | 2.65989000 | 1.39082200 | 3.14617700 |
| F | 3.46480800 | 1.19867400 | 4.19828600 |
| F | 2.46029000 | 2.70704500 | 2.99365800 |
| F | 1.46483400 | 0.82056400 | 3.42416600 |
| O | -4.31213600 | 2.23492100 | 1.38049500 |
| S | -2.87470100 | 1.95713000 | 1.49964400 |
| O | -2.30552700 | 1.86214200 | 2.85725300 |
| O | -2.39842600 | 0.85598100 | 0.57973900 |
| C | -2.02963400 | 3.44144100 | 0.76936000 |
| F | -2.41945700 | 3.64542000 | -0.49452700 |
| F | -0.69499000 | 3.25627700 | 0.77949000 |
| F | -2.30749500 | 4.53859800 | 1.48559000 |
| O | -0.05433400 | 1.14588900 | -1.50576700 |
| S | 0.51632800 | 0.94181700 | -2.88719900 |
| O | 1.57168100 | -0.08542900 | -2.95219100 |
| O | -0.50372900 | 0.92051100 | -3.94873000 |
| C | 1.41002500 | 2.55020300 | -3.14358400 |

| | | | |
|---|---|---|---|
| F | 0.55426100 | 3.58068700 | -3.06364300 |
| F | 1.98188900 | 2.56775200 | -4.35801200 |
| F | 2.36827800 | 2.72307400 | -2.22358600 |
| O | -1.08417400 | -1.80221100 | -1.01950400 |
| C | -1.48404500 | -2.75533200 | -1.82572800 |
| C | -0.70595000 | -3.88942000 | -2.17356400 |
| C | -1.23155400 | -4.88642900 | -3.05334800 |
| C | 0.61628800 | -4.09574100 | -1.66876900 |
| C | -0.49596300 | -6.00922000 | -3.39896100 |
| H | -2.23318600 | -4.75709800 | -3.45762800 |
| C | 1.35370900 | -5.22236600 | -2.01527100 |
| H | 1.05033600 | -3.35434000 | -1.00447400 |
| C | 0.79073500 | -6.16562700 | -2.87336400 |
| H | -0.89860500 | -6.76530700 | -4.06709200 |
| H | 2.35843000 | -5.37858800 | -1.63192900 |
| La | -0.14958200 | -0.37375500 | 0.56564400 |
| H | -3.64674100 | -4.30734100 | 1.01730400 |
| H | -2.48693300 | -2.65821300 | -2.25655500 |
| C | -4.29672300 | -2.15029700 | 0.97708400 |
| H | -4.63727400 | -1.74769700 | 1.94204800 |
| H | -1.69999500 | -5.15628500 | 1.63985600 |
| H | -0.34053200 | -4.05388500 | 1.26104500 |
| F | 1.51307200 | -7.27197500 | -3.21191500 |
| H | -5.17139000 | -2.49261100 | 0.41947500 |
| H | -3.80868700 | -1.32787400 | 0.44554200 |
| H | -0.94078200 | -4.18048500 | 2.93894300 |

Energies (0K) = -3609.5816331
Energies (0K) + ZPE = -3609.291669
Enthalpies (298K) = -3609.247311
Free Energies (298K) = -3609.377333

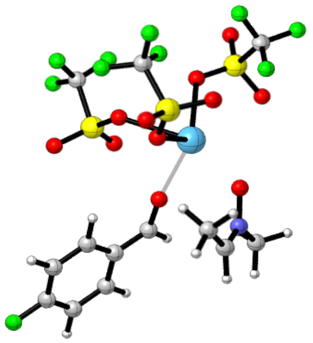

**B1′**
Number of imaginary frequencies = 0
-1 2

| | | | |
|---|---|---|---|
| C | -0.62909600 | -4.56747000 | 1.57181200 |

| | | | |
|---|---|---|---|
| C | -2.85018300 | -3.58619000 | 1.56523700 |
| N | -1.57432400 | -3.45500000 | 1.73897100 |
| O | -1.03029300 | -2.31803900 | 2.12749500 |
| O | 5.02218200 | 1.17757500 | 1.49718400 |
| S | 3.77655400 | 0.42914500 | 1.72417300 |
| O | 2.69408700 | 0.72529900 | 0.71975100 |
| O | 3.90663300 | -1.00549200 | 2.05272000 |
| C | 3.06054100 | 1.16860600 | 3.27099400 |
| F | 3.87524000 | 0.98087600 | 4.31619000 |
| F | 2.84386900 | 2.48299500 | 3.12637300 |
| F | 1.87468300 | 0.58230700 | 3.55374700 |
| O | -3.93612800 | 1.92223500 | 1.63085300 |
| S | -2.51325600 | 1.59127000 | 1.77726700 |
| O | -2.00803100 | 1.31122900 | 3.13415100 |
| O | -2.01921800 | 0.59772500 | 0.74691900 |
| C | -1.59716700 | 3.12561300 | 1.26614400 |
| F | -1.93315400 | 3.49813200 | 0.02595600 |
| F | -0.26869500 | 2.88934000 | 1.29313100 |
| F | -1.86132400 | 4.13419500 | 2.10486300 |
| O | 0.31800900 | 0.92496100 | -1.37400900 |
| S | 0.90226500 | 0.67145800 | -2.74317900 |
| O | 1.86125800 | -0.44762400 | -2.77634200 |
| O | -0.08889500 | 0.73793100 | -3.82872300 |
| C | 1.94348400 | 2.19136300 | -2.98497200 |
| F | 1.18301400 | 3.29564000 | -2.93757300 |
| F | 2.54479100 | 2.14093000 | -4.18442700 |
| F | 2.88897200 | 2.28740100 | -2.04225600 |
| O | -0.69933500 | -2.02841000 | -0.89479700 |
| C | -0.72213400 | -3.01377200 | -1.76378900 |
| C | -1.68509700 | -3.13713800 | -2.79735500 |
| C | -1.65680200 | -4.25909700 | -3.68274900 |
| C | -2.71274200 | -2.16456700 | -3.00569500 |
| C | -2.58520900 | -4.40191300 | -4.70269100 |
| H | -0.88646500 | -5.01608100 | -3.55171800 |
| C | -3.64259000 | -2.30840000 | -4.02823800 |
| H | -2.75534000 | -1.29766700 | -2.35401000 |
| C | -3.56948500 | -3.42252300 | -4.86410400 |
| H | -2.56024700 | -5.25501100 | -5.37481300 |
| H | -4.42116100 | -1.56784700 | -4.18970900 |
| La | 0.25217100 | -0.59332800 | 0.68808500 |
| H | -3.19159100 | -4.57296700 | 1.27522600 |
| H | 0.04965200 | -3.78809800 | -1.67976000 |
| C | -3.79007600 | -2.45702400 | 1.77614100 |
| H | -3.76908200 | -2.13287600 | 2.82559700 |

| | | | |
|---|---|---|---|
| H | -1.15914400 | -5.46905600 | 1.26437300 |
| H | 0.10235900 | -4.27774900 | 0.81545000 |
| F | -4.48216600 | -3.56191300 | -5.86866500 |
| H | -4.80742100 | -2.76008600 | 1.51989800 |
| H | -3.50392100 | -1.58858800 | 1.17214900 |
| H | -0.12659000 | -4.72652000 | 2.52890800 |

Energies (0K) = -3609.5812988

Energies (0K) + ZPE = -3609.29067

Enthalpies (298K) = -3609.246834

Free Energies (298K) = -3609.373379

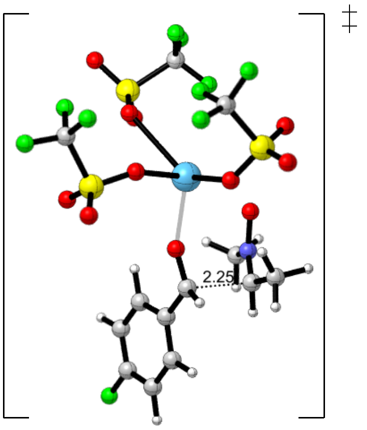


**TS$_{B1}$**

Number of imaginary frequencies = 1

-1 2

| | | | |
|---|---|---|---|
| C | -5.44052500 | -1.02502800 | 1.34189000 |
| N | -4.48603500 | -0.83113600 | 2.25858800 |
| O | -4.21153300 | 0.39550600 | 2.72933300 |
| O | 2.10772400 | 3.66011300 | 2.34873900 |
| S | 0.85339800 | 2.91320200 | 2.53203100 |
| O | -0.18951200 | 3.20120600 | 1.48784000 |
| O | 0.97244300 | 1.48068800 | 2.87509200 |
| C | 0.08726800 | 3.66244900 | 4.05090600 |
| F | 0.86798300 | 3.48270400 | 5.12351200 |
| F | -0.12411000 | 4.97631100 | 3.89058500 |
| F | -1.10768300 | 3.08020600 | 4.30312000 |
| O | -6.83636900 | 4.69386100 | 1.59075000 |
| S | -5.46926200 | 4.30215500 | 1.96159500 |
| O | -5.18436500 | 4.10315700 | 3.39576300 |
| O | -4.89547600 | 3.21824300 | 1.08094900 |
| C | -4.40993500 | 5.75895200 | 1.50544700 |
| F | -4.53664000 | 6.06896000 | 0.21005500 |
| F | -3.11426800 | 5.47489400 | 1.74929500 |
| F | -4.74848300 | 6.83028800 | 2.23563500 |

| | | | |
|---|---|---|---|
| O | -2.47108500 | 3.40054400 | -0.67233500 |
| S | -1.81520400 | 3.31391400 | -2.02551700 |
| O | -0.77914000 | 2.26922100 | -2.12776000 |
| O | -2.76632500 | 3.41889800 | -3.14512600 |
| C | -0.87662300 | 4.91683000 | -2.07184200 |
| F | -1.70947600 | 5.95304000 | -1.88433000 |
| F | -0.27680600 | 5.06524500 | -3.26413300 |
| F | 0.06380800 | 4.95390600 | -1.11978500 |
| O | -3.60563300 | 0.45159200 | -0.19892300 |
| C | -4.38293100 | -0.52763200 | -0.57563000 |
| C | -3.81538400 | -1.80377600 | -1.02576000 |
| C | -4.63016000 | -2.74773800 | -1.68767200 |
| C | -2.45774900 | -2.12319600 | -0.81401400 |
| C | -4.11586600 | -3.96683500 | -2.11956500 |
| H | -5.67627300 | -2.51422000 | -1.87146500 |
| C | -1.93039700 | -3.34023300 | -1.24123200 |
| H | -1.81334900 | -1.40271100 | -0.32004200 |
| C | -2.77045000 | -4.24232100 | -1.88616000 |
| H | -4.73449800 | -4.69434900 | -2.63603600 |
| H | -0.88523700 | -3.59168500 | -1.08773400 |
| La | -2.66784500 | 1.89866100 | 1.41791300 |
| H | -5.64710600 | -2.07082200 | 1.14173100 |
| H | -5.34077500 | -0.25782800 | -1.03569700 |
| F | -2.26133000 | -5.42781100 | -2.30821800 |
| C | -3.57426600 | -1.87777400 | 2.69917000 |
| H | -2.55589600 | -1.64935000 | 2.36683300 |
| H | -3.58842000 | -1.91406400 | 3.79361300 |
| H | -3.88765800 | -2.84163900 | 2.29531600 |
| C | -6.56489300 | -0.03666300 | 1.22826200 |
| H | -7.24997700 | -0.34599000 | 0.43416600 |
| H | -7.13035700 | -0.00085100 | 2.17096000 |
| H | -6.20802000 | 0.97445500 | 1.01818900 |

Energies (0K) = -3609.5728815

Energies (0K) + ZPE = -3609.282154

Enthalpies (298K) = -3609.239414

Free Energies (298K) = -3609.363146

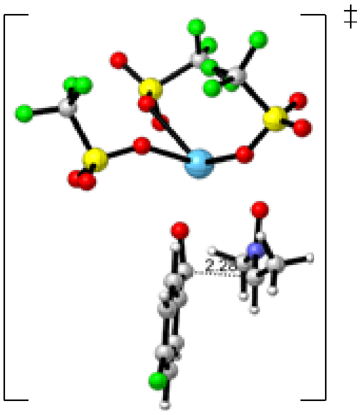

**TS$_{B1'}$**

Number of imaginary frequencies = 1

-1 2

| | | | |
|---|---|---|---|
| C | -3.34654700 | -2.19545300 | 2.19809400 |
| C | -5.33372300 | -1.35219200 | 1.03571800 |
| N | -4.36130900 | -1.18529300 | 1.93533100 |
| O | -4.10493400 | 0.02349700 | 2.46021700 |
| O | 2.01322600 | 3.51181700 | 2.11678500 |
| S | 0.76047700 | 2.77158700 | 2.33290600 |
| O | -0.31047400 | 3.07176500 | 1.31861400 |
| O | 0.87971600 | 1.33678400 | 2.66571900 |
| C | 0.03742100 | 3.51816000 | 3.87361200 |
| F | 0.83944400 | 3.31949700 | 4.92681400 |
| F | -0.16061000 | 4.83560000 | 3.72828700 |
| F | -1.15867200 | 2.94763700 | 4.14530800 |
| O | -6.99287900 | 4.30972800 | 2.09193500 |
| S | -5.55966900 | 4.03551000 | 2.25812500 |
| O | -5.04237600 | 3.90768000 | 3.63337800 |
| O | -5.04357300 | 2.95983300 | 1.32891900 |
| C | -4.69761800 | 5.53898300 | 1.58838300 |
| F | -5.05855400 | 5.77489500 | 0.32135800 |
| F | -3.36251000 | 5.35486900 | 1.62209100 |
| F | -4.99241600 | 6.61643700 | 2.32657100 |
| O | -2.67134800 | 3.29495200 | -0.78486700 |
| S | -2.04630800 | 3.14667600 | -2.14967200 |
| O | -1.02436500 | 2.08718700 | -2.22721900 |
| O | -3.01728900 | 3.22252800 | -3.25370700 |
| C | -1.08734600 | 4.73256400 | -2.27859400 |
| F | -1.90610400 | 5.78861700 | -2.15078400 |
| F | -0.48416000 | 4.80800100 | -3.47599800 |
| F | -0.14725500 | 4.80877800 | -1.32819800 |
| O | -3.69048100 | 0.40580200 | -0.44826000 |
| C | -4.16171000 | -0.74978000 | -0.82832700 |
| C | -5.13473700 | -0.84505500 | -1.91333100 |
| C | -5.46158500 | -2.10359300 | -2.46691400 |
| C | -5.76687000 | 0.30399300 | -2.43850400 |

| | | | |
|---|---|---|---|
| C | -6.38428500 | -2.21710100 | -3.50201100 |
| H | -4.97745500 | -2.99817900 | -2.08203400 |
| C | -6.69045000 | 0.20190600 | -3.47628600 |
| H | -5.51396600 | 1.27758200 | -2.03223900 |
| C | -6.98417800 | -1.05766800 | -3.98978600 |
| H | -6.63570000 | -3.17942800 | -3.93743500 |
| H | -7.17466100 | 1.08004500 | -3.89302200 |
| La | -2.76919300 | 1.76805400 | 1.27916600 |
| H | -5.50319900 | -2.38673100 | 0.75694000 |
| H | -3.52740600 | -1.63376200 | -0.68946000 |
| C | -6.47440400 | -0.38575500 | 0.98641600 |
| H | -6.99868100 | -0.37216700 | 1.95302500 |
| H | -3.67597100 | -3.16374300 | 1.81690800 |
| H | -2.40365400 | -1.90841200 | 1.71562400 |
| F | -7.88025500 | -1.16119700 | -5.00516600 |
| H | -7.18694800 | -0.69168800 | 0.21715800 |
| H | -6.14015500 | 0.63425200 | 0.78070400 |
| H | -3.18705400 | -2.25713400 | 3.27848100 |

Energies (0K) = -3609.5736642

Energies (0K) + ZPE = -3609.282913

Enthalpies (298K) = -3609.240224

Free Energies (298K) = -3609.363248

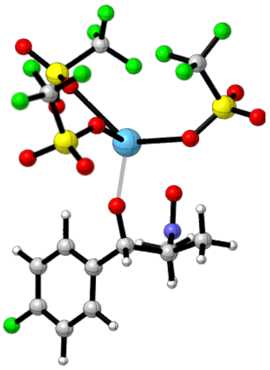

**C1**

Number of imaginary frequencies = 1

-1 2

| | | | |
|---|---|---|---|
| C | -1.69172100 | -4.52374300 | 1.71921300 |
| C | -2.71456700 | -3.13062100 | -0.19043900 |
| N | -2.03044100 | -3.24788900 | 1.10215000 |
| O | -1.54308000 | -2.18809200 | 1.66211200 |
| O | 4.80057500 | 1.07607100 | 1.17489800 |
| S | 3.50091500 | 0.44606600 | 1.44890900 |
| O | 2.39771900 | 0.88625800 | 0.51966600 |
| O | 3.49652800 | -1.00746100 | 1.71764700 |
| C | 2.94487600 | 1.19048800 | 3.05817000 |

| | | | |
|---|---|---|---|
| F | 3.78678200 | 0.87319900 | 4.04880500 |
| F | 2.86672400 | 2.52511000 | 2.97216200 |
| F | 1.71808000 | 0.72238200 | 3.38396500 |
| O | -4.25796100 | 2.36901700 | 1.33803700 |
| S | -2.86101600 | 1.99346600 | 1.59144500 |
| O | -2.46385600 | 1.74390000 | 2.98943500 |
| O | -2.33905600 | 0.94956600 | 0.62841300 |
| C | -1.86379000 | 3.48401300 | 1.09951600 |
| F | -2.12017300 | 3.84131600 | -0.16398000 |
| F | -0.54664000 | 3.21040000 | 1.20311600 |
| F | -2.14134800 | 4.51774000 | 1.90341800 |
| O | 0.03069600 | 1.31067900 | -1.44366200 |
| S | 0.20375600 | 1.27883300 | -2.94160500 |
| O | 1.25378300 | 0.35482300 | -3.40486100 |
| O | -1.06609100 | 1.28326000 | -3.68638900 |
| C | 0.91232600 | 2.97183200 | -3.22702300 |
| F | 0.06484400 | 3.91818400 | -2.79639700 |
| F | 1.13285700 | 3.16353800 | -4.53670200 |
| F | 2.07672400 | 3.11824700 | -2.57695300 |
| O | -0.97163600 | -1.64250100 | -1.05662400 |
| C | -1.67481100 | -2.79077500 | -1.33054000 |
| C | -0.77703900 | -4.00225000 | -1.61548300 |
| C | -1.31420300 | -5.15031400 | -2.21972200 |
| C | 0.58194000 | -3.99931400 | -1.28313000 |
| C | -0.52633300 | -6.27331700 | -2.47284100 |
| H | -2.36445400 | -5.16869000 | -2.50288300 |
| C | 1.39132100 | -5.11343300 | -1.52822900 |
| H | 1.01754200 | -3.10866000 | -0.84144900 |
| C | 0.81757600 | -6.23233000 | -2.11626600 |
| H | -0.93634100 | -7.16271500 | -2.94153400 |
| H | 2.44720000 | -5.11604700 | -1.27498100 |
| La | -0.08084700 | -0.25627100 | 0.53692000 |
| H | -3.13826400 | -4.11708000 | -0.39138000 |
| H | -2.31319800 | -2.65534300 | -2.22440700 |
| C | -3.84434600 | -2.10215000 | -0.11501400 |
| H | -4.54821400 | -2.36373300 | 0.68320700 |
| H | -2.28336300 | -5.31922700 | 1.26481200 |
| H | -0.62409400 | -4.73478600 | 1.58173000 |
| F | 1.59331000 | -7.31997400 | -2.35982400 |
| H | -4.38981900 | -2.10365200 | -1.06504500 |
| H | -3.46597500 | -1.09464900 | 0.06683400 |
| H | -1.90854100 | -4.46290600 | 2.79012200 |

Energies (0K) = -3609.5973772

Energies (0K) + ZPE = -3609.303168

Enthalpies (298K) = -3609.260795
Free Energies (298K) = -3609.383264

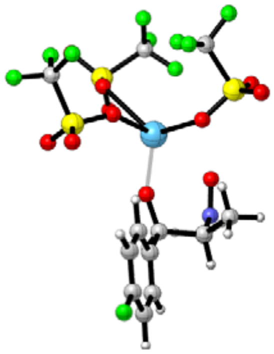

**C1′**
Number of imaginary frequencies = 1
-1 2

| | | | |
|---|---|---|---|
| C | -3.70187800 | -2.32896200 | 2.51558200 |
| C | -5.28264400 | -1.17089500 | 0.88027000 |
| N | -4.44836500 | -1.15116100 | 2.08988000 |
| O | -4.06119200 | -0.01256000 | 2.57170600 |
| O | 2.09266000 | 3.40366600 | 1.87047500 |
| S | 0.83033400 | 2.69782400 | 2.13522800 |
| O | -0.25333500 | 2.98919100 | 1.12862200 |
| O | 0.92094900 | 1.27214100 | 2.50957900 |
| C | 0.14833300 | 3.51059700 | 3.66191400 |
| F | 0.96768100 | 3.33211600 | 4.70463900 |
| F | -0.02641400 | 4.82518500 | 3.47175300 |
| F | -1.05308500 | 2.97347800 | 3.97576800 |
| O | -6.94558200 | 4.25265600 | 2.16169200 |
| S | -5.52456900 | 3.91893500 | 2.32299300 |
| O | -5.05612800 | 3.56378100 | 3.67544100 |
| O | -4.99957800 | 2.99464800 | 1.24638400 |
| C | -4.60759600 | 5.48539500 | 1.92542400 |
| F | -4.96629700 | 5.96227700 | 0.72787400 |
| F | -3.28008500 | 5.24114900 | 1.90203900 |
| F | -4.84609000 | 6.42184000 | 2.85066300 |
| O | -2.83977900 | 3.31478200 | -0.90703300 |
| S | -2.29774600 | 3.24653800 | -2.31543300 |
| O | -1.18688300 | 2.29223800 | -2.48007000 |
| O | -3.35000100 | 3.23642300 | -3.34415700 |
| C | -1.51288600 | 4.92371500 | -2.47299500 |
| F | -2.42781000 | 5.88861700 | -2.29878000 |
| F | -0.97416500 | 5.06259800 | -3.69387900 |
| F | -0.54413600 | 5.08901000 | -1.56179200 |
| O | -3.65002900 | 0.26502800 | -0.24017200 |

| | | | |
|---|---|---|---|
| C | -4.34466500 | -0.91674700 | -0.37020200 |
| C | -5.16792300 | -0.98630900 | -1.65871900 |
| C | -5.68255300 | -2.20954300 | -2.11449000 |
| C | -5.41741100 | 0.16938400 | -2.40900400 |
| C | -6.43878500 | -2.28322600 | -3.28566500 |
| H | -5.48673200 | -3.12179100 | -1.55518000 |
| C | -6.16988800 | 0.11596700 | -3.58540900 |
| H | -4.99948500 | 1.11337600 | -2.07690100 |
| C | -6.66972400 | -1.11191500 | -3.99876500 |
| H | -6.83766900 | -3.22570500 | -3.64804100 |
| H | -6.36215800 | 1.00628700 | -4.17661800 |
| La | -2.71674700 | 1.81843400 | 1.14196900 |
| H | -5.66962600 | -2.19110400 | 0.80801100 |
| H | -3.65129600 | -1.77767900 | -0.38769800 |
| C | -6.44218200 | -0.18618000 | 1.01149700 |
| H | -6.99394300 | -0.37116000 | 1.93992900 |
| H | -4.25898500 | -3.23065500 | 2.25414700 |
| H | -2.71842500 | -2.34996600 | 2.02669300 |
| F | -7.40004600 | -1.17464800 | -5.14249600 |
| H | -7.13251900 | -0.32725200 | 0.17497500 |
| H | -6.10131100 | 0.85058200 | 1.01089600 |
| H | -3.56081500 | -2.27507400 | 3.59783000 |

Energies (0K) = -3609.5973621

Energies (0K) + ZPE = -3609.303574

Enthalpies (298K) = -3609.260824

Free Energies (298K) = -3609.385282

## Single-Crystal X-Ray Diffraction Analysis of 6a-9•HCl, 6b-3•HCl and 6k-3•HCl:

***Supplementary Table 5***. Crystal data and structure refinement for **6a-9•HCl**

| | | |
|---|---|---|
| Identification code | **6a-9•HCl** | |
| CCDC | 1537335 | |
| Empirical formula | $C_{19}H_{26}ClNOS$ | |
| Formula weight | 351.92 | |
| Temperature | 173.00(10) | |
| Wavelength | 1.54184 Å | |
| Crystal system, space group | Monoclinic, $P2_1$ | |
| Unit cell dimensions | a = 7.67810(10) Å | alpha = 90 deg. |
| | b = 7.98550(10) Å | beta = 91.5140(10) deg. |
| | c = 15.5357(2) Å | gamma = 90 deg. |
| Volume | 952.22(2) Å ^3 | |
| Z, Calculated density | 2, 1.227 g/cm^3 | |
| Absorption coefficient | 2.817 mm^-1 | |
| F (000) | 376.0 | |

| | |
|---|---|
| Crystal size | 0.3 × 0.1 × 0.05 mm |
| 2 Theta range for data collection | 11.396 to 153.66 deg. |
| Limiting indices | $-9 \le h \le 9$, $-10 \le k \le 10$, $-19 \le l \le 19$ |
| Reflections collected / unique | 15866 / 3949 [$R_{int}$ = 0.0589, $R_{sigma}$ = 0.0498] |
| Completeness to theta = 67.684 | 0.999 |
| Absorption correction | multi-scan |
| Max. and min. transmission | 1.00000 and 0.36743 |
| Refinement method | Full-matrix least-squares on F^2 |
| Data / restraints / parameters | 3949/1/212 |
| Goodness-of-fit on F^2 | 1.102 |
| Final R indices [I>2sigma(I)] | $R_1$ = 0.0366, $wR_2$ = 0.0884 |
| R indices (all data) | $R_1$ = 0.0423, $wR_2$ = 0.0905 |
| Absolute structure parameter | 0.007(11) |
| Largest diff. peak and hole | 0.32 and -0.32 e.Å^-3 |

***Supplementary Table 6.*** Fractional atomic coordinates (×$10^4$) and equivalent isotropic displacement parameters (Å$^2$×$10^3$) for **6a-9•HCl**. $U_{eq}$ is defined as 1/3 of the trace of the orthogonalized $U_{IJ}$ tensor

| Atom | x | y | z | U(eq) |
|---|---|---|---|---|
| Cl(1) | 10599.7(14) | 4558.6(10) | -519.7(5) | 44.3(2) |
| S(1) | 3036.4(11) | 1351.1(11) | 4597.5(5) | 36.6(2) |
| O(1) | 9498(3) | 2634(3) | 1751.1(14) | 31.4(5) |
| N(1) | 9192(3) | 5860(3) | 1182.7(14) | 23.5(5) |
| C(8) | 6157(4) | 2436(4) | 3924(2) | 32.6(7) |
| C(6) | 6632(4) | 2578(4) | 2385.9(19) | 25.0(6) |
| C(14) | 10839(4) | 6548(4) | 2551.6(18) | 27.8(6) |
| C(9) | 4522(4) | 1781(4) | 3774.6(19) | 28.0(6) |
| C(1) | 7739(4) | 3190(4) | 1656.7(18) | 24.1(6) |
| C(2) | 7711(4) | 5120(3) | 1667.5(17) | 20.9(6) |
| C(13) | 10955(4) | 5962(4) | 1633(2) | 30.5(7) |
| C(19) | 10212(4) | 8125(4) | 2746(2) | 30.7(7) |
| C(3) | 5957(4) | 5833(4) | 1327.4(19) | 27.3(6) |
| C(7) | 7206(4) | 2822(4) | 3236(2) | 32.2(7) |
| C(11) | 5001(5) | 1872(4) | 2246.2(19) | 31.7(7) |
| C(10) | 3942(4) | 1472(4) | 2926.1(19) | 33.0(7) |
| C(17) | 10571(5) | 7557(5) | 4258(2) | 38.8(8) |
| C(4) | 5631(5) | 7598(4) | 1675(2) | 36.6(7) |
| C(16) | 11204(5) | 5992(5) | 4072(2) | 40.5(9) |
| C(5) | 5762(5) | 5791(5) | 342(2) | 43.8(9) |
| C(18) | 10063(5) | 8629(5) | 3600(2) | 37.9(8) |
| C(15) | 11336(5) | 5481(4) | 3221(2) | 36.4(8) |
| C(12) | 4122(6) | 2098(6) | 5563(2) | 47.0(9) |

***Supplementary Table 7***. Bond lengths for **6a-9•HCl**

| Atom | Atom | Length/Å |
|---|---|---|
| S(1) | C(9) | 1.769(3) |
| S(1) | C(12) | 1.798(4) |
| O(1) | C(1) | 1.426(4) |
| N(1) | C(2) | 1.502(4) |
| N(1) | C(13) | 1.510(4) |
| C(8) | C(9) | 1.374(5) |
| C(8) | C(7) | 1.390(5) |
| C(6) | C(1) | 1.515(4) |
| C(6) | C(7) | 1.395(4) |
| C(6) | C(11) | 1.385(5) |
| C(14) | C(13) | 1.507(4) |
| C(14) | C(19) | 1.384(5) |
| C(14) | C(15) | 1.390(4) |
| C(9) | C(10) | 1.402(4) |
| C(1) | C(2) | 1.542(4) |
| C(2) | C(3) | 1.542(4) |
| C(19) | C(18) | 1.394(4) |
| C(3) | C(4) | 1.532(5) |
| C(3) | C(5) | 1.534(4) |
| C(11) | C(10) | 1.387(5) |
| C(17) | C(16) | 1.375(6) |
| C(17) | C(18) | 1.382(5) |
| C(16) | C(15) | 1.391(5) |

***Supplementary Table 8.*** Bond angles for **6a-9•HCl**

| Atom | Atom | Atom | Angle/deg. |
|---|---|---|---|
| C(9) | S(1) | C(12) | 104.17(17) |
| C(2) | N(1) | C(13) | 118.1(2) |
| C(9) | C(8) | C(7) | 119.9(3) |
| C(7) | C(6) | C(1) | 119.6(3) |
| C(11) | C(6) | C(1) | 122.5(3) |
| C(11) | C(6) | C(7) | 117.8(3) |
| C(19) | C(14) | C(13) | 121.3(3) |
| C(19) | C(14) | C(15) | 119.0(3) |
| C(15) | C(14) | C(13) | 119.7(3) |
| C(8) | C(9) | S(1) | 123.7(2) |
| C(8) | C(9) | C(10) | 119.5(3) |
| C(10) | C(9) | S(1) | 116.8(3) |
| O(1) | C(1) | C(6) | 111.8(2) |
| O(1) | C(1) | C(2) | 108.9(2) |
| C(6) | C(1) | C(2) | 107.8(2) |

| | | | |
|---|---|---|---|
| N(1) | C(2) | C(1) | 112.2(2) |
| N(1) | C(2) | C(3) | 110.4(2) |
| C(1) | C(2) | C(3) | 112.2(2) |
| C(14) | C(13) | N(1) | 112.4(2) |
| C(14) | C(19) | C(18) | 120.5(3) |
| C(4) | C(3) | C(2) | 111.6(2) |
| C(4) | C(3) | C(5) | 111.0(3) |
| C(5) | C(3) | C(2) | 113.3(3) |
| C(8) | C(7) | C(6) | 121.5(3) |
| C(6) | C(11) | C(10) | 121.3(3) |
| C(11) | C(10) | C(9) | 119.8(3) |
| C(16) | C(17) | C(18) | 120.1(3) |
| C(17) | C(16) | C(15) | 120.1(3) |
| C(17) | C(18) | C(19) | 119.8(3) |
| C(14) | C(15) | C(16) | 120.4(3) |

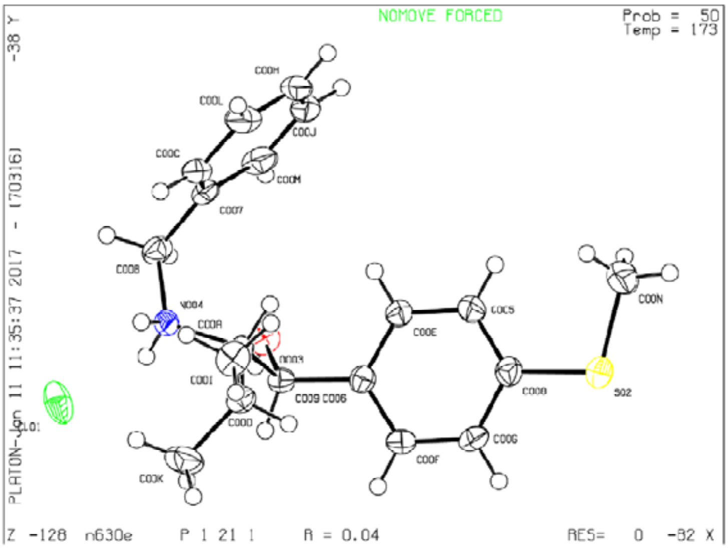


***Supplementary Table 9***. Crystal data and structure refinement for **6b-3•HCl•$H_2O$**

| | | |
|---|---|---|
| Identification code | **6b-3•HCl** | |
| CCDC | 1537337 | |
| Empirical formula | $C_{18}H_{26.33}ClNO_{2.17}S$ | |
| Formula weight | 358.88 | |
| Temperature | 173.00(10) | |
| Wavelength | 1.54184 Å | |
| Crystal system, space group | Monoclinic, $P2_1$ | |
| Unit cell dimensions | a = 9.9390(3) Å | alpha = 90 deg. |
| | b = 15.6514(3) Å | beta = 96.850(2) deg. |
| | c = 12.5115(2) Å | gamma = 90 deg. |

| | |
|---|---|
| Volume | 1932.39(8) Å ^3 |
| Z, Calculated density | 4, 1.234 g/cm^3 |
| Absorption coefficient | 2.831 mm^-1 |
| F (000) | 767.0 |
| Crystal size | 0.1 × 0.08 × 0.02 mm |
| 2 Theta range for data collection | 7.116 to 129.998 deg. |
| Limiting indices | $-11 \le h \le 11$, $-18 \le k \le 18$, $-14 \le l \le 14$ |
| Reflections collected / unique | 30230 / 6450 [$R_{int}$ = 0.0866, $R_{sigma}$ = 0.0645] |
| Completeness to theta = 64.999 | 0.987 |
| Absorption correction | multi-scan |
| Max. and min. transmission | 1.00000 and 0.47199 |
| Refinement method | Full-matrix least-squares on F^2 |
| Data / restraints / parameters | 6450/7/451 |
| Goodness-of-fit on F^2 | 1.051 |
| Final R indices [I>2sigma(I)] | $R_1$ = 0.0554, $wR_2$ = 0.1215 |
| R indices (all data) | $R_1$ = 0.0623, $wR_2$ = 0.1247 |
| Absolute structure parameter | 0.010(13) |
| Largest diff. peak and hole | 0.35 and -0.22 e.Å^-3 |

***Supplementary Table 10.*** Fractional atomic coordinates ($\times 10^4$) and equivalent isotropic displacement parameters ($Å^2 \times 10^3$) for **6b-3•HCl•H₂O**. $U_{eq}$ is defined as 1/3 of the trace of the orthogonalized $U_{IJ}$ tensor

| Atom | x | y | z | U(eq) |
|---|---|---|---|---|
| Cl(2) | -5599.9(14) | 4091.2(7) | 4412.2(9) | 43.6(3) |
| Cl(1) | -9969.7(14) | 901.4(6) | 5532.2(9) | 45.0(3) |
| S(003) | -10431.3(15) | 1004.9(9) | -1029.8(9) | 49.3(3) |
| S(004) | -4800.8(16) | 3969.2(8) | 10964.8(10) | 50.4(3) |
| O(21) | -6233(4) | 2551(2) | 5844(2) | 38.6(7) |
| O(11) | -11292(4) | 2281.8(19) | 3974(3) | 38.1(7) |
| N(21) | -4937(4) | 1121(2) | 5332(3) | 31.3(8) |
| O(1) | -2687(4) | 3828(3) | 5565(3) | 55.7(10) |
| C(113) | -9379(5) | 3749(3) | 6543(4) | 36.7(10) |
| C(209) | -3756(6) | 3363(3) | 9226(4) | 44.5(12) |
| C(208) | -4950(5) | 3467(3) | 9688(4) | 36.8(11) |
| N(11) | -10265(4) | 3847(2) | 4616(3) | 31.3(8) |
| C(206) | -6177(5) | 2846(3) | 8099(4) | 34.3(10) |
| C(205) | -4982(5) | 2752(3) | 7634(4) | 31.6(9) |
| C(202) | -4788(5) | 1405(3) | 6506(3) | 30.5(9) |
| C(101) | -10218(5) | 2595(3) | 3427(3) | 29.4(9) |
| C(105) | -10270(5) | 2230(3) | 2298(3) | 32.2(10) |
| C(204) | -3387(5) | 1156(3) | 7029(4) | 39.8(11) |
| C(203) | -5900(5) | 929(3) | 7002(3) | 36.5(10) |

| | | | | |
|---|---|---|---|---|
| C(207) | -6158(6) | 3199(3) | 9122(4) | 38.6(11) |
| C(108) | -10291(5) | 1506(3) | 243(4) | 36.2(10) |
| C(201) | -5002(5) | 2386(3) | 6506(3) | 31.0(9) |
| C(102) | -10309(5) | 3584(3) | 3444(3) | 30.9(9) |
| C(110) | -11481(5) | 2066(3) | 1658(4) | 36.6(10) |
| C(112) | -9109(6) | 3543(3) | 5406(4) | 42.6(11) |
| C(210) | -3773(5) | 3016(3) | 8201(4) | 40.3(11) |
| C(218) | -4914(6) | 1887(3) | 2761(4) | 41.3(12) |
| C(103) | -9114(5) | 3992(3) | 2978(3) | 37.5(10) |
| C(107) | -9072(5) | 1675(3) | 866(4) | 38(1) |
| C(213) | -4378(5) | 1276(3) | 3469(3) | 35.4(10) |
| C(217) | -5270(6) | 1682(4) | 1676(4) | 49.6(13) |
| C(106) | -9068(5) | 2038(3) | 1888(4) | 35.8(10) |
| C(114) | -9854(6) | 3125(3) | 7185(4) | 43.1(12) |
| C(118) | -9135(7) | 4558(3) | 6961(4) | 53.1(15) |
| C(214) | -4185(8) | 453(3) | 3095(4) | 57.9(16) |
| C(109) | -11503(5) | 1713(3) | 650(4) | 38.9(11) |
| C(212) | -3945(6) | 1486(4) | 4632(4) | 45.6(12) |
| C(115) | -10055(7) | 3308(4) | 8239(5) | 53.0(14) |
| C(216) | -5087(7) | 871(5) | 1325(4) | 61.1(16) |
| C(111) | -8745(6) | 1017(4) | -1373(4) | 53.4(14) |
| O(2B) | -7574(7) | 720(7) | 4071(7) | 53(2) |
| C(104) | -11654(5) | 3905(3) | 2897(4) | 37.4(10) |
| C(116) | -9803(8) | 4118(5) | 8650(4) | 66.6(18) |
| C(117) | -9350(9) | 4738(4) | 7999(5) | 72(2) |
| C(215) | -4555(10) | 263(4) | 2033(5) | 77(2) |
| C(211) | -6494(8) | 4052(5) | 11251(5) | 70.8(19) |
| O(3A) | -2482(15) | 4942(10) | 7300(10) | 71(5) |
| O(2A) | -7560(12) | 1199(11) | 4311(9) | 47(3) |

***Supplementary Table 11.*** Bond lengths for **6b-3•HCl•$H_2$O**

| Atom | Atom | Length/Å |
|---|---|---|
| S(003) | C(108) | 1.765(4) |
| S(003) | C(111) | 1.780(6) |
| S(004) | C(208) | 1.771(5) |
| S(004) | C(211) | 1.766(7) |
| O(21) | C(201) | 1.416(6) |
| O(11) | C(101) | 1.422(5) |
| N(21) | C(202) | 1.524(5) |
| N(21) | C(212) | 1.508(6) |

| | | |
|---|---|---|
| C(113) | C(112) | 1.513(6) |
| C(113) | C(114) | 1.382(8) |
| C(113) | C(118) | 1.380(7) |
| C(209) | C(208) | 1.390(8) |
| C(209) | C(210) | 1.391(7) |
| C(208) | C(207) | 1.384(8) |
| N(11) | C(102) | 1.519(5) |
| N(11) | C(112) | 1.500(6) |
| C(206) | C(205) | 1.392(7) |
| C(206) | C(207) | 1.392(7) |
| C(205) | C(201) | 1.521(6) |
| C(205) | C(210) | 1.384(7) |
| C(202) | C(204) | 1.517(7) |
| C(202) | C(203) | 1.525(6) |
| C(202) | C(201) | 1.549(6) |
| C(101) | C(105) | 1.519(6) |
| C(101) | C(102) | 1.551(5) |
| C(105) | C(110) | 1.388(7) |
| C(105) | C(106) | 1.388(7) |
| C(108) | C(107) | 1.386(7) |
| C(108) | C(109) | 1.400(7) |
| C(102) | C(103) | 1.524(7) |
| C(102) | C(104) | 1.513(7) |
| C(110) | C(109) | 1.375(7) |
| C(218) | C(213) | 1.368(7) |
| C(218) | C(217) | 1.398(8) |
| C(107) | C(106) | 1.399(7) |
| C(213) | C(214) | 1.391(7) |
| C(213) | C(212) | 1.505(6) |
| C(217) | C(216) | 1.362(10) |
| C(114) | C(115) | 1.387(8) |
| C(118) | C(117) | 1.371(8) |
| C(214) | C(215) | 1.369(9) |
| C(115) | C(116) | 1.381(10) |
| C(216) | C(215) | 1.363(10) |
| C(116) | C(117) | 1.377(11) |

***Supplementary Table 12.*** Bond angles for **6b-3•HCl•$H_2$O**

| Atom | Atom | Atom | Angle/deg. |
|---|---|---|---|
| C(108) | S(003) | C(111) | 103.8(3) |
| C(211) | S(004) | C(208) | 103.7(3) |
| C(212) | N(21) | C(202) | 117.0(4) |
| C(114) | C(113) | C(112) | 120.5(4) |

| | | | |
|---|---|---|---|
| C(118) | C(113) | C(112) | 120.5(5) |
| C(118) | C(113) | C(114) | 119.0(5) |
| C(208) | C(209) | C(210) | 120.8(5) |
| C(209) | C(208) | S(004) | 116.3(4) |
| C(207) | C(208) | S(004) | 124.7(4) |
| C(207) | C(208) | C(209) | 118.9(5) |
| C(112) | N(11) | C(102) | 118.9(4) |
| C(205) | C(206) | C(207) | 120.5(5) |
| C(206) | C(205) | C(201) | 120.7(4) |
| C(210) | C(205) | C(206) | 119.1(4) |
| C(210) | C(205) | C(201) | 120.2(4) |
| N(21) | C(202) | C(203) | 104.9(4) |
| N(21) | C(202) | C(201) | 106.9(3) |
| C(204) | C(202) | N(21) | 108.6(4) |
| C(204) | C(202) | C(203) | 111.8(4) |
| C(204) | C(202) | C(201) | 112.0(4) |
| C(203) | C(202) | C(201) | 112.2(4) |
| O(11) | C(101) | C(105) | 111.9(4) |
| O(11) | C(101) | C(102) | 106.8(3) |
| C(105) | C(101) | C(102) | 113.1(3) |
| C(110) | C(105) | C(101) | 122.5(4) |
| C(106) | C(105) | C(101) | 119.3(4) |
| C(106) | C(105) | C(110) | 118.2(4) |
| C(208) | C(207) | C(206) | 120.4(5) |
| C(107) | C(108) | S(003) | 124.2(4) |
| C(107) | C(108) | C(109) | 118.9(4) |
| C(109) | C(108) | S(003) | 116.9(4) |
| O(21) | C(201) | C(205) | 112.8(4) |
| O(21) | C(201) | C(202) | 106.9(4) |
| C(205) | C(201) | C(202) | 112.7(3) |
| N(11) | C(102) | C(101) | 106.7(3) |
| N(11) | C(102) | C(103) | 108.8(4) |
| C(103) | C(102) | C(101) | 111.3(4) |
| C(104) | C(102) | N(11) | 105.6(4) |
| C(104) | C(102) | C(101) | 112.0(4) |
| C(104) | C(102) | C(103) | 112.1(4) |
| C(109) | C(110) | C(105) | 121.4(5) |
| N(11) | C(112) | C(113) | 110.1(4) |
| C(205) | C(210) | C(209) | 120.3(5) |
| C(213) | C(218) | C(217) | 120.1(5) |
| C(108) | C(107) | C(106) | 119.9(5) |
| C(218) | C(213) | C(214) | 119.2(4) |
| C(218) | C(213) | C(212) | 121.2(4) |
| C(214) | C(213) | C(212) | 119.5(5) |

| | | | |
|---|---|---|---|
| C(216) | C(217) | C(218) | 119.9(5) |
| C(105) | C(106) | C(107) | 121.2(5) |
| C(113) | C(114) | C(115) | 120.3(5) |
| C(117) | C(118) | C(113) | 120.5(5) |
| C(215) | C(214) | C(213) | 119.7(5) |
| C(110) | C(109) | C(108) | 120.4(5) |
| C(213) | C(212) | N(21) | 110.3(4) |
| C(116) | C(115) | C(114) | 120.2(5) |
| C(217) | C(216) | C(215) | 119.8(5) |
| C(117) | C(116) | C(115) | 119.0(5) |
| C(118) | C(117) | C(116) | 120.9(6) |
| C(216) | C(215) | C(214) | 121.2(6) |

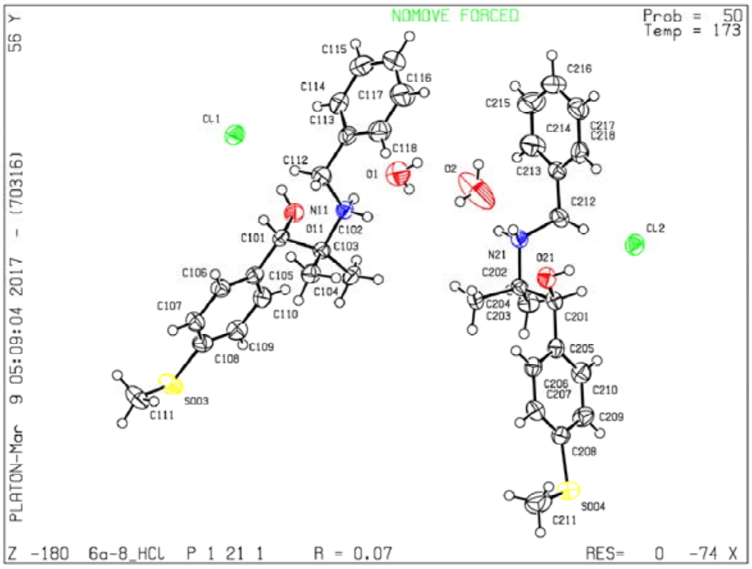


***Supplementary Table 13***. Crystal data and structure refinement for **6k-3•HCl**

| | | |
|---|---|---|
| Identification code | **6k-3•HCl** | |
| CCDC | 1537338 | |
| Empirical formula | $C_{14}H_{22}ClNOS$ | |
| Formula weight | 287.83 | |
| Temperature | 173.00(10) | |
| Wavelength | 1.54184 Å | |
| Crystal system, space group | Orthorhombic, $P2_12_12_1$ | |
| Unit cell dimensions | a = 7.23813(6) Å | alpha = 90 deg. |
| | b = 8.06555(6) Å | beta = 90 deg. |
| | c = 26.48022(19) Å | gamma = 90 deg. |
| Volume | 1545.90(2) Å ^3 | |
| Z, Calculated density | 4, 1.237 g/cm^3 | |
| Absorption coefficient | 3.353 mm^-1 | |

|  |  |
|---|---|
| F (000) | 616.0 |
| Crystal size | 0.3 × 0.11 × 0.08 mm |
| 2 Theta range for data collection | 11.468 to 153.734 deg. |
| Limiting indices | $-9 \leq h \leq 8$, $-10 \leq k \leq 10$, $-33 \leq l \leq 33$ |
| Reflections collected / unique | 27152 / 3247 [$R_{int}$ = 0.0571, $R_{sigma}$ = 0.0292] |
| Completeness to theta = 67.684 | 0.999 |
| Absorption correction | multi-scan |
| Max. and min. transmission | 1.00000 and 0.58671 |
| Refinement method | Full-matrix least-squares on F^2 |
| Data / restraints / parameters | 3247/0/167 |
| Goodness-of-fit on F^2 | 1.023 |
| Final R indices [I>2sigma(I)] | $R_1$ = 0.0284, $wR_2$ = 0.0710 |
| R indices (all data) | $R_1$ = 0.0335, $wR_2$ = 0.0716 |
| Absolute structure parameter | -0.002(6) |
| Largest diff. peak and hole | 0.21/-0.19 e.Å^-3 |

***Supplementary Table 14.*** Fractional atomic coordinates ($\times 10^4$) and equivalent isotropic displacement parameters ($Å^2 \times 10^3$) for **6k-3•HCl**. $U_{eq}$ is defined as 1/3 of the trace of the orthogonalized $U_{IJ}$ tensor

| Atom | x | y | z | U(eq) |
|---|---|---|---|---|
| Cl(1) | -653.8(7) | 1768.0(6) | 5359.8(2) | 32.40(13) |
| S(1) | 8859.6(8) | 7200.1(7) | 7672.5(2) | 38.05(14) |
| O(1) | 3756(2) | 4629.9(17) | 5727.2(6) | 34.4(3) |
| N(1) | 3457(2) | 1151.5(18) | 5625.2(6) | 24.1(3) |
| C(5) | 3712(3) | -712(2) | 5657.4(7) | 26.8(4) |
| C(8) | 6114(3) | 4489(2) | 6357.8(7) | 25.9(4) |
| C(12) | 8863(3) | 5700(2) | 6721.7(7) | 28.7(4) |
| C(13) | 7990(3) | 4845(2) | 6334.0(7) | 28.2(4) |
| C(9) | 5114(3) | 5026(3) | 6776.9(8) | 31.3(4) |
| C(10) | 5975(3) | 5874(3) | 7167.3(8) | 32.1(4) |
| C(4) | 5633(3) | -818(2) | 5889.2(8) | 31.4(4) |
| C(1) | 5188(3) | 3590(2) | 5925.4(7) | 25.9(4) |
| C(11) | 7871(3) | 6193(2) | 7144.2(7) | 27.6(4) |
| C(2) | 4345(3) | 1932(2) | 6083.8(7) | 26.5(4) |
| C(3) | 5741(3) | 614(2) | 6270.2(7) | 32.2(4) |
| C(6) | 2225(3) | -1415(3) | 6004.4(9) | 37.8(5) |
| C(14) | 11294(3) | 7049(3) | 7555.0(9) | 40.2(5) |
| C(7) | 3563(4) | -1421(3) | 5125.4(8) | 37.2(5) |

***Supplementary Table 15.*** Bond lengths for **6k-3•HCl**

| Atom | Atom | Length/Å |
|---|---|---|
| S(1) | C(11) | 1.769(2) |
| S(1) | C(14) | 1.794(3) |

| | | |
|---|---|---|
| O(1) | C(1) | 1.433(2) |
| N(1) | C(5) | 1.517(2) |
| N(1) | C(2) | 1.511(2) |
| C(5) | C(4) | 1.523(3) |
| C(5) | C(6) | 1.524(3) |
| C(5) | C(7) | 1.524(3) |
| C(8) | C(13) | 1.389(3) |
| C(8) | C(9) | 1.394(3) |
| C(8) | C(1) | 1.512(3) |
| C(12) | C(13) | 1.389(3) |
| C(12) | C(11) | 1.388(3) |
| C(9) | C(10) | 1.387(3) |
| C(10) | C(11) | 1.398(3) |
| C(4) | C(3) | 1.535(3) |
| C(1) | C(2) | 1.529(2) |
| C(2) | C(3) | 1.548(3) |

***Supplementary Table 16.*** Bond angles for **6k-3•HCl**

| Atom | Atom | Atom | Angle/deg. |
|---|---|---|---|
| C(11) | S(1) | C(14) | 103.23(10) |
| C(2) | N(1) | C(5) | 108.40(14) |
| N(1) | C(5) | C(4) | 100.93(15) |
| N(1) | C(5) | C(6) | 108.45(16) |
| N(1) | C(5) | C(7) | 108.14(15) |
| C(4) | C(5) | C(6) | 112.38(18) |
| C(4) | C(5) | C(7) | 114.58(18) |
| C(6) | C(5) | C(7) | 111.57(18) |
| C(13) | C(8) | C(9) | 118.66(18) |
| C(13) | C(8) | C(1) | 119.84(18) |
| C(9) | C(8) | C(1) | 121.46(19) |
| C(11) | C(12) | C(13) | 120.2(2) |
| C(8) | C(13) | C(12) | 120.91(19) |
| C(10) | C(9) | C(8) | 120.9(2) |
| C(9) | C(10) | C(11) | 119.97(19) |
| C(5) | C(4) | C(3) | 105.61(16) |
| O(1) | C(1) | C(8) | 108.51(15) |
| O(1) | C(1) | C(2) | 108.87(16) |
| C(8) | C(1) | C(2) | 112.87(15) |
| C(12) | C(11) | S(1) | 124.05(17) |
| C(12) | C(11) | C(10) | 119.38(18) |
| C(10) | C(11) | S(1) | 116.57(15) |
| N(1) | C(2) | C(1) | 108.27(14) |
| N(1) | C(2) | C(3) | 104.36(14) |

| | | | |
|---|---|---|---|
| C(1) | C(2) | C(3) | 115.32(17) |
| C(4) | C(3) | C(2) | 105.89(16) |

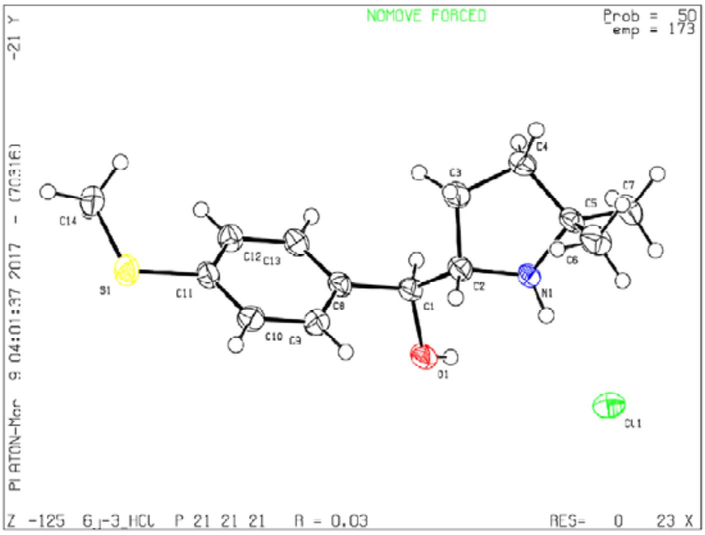

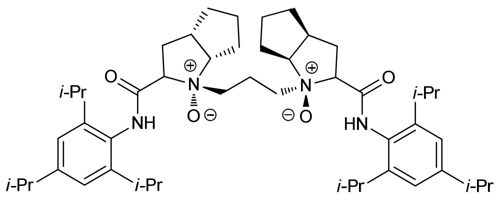


**L1-b**

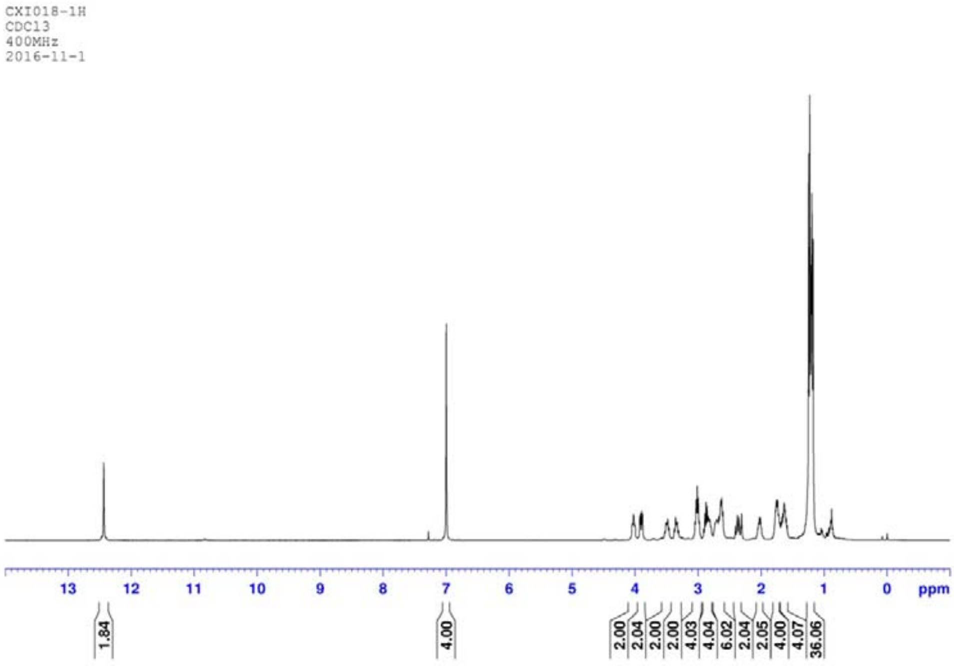


***Supplementary Figure 12.*** $^{1}$H NMR ($CDCl_3$) of compound **L1-b**.

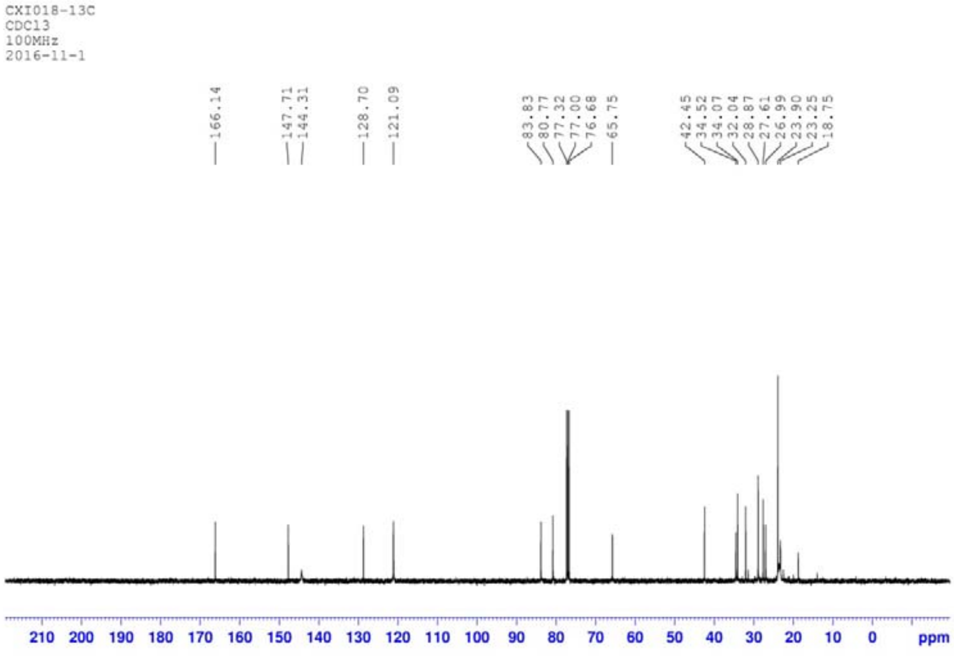


***Supplementary Figure 13.*** $^{13}$C NMR ($CDCl_3$) of compound **L1-b**.

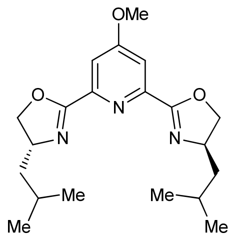


**L2-b**

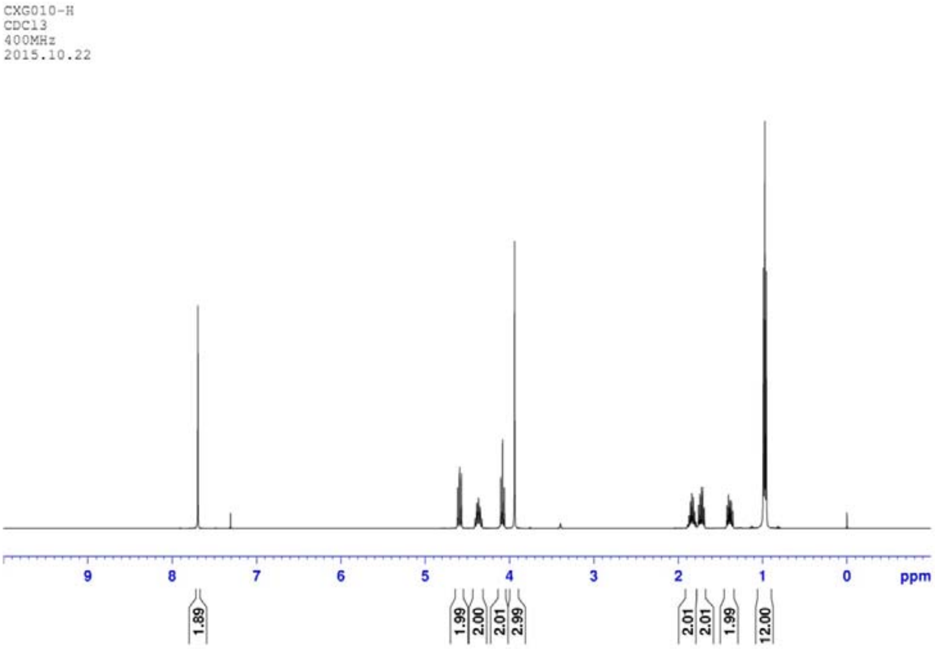


***Supplementary Figure 14.*** $^{1}$H NMR ($CDCl_3$) of compound **L2-b**.

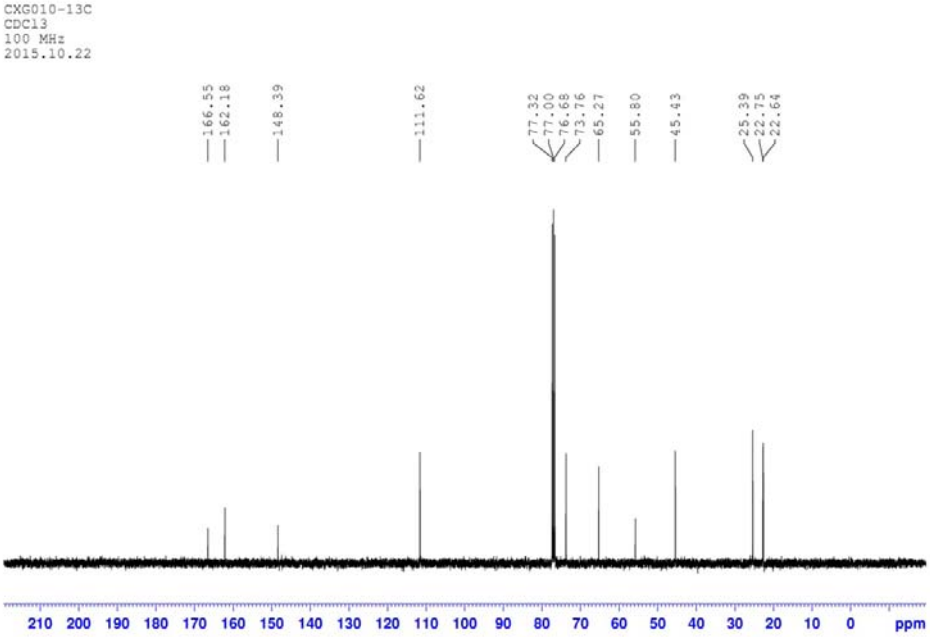


***Supplementary Figure 15.*** $^{13}$C NMR ($CDCl_3$) of compound **L2-b**.

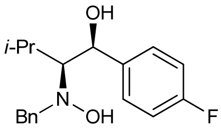


**2a-1**

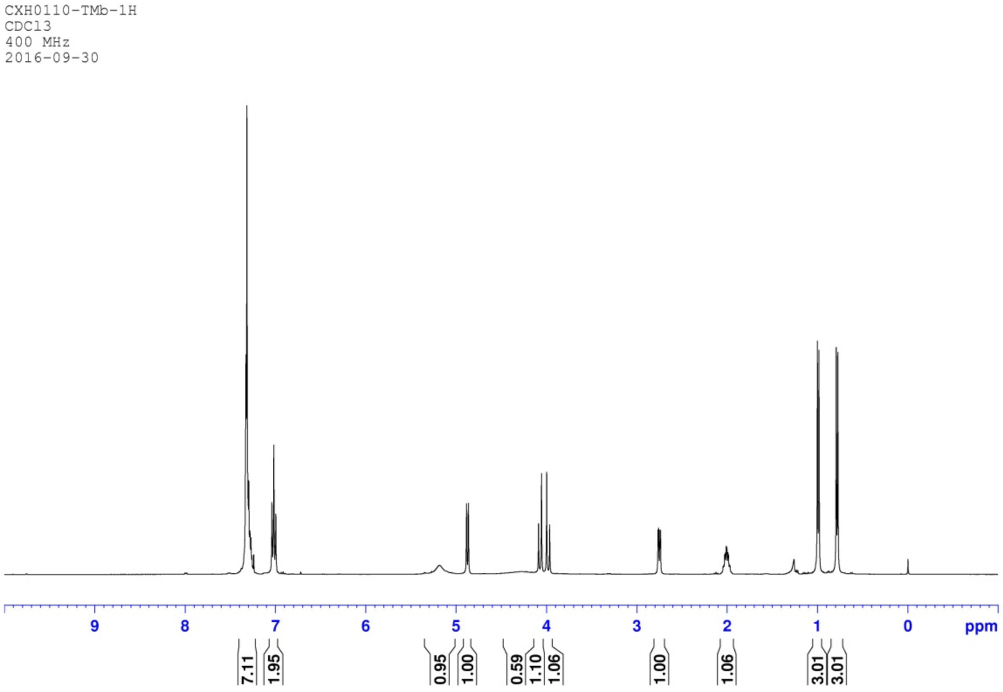


***Supplementary Figure 16.*** $^{1}$H NMR ($CDCl_3$) of compound **2a-1**.

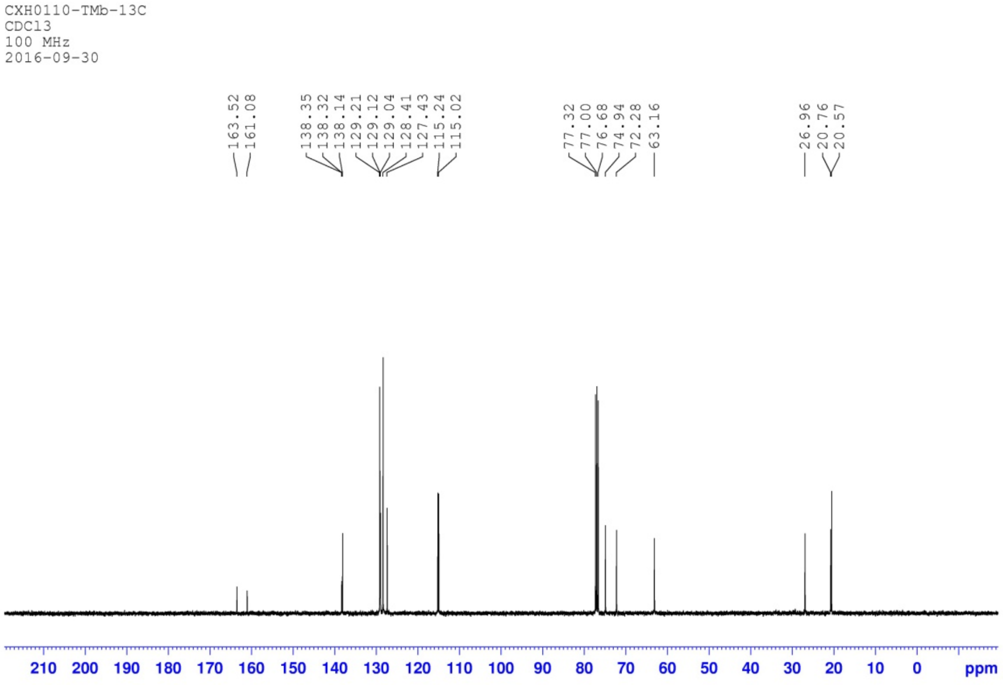


***Supplementary Figure 17.*** $^{13}$C NMR ($CDCl_3$) of compound **2a-1**.

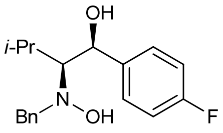


**2a-1**

[Lux® Amylose-1 column, 30 ºC, hexane/EtOH = 85/15 (v/v), 1.0 mL/min, 220 nm]

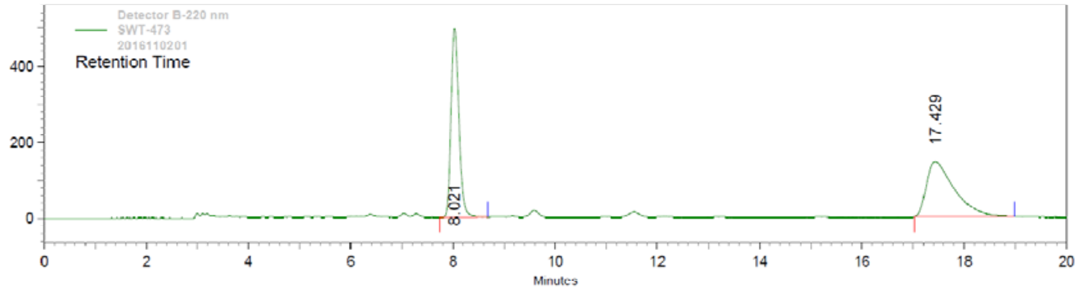


Detector B-220 nm

| Pk # | Retention Time | Area | Area Percent | Height |
|---|---|---|---|---|
| 1 | 8.021 | 5453480 | 49.54 | 497022 |
| 2 | 17.429 | 5554977 | 50.46 | 145289 |

***Supplementary Figure 18.*** Racemate of compound **2a-1**.

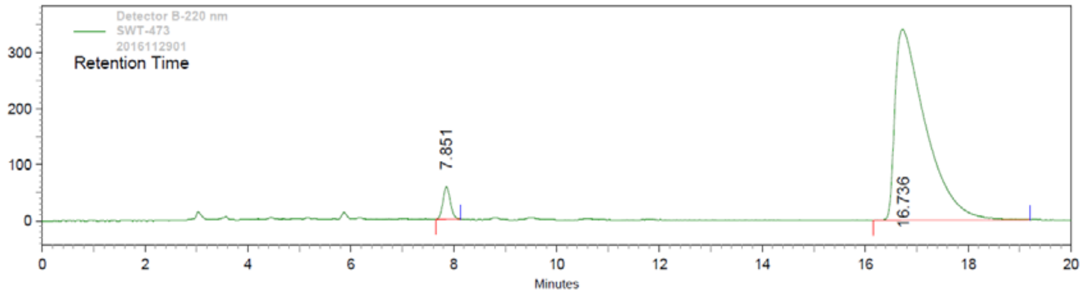


Detector B-220 nm

| Pk # | Retention Time | Area | Area Percent | Height |
|---|---|---|---|---|
| 1 | 7.851 | 586999 | 4.00 | 57895 |
| 2 | 16.736 | 14077559 | 96.00 | 340318 |

***Supplementary Figure 19.*** Enantioenriched mixture of compound **2a-1**.

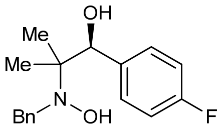

**2b-1**

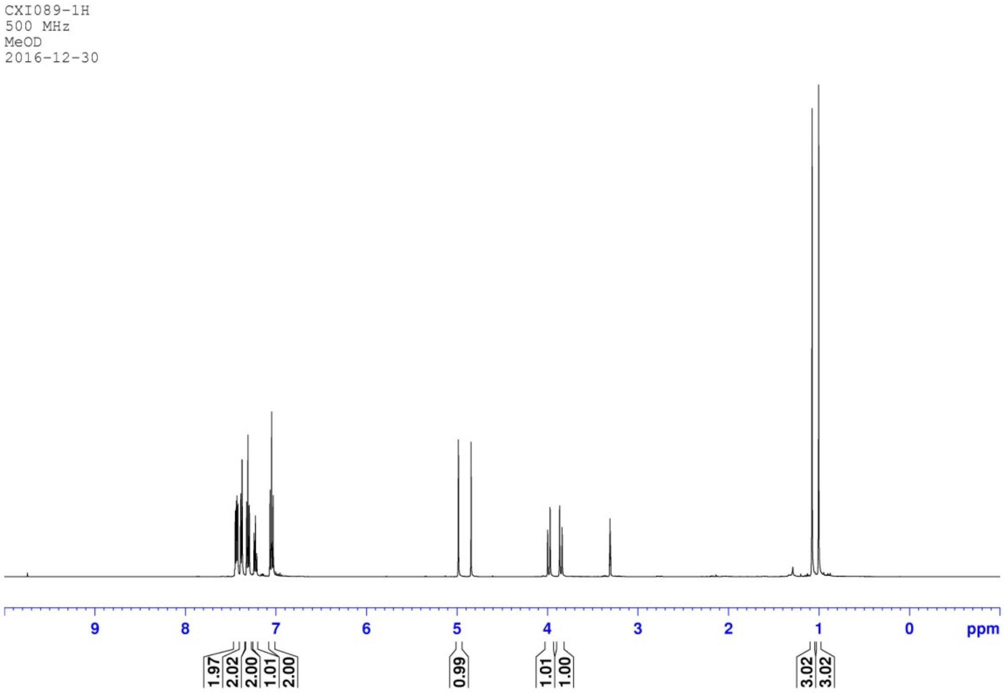


***Supplementary Figure 20.*** $^1$H NMR (MeOD) of compound **2b-1**.

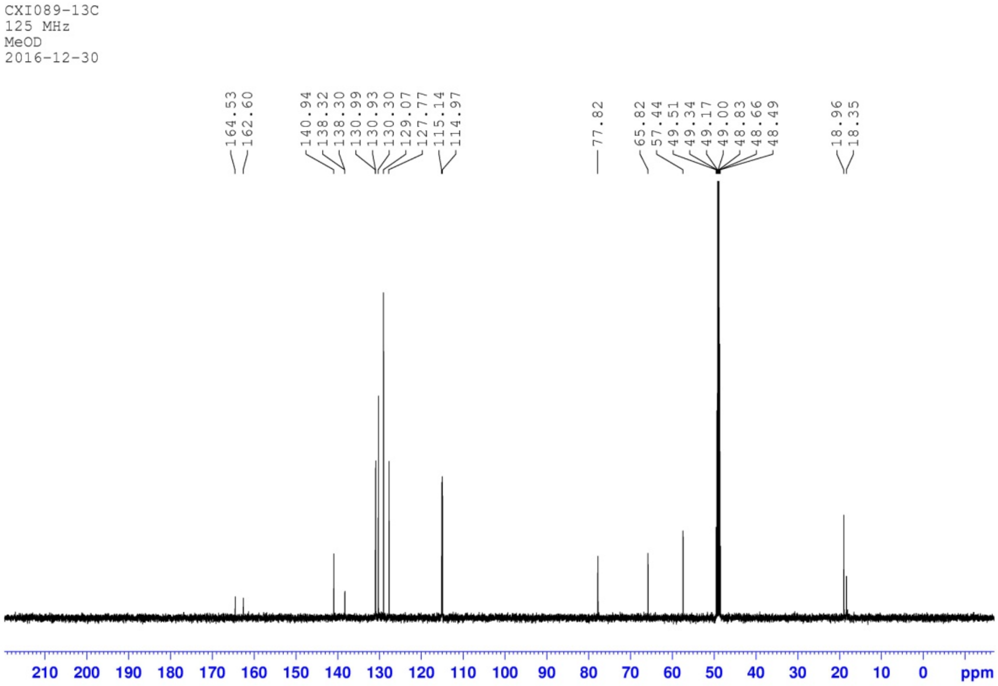


***Supplementary Figure 21.*** $^{13}$C NMR (MeOD) of compound **2b-1**.

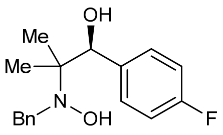

**2b-1**

[Lux® Amylose-1 column, 30 ºC, hexane/EtOH = 60/40 (v/v), 1.0 mL/min, 220 nm]

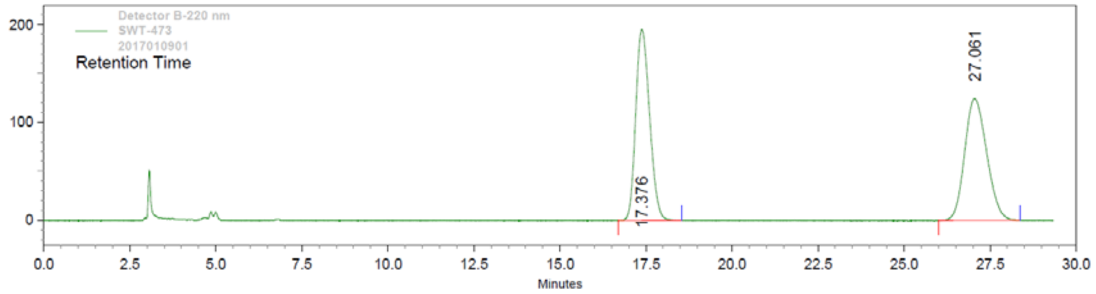


Detector B-220 nm

| Pk # | Retention Time | Area | Area Percent | Height |
|---|---|---|---|---|
| 1 | 17.376 | 5467730 | 49.81 | 195513 |
| 2 | 27.061 | 5509160 | 50.19 | 124332 |

***Supplementary Figure 22.*** Racemate of compound **2b-1**.

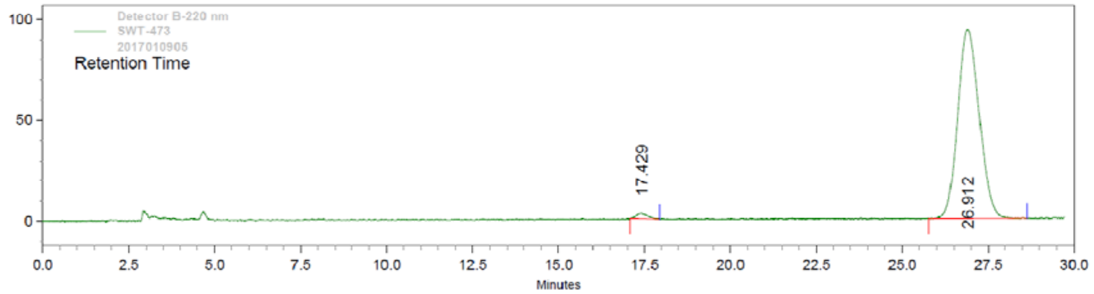


Detector B-220 nm

| Pk # | Retention Time | Area | Area Percent | Height |
|---|---|---|---|---|
| 1 | 17.429 | 61294 | 1.48 | 2833 |
| 2 | 26.912 | 4076642 | 98.52 | 93447 |

***Supplementary Figure 23.*** Enantioenriched mixture of compound **2b-1**.

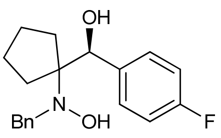


**2c**

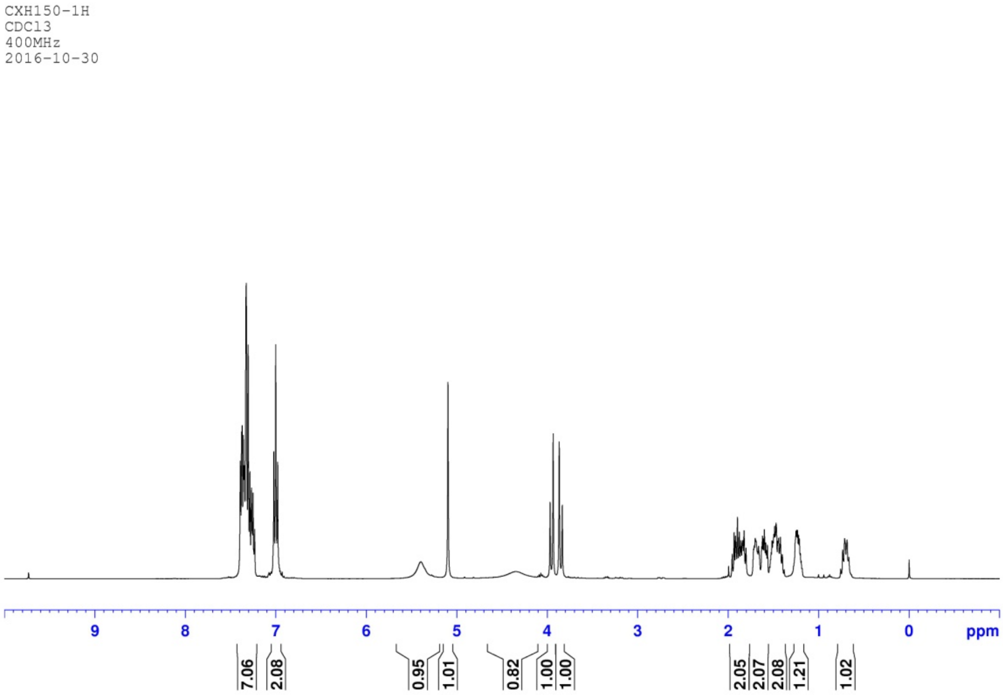


***Supplementary Figure 24.*** $^{1}$H NMR ($CDCl_3$) of compound **2c**.

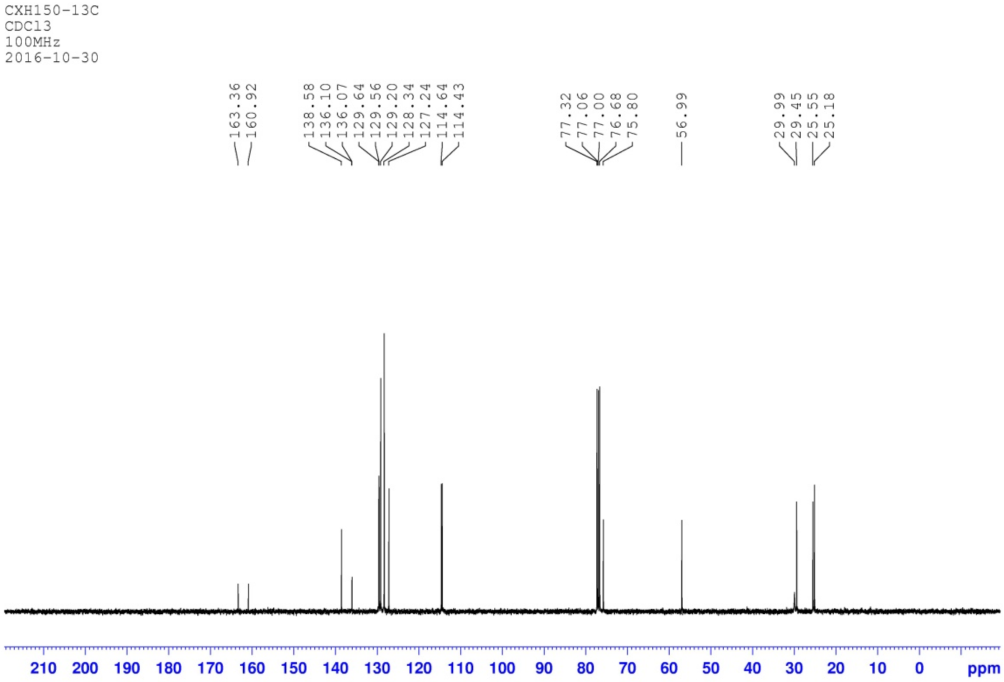


***Supplementary Figure 25.*** $^{13}$C NMR ($CDCl_3$) of compound **2c**.

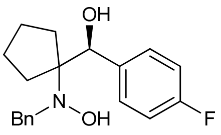


**2c**

[Lux® Amylose-1 column, 30 ºC, hexane/EtOH = 60/40 (v/v), 1.0 mL/min, 220 nm]

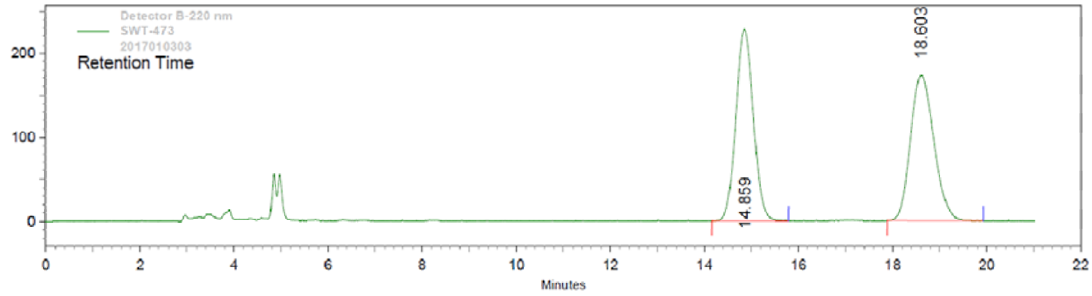


Detector B-220 nm

| Pk # | Retention Time | Area | Area Percent | Height |
|---|---|---|---|---|
| 1 | 14.859 | 5917112 | 49.87 | 227899 |
| 2 | 18.603 | 5947977 | 50.13 | 172520 |

***Supplementary Figure 26.*** Racemate of compound **2c**.

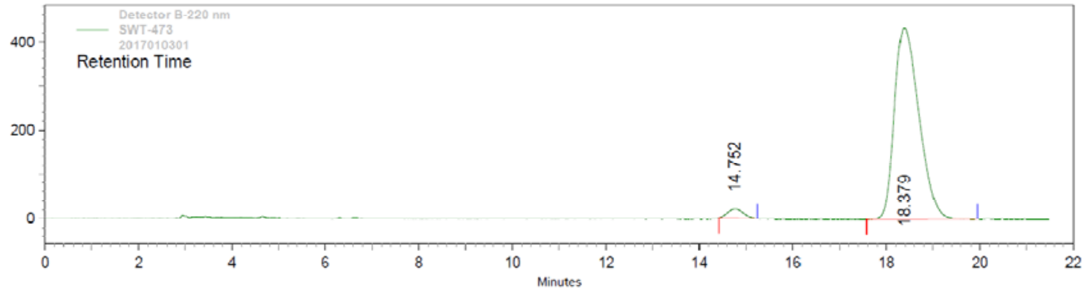


Detector B-220 nm

| Pk # | Retention Time | Area | Area Percent | Height |
|---|---|---|---|---|
| 1 | 14.752 | 495954 | 3.07 | 21489 |
| 2 | 18.379 | 15684191 | 96.93 | 431438 |

***Supplementary Figure 27.*** Enantioenriched mixture of compound **2c**.

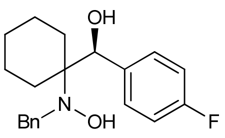


**2d**

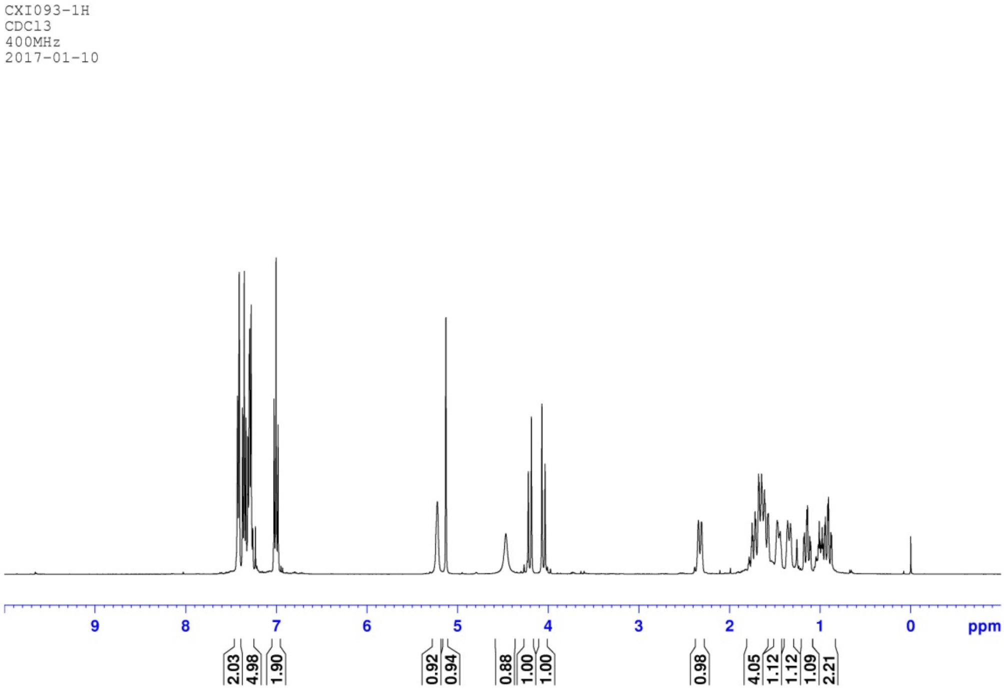


***Supplementary Figure 28.*** $^{1}$H NMR ($CDCl_3$) of compound **2d**.

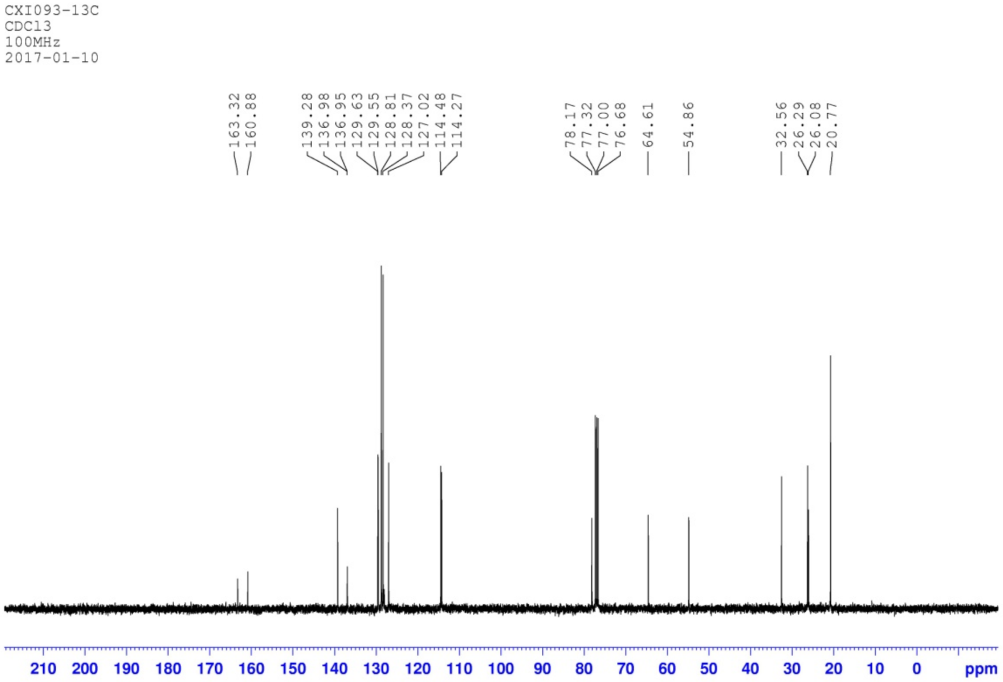


***Supplementary Figure 29.*** $^{13}$C NMR ($CDCl_3$) of compound **2d**.

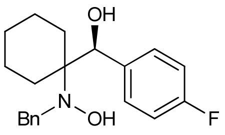


**2d**

[Lux® Amylose-1 column, 30 ºC, hexane/EtOH = 60/40 (v/v), 1.0 mL/min, 225 nm]

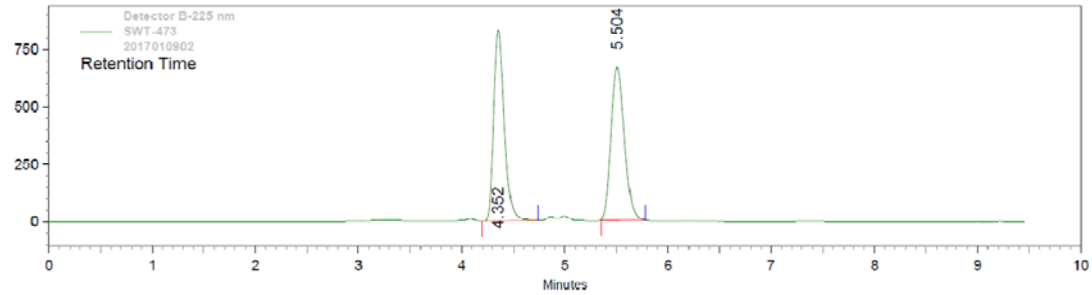


Detector B-225 nm

| Pk # | Retention Time | Area | Area Percent | Height |
|---|---|---|---|---|
| 1 | 4.352 | 6023879 | 49.68 | 833612 |
| 2 | 5.504 | 6101631 | 50.32 | 668896 |

***Supplementary Figure 30.*** Racemate of compound **2d**.

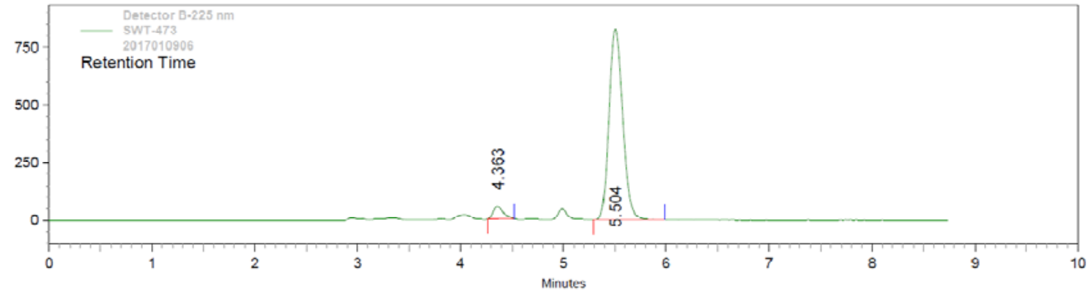


Detector B-225 nm

| Pk # | Retention Time | Area | Area Percent | Height |
|---|---|---|---|---|
| 1 | 4.363 | 342358 | 4.22 | 52792 |
| 2 | 5.504 | 7760813 | 95.78 | 828363 |

***Supplementary Figure 31.*** Enantioenriched mixture of compound **2d**.

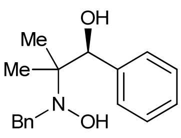


**2b-2**

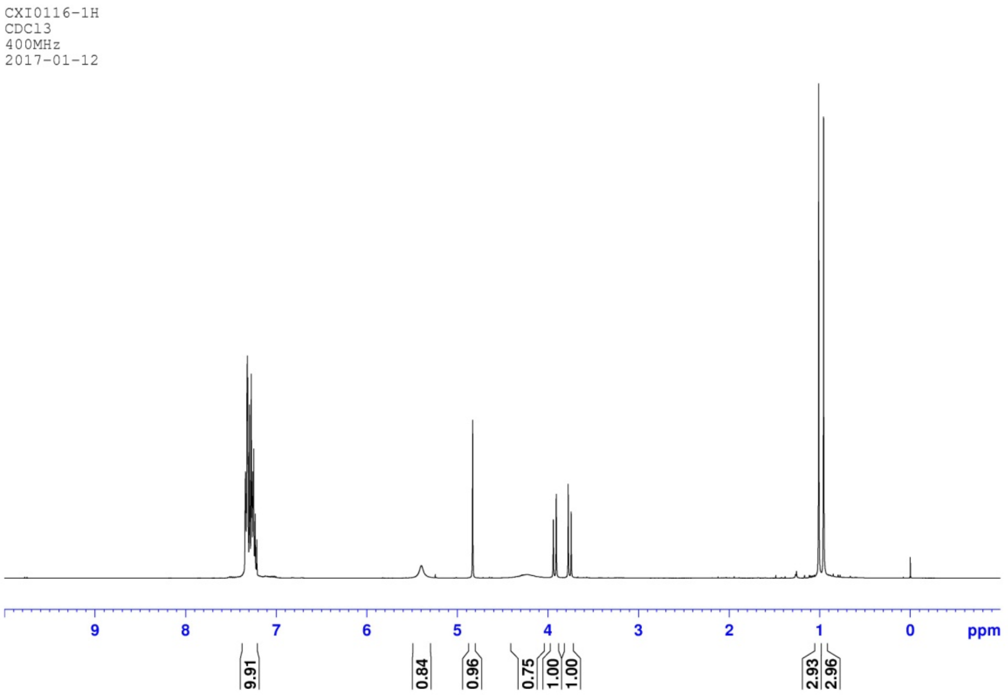


***Supplementary Figure 32.*** $^{1}$H NMR ($CDCl_3$) of compound **2b-2**.

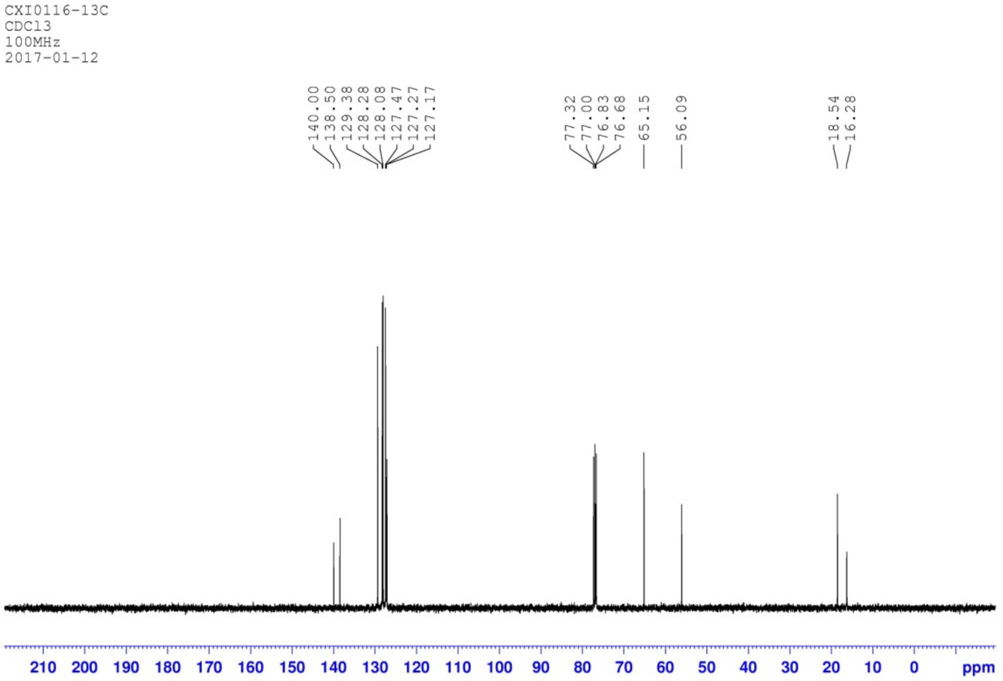


***Supplementary Figure 33.*** $^{13}$C NMR ($CDCl_3$) of compound **2b-2**.

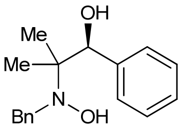


**2b-2**

[Lux® Amylose-1 column, 30 ºC, hexane/EtOH = 60/40 (v/v), 1.0 mL/min, 220 nm]

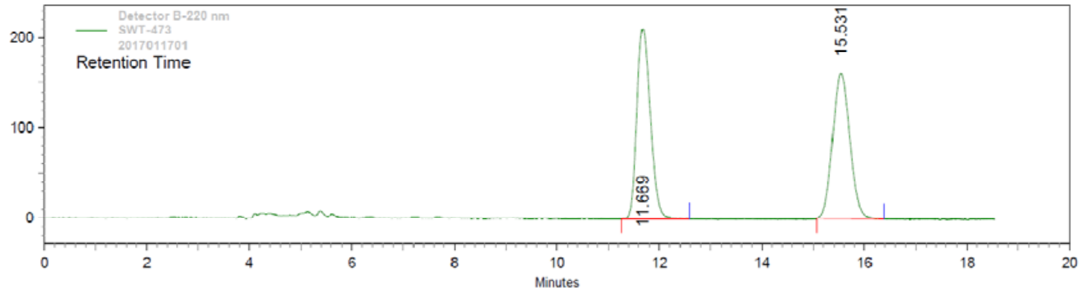


Detector B-220 nm

| Pk # | Retention Time | Area | Area Percent | Height |
|---|---|---|---|---|
| 1 | 11.669 | 3768416 | 49.99 | 210600 |
| 2 | 15.531 | 3770031 | 50.01 | 161108 |

***Supplementary Figure 34.*** Racemate of compound **2b-2**.

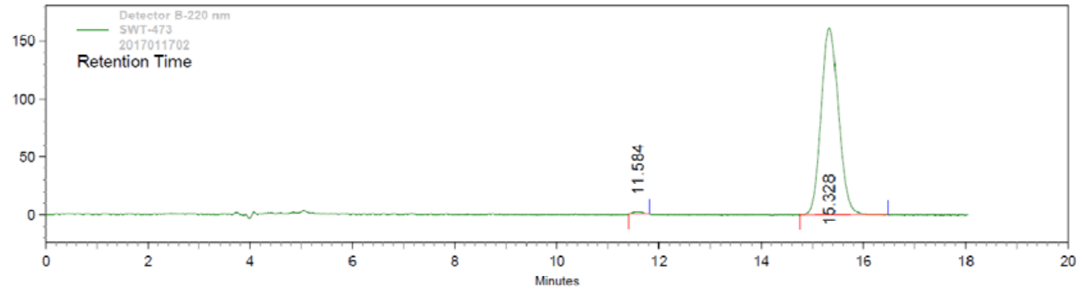


Detector B-220 nm

| Pk # | Retention Time | Area | Area Percent | Height |
|---|---|---|---|---|
| 1 | 11.584 | 22725 | 0.60 | 1961 |
| 2 | 15.328 | 3766262 | 99.40 | 161278 |

***Supplementary Figure 35.*** Enantioenriched mixture of compound **2b-2**.

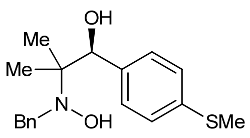


**2b-3**

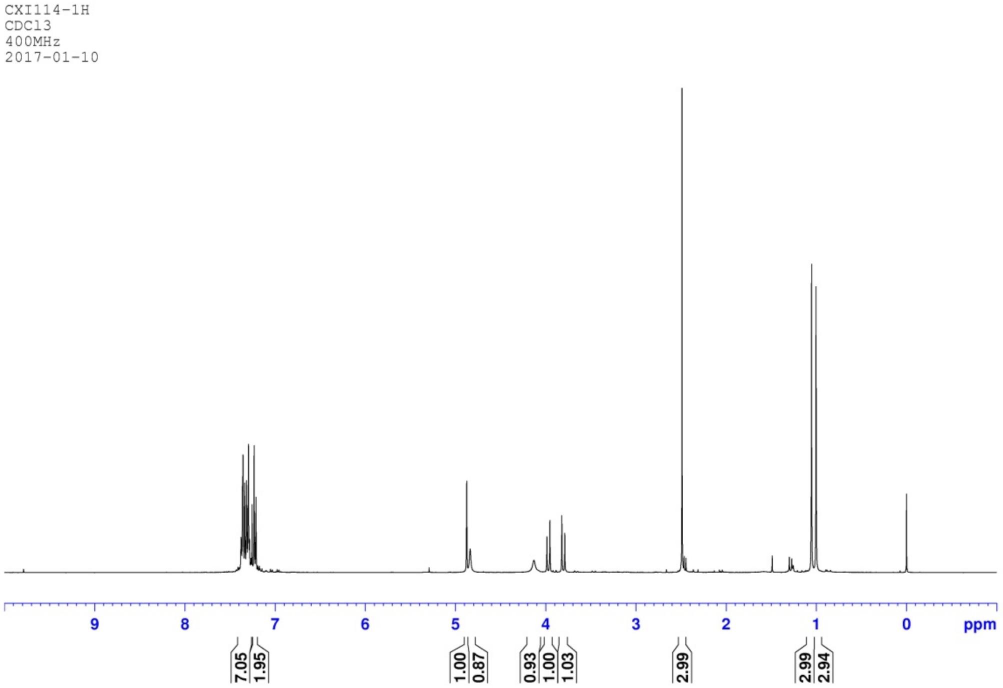


***Supplementary Figure 36.*** $^{1}$H NMR ($CDCl_3$) of compound **2b-3**.

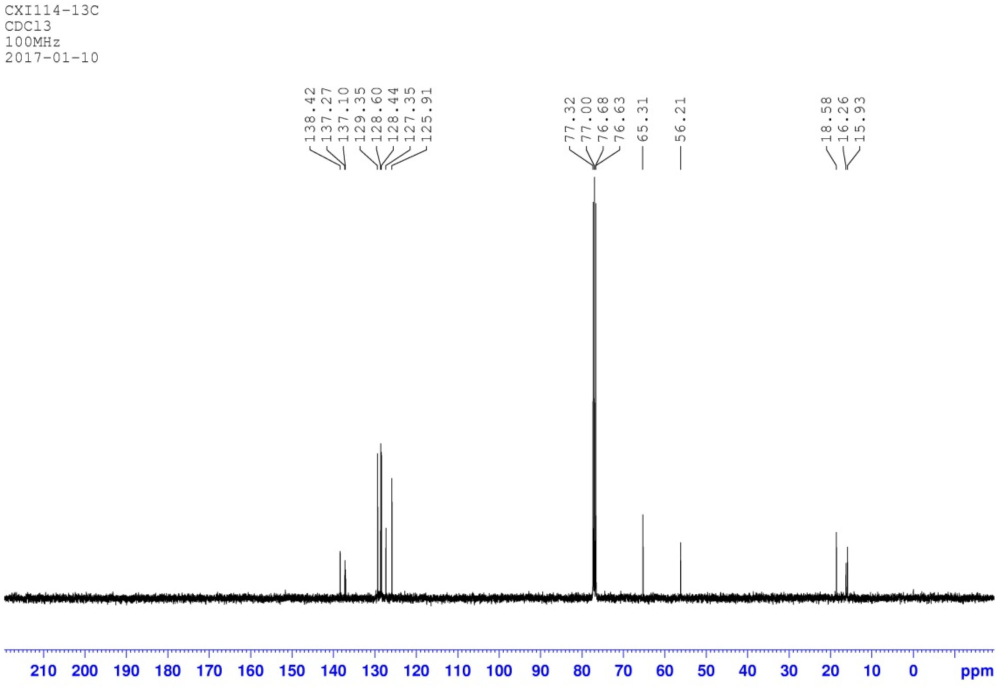


***Supplementary Figure 37.*** $^{13}$C NMR ($CDCl_3$) of compound **2b-3**.

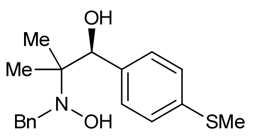

**2b-3**

[Lux® Amylose-1 column, 30 ºC, hexane/*i*-PrOH = 85/15 (v/v), 1.0 mL/min, 259 nm]

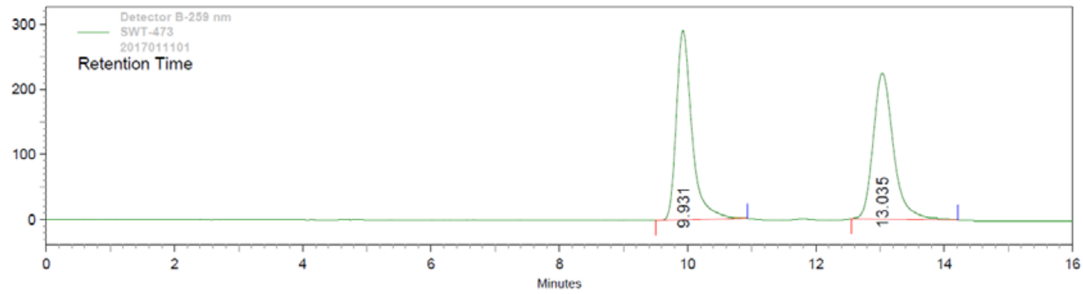


Detector B-259 nm

| Pk # | Retention Time | Area | Area Percent | Height |
|---|---|---|---|---|
| 1 | 9.931 | 4940188 | 50.05 | 291190 |
| 2 | 13.035 | 4930906 | 49.95 | 224641 |

***Supplementary Figure 38.*** Racemate of compound **2b-3**.

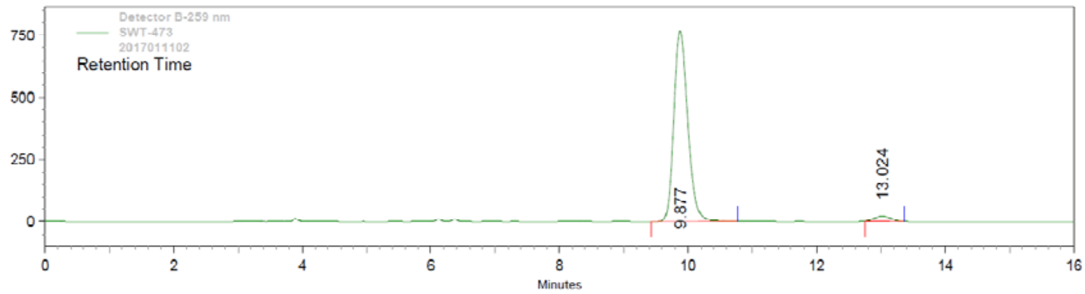


Detector B-259 nm

| Pk # | Retention Time | Area | Area Percent | Height |
|---|---|---|---|---|
| 1 | 9.877 | 11799465 | 97.15 | 768638 |
| 2 | 13.024 | 346714 | 2.85 | 19539 |

***Supplementary Figure 39.*** Enantioenriched mixture of compound **2b-3**.

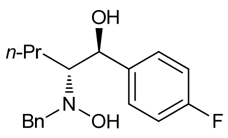


diastereomer of **2e** (major)

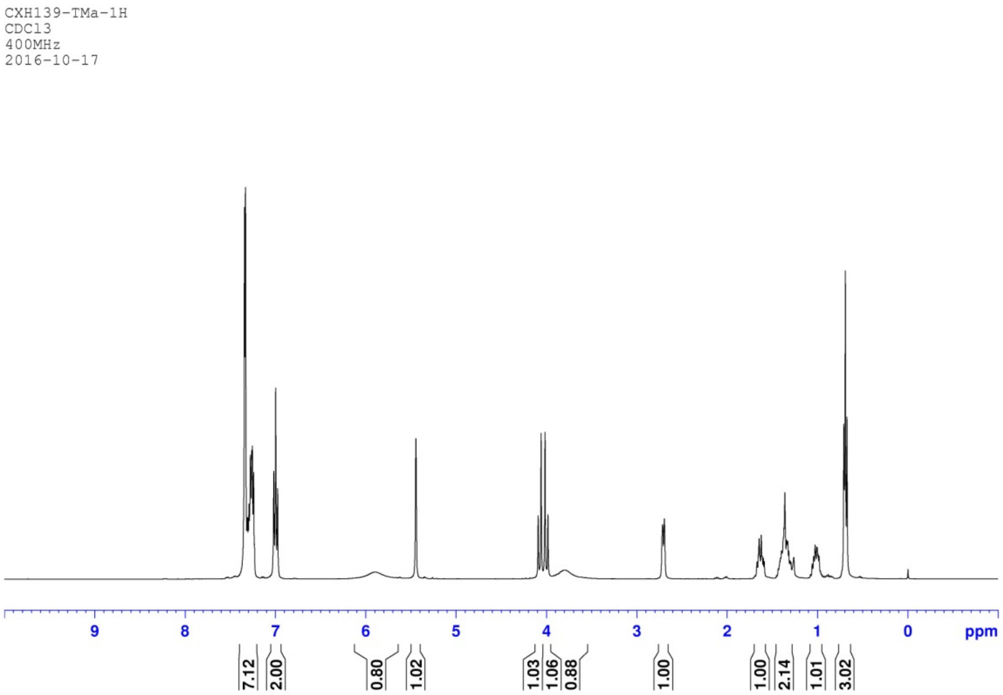


***Supplementary Figure 40.*** $^{1}$H NMR ($CDCl_3$) of the diastereomer of **2e**.

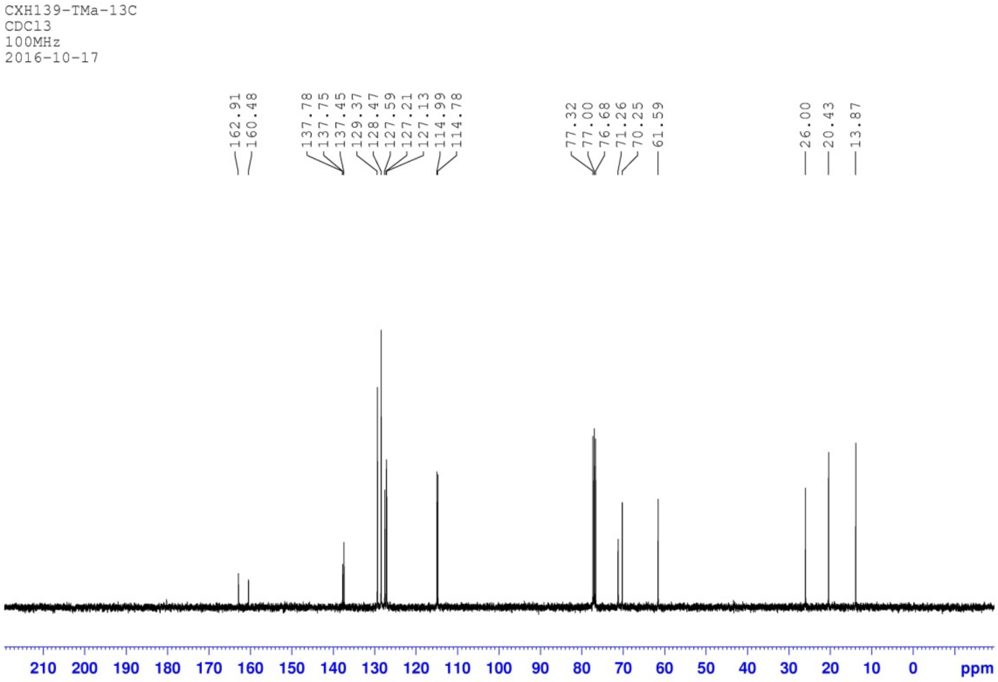


***Supplementary Figure 41.*** $^{13}$C NMR ($CDCl_3$) of the diastereomer of **2e**.

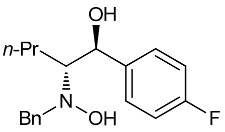 diastereomer of **2e** (major)

[Lux® Amylose-1 column, 30 ºC, hexane/EtOH = 80/20 (v/v), 1.0 mL/min, 265 nm]

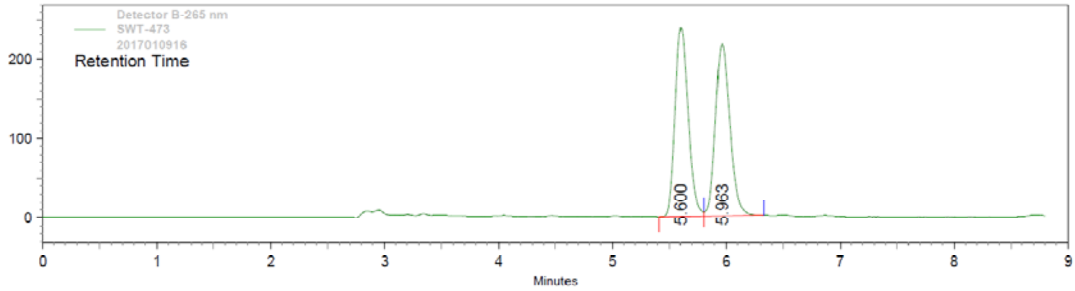


Detector B-265 nm

| Pk # | Retention Time | Area | Area Percent | Height |
|---|---|---|---|---|
| 1 | 5.600 | 1887347 | 49.62 | 238912 |
| 2 | 5.963 | 1916334 | 50.38 | 217813 |

***Supplementary Figure 42.*** Racemate of the diastereomer of **2e**.

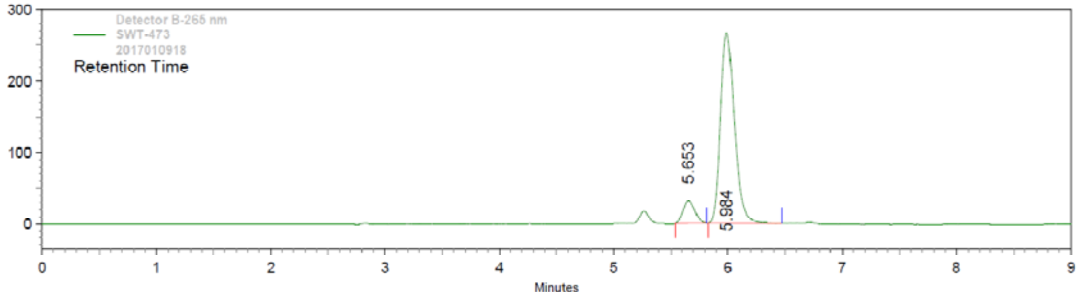


Detector B-265 nm

| Pk # | Retention Time | Area | Area Percent | Height |
|---|---|---|---|---|
| 1 | 5.653 | 218832 | 8.94 | 31100 |
| 2 | 5.984 | 2229724 | 91.06 | 265415 |

***Supplementary Figure 43.*** Enantioenriched mixture of the diastereomer of **2e**.

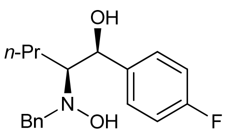

**2e** (minor)

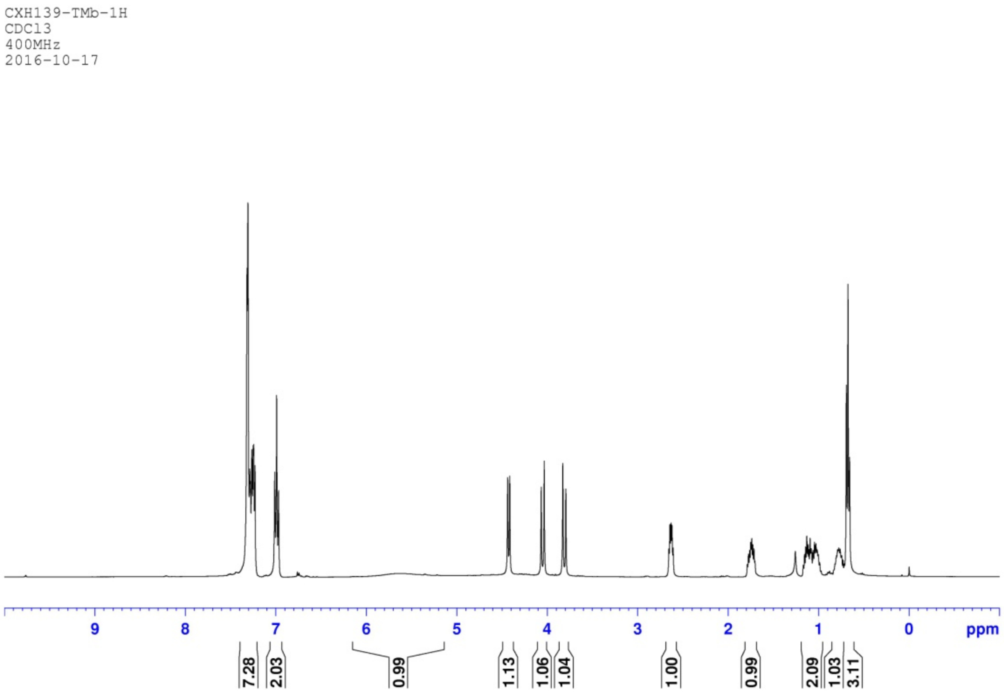


***Supplementary Figure 44.*** $^{1}$H NMR ($CDCl_3$) of compound **2e**.

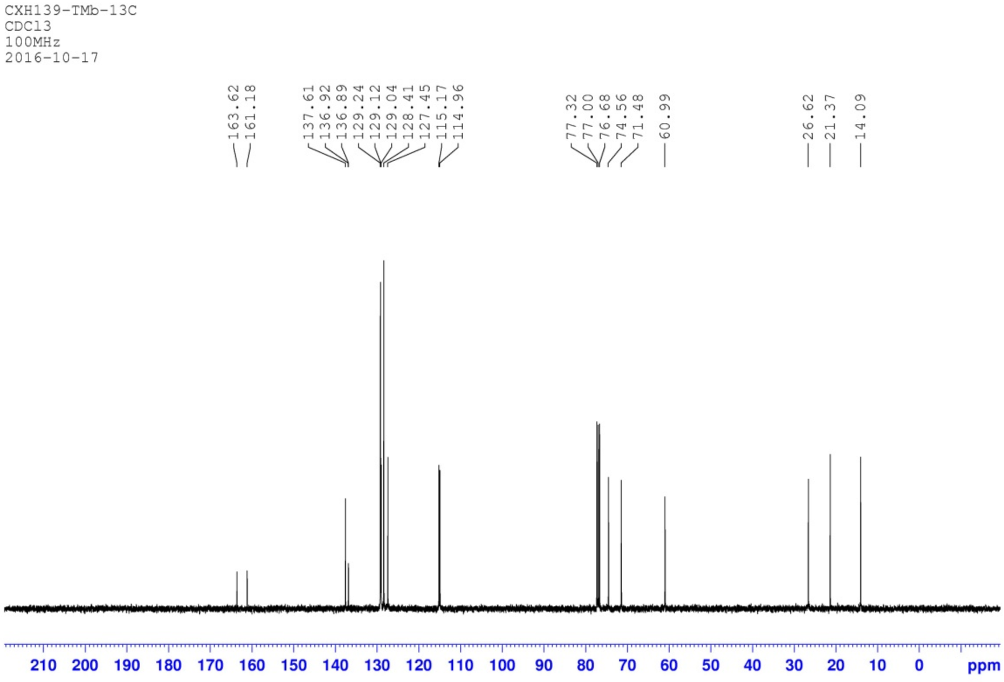


***Supplementary Figure 45.*** $^{13}$C NMR ($CDCl_3$) of compound **2e**.

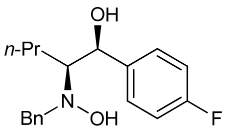

**2e** (minor)

[Lux® Amylose-1 column, 30 ºC, hexane/EtOH = 60/40 (v/v), 1.0 mL/min, 265 nm]

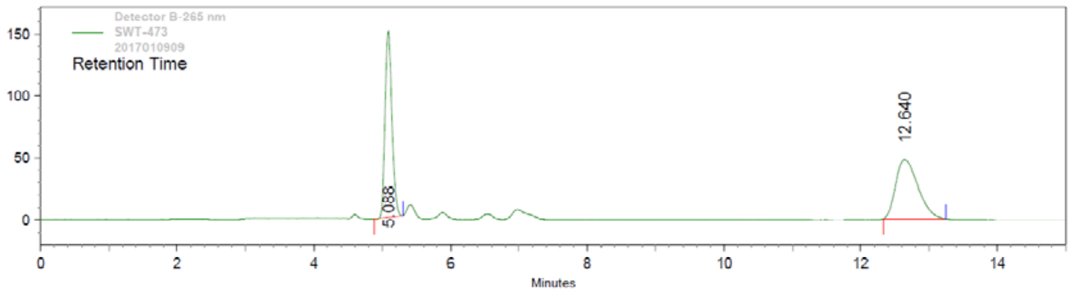


Detector B-265 nm

| | Pk # | Retention Time | Area | Area Percent | Height |
|---|---|---|---|---|---|
| 1 | | 5.088 | 1067356 | 49.90 | 151037 |
| 2 | | 12.640 | 1071474 | 50.10 | 47843 |

***Supplementary Figure 46.*** Racemate of compound **2e**.

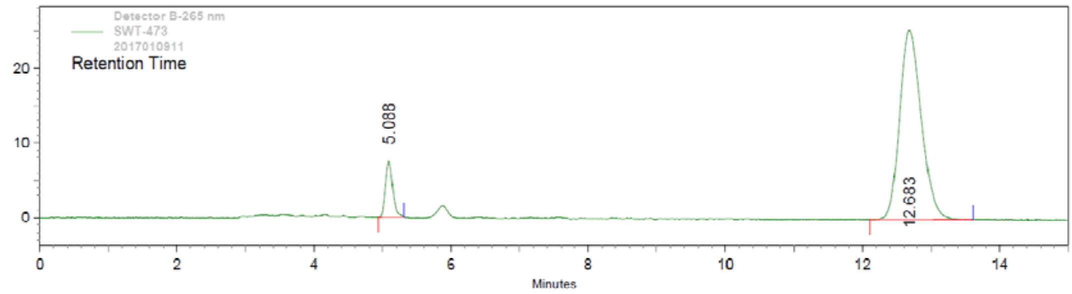


Detector B-265 nm

| | Pk # | Retention Time | Area | Area Percent | Height |
|---|---|---|---|---|---|
| 1 | | 5.088 | 53457 | 8.66 | 7487 |
| 2 | | 12.683 | 563763 | 91.34 | 25425 |

***Supplementary Figure 47.*** Enantioenriched mixture of compound **2e**.

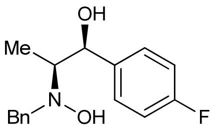


**2f-1** [mixture of diastereomers (*anti*/*syn* = 1.8/1)]

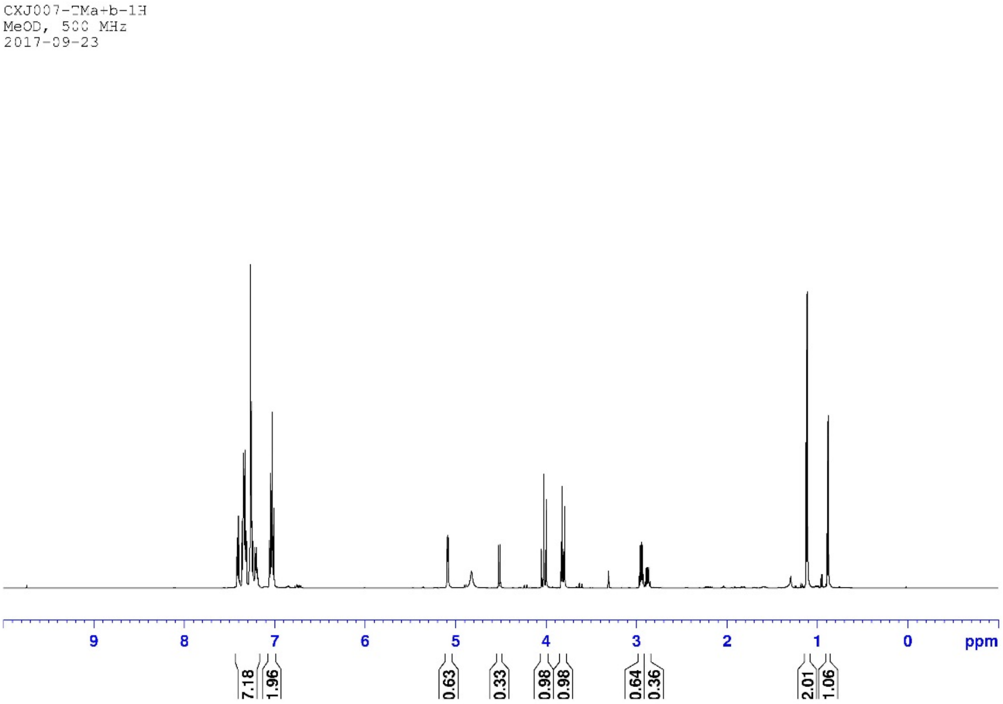


***Supplementary Figure 48.*** $^{1}$H NMR (MeOD) of the diastereomeric mixture of **2f-1** (*anti*/*syn* = 1.8/1).

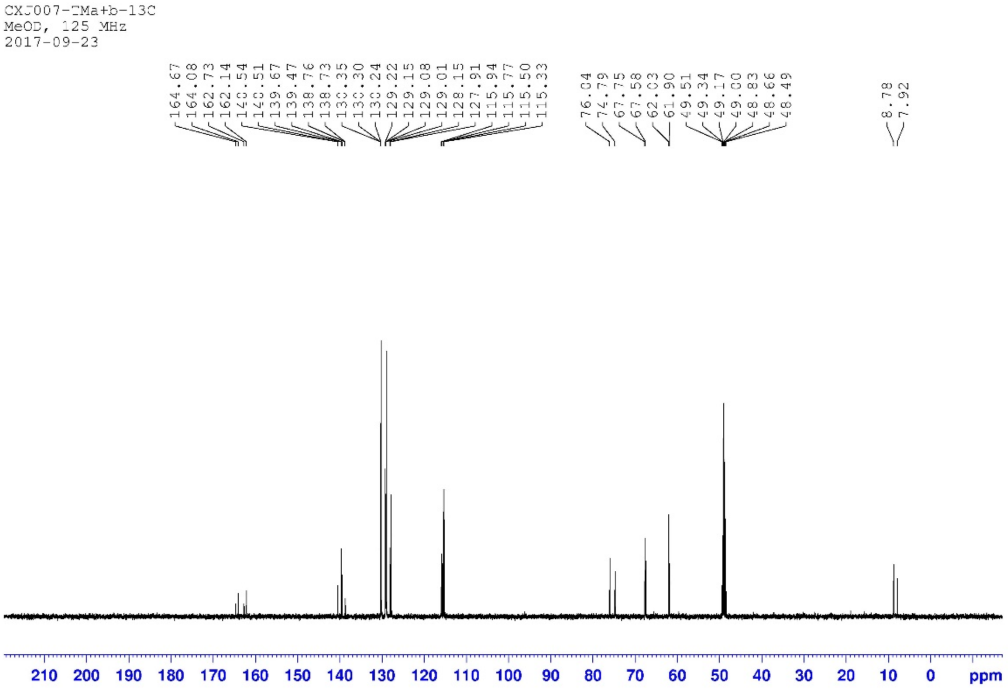


***Supplementary Figure 49.*** $^{13}$C NMR (MeOD) of the diastereomeric mixture of **2f-1** (*anti*/*syn* = 1.8/1).

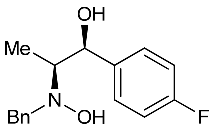

**2f-1**

[Lux® Amylose-1 column, 30 ºC, hexane/EtOH = 90/10 (v/v), 1.0 mL/min, 220 nm]

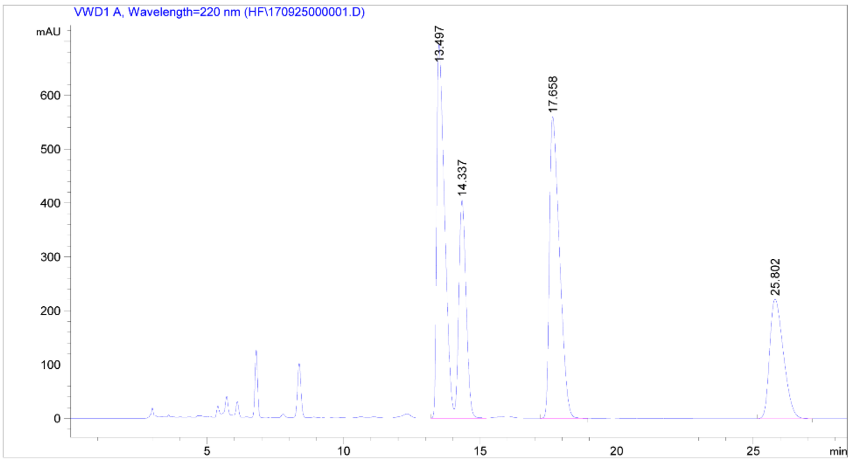


| Pk # | Retention Time [min] | Area [mAU*s] | Height [mAU] | Area Percent % |
|---|---|---|---|---|
| 1 | 13.497 | 1.39515e4 | 695.31830 | 31.9766 |
| 2 | 14.337 | 7537.83789 | 404.98318 | 17.2766 |
| 3 | 17.658 | 1.43654e4 | 559.94757 | 32.9252 |
| 4 | 25.802 | 7775.60791 | 221.91034 | 17.8216 |
| Totals: | | 4.36304e4 | 1882.15939 | 100.0000 |

***Supplementary Figure 50.*** Racemic diastereomeric mixture of **2f-1** (*anti*/*syn* = 1.8/1).

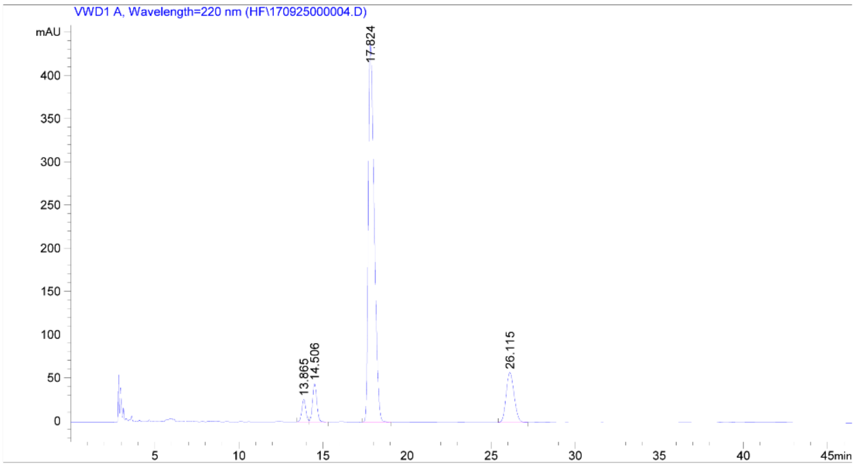


| Pk # | Retention Time [min] | Area [mAU*s] | Height [mAU] | Area Percent % |
|---|---|---|---|---|
| 1 | 13.865 | 436.49664 | 25.91280 | 3.1573 |
| 2 | 14.506 | 786.12903 | 44.09354 | 5.6862 |
| 3 | 17.824 | 1.06956e4 | 437.80728 | 77.3628 |
| 4 | 26.115 | 1907.00378 | 57.84807 | 13.7937 |
| Totals: | | 1.38252e4 | 565.66169 | 100.0000 |

***Supplementary Figure 51.*** Enantioenriched diastereomeric mixture of **2f-1** (*anti*/*syn* = 4.1/1).

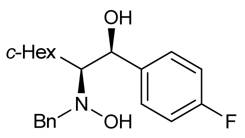


**2g** (major)

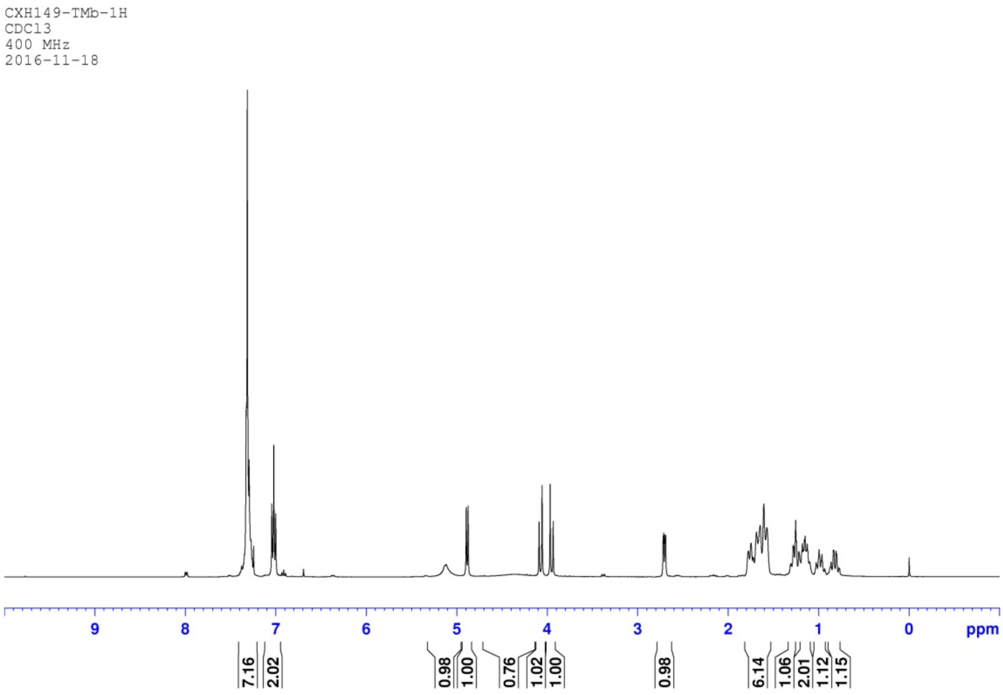


***Supplementary Figure 52.*** $^{1}$H NMR ($CDCl_3$) of compound **2g**.

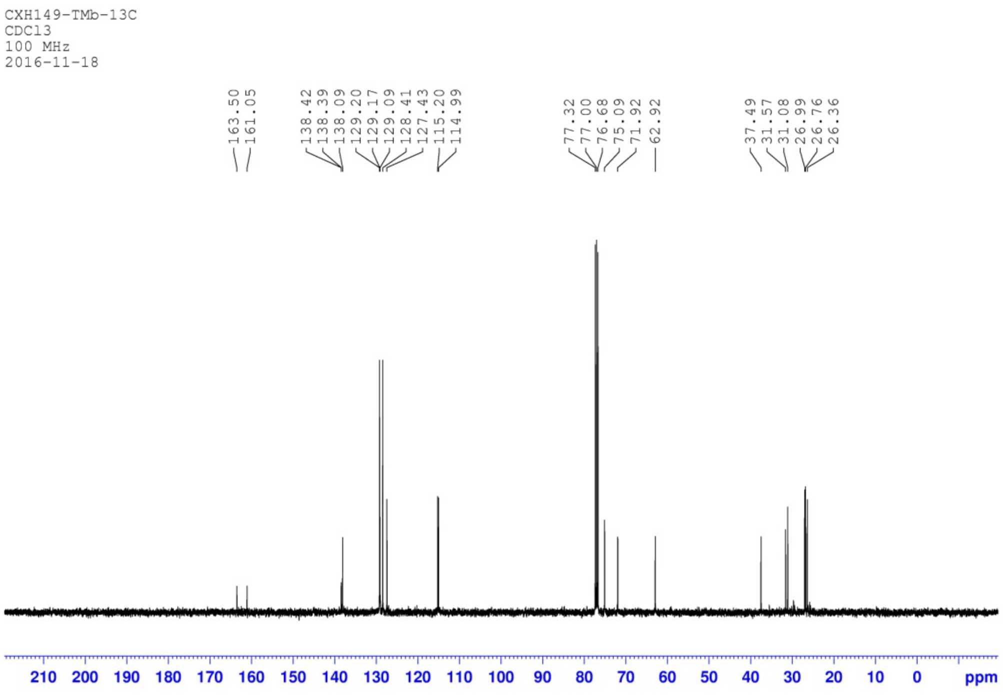


***Supplementary Figure 53.*** $^{13}$C NMR ($CDCl_3$) of compound **2g**.

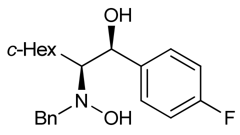

**2g** (major)

[Lux® Amylose-1 column, 30 ºC, hexane/EtOH = 80/20 (v/v), 1.0 mL/min, 225 nm]

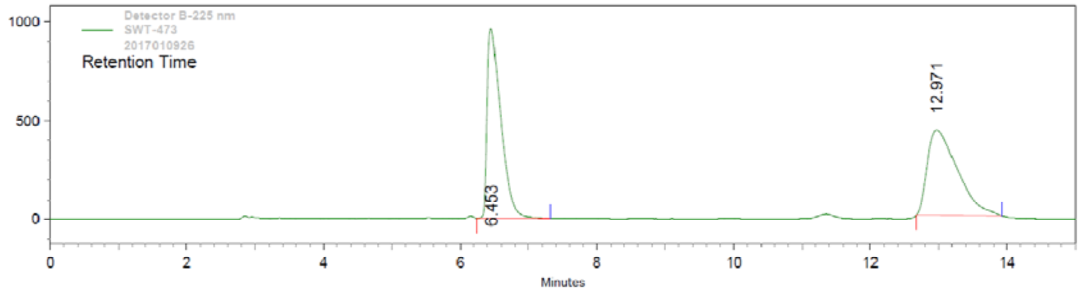


Detector B-225 nm

| | Pk # | Retention Time | Area | Area Percent | Height |
|---|---|---|---|---|---|
| 1 | | 6.453 | 13247334 | 49.79 | 961082 |
| 2 | | 12.971 | 13358417 | 50.21 | 430960 |

***Supplementary Figure 54.*** Racemate of compound **2g**.

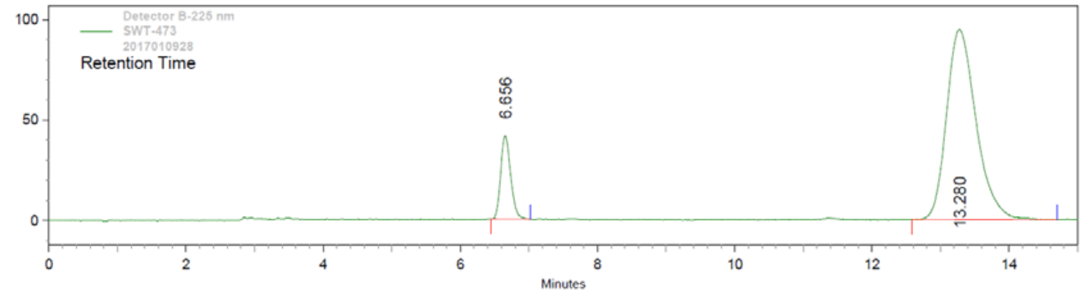


Detector B-225 nm

| | Pk # | Retention Time | Area | Area Percent | Height |
|---|---|---|---|---|---|
| 1 | | 6.656 | 415955 | 12.82 | 41321 |
| 2 | | 13.280 | 2829226 | 87.18 | 94508 |

***Supplementary Figure 55.*** Enantioenriched mixture of compound **2g**.

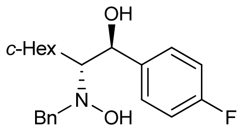


diastereomer of **2g** (minor)

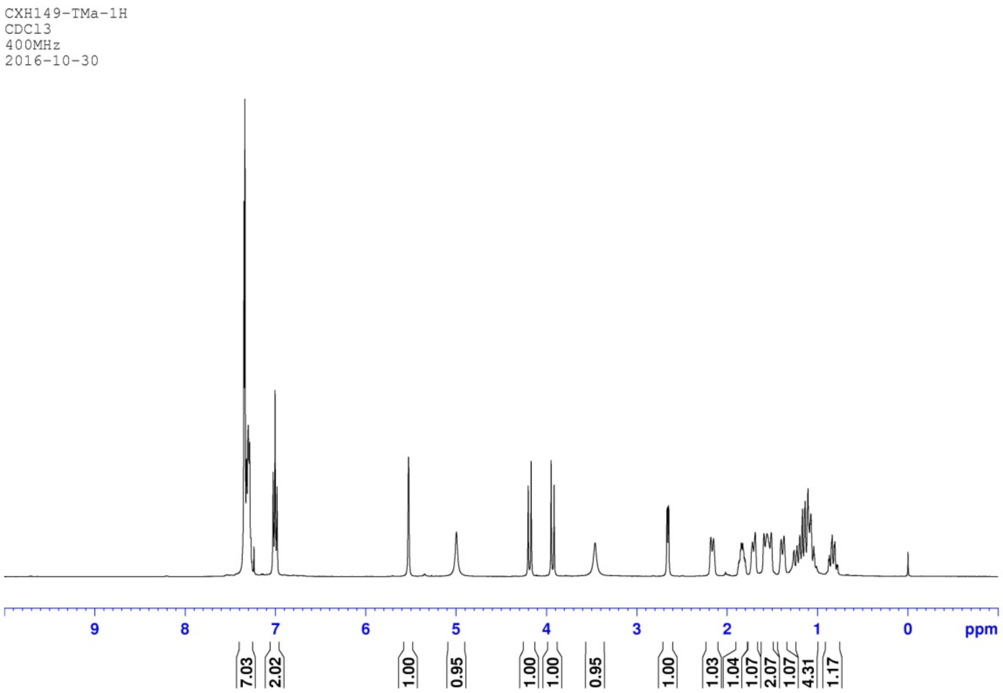


***Supplementary Figure 56.*** $^{1}$H NMR ($CDCl_3$) of the diastereomer of **2g**.

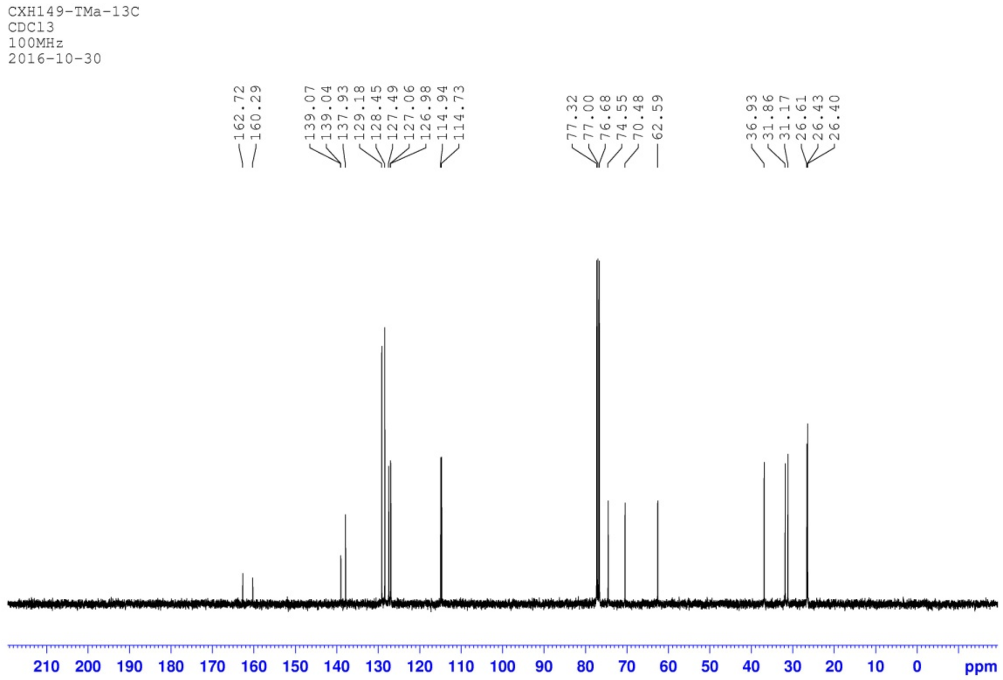


***Supplementary Figure 57.*** $^{13}$C NMR ($CDCl_3$) of the diastereomer of **2g**.

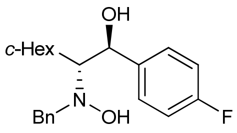

diastereomer of **2g** (minor)

[Lux® Amylose-1 column, 30 ºC, hexane/EtOH = 85/15 (v/v), 1.0 mL/min, 225 nm]

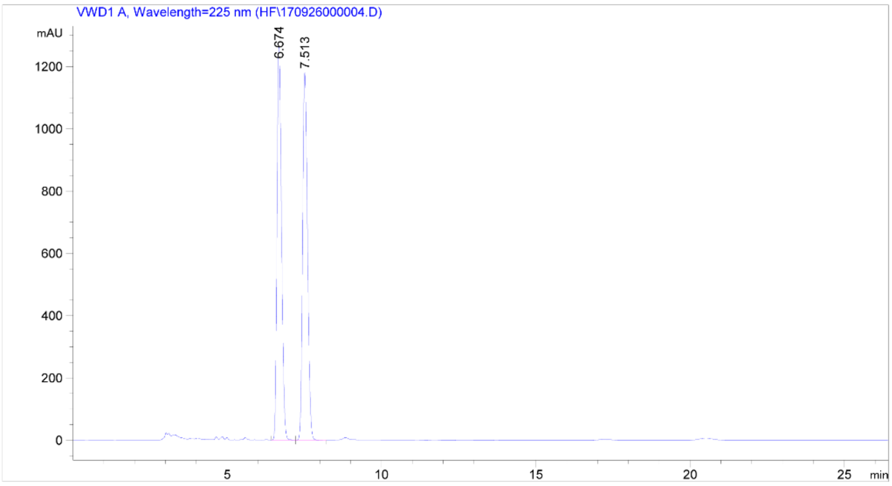


| Pk # | Retention Time [min] | Area [mAU*s] | Height [mAU] | Area Percent % |
|---|---|---|---|---|
| 1 | 6.674 | 1.29336e4 | 1266.73926 | 49.9329 |
| 2 | 7.513 | 1.29684e4 | 1181.47131 | 50.0671 |
| Totals: | | 2.59020e4 | 2448.21057 | 100.0000 |

***Supplementary Figure 58.*** Racemate of the diastereomer of **2g**.

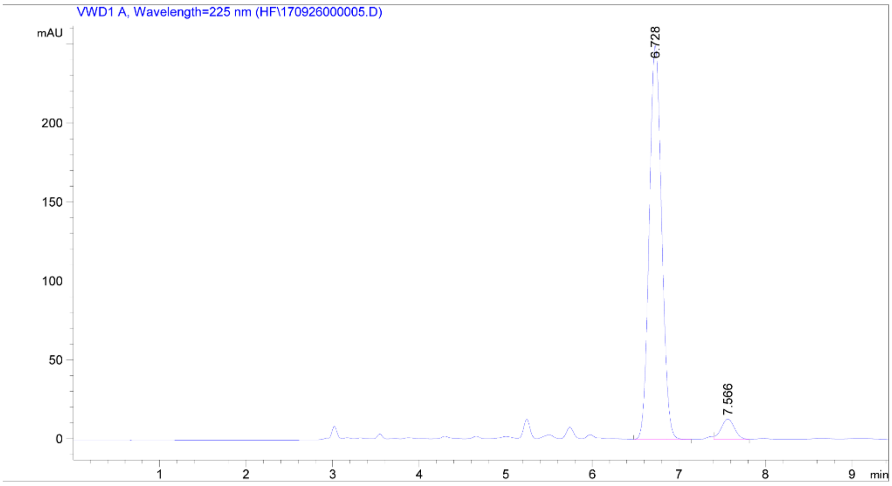


| Pk # | Retention Time [min] | Area [mAU*s] | Height [mAU] | Area Percent % |
|---|---|---|---|---|
| 1 | 6.728 | 2481.00146 | 249.51520 | 94.7077 |
| 2 | 7.566 | 138.64044 | 12.95741 | 5.2923 |
| Totals: | | 2619.64191 | 262.47260 | 100.0000 |

***Supplementary Figure 59.*** Enantioenriched mixture of the diastereomer of **2g**.

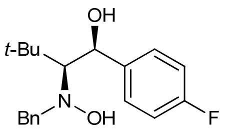


**2h**

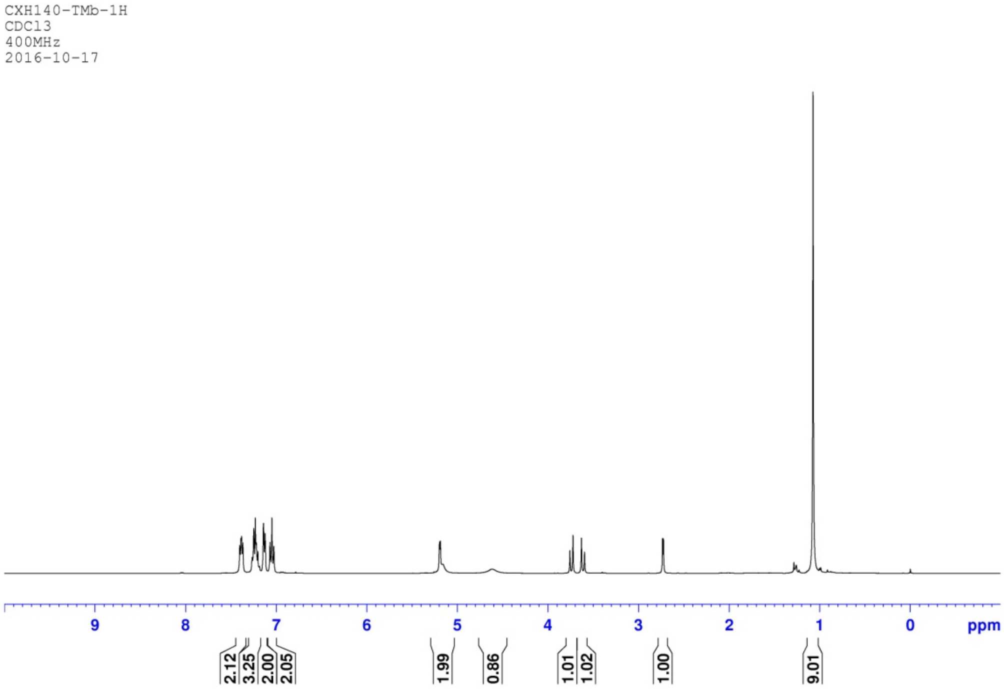


***Supplementary Figure 60.*** $^{1}$H NMR ($CDCl_3$) of compound **2h**.

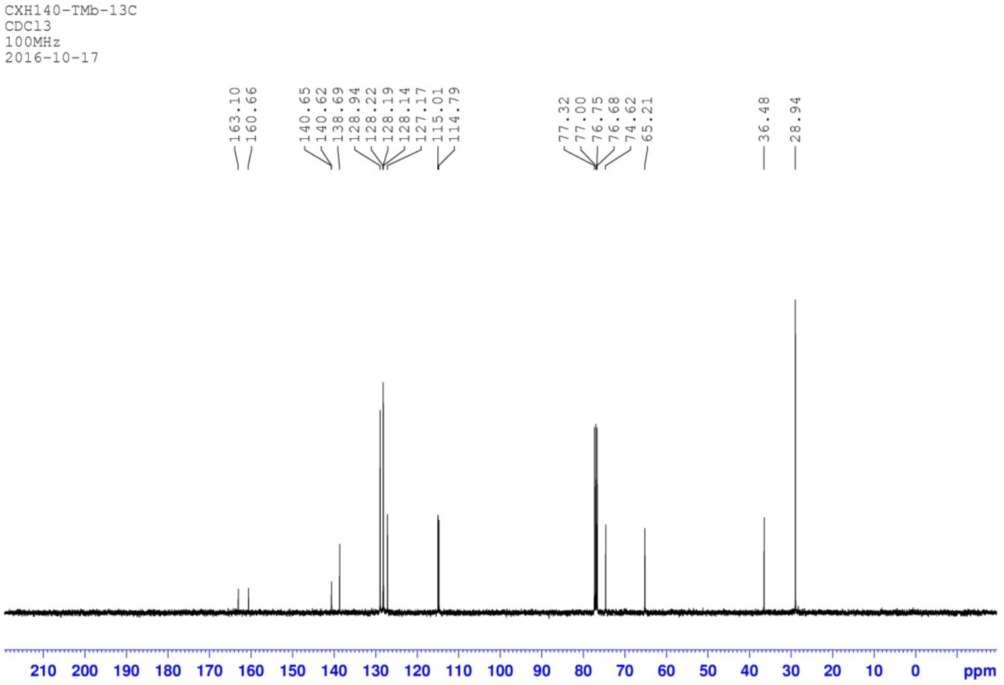


***Supplementary Figure 61.*** $^{13}$C NMR ($CDCl_3$) of compound **2h**.

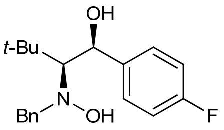

**2h**

[Lux® Amylose-1 column, 30 ºC, hexane/EtOH = 80/20 (v/v), 1.0 mL/min, 230 nm]

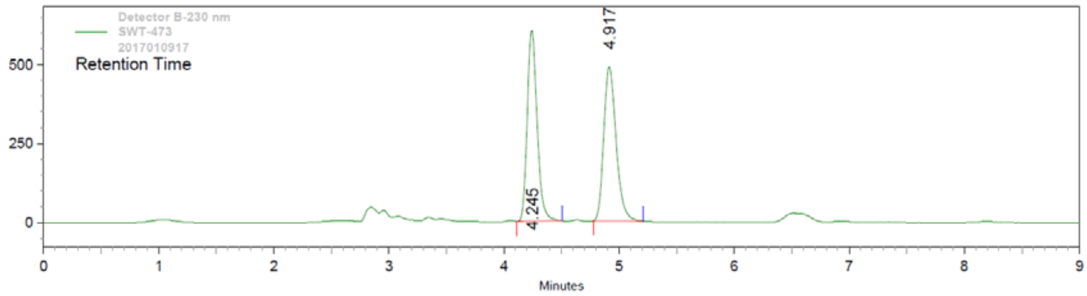


Detector B-230 nm

| Pk # | Retention Time | Area | Area Percent | Height |
|---|---|---|---|---|
| 1 | 4.245 | 3602157 | 49.89 | 603004 |
| 2 | 4.917 | 3617699 | 50.11 | 488489 |

***Supplementary Figure 62.*** Racemate of compound **2h**.

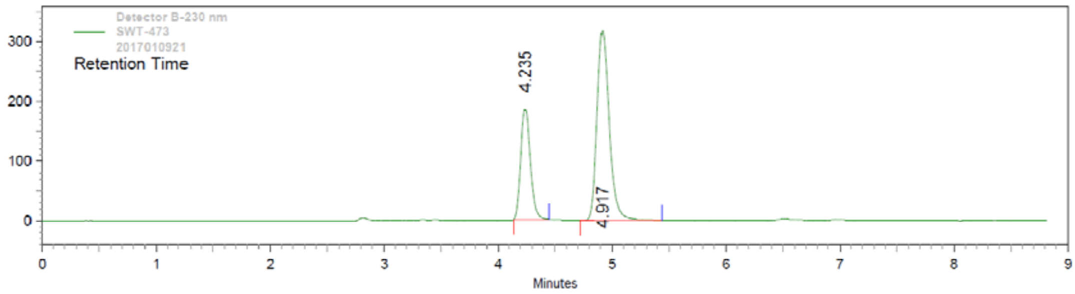


Detector B-230 nm

| Pk # | Retention Time | Area | Area Percent | Height |
|---|---|---|---|---|
| 1 | 4.235 | 1035087 | 30.96 | 185004 |
| 2 | 4.917 | 2308319 | 69.04 | 318433 |

***Supplementary Figure 63.*** Enantioenriched mixture of compound **2h**.

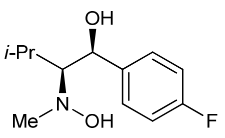


**2i**

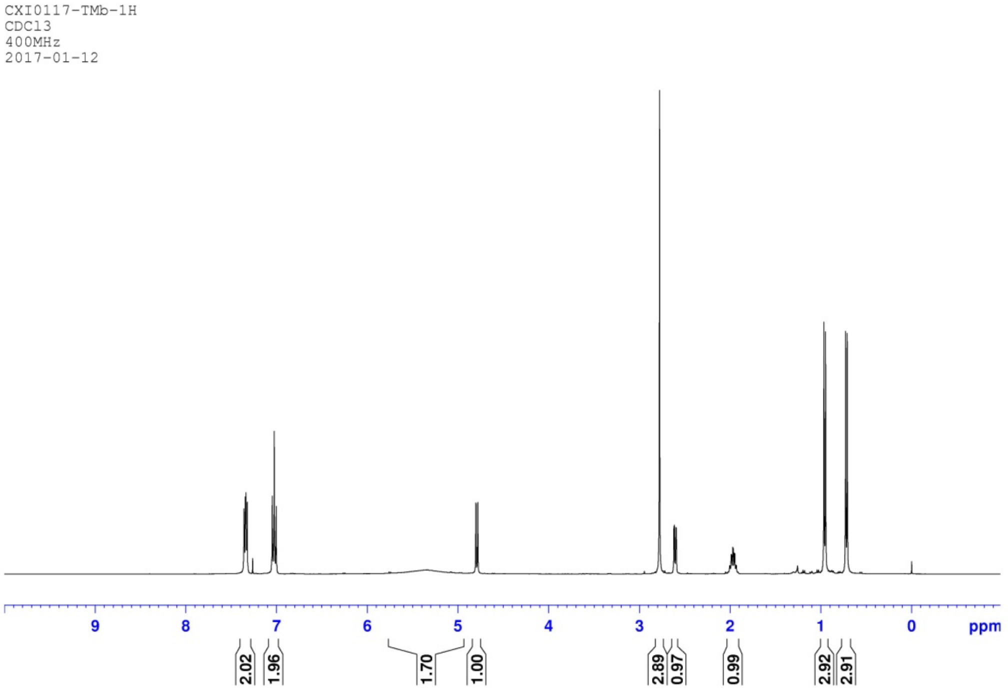


***Supplementary Figure 64.*** $^{1}$H NMR ($CDCl_3$) of compound **2i**.

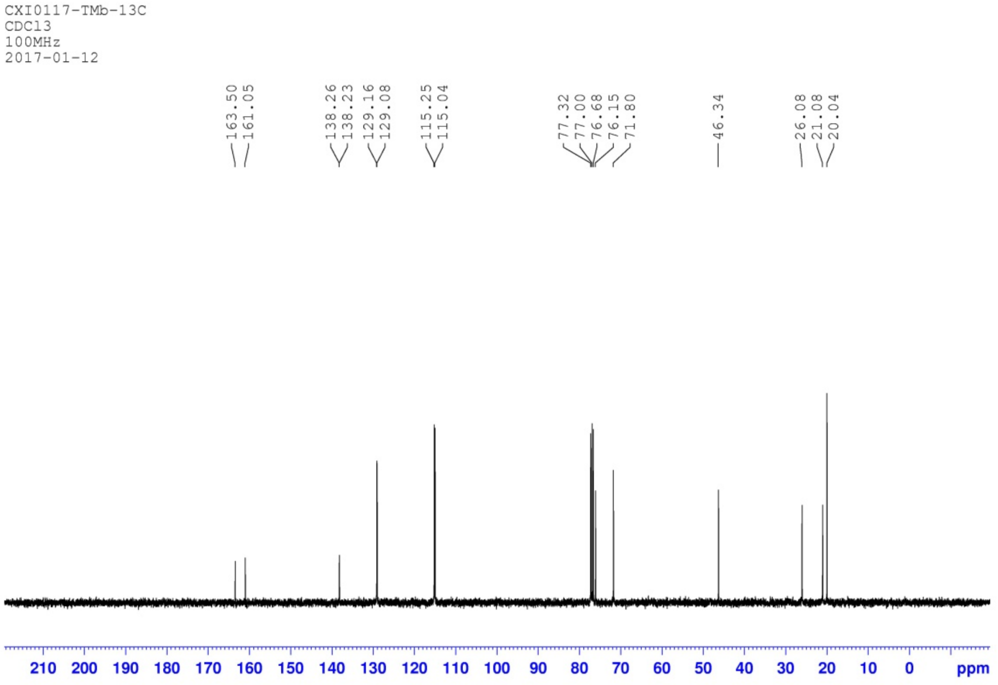


***Supplementary Figure 65.*** $^{13}$C NMR ($CDCl_3$) of compound **2i**.

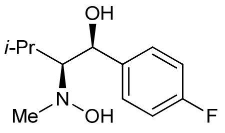

**2i**

[Lux® Amylose-1 column, 30 ºC, hexane/EtOH = 90/10 (v/v), 1.0 mL/min, 220 nm]

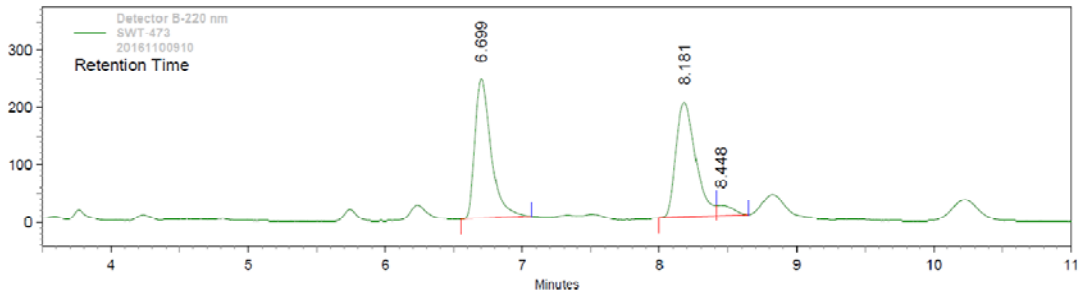


Detector B-220 nm

| | Pk # | Retention Time | Area | Area Percent | Height |
|---|---|---|---|---|---|
| 1 | | 6.699 | 2034313 | 48.28 | 243358 |
| 2 | | 8.181 | 2033228 | 48.25 | 200396 |
| 3 | | 8.448 | 146456 | 3.48 | 18682 |

***Supplementary Figure 66.*** Racemate of compound **2i**.

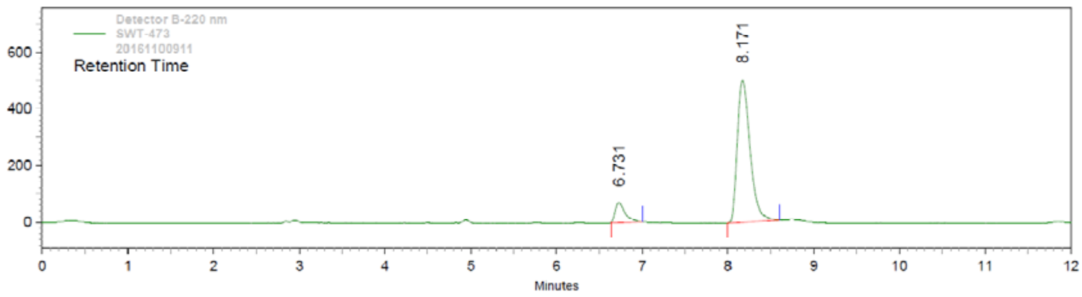


Detector B-220 nm

| | Pk # | Retention Time | Area | Area Percent | Height |
|---|---|---|---|---|---|
| 1 | | 6.731 | 564936 | 9.66 | 68421 |
| 2 | | 8.171 | 5284531 | 90.34 | 501303 |

***Supplementary Figure 67.*** Enantioenriched mixture of compound **2i**.

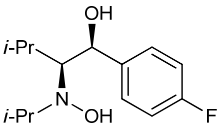


**2j**

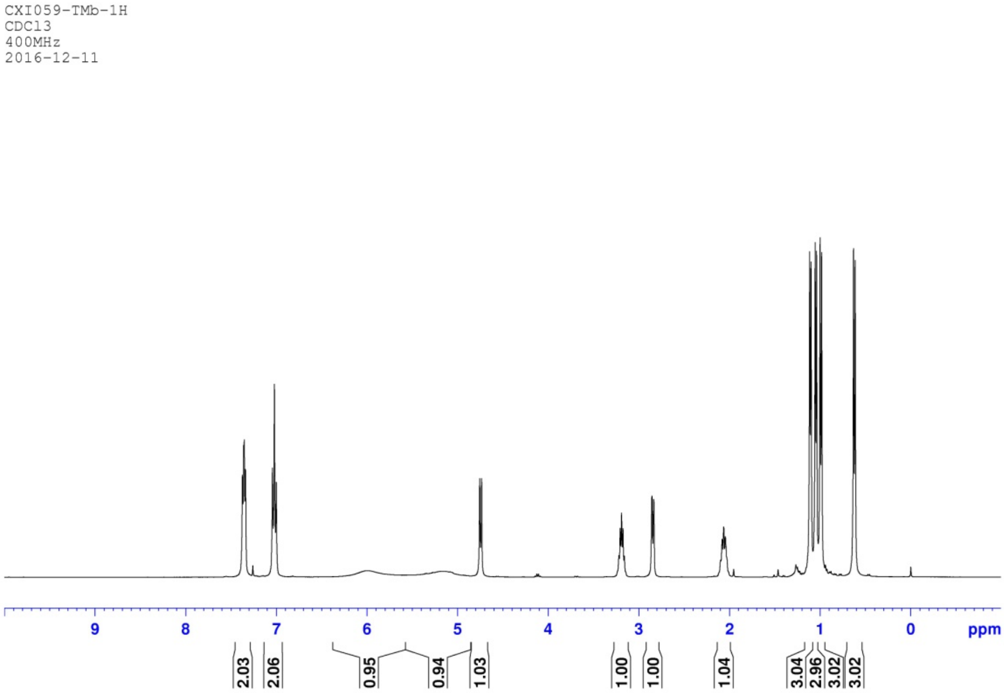


***Supplementary Figure 68.*** $^{1}$H NMR ($CDCl_3$) of compound **2j**.

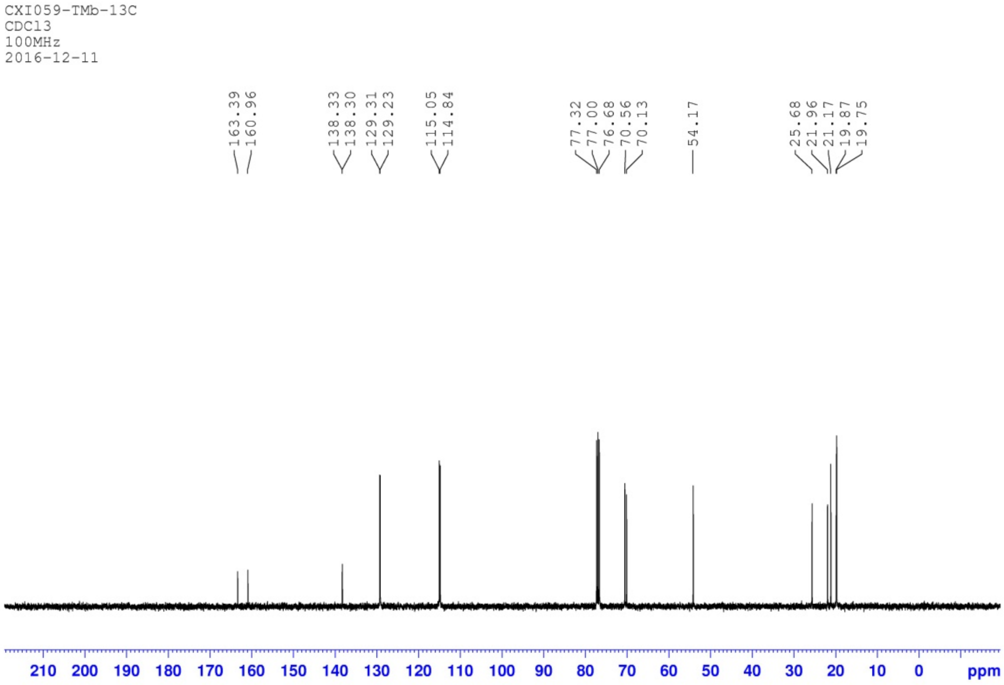


***Supplementary Figure 69.*** $^{13}$C NMR ($CDCl_3$) of compound **2j**.

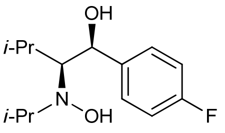


**2j**

[Lux® Amylose-1 column, 30 ºC, hexane/EtOH = 80/20 (v/v), 1.0 mL/min, 265 nm]

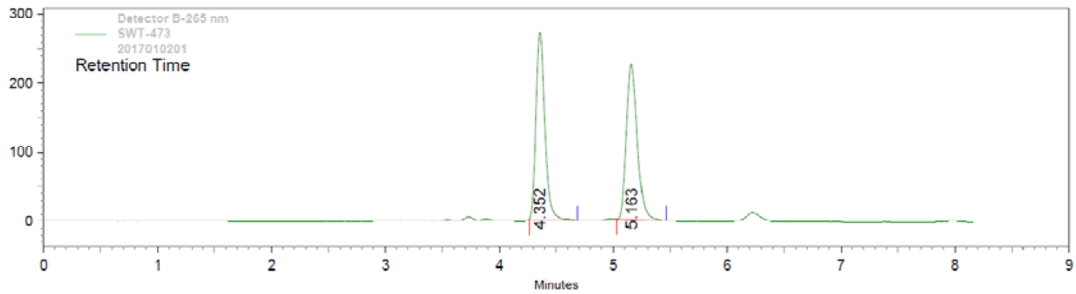


Detector B-265 nm

| Pk # | Retention Time | Area | Area Percent | Height |
|---|---|---|---|---|
| 1 | 4.352 | 1525904 | 50.07 | 273356 |
| 2 | 5.163 | 1521489 | 49.93 | 225573 |

***Supplementary Figure 70.*** Racemate of compound **2j**.

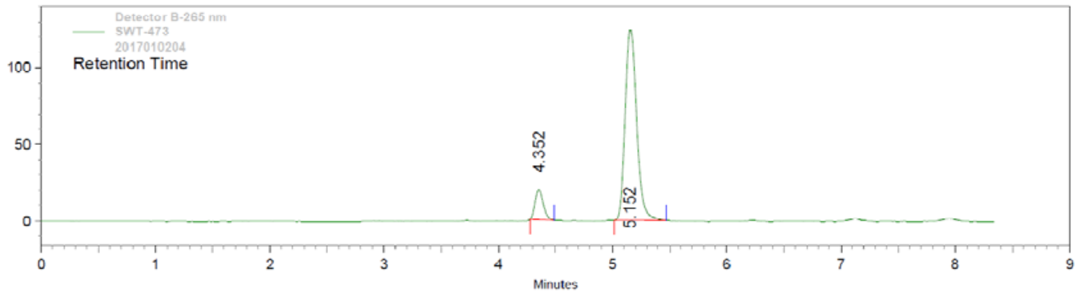


Detector B-265 nm

| Pk # | Retention Time | Area | Area Percent | Height |
|---|---|---|---|---|
| 1 | 4.352 | 97055 | 10.38 | 19320 |
| 2 | 5.152 | 838037 | 89.62 | 123964 |

***Supplementary Figure 71.*** Enantioenriched mixture of compound **2j**.

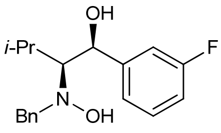


**2a-2**

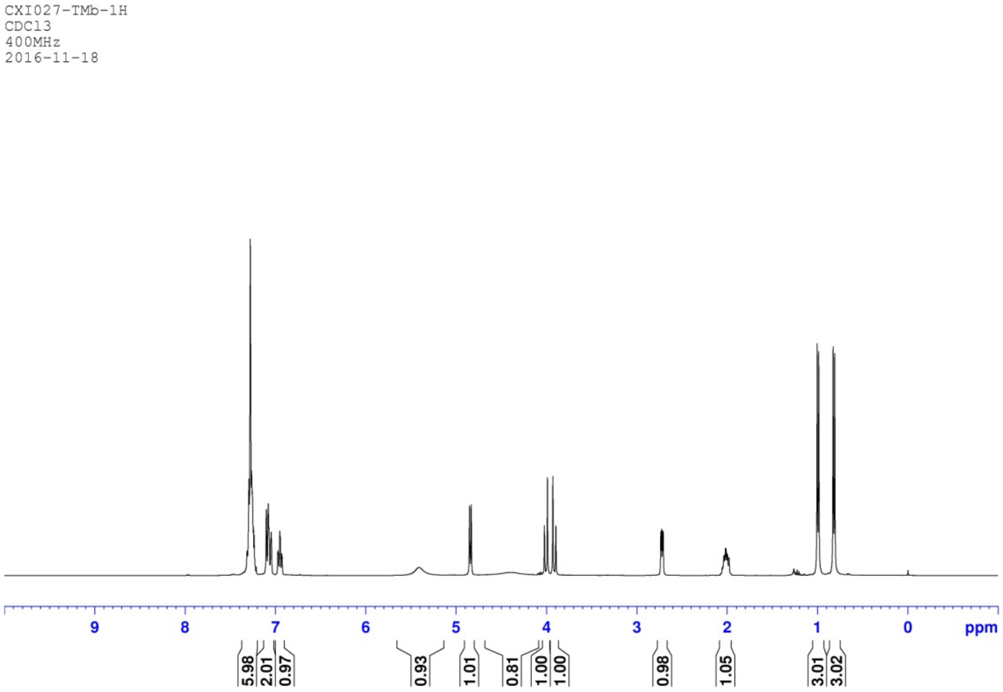


***Supplementary Figure 72.*** $^{1}$H NMR ($CDCl_3$) of compound **2a-2**.

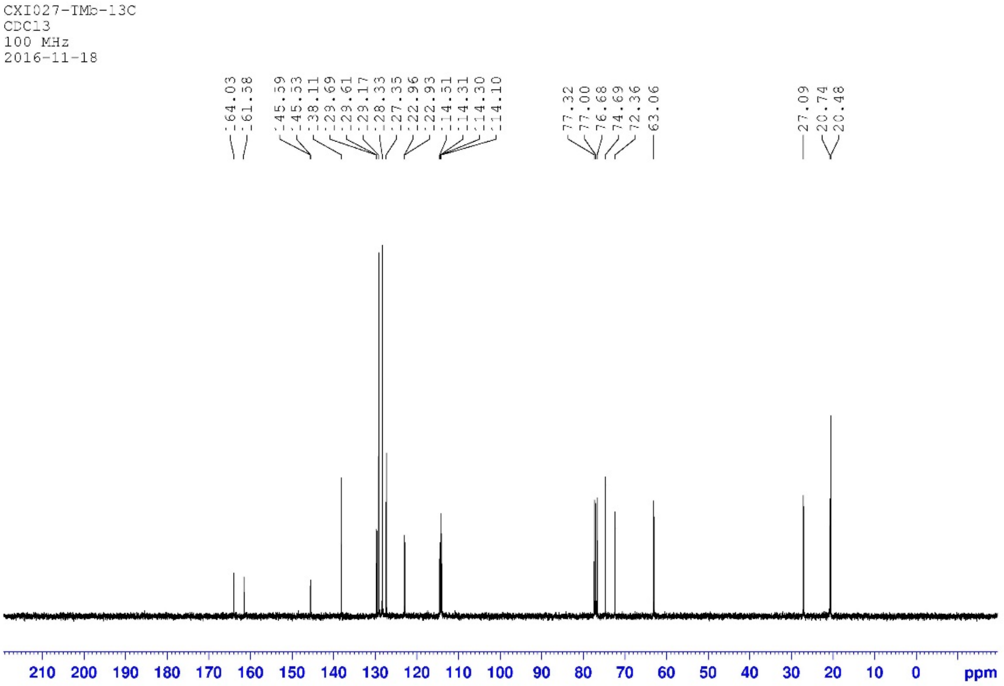


***Supplementary Figure 73.*** $^{13}$C NMR ($CDCl_3$) of compound **2a-2**.

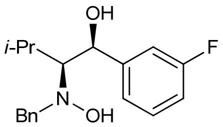


**2a-2**

[Lux® Amylose-1 column, 30 ºC, hexane/EtOH = 80/20 (v/v), 1.0 mL/min, 220 nm]

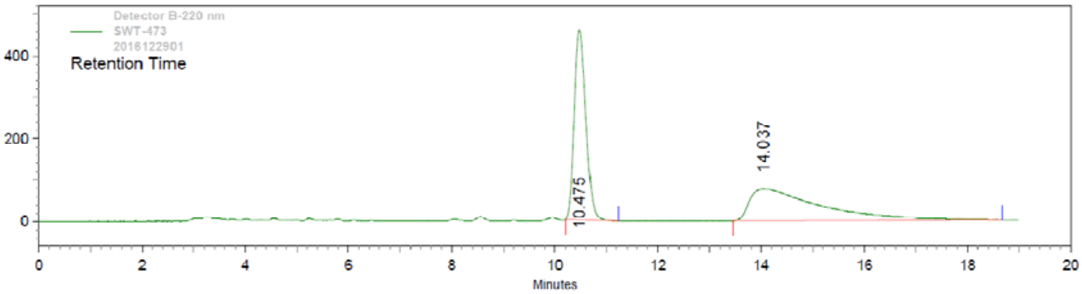


Detector B-220 nm

| Pk # | Retention Time | Area | Area Percent | Height |
|---|---|---|---|---|
| 1 | 10.475 | 7231071 | 50.17 | 459938 |
| 2 | 14.037 | 7182349 | 49.83 | 75585 |

***Supplementary Figure 74.*** Racemate of compound **2a-2**.

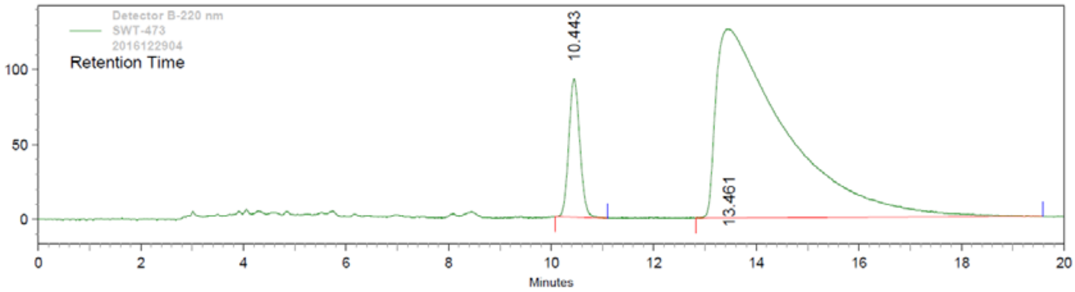


Detector B-220 nm

| Pk # | Retention Time | Area | Area Percent | Height |
|---|---|---|---|---|
| 1 | 10.443 | 1378338 | 10.39 | 92543 |
| 2 | 13.461 | 11888908 | 89.61 | 126400 |

***Supplementary Figure 75.*** Enantioenriched mixture of compound **2a-2**.

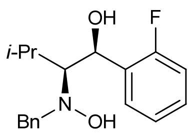


**2a-3**

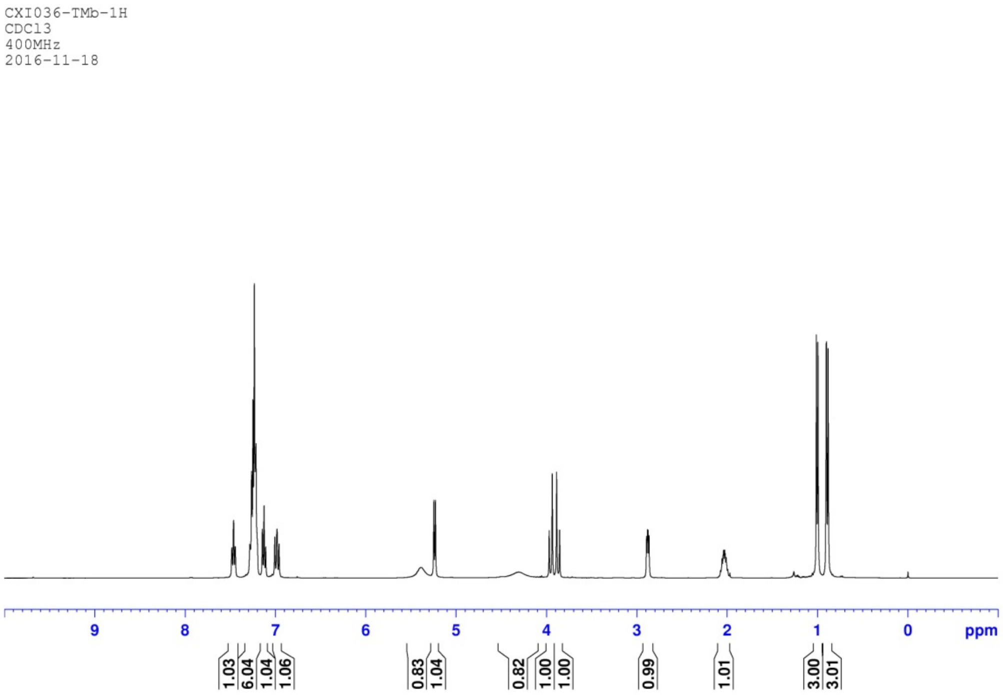


***Supplementary Figure 76.*** $^{1}$H NMR ($CDCl_3$) of compound **2a-3**.

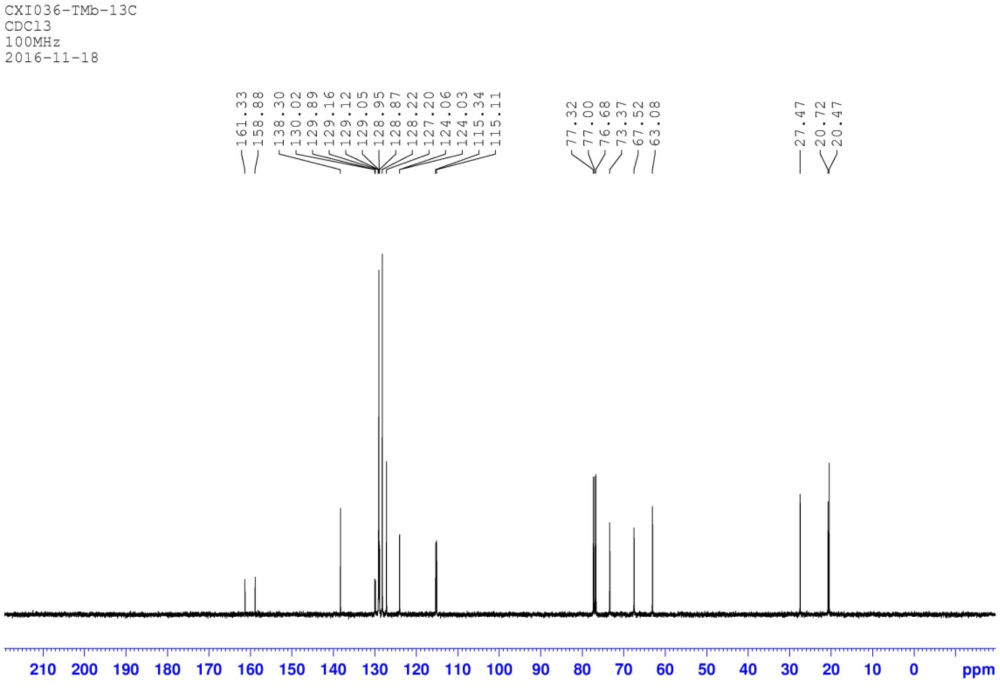


***Supplementary Figure 77.*** $^{13}$C NMR ($CDCl_3$) of compound **2a-3**.

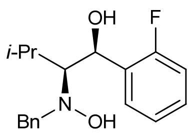

**2a-3**

[Lux® Amylose-1 column, 30 ºC, hexane/*i*-PrOH = 85/15 (v/v), 1.0 mL/min, 225 nm]

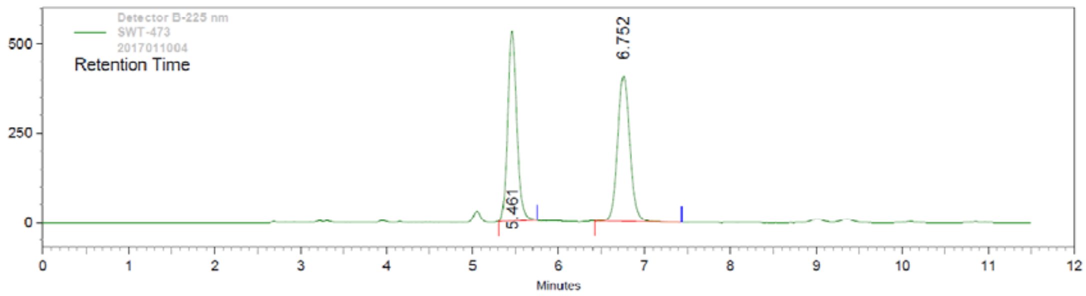


Detector B-225 nm

| Pk # | Retention Time | Area | Area Percent | Height |
|---|---|---|---|---|
| 1 | 5.461 | 3945277 | 50.18 | 531067 |
| 2 | 6.752 | 3916467 | 49.82 | 405650 |

***Supplementary Figure 78.*** Racemate of compound **2a-3**.

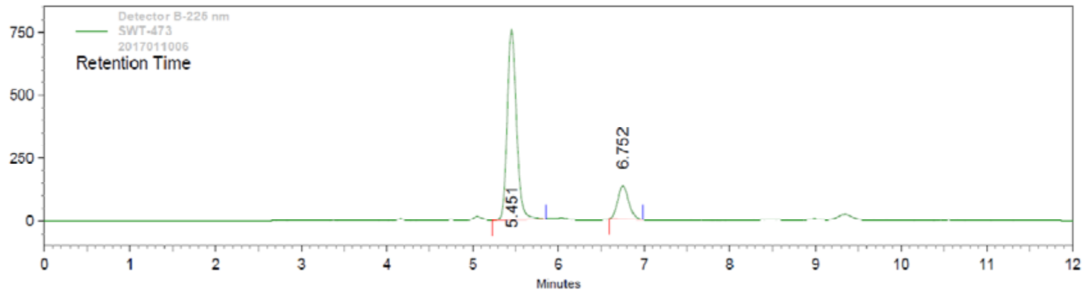


Detector B-225 nm

| Pk # | Retention Time | Area | Area Percent | Height |
|---|---|---|---|---|
| 1 | 5.451 | 5846144 | 82.85 | 757459 |
| 2 | 6.752 | 1210234 | 17.15 | 132003 |

***Supplementary Figure 79.*** Enantioenriched mixture of compound **2a-3**.

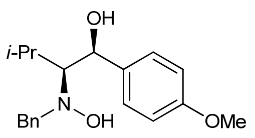


**2a-4**

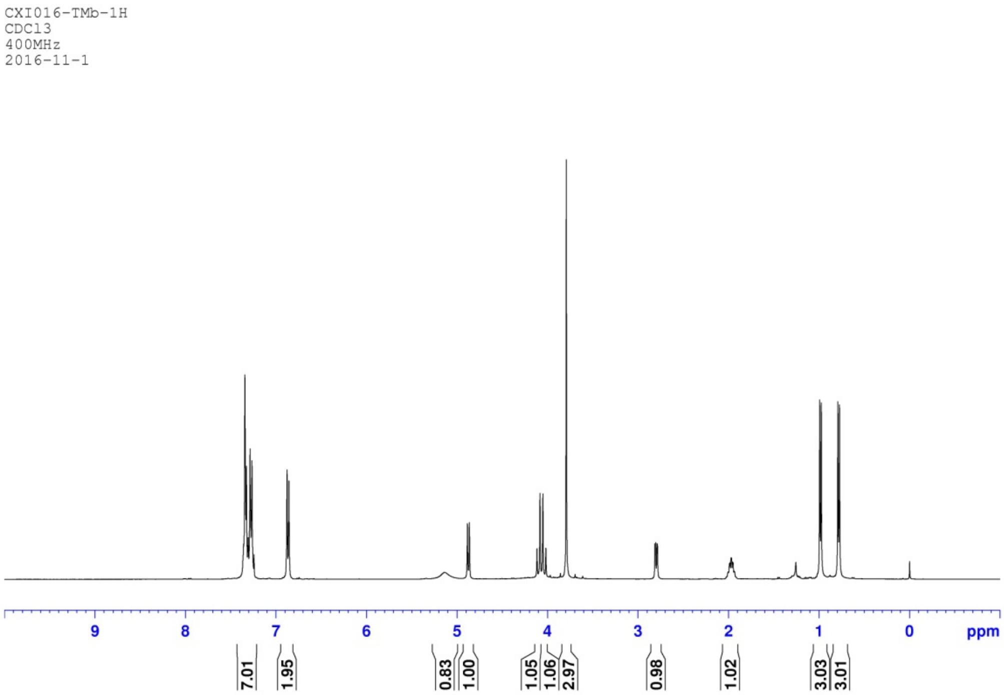


***Supplementary Figure 80.*** $^{1}$H NMR ($CDCl_3$) of compound **2a-4**.

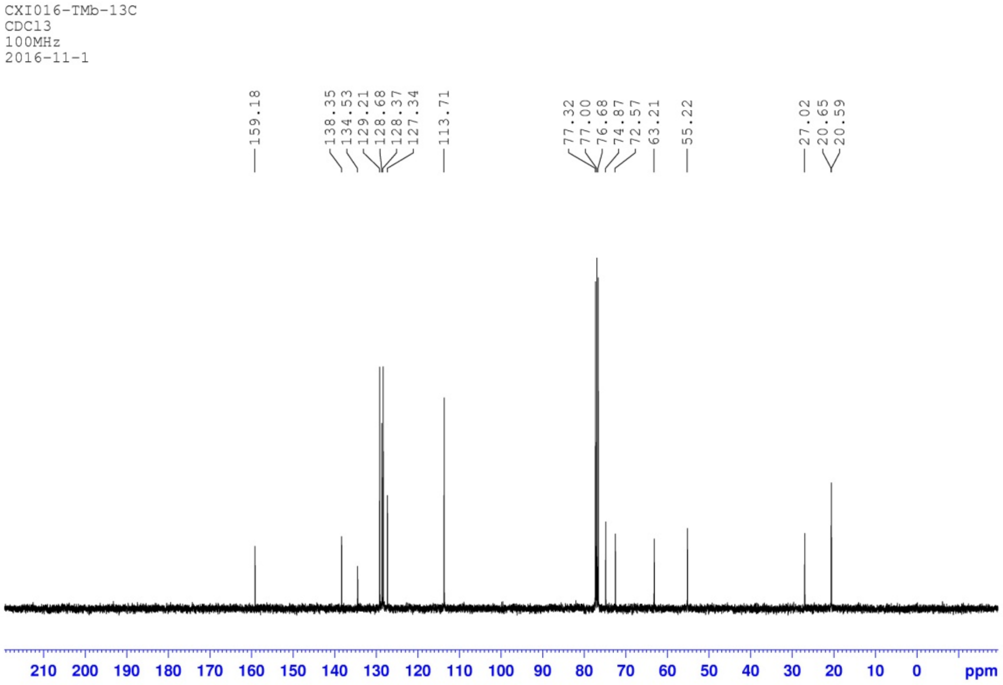


***Supplementary Figure 81.*** $^{13}$C NMR ($CDCl_3$) of compound **2a-4**.

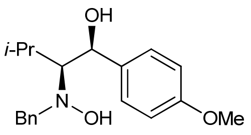


**2a-4**

[Lux® Amylose-1 column, 30 ºC, hexane/EtOH = 80/20 (v/v), 1.0 mL/min, 225 nm]

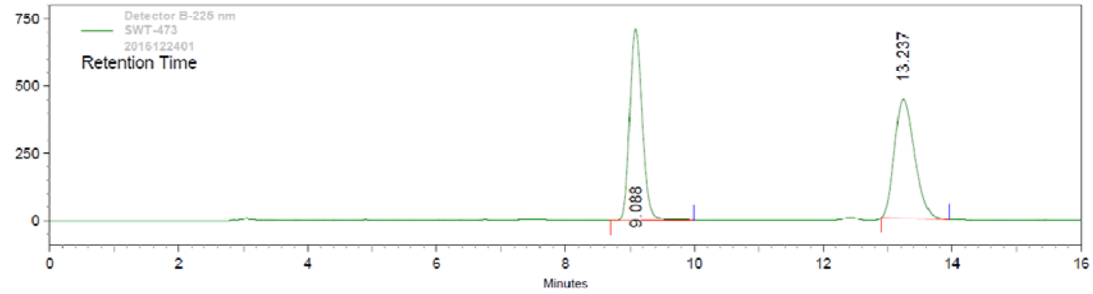


Detector B-225 nm

| Pk # | Retention Time | Area | Area Percent | Height |
|---|---|---|---|---|
| 1 | 9.088 | 9548692 | 50.00 | 710501 |
| 2 | 13.237 | 9549502 | 50.00 | 442468 |

***Supplementary Figure 82.*** Racemate of compound **2a-4**.

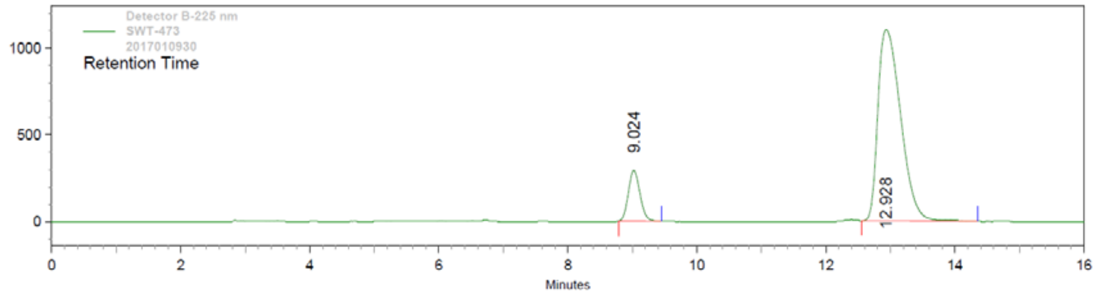


Detector B-225 nm

| Pk # | Retention Time | Area | Area Percent | Height |
|---|---|---|---|---|
| 1 | 9.024 | 3669867 | 11.95 | 293503 |
| 2 | 12.928 | 27039793 | 88.05 | 1104125 |

***Supplementary Figure 83.*** Enantioenriched mixture of compound **2a-4**.

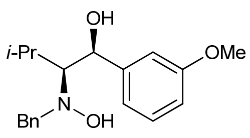


**2a-5**

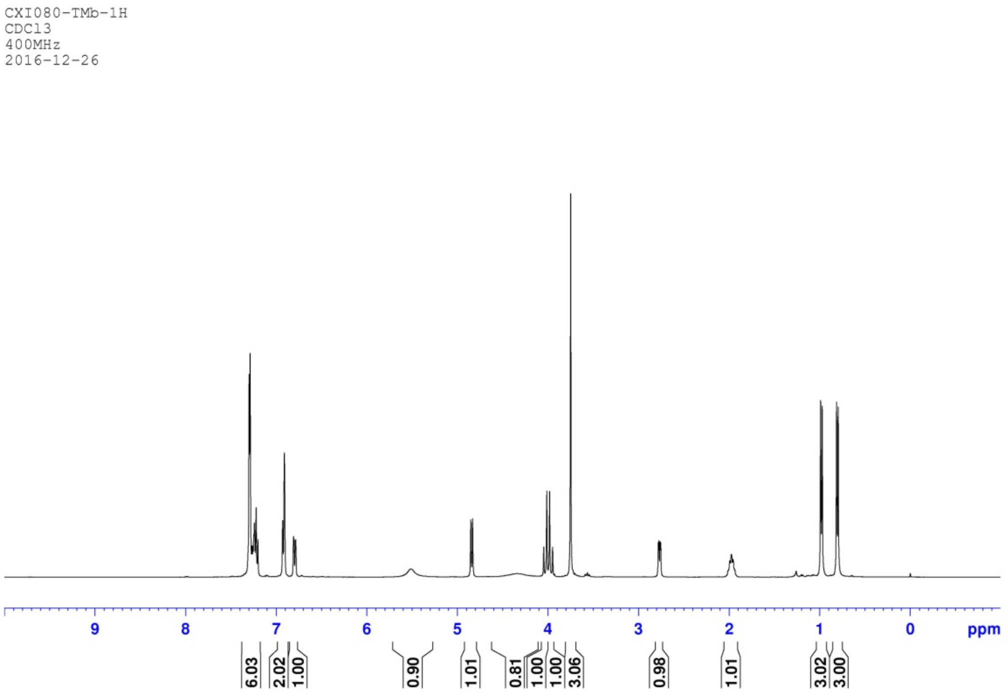


***Supplementary Figure 84.*** $^{1}$H NMR ($CDCl_3$) of compound **2a-5**.

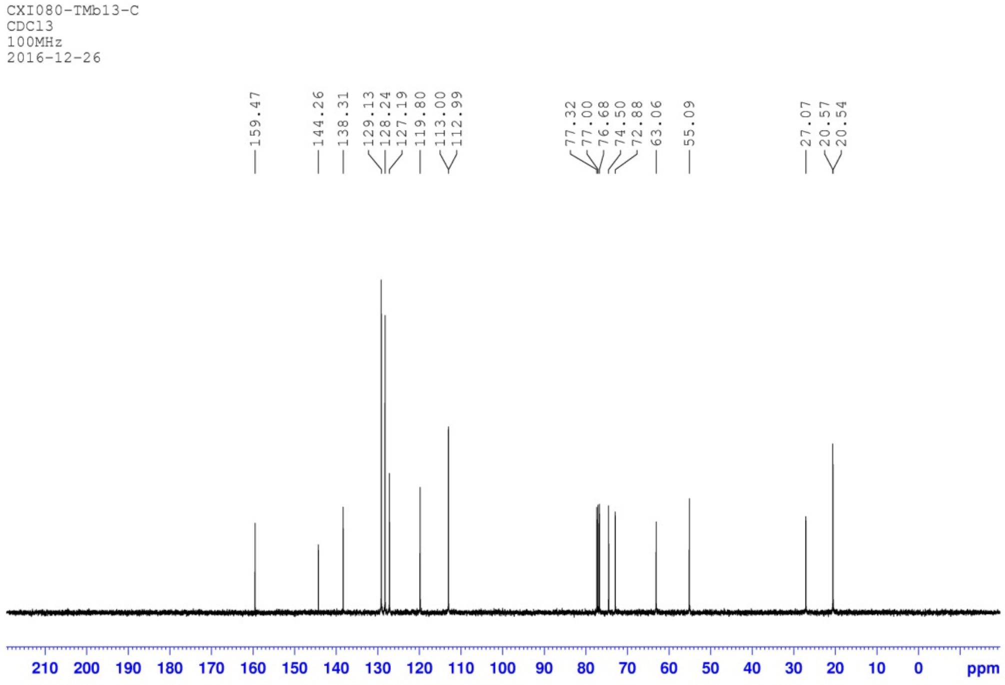


***Supplementary Figure 85.*** $^{13}$C NMR ($CDCl_3$) of compound **2a-5**.

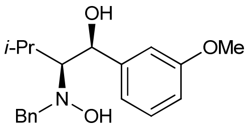


**2a-5**

[Lux® Amylose-1 column, 30 ºC, hexane/EtOH = 80/20 (v/v), 1.0 mL/min, 220 nm]

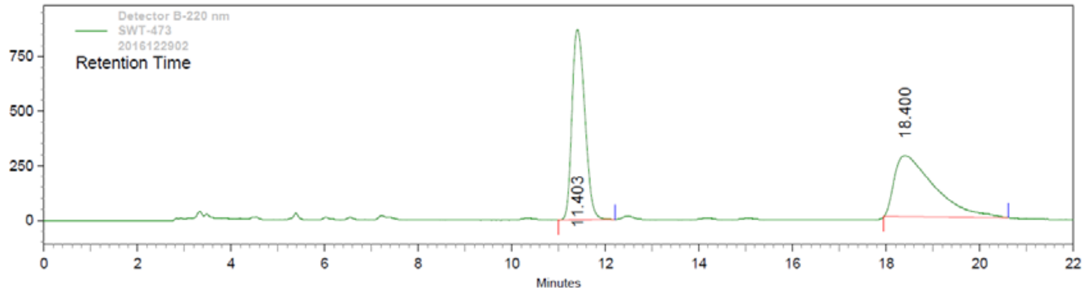


Detector B-220 nm

| Pk # | Retention Time | Area | Area Percent | Height |
|---|---|---|---|---|
| 1 | 11.403 | 16635941 | 50.00 | 872103 |
| 2 | 18.400 | 16639232 | 50.00 | 280416 |

***Supplementary Figure 86.*** Racemate of compound **2a-5**.

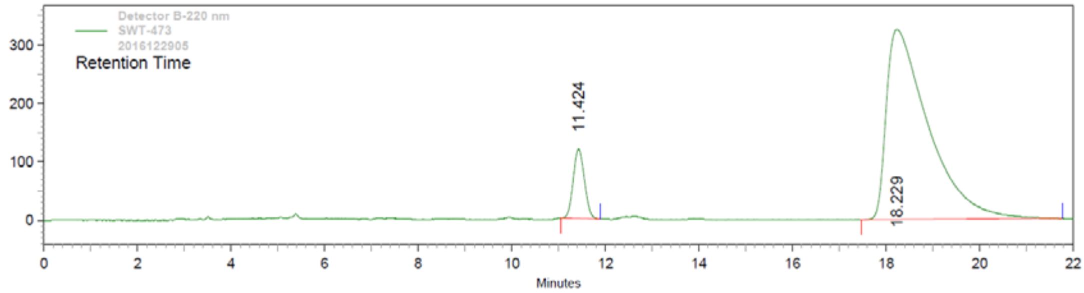


Detector B-220 nm

| Pk # | Retention Time | Area | Area Percent | Height |
|---|---|---|---|---|
| 1 | 11.424 | 1966910 | 8.83 | 118589 |
| 2 | 18.229 | 20303561 | 91.17 | 326219 |

***Supplementary Figure 87.*** Enantioenriched mixture of compound **2a-5**.

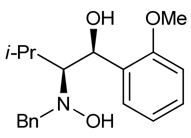


**2a-6**

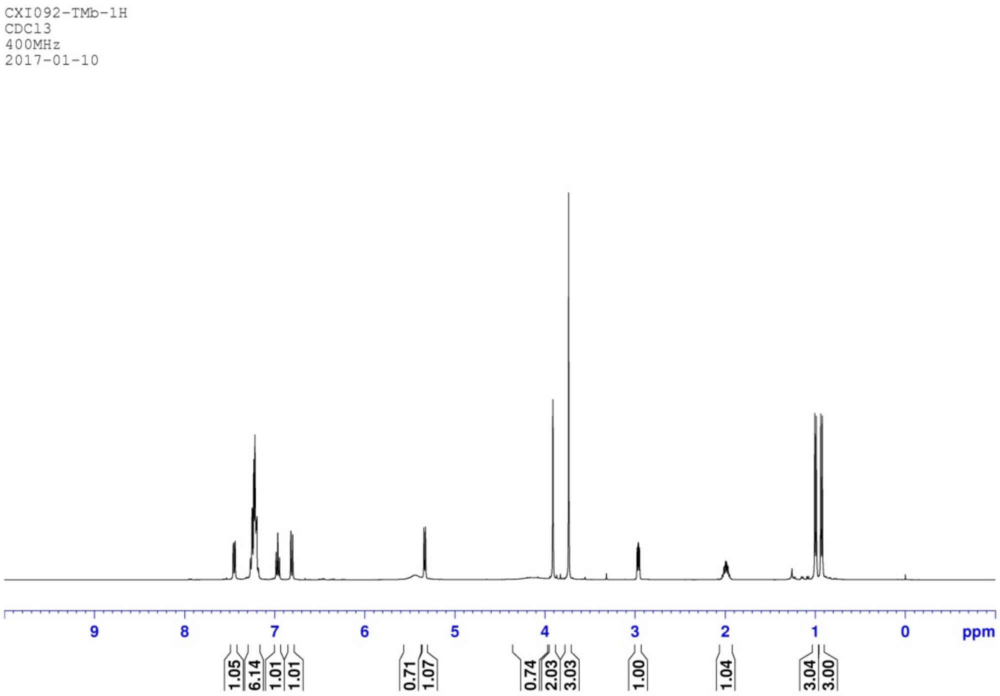


***Supplementary Figure 88.*** $^{1}$H NMR ($CDCl_3$) of compound **2a-6**.

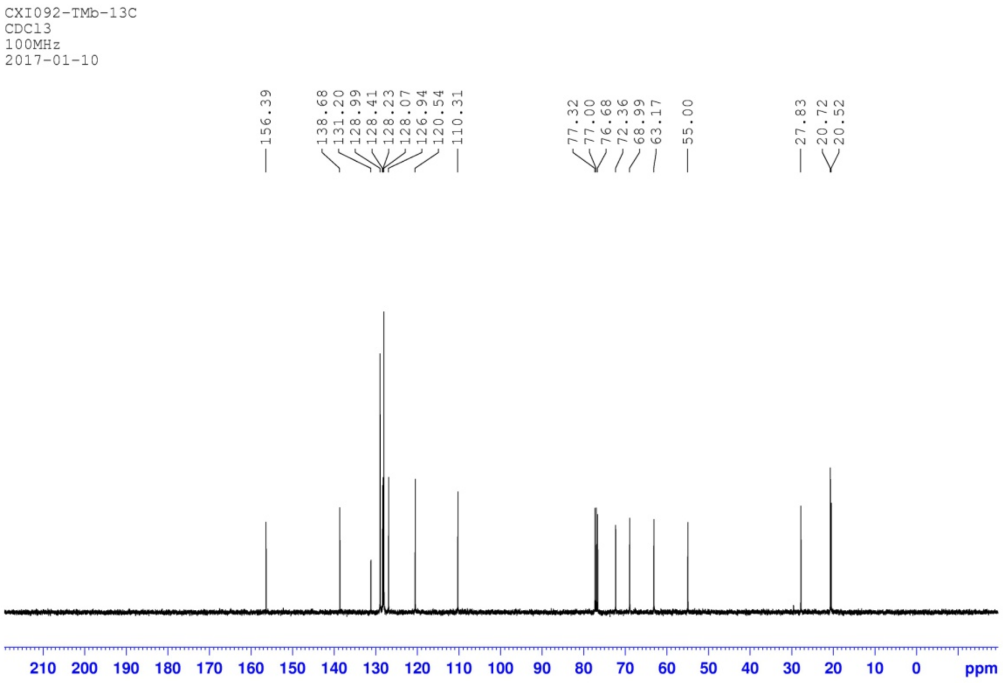


***Supplementary Figure 89.*** $^{13}$C NMR ($CDCl_3$) of compound **2a-6**.

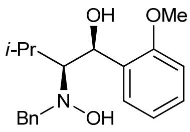


**2a-6**

[Lux® Amylose-1 column, 30 ºC, hexane/EtOH = 85/15 (v/v), 1.0 mL/min, 230 nm]

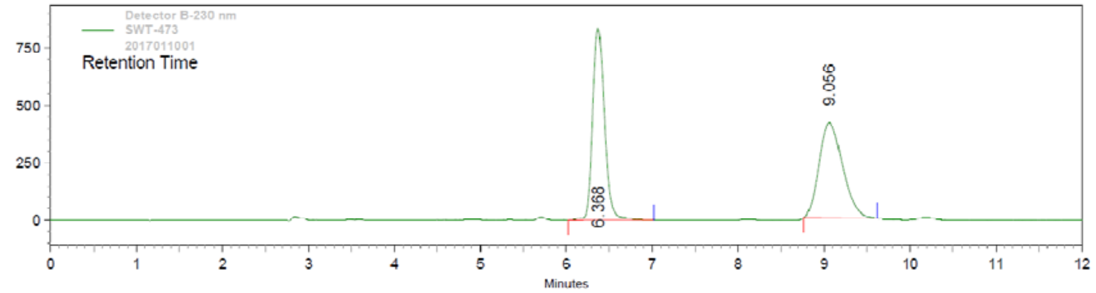


Detector B-230 nm

| Pk # | Retention Time | Area | Area Percent | Height |
|---|---|---|---|---|
| 1 | 6.368 | 7826096 | 49.80 | 832193 |
| 2 | 9.056 | 7888798 | 50.20 | 412285 |

***Supplementary Figure 90.*** Racemate of compound **2a-6**.

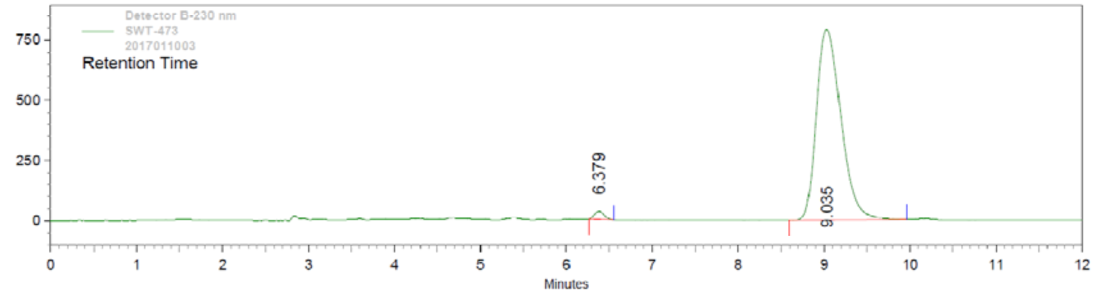


Detector B-230 nm

| Pk # | Retention Time | Area | Area Percent | Height |
|---|---|---|---|---|
| 1 | 6.379 | 246015 | 1.53 | 32516 |
| 2 | 9.035 | 15809419 | 98.47 | 793695 |

***Supplementary Figure 91.*** Enantioenriched mixture of compound **2a-6**.

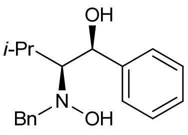


**2a-7**

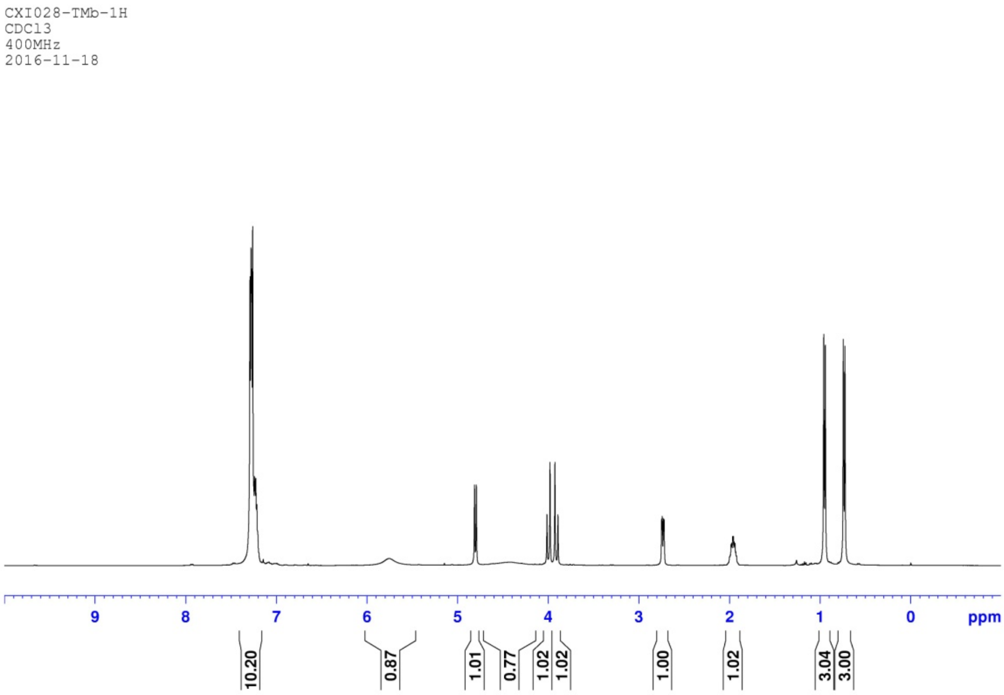


***Supplementary Figure 92.*** $^{1}$H NMR ($CDCl_3$) of compound **2a-7**.

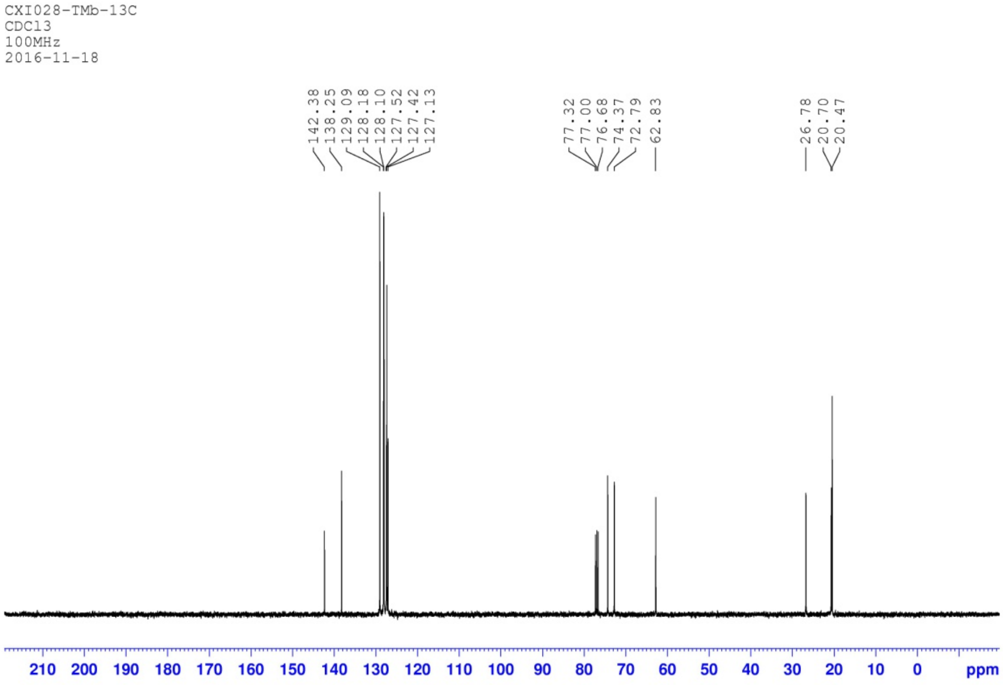


***Supplementary Figure 93.*** $^{13}$C NMR ($CDCl_3$) of compound **2a-7**.

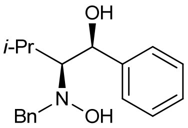


**2a-7**

[Lux® Amylose-1 column, 30 ºC, hexane/EtOH = 80/20 (v/v), 1.0 mL/min, 220 nm]

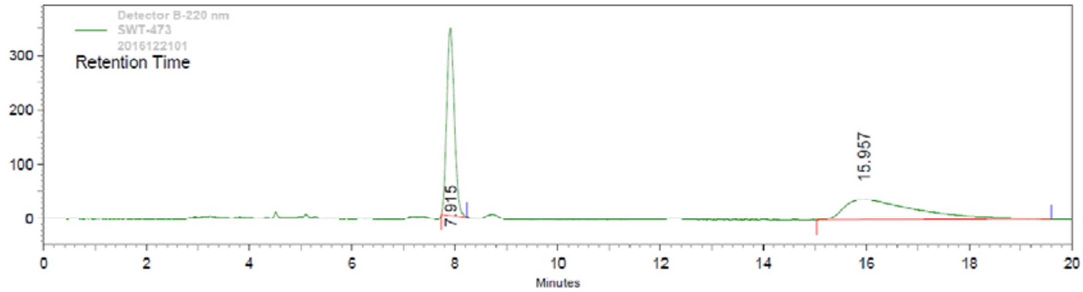


Detector B-220 nm

| Pk # | Retention Time | Area | Area Percent | Height |
|---|---|---|---|---|
| 1 | 7.915 | 3628323 | 50.05 | 343632 |
| 2 | 15.957 | 3620498 | 49.95 | 37401 |

***Supplementary Figure 94.*** Racemate of compound **2a-7**.

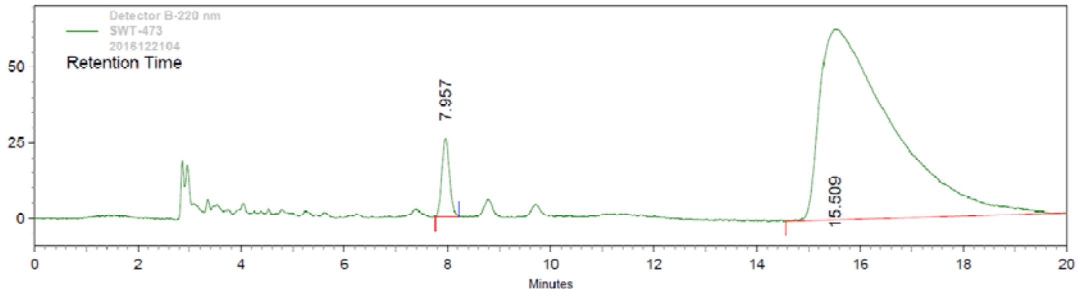


Detector B-220 nm

| Pk # | Retention Time | Area | Area Percent | Height |
|---|---|---|---|---|
| 1 | 7.957 | 268883 | 4.21 | 25726 |
| 2 | 15.509 | 6120301 | 95.79 | 63108 |

***Supplementary Figure 95.*** Enantioenriched mixture of compound **2a-7**.

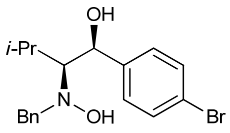


**2a-8**

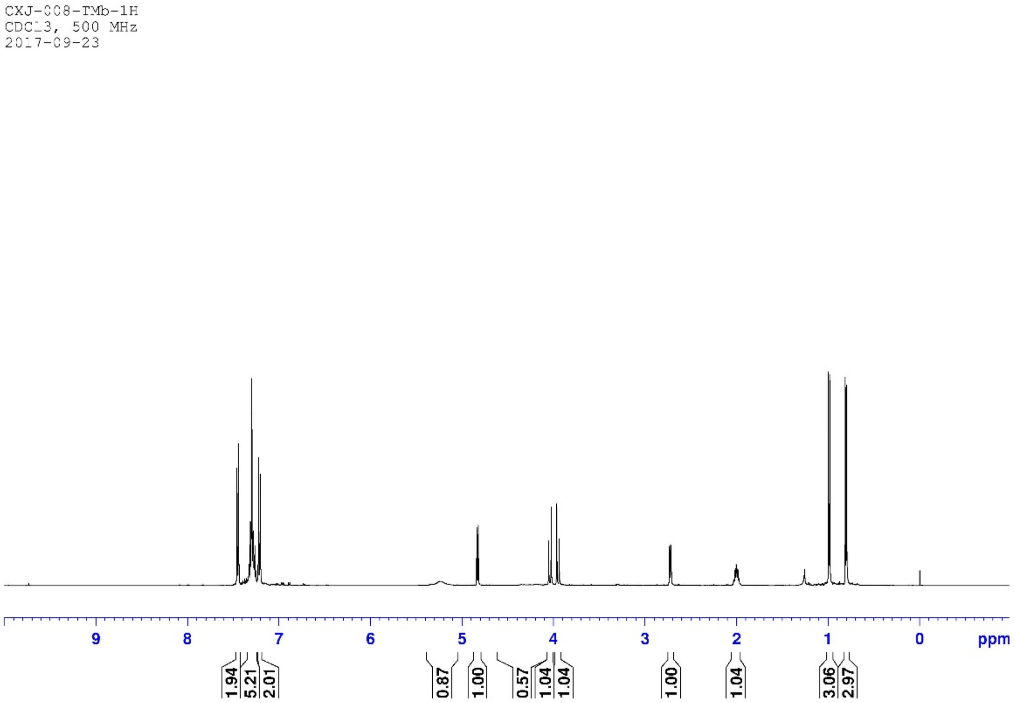


***Supplementary Figure 96.*** $^{1}$H NMR ($CDCl_3$) of compound **2a-8**.

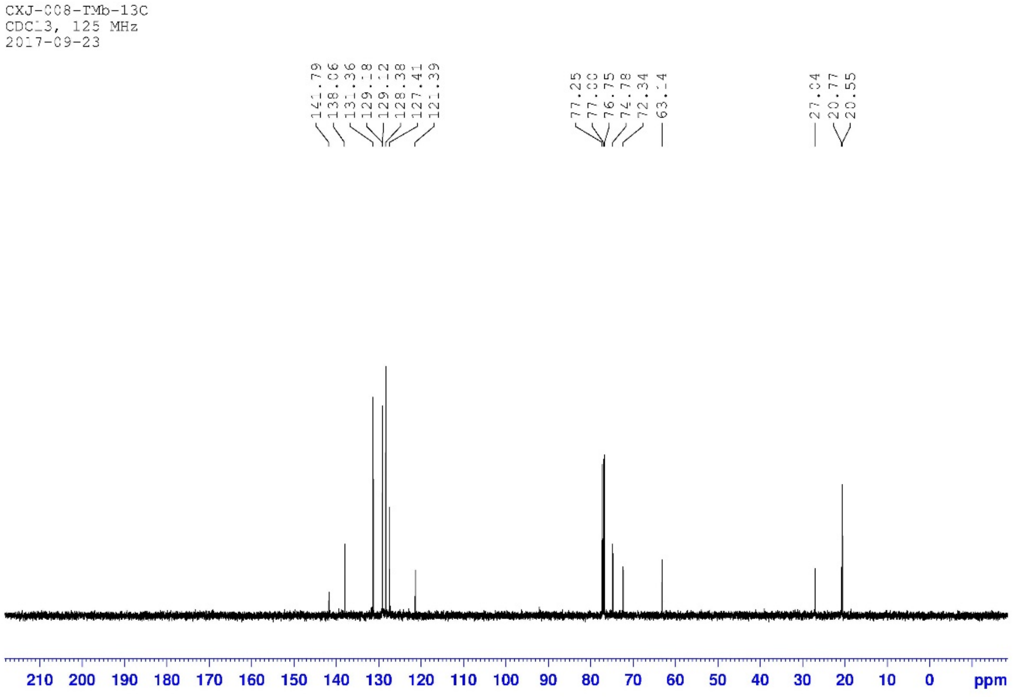


***Supplementary Figure 97.*** $^{13}$C NMR ($CDCl_3$) of compound **2a-8**.

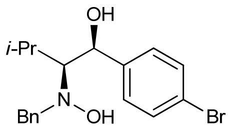

**2a-8**

[Lux® Amylose-1 column, 30 ºC, hexane/EtOH = 85/15 (v/v), 1.0 mL/min, 220 nm]

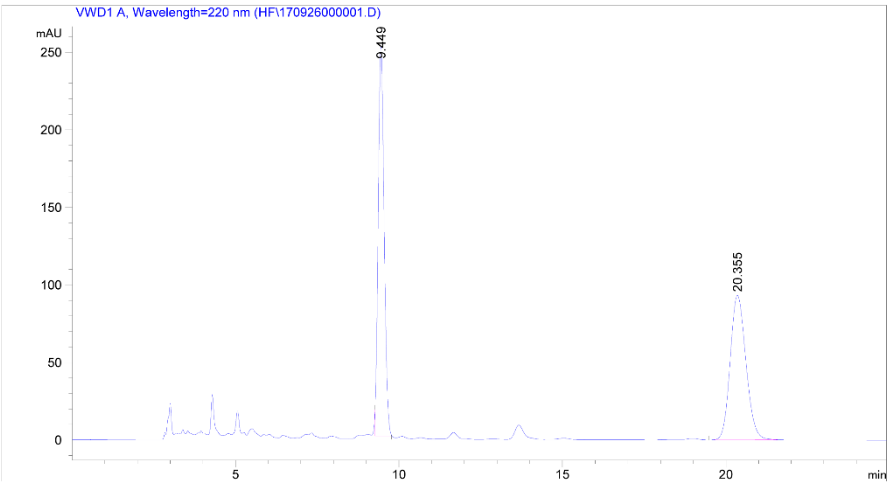


| Pk # | Retention Time [min] | Area [mAU*s] | Height [mAU] | Area Percent % |
|---|---|---|---|---|
| 1 | 9.449 | 3188.23486 | 251.30400 | 50.5575 |
| 2 | 20.355 | 3117.92554 | 93.22905 | 49.4425 |
| Totals: | | 6306.16040 | 344.53305 | 100.0000 |

***Supplementary Figure 98.*** Racemate of compound **2a-8**.

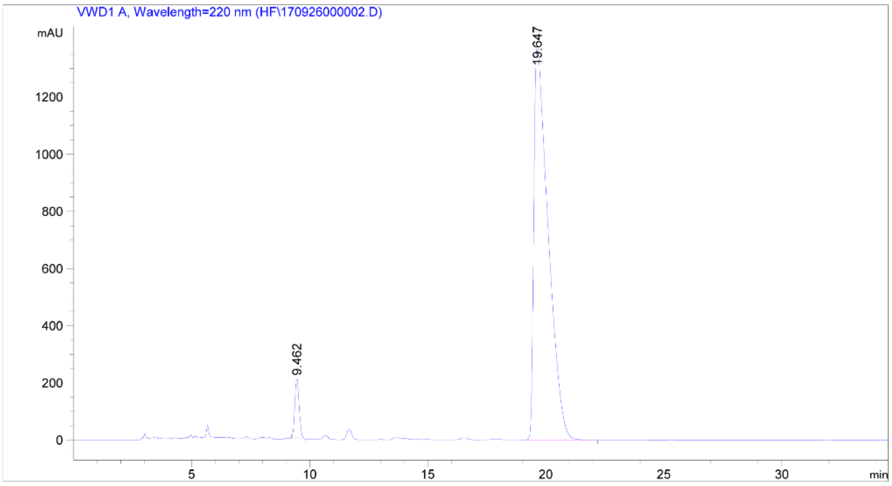


| Pk # | Retention Time [min] | Area [mAU*s] | Height [mAU] | Area Percent % |
|---|---|---|---|---|
| 1 | 9.462 | 2699.80225 | 209.19240 | 4.4457 |
| 2 | 19.647 | 5.80293e4 | 1379.35938 | 95.5543 |
| Totals: | | 6.07291e4 | 1588.55177 | 100.0000 |

***Supplementary Figure 99.*** Enantioenriched mixture of compound **2a-8**.

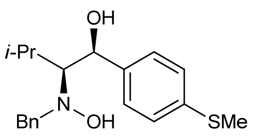


**2a-9**

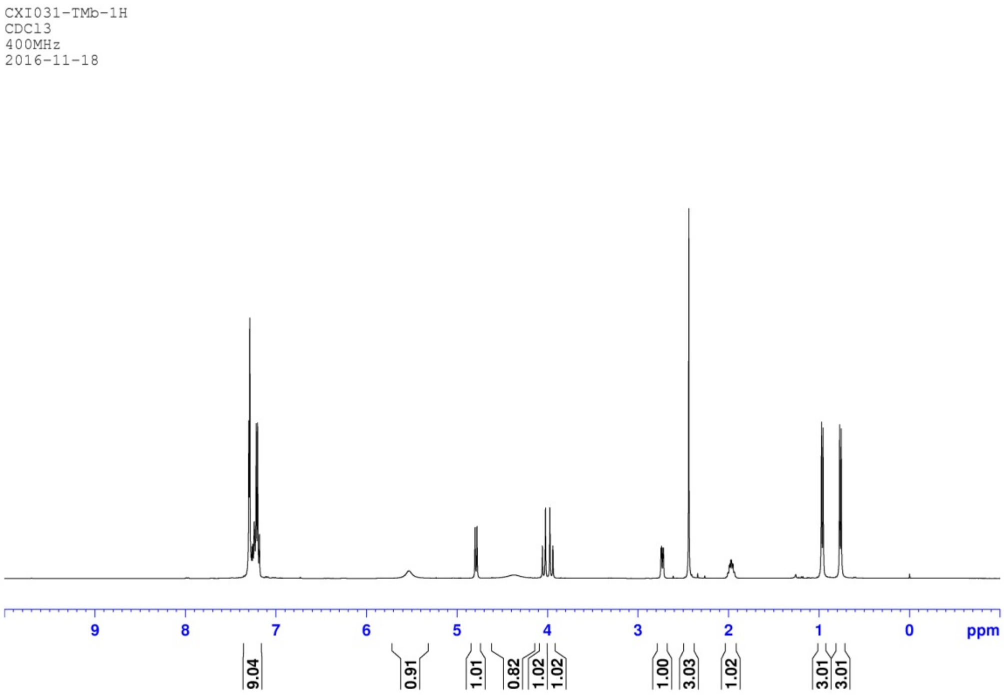


***Supplementary Figure 100.*** $^{1}$H NMR ($CDCl_3$) of compound **2a-9**.

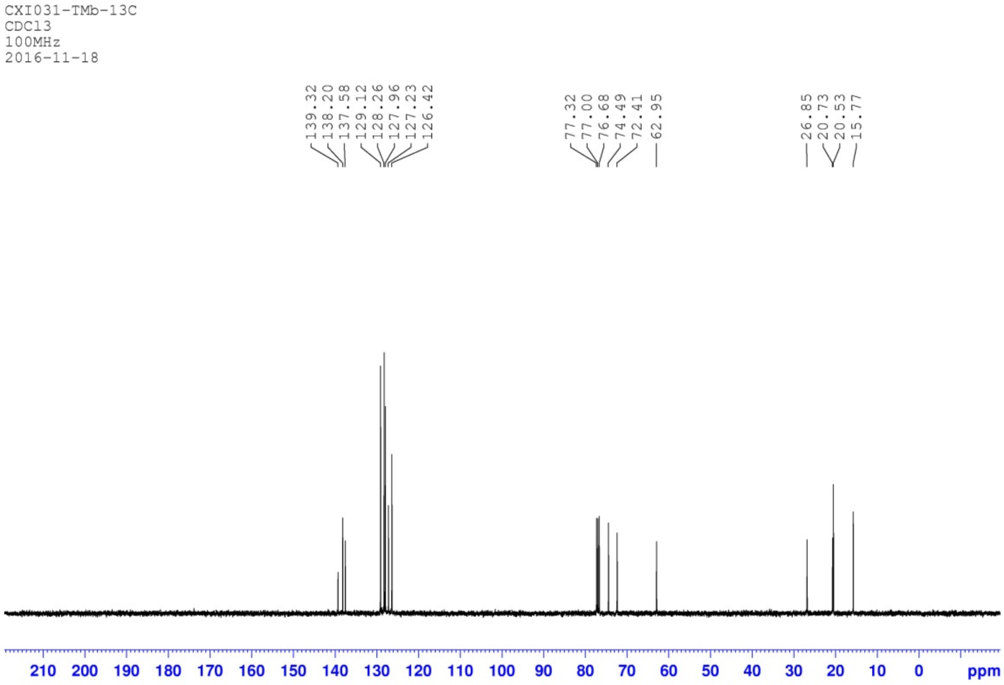


***Supplementary Figure 101.*** $^{13}$C NMR ($CDCl_3$) of compound **2a-9**.

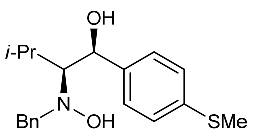

**2a-9**

[Lux® Amylose-1 column, 30 ºC, hexane/EtOH = 80/20 (v/v), 1.0 mL/min, 268 nm]

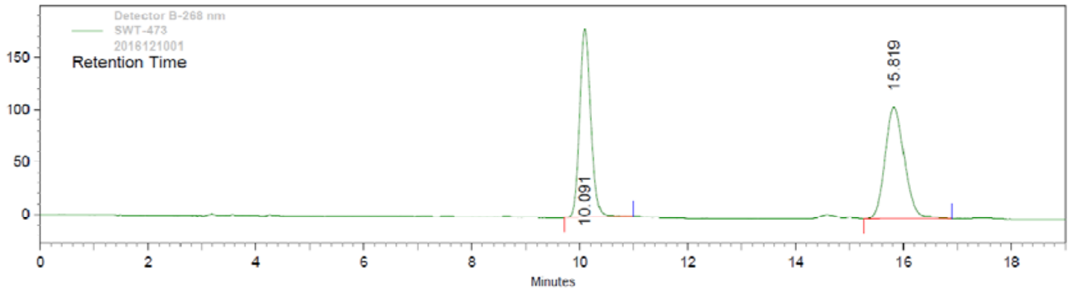


Detector B-268 nm

| Pk # | Retention Time | Area | Area Percent | Height |
|---|---|---|---|---|
| 1 | 10.091 | 2622062 | 49.96 | 179697 |
| 2 | 15.819 | 2626382 | 50.04 | 105748 |

***Supplementary Figure 102.*** Racemate of compound **2a-9**.

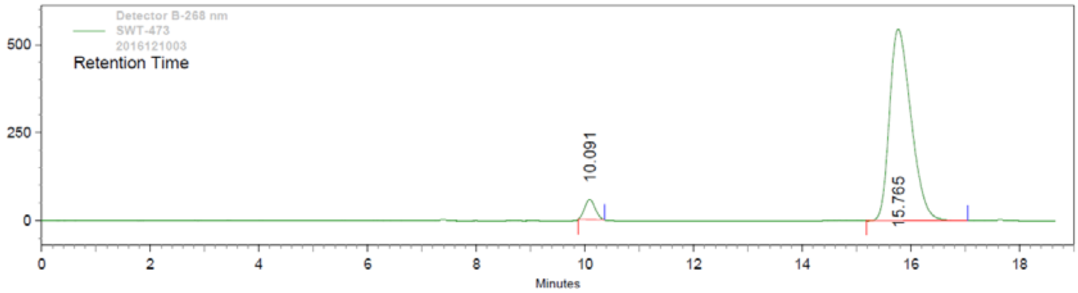


Detector B-268 nm

| Pk # | Retention Time | Area | Area Percent | Height |
|---|---|---|---|---|
| 1 | 10.091 | 755231 | 4.79 | 56414 |
| 2 | 15.765 | 14998789 | 95.21 | 545316 |

***Supplementary Figure 103.*** Enantioenriched mixture of compound **2a-9**.

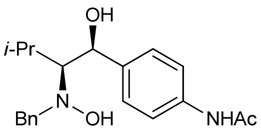


**2a-10**

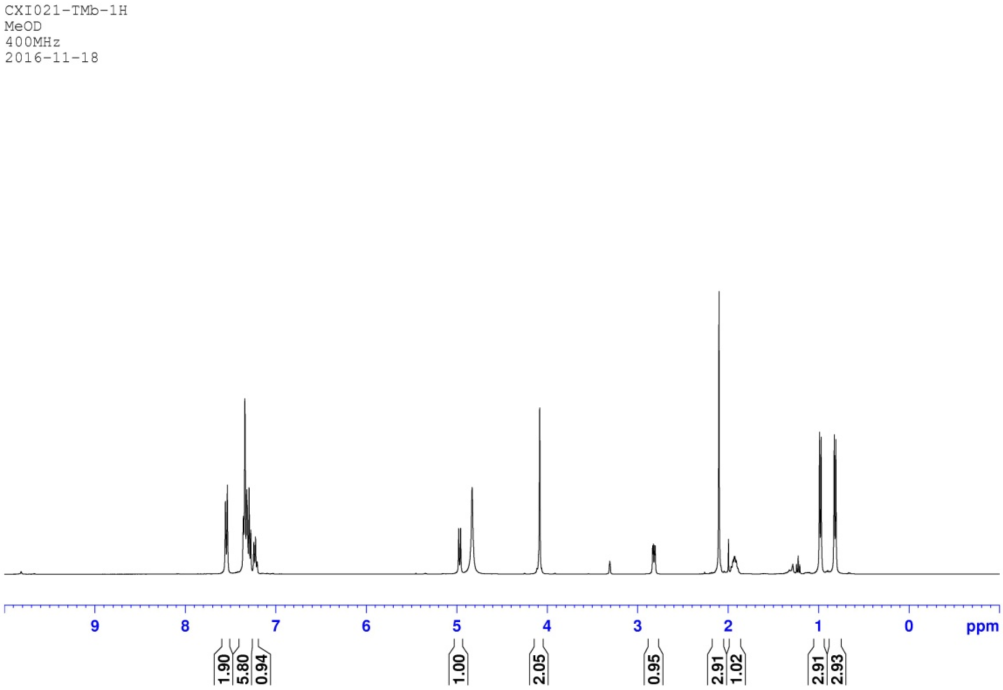


***Supplementary Figure 104.*** $^{1}$H NMR (MeOD) of compound **2a-10**.

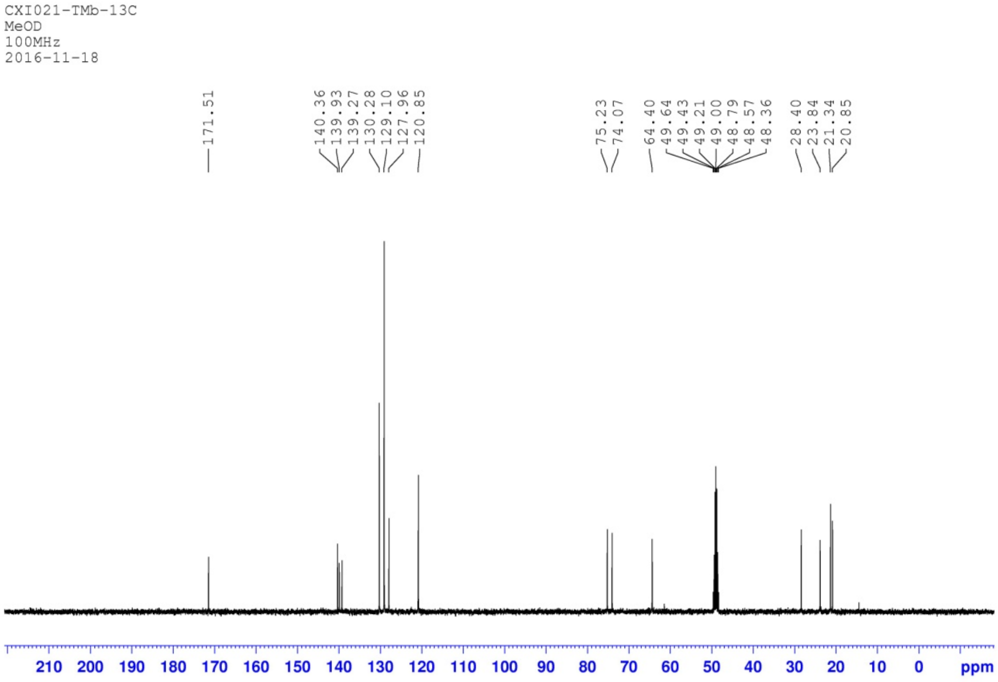


***Supplementary Figure 105.*** $^{13}$C NMR (MeOD) of compound **2a-10**.

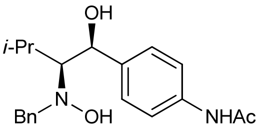


**2a-10**

[Lux® Amylose-1 column, 30 ºC, hexane/EtOH = 80/20 (v/v), 1.0 mL/min, 240 nm]

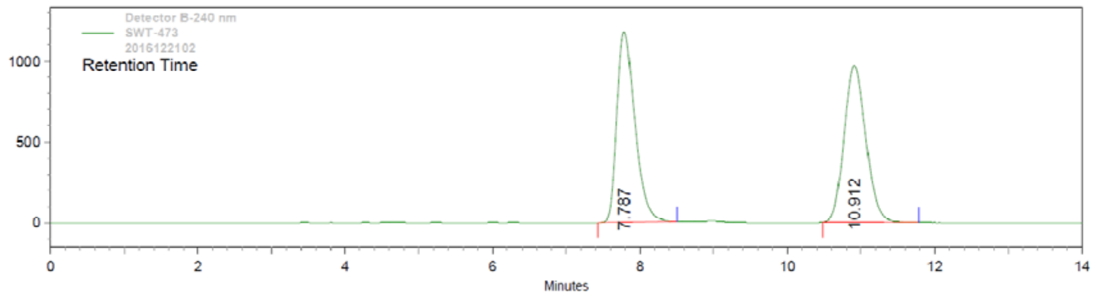


Detector B-240 nm

| Pk # | Retention Time | Area | Area Percent | Height |
|---|---|---|---|---|
| 1 | 7.787 | 20064807 | 49.95 | 1181029 |
| 2 | 10.912 | 20102171 | 50.05 | 970316 |

***Supplementary Figure 106.*** Racemate of compound **2a-10**.

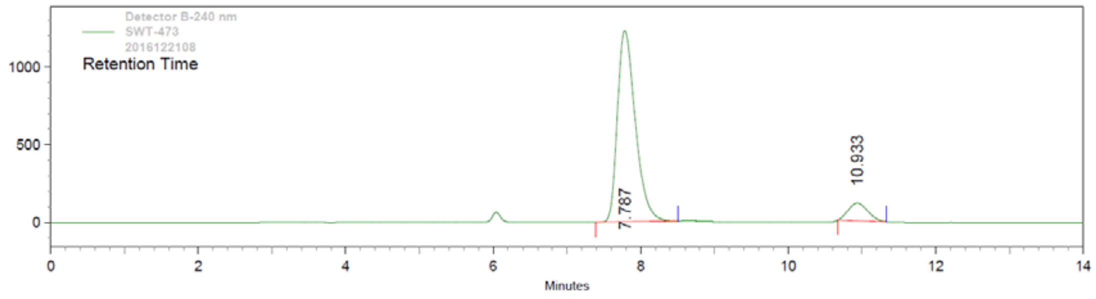


Detector B-240 nm

| Pk # | Retention Time | Area | Area Percent | Height |
|---|---|---|---|---|
| 1 | 7.787 | 20757686 | 91.02 | 1230972 |
| 2 | 10.933 | 2047304 | 8.98 | 111181 |

***Supplementary Figure 107.*** Enantioenriched mixture of compound **2a-10**.

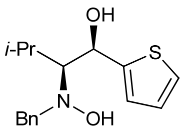

**2a-11**

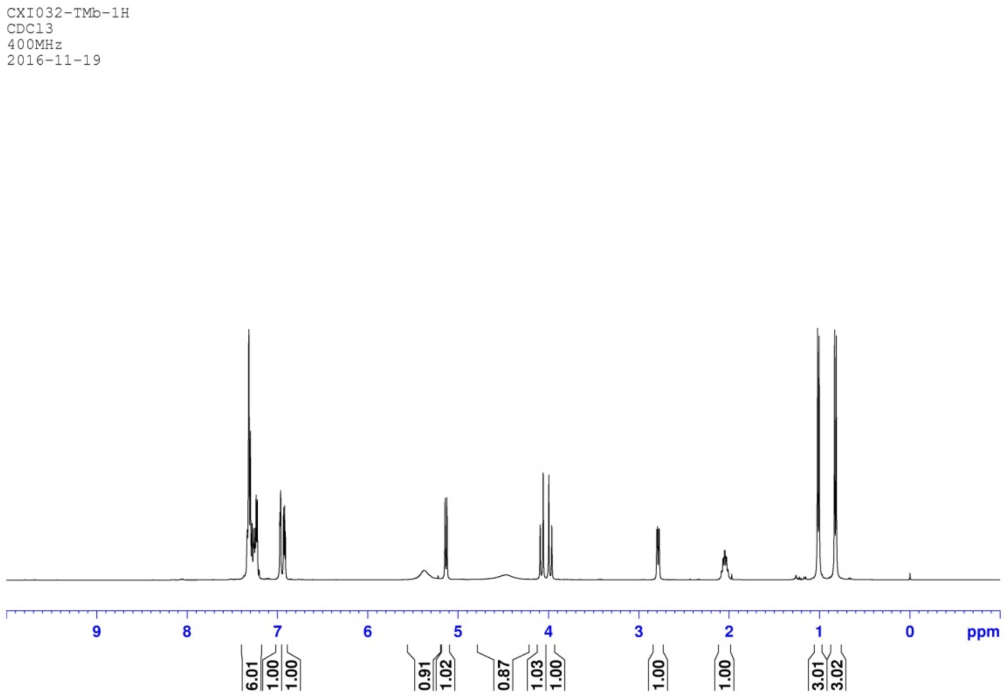


***Supplementary Figure 108.*** $^{1}$H NMR ($CDCl_3$) of compound **2a-11**.

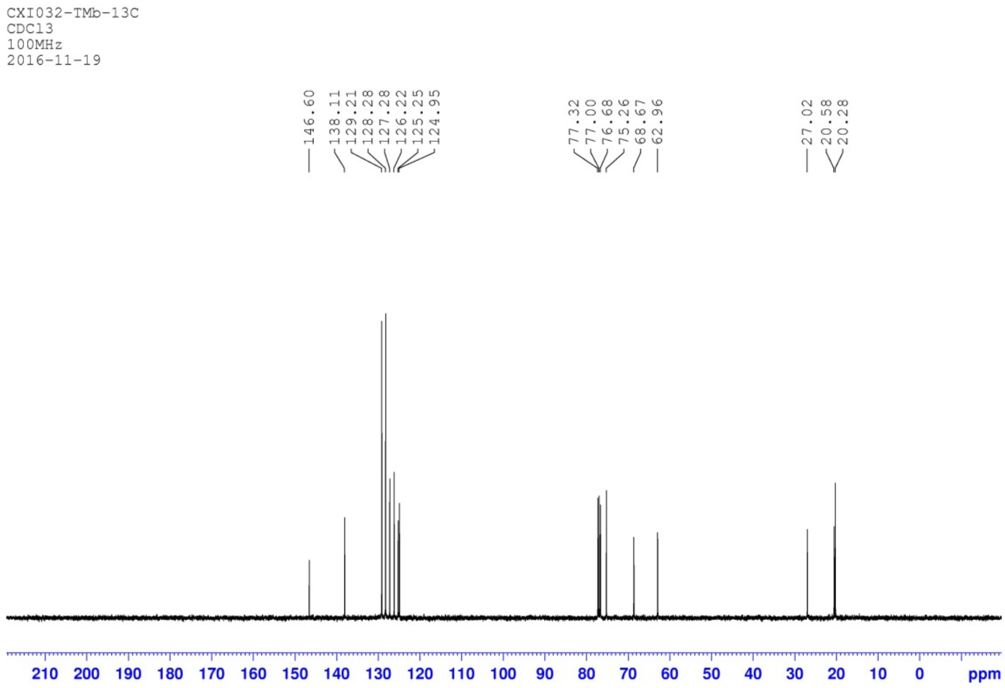


***Supplementary Figure 109.*** $^{13}$C NMR ($CDCl_3$) of compound **2a-11**.

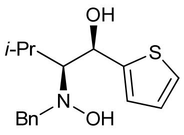

**2a-11**

[Lux® Amylose-1 column, 30 ºC, hexane/EtOH = 80/20 (v/v), 1.0 mL/min, 235 nm]

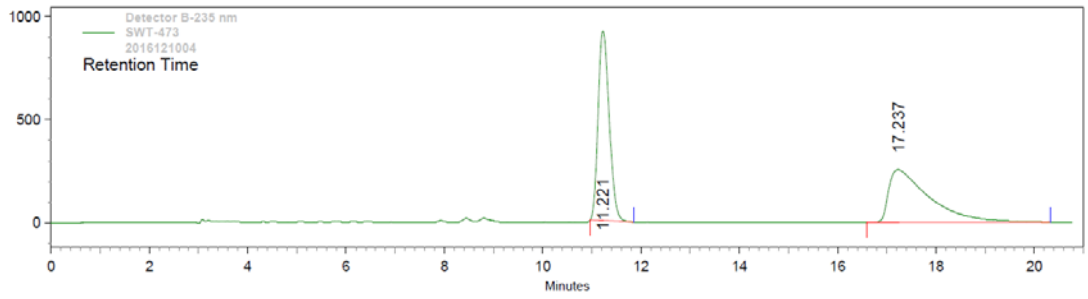


Detector B-235 nm

| Pk # | Retention Time | Area | Area Percent | Height |
|---|---|---|---|---|
| 1 | 11.221 | 14453906 | 50.16 | 920646 |
| 2 | 17.237 | 14361517 | 49.84 | 253102 |

***Supplementary Figure 110.*** Racemate of compound **2a-11**.

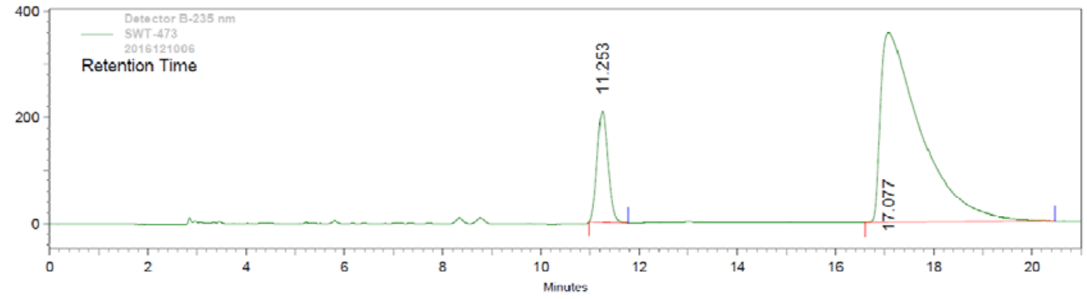


Detector B-235 nm

| Pk # | Retention Time | Area | Area Percent | Height |
|---|---|---|---|---|
| 1 | 11.253 | 3129608 | 13.41 | 207599 |
| 2 | 17.077 | 20201701 | 86.59 | 356242 |

***Supplementary Figure 111.*** Enantioenriched mixture of compound **2a-11**.

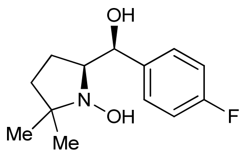


**2k-1** (major)

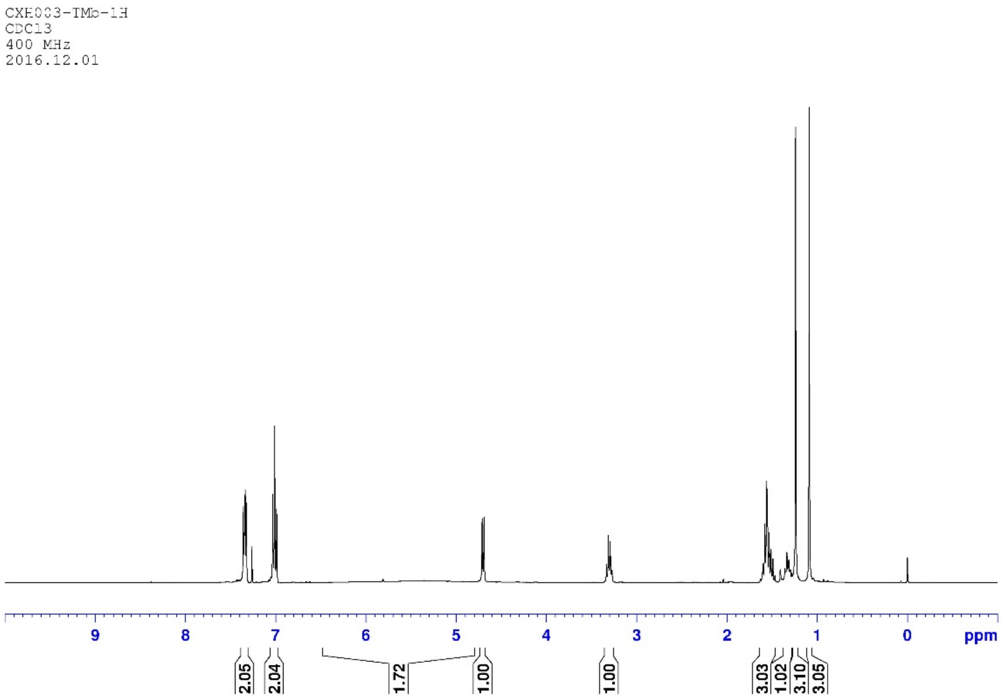


***Supplementary Figure 112.*** $^{1}$H NMR ($CDCl_3$) of compound **2k-1**.

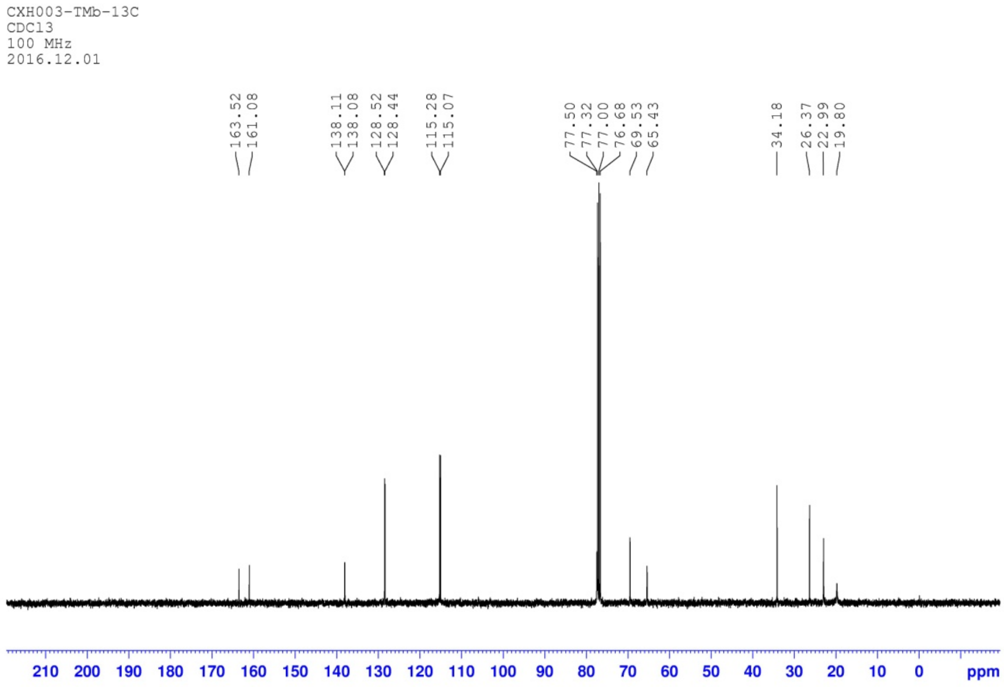


***Supplementary Figure 113.*** $^{13}$C NMR ($CDCl_3$) of compound **2k-1**.

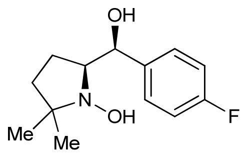

**2k-1** (major)

[Lux® Amylose-1 column, 30 ºC, hexane/EtOH = 80/20 (v/v), 1.0 mL/min, 270 nm]

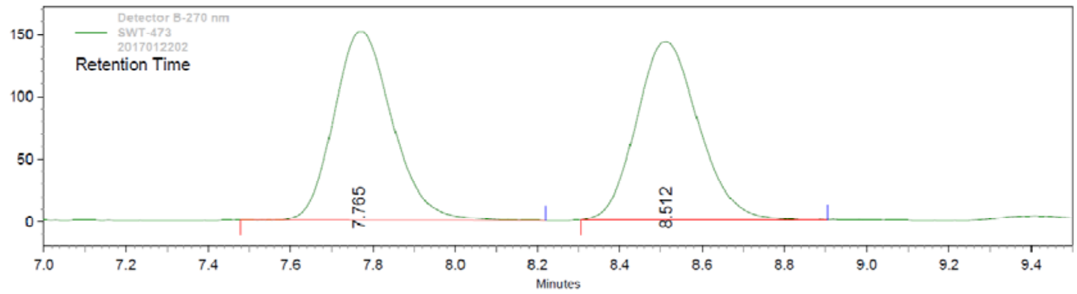


Detector B-270 nm

| Pk # | Retention Time | Area | Area Percent | Height |
|---|---|---|---|---|
| 1 | 7.765 | 1489600 | 49.97 | 151484 |
| 2 | 8.512 | 1491152 | 50.03 | 142705 |

***Supplementary Figure 114.*** Racemate of compound **2k-1**.

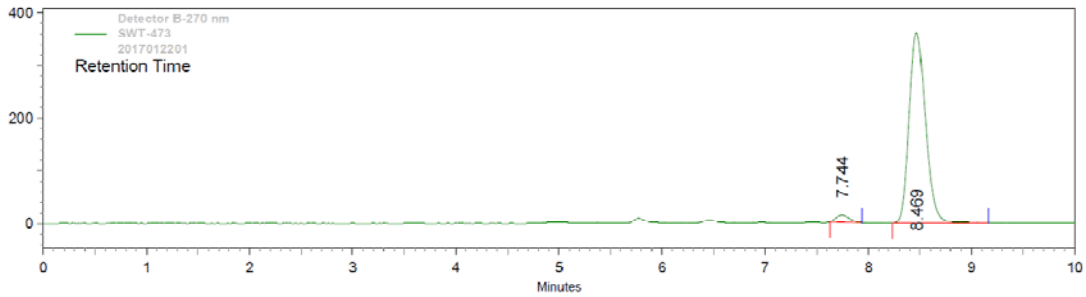


Detector B-270 nm

| Pk # | Retention Time | Area | Area Percent | Height |
|---|---|---|---|---|
| 1 | 7.744 | 121378 | 2.97 | 13982 |
| 2 | 8.469 | 3970612 | 97.03 | 362665 |

***Supplementary Figure 115.*** Enantioenriched mixture of compound **2k-1**.

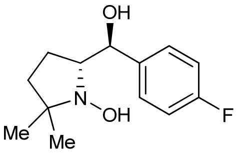


diastereomer of **2k-1** (minor)

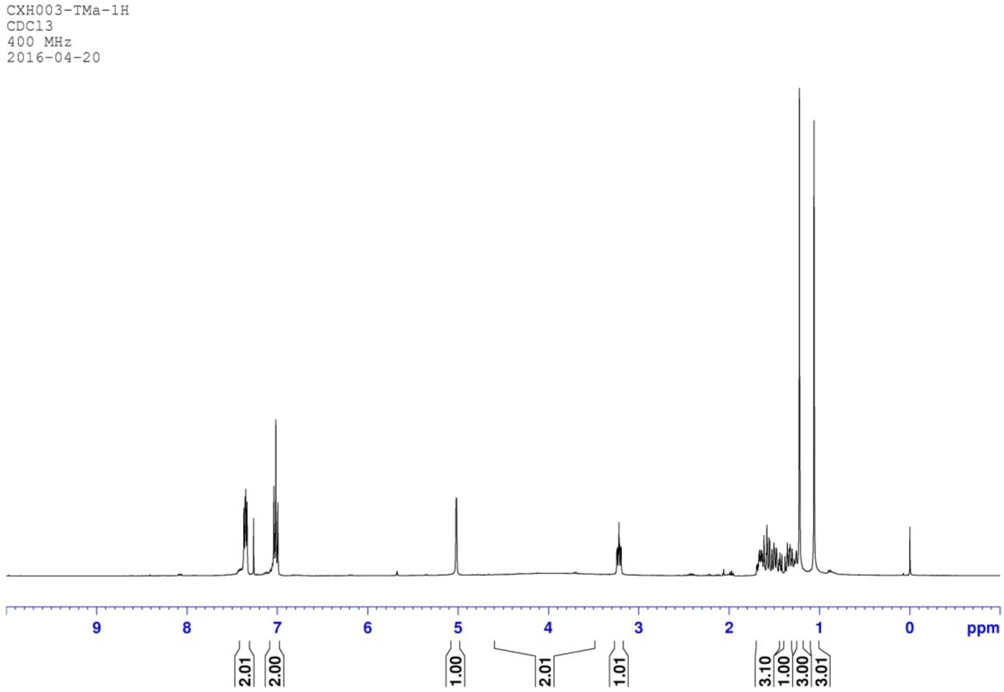


***Supplementary Figure 116.*** $^{1}$H NMR ($CDCl_3$) of the diastereomer of **2k-1**.

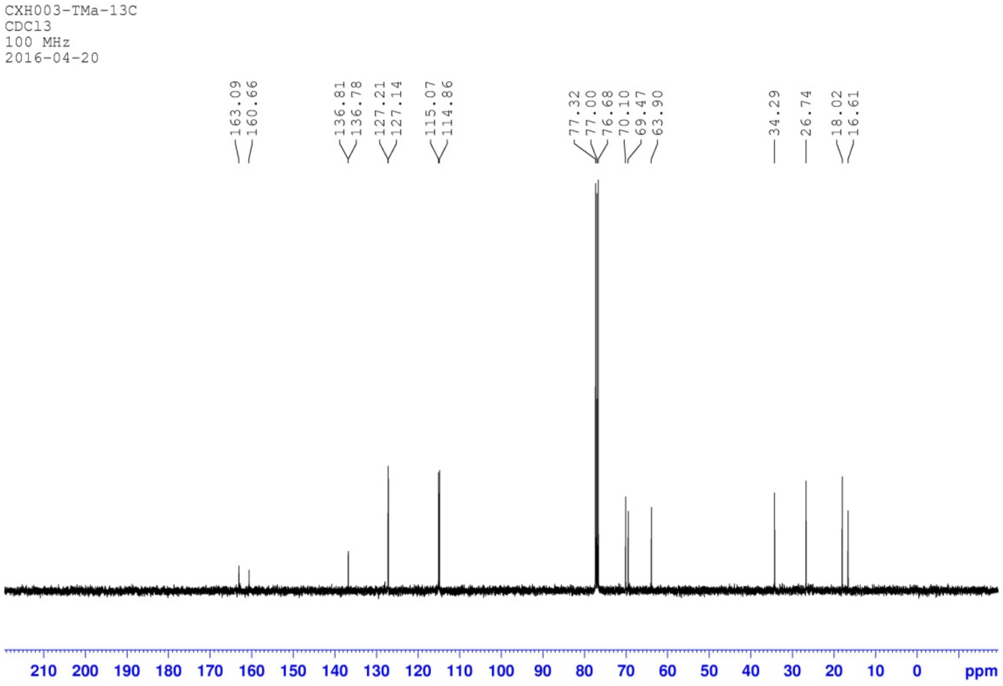


***Supplementary Figure 117.*** $^{13}$C NMR ($CDCl_3$) of the diastereomer of **2k-1**.

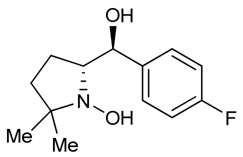

diastereomer of **2k-1** (minor)

[Lux® Amylose-1 column, 30 ºC, hexane/EtOH = 85/15 (v/v), 1.0 mL/min, 270 nm]

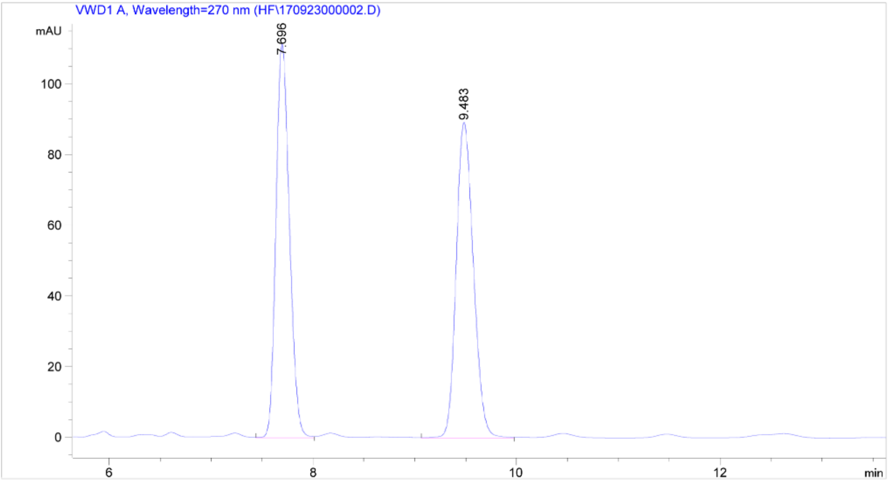


| Pk # | Retention Time [min] | Area [mAU*s] | Height [mAU] | Area Percent % |
|---|---|---|---|---|
| 1 | 7.696 | 1025.07556 | 111.50843 | 49.5229 |
| 2 | 9.483 | 1044.82837 | 89.20393 | 50.4771 |
| Totals: | | 2069.90393 | 200.71236 | 100.0000 |

***Supplementary Figure 118.*** Racemate of the diastereomer of **2k-1**.

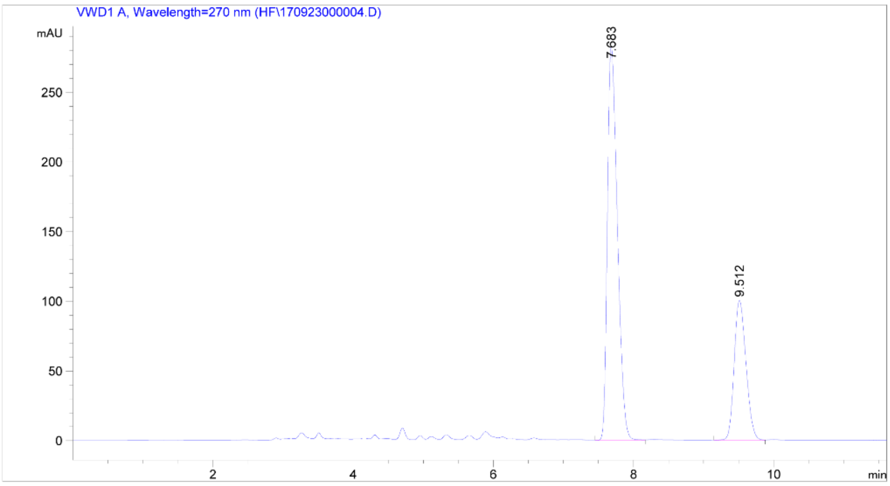


| Pk # | Retention Time [min] | Area [mAU*s] | Height [mAU] | Area Percent % |
|---|---|---|---|---|
| 1 | 7.683 | 2810.85400 | 283.10849 | 70.4526 |
| 2 | 9.512 | 1178.85583 | 100.39719 | 29.5474 |
| Totals: | | 3989.70984 | 383.50568 | 100.0000 |

***Supplementary Figure 119.*** Enantioenriched mixture of the diastereomer of **2k-1**.

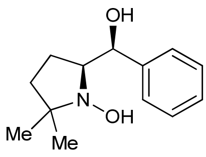


**2k-2** (major)

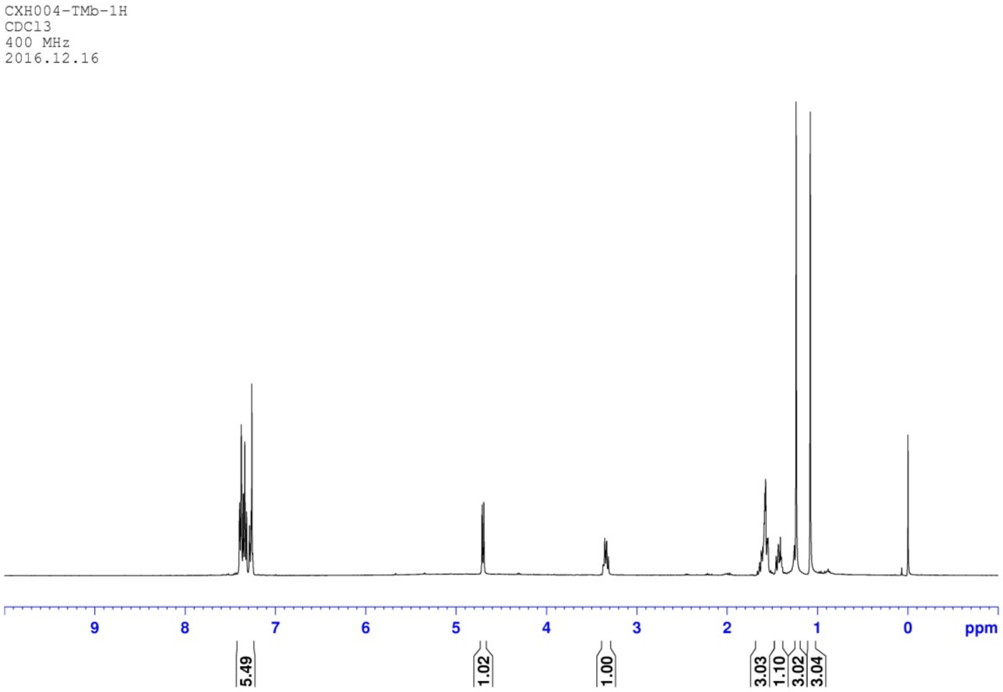


***Supplementary Figure 120.*** $^1$H NMR ($CDCl_3$) of compound **2k-2**.

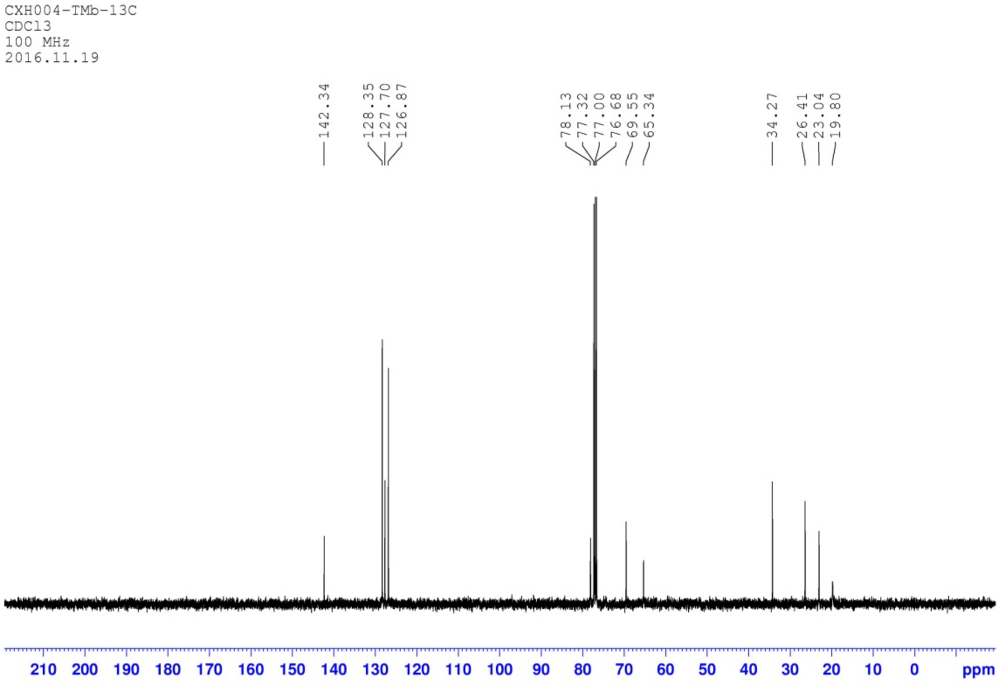


***Supplementary Figure 121.*** $^{13}$C NMR ($CDCl_3$) of compound **2k-2**.

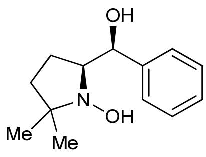

**2k-2** (major)

[Lux® Amylose-1 column, 30 ºC, hexane/EtOH = 80/20 (v/v), 1.0 mL/min, 220 nm]

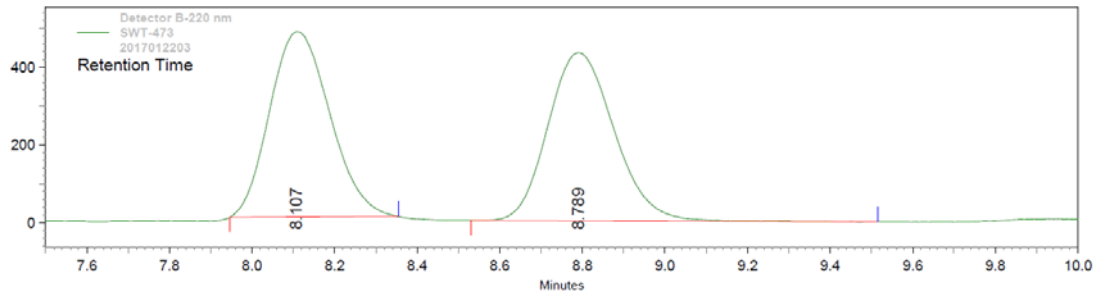

Detector B-220 nm

| Pk # | Retention Time | Area | Area Percent | Height |
|---|---|---|---|---|
| 1 | 8.107 | 4818013 | 50.00 | 477288 |
| 2 | 8.789 | 4817168 | 50.00 | 433144 |

***Supplementary Figure 122.*** Racemate of compound **2k-2**.

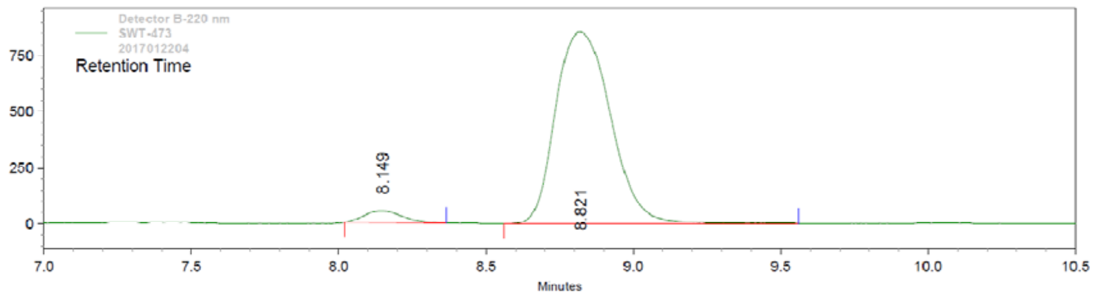

Detector B-220 nm

| Pk # | Retention Time | Area | Area Percent | Height |
|---|---|---|---|---|
| 1 | 8.149 | 448823 | 3.90 | 50046 |
| 2 | 8.821 | 11073889 | 96.10 | 854629 |

***Supplementary Figure 123.*** Enantioenriched mixture of compound **2k-2**.

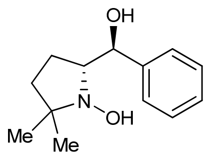


diastereomer of **2k-2** (minor)

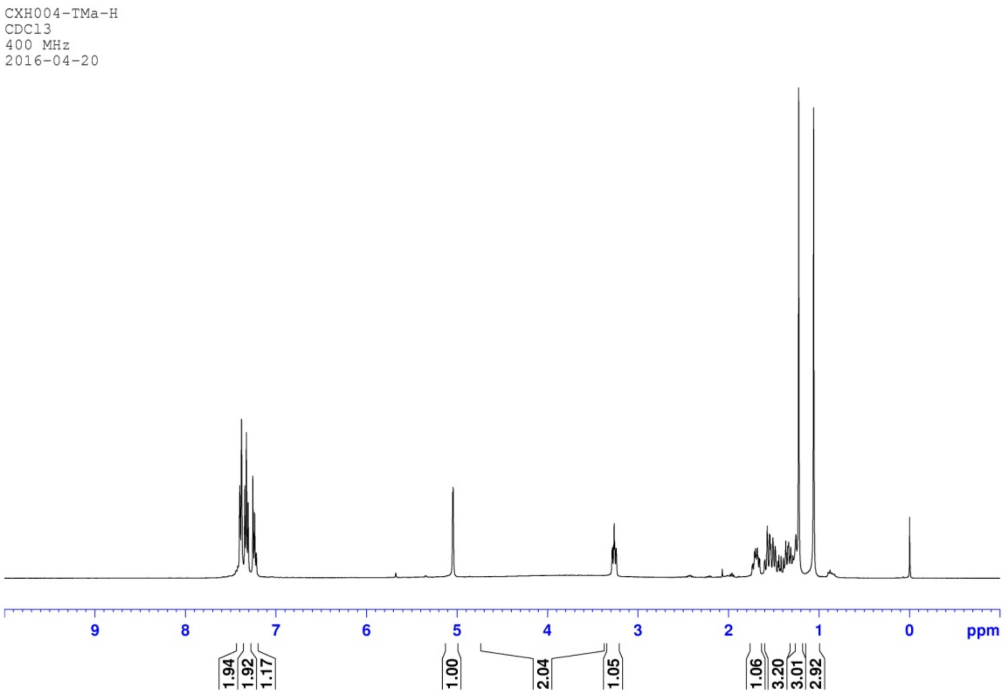


***Supplementary Figure 124.*** $^{1}$H NMR ($CDCl_3$) of the diastereomer of **2k-2**.

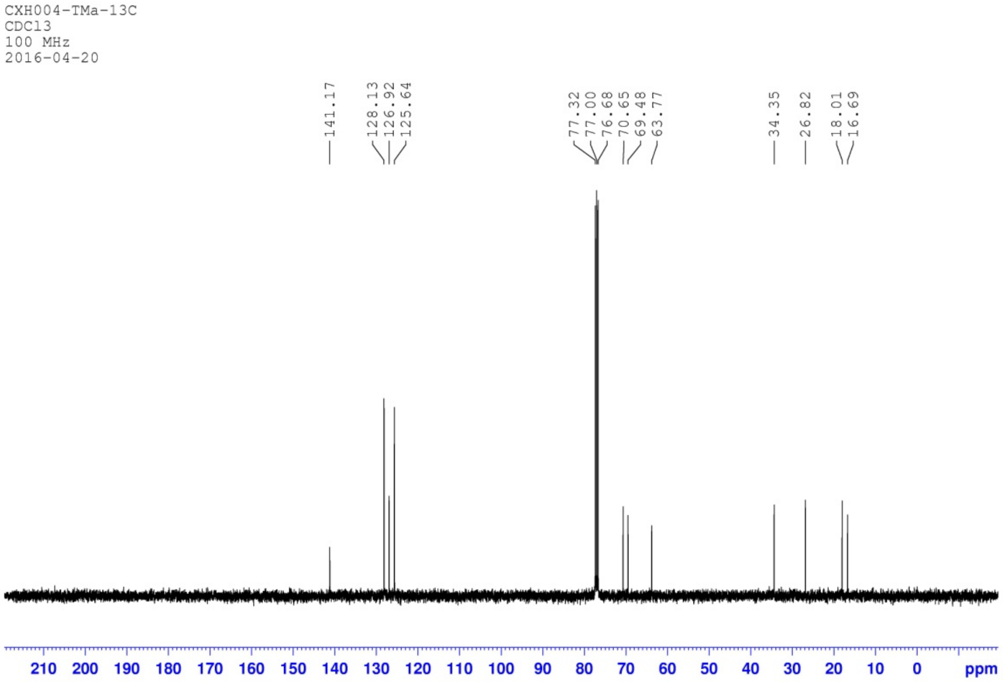


***Supplementary Figure 125.*** $^{13}$C NMR ($CDCl_3$) of the diastereomer of **2k-2**.

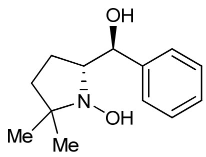


diastereomer of **2k-2** (minor)

[Lux® Amylose-1 column, 30 ºC, hexane/EtOH = 95/5 (v/v), 1.0 mL/min, 220 nm]

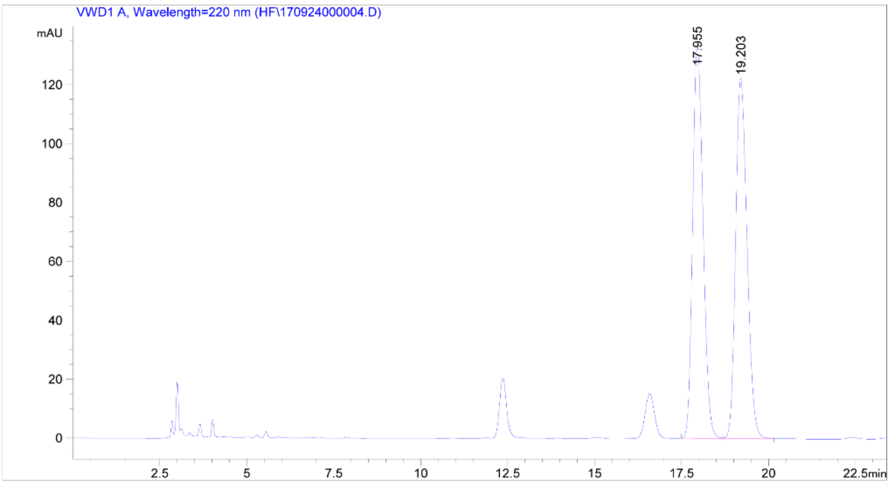


| Pk # | Retention Time [min] | Area [mAU*s] | Height [mAU] | Area Percent % |
|---|---|---|---|---|
| 1 | 17.955 | 2786.83252 | 133.47337 | 49.9584 |
| 2 | 19.203 | 2791.47876 | 122.29587 | 50.0416 |
| Totals: | | 5578.31128 | 255.76924 | 100.0000 |

***Supplementary Figure 126.*** Racemate of the diastereomer of **2k-2**.

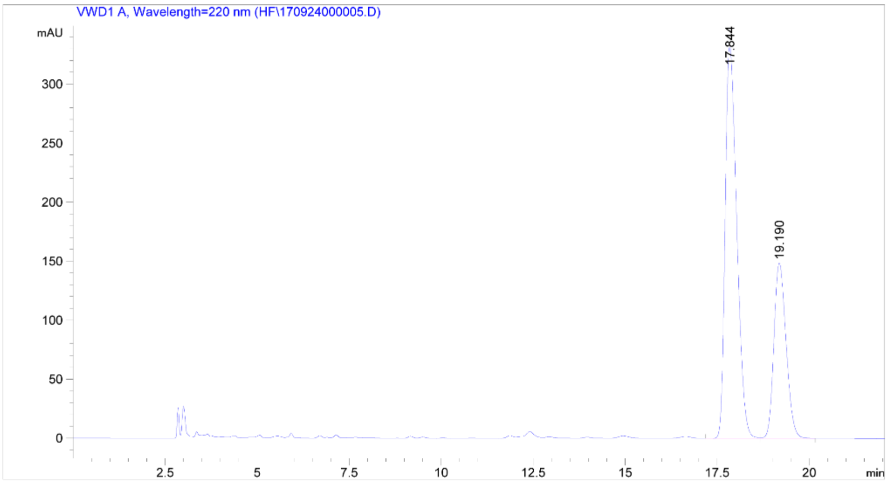


| Pk # | Retention Time [min] | Area [mAU*s] | Height [mAU] | Area Percent % |
|---|---|---|---|---|
| 1 | 17.844 | 7275.76367 | 332.85065 | 68.0117 |
| 2 | 19.190 | 3422.04175 | 148.47241 | 31.9883 |
| Totals: | | 1.06978e4 | 481.32306 | 100.0000 |

***Supplementary Figure 127.*** Enantioenriched mixture of the diastereomer of **2k-2**.

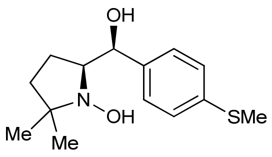


**2k-3** (major)

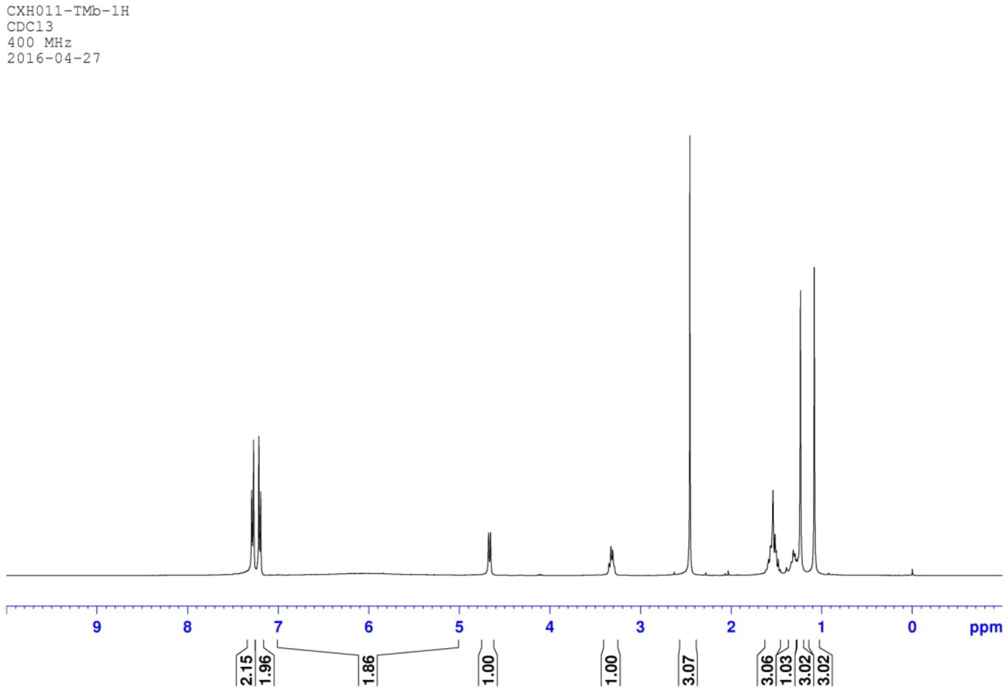


***Supplementary Figure 128.*** $^{1}$H NMR ($CDCl_3$) of compound **2k-3**.

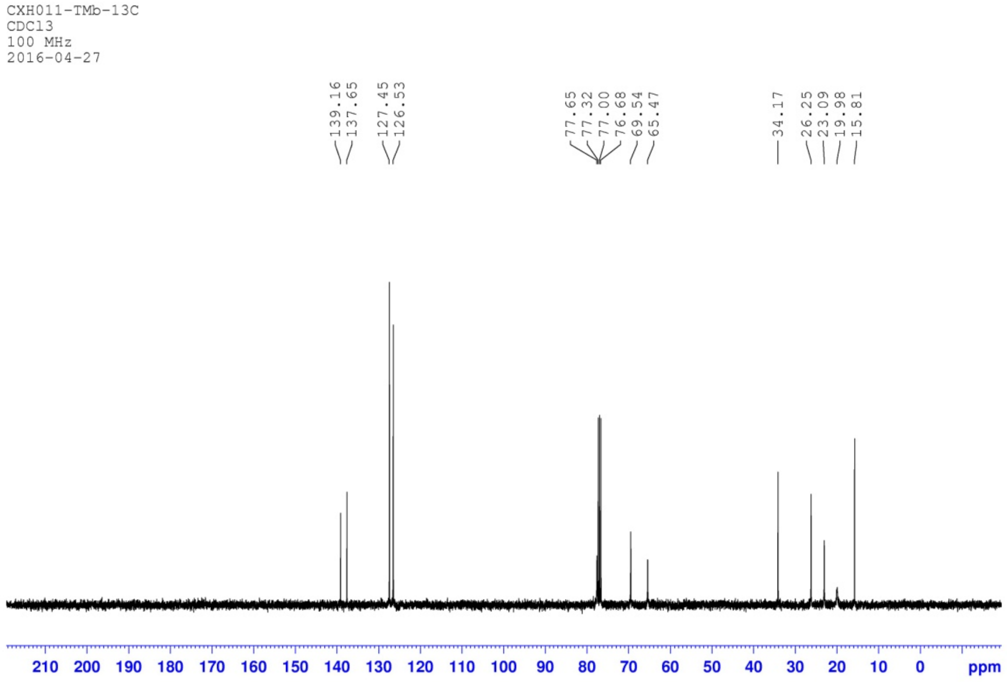


***Supplementary Figure 129.*** $^{13}$C NMR ($CDCl_3$) of compound **2k-3**.

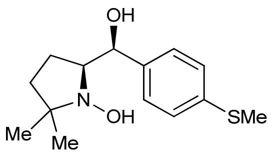


**2k-3** (major)

[Lux® Amylose-1 column, 30 ºC, hexane/EtOH = 80/20 (v/v), 1.0 mL/min, 270 nm]

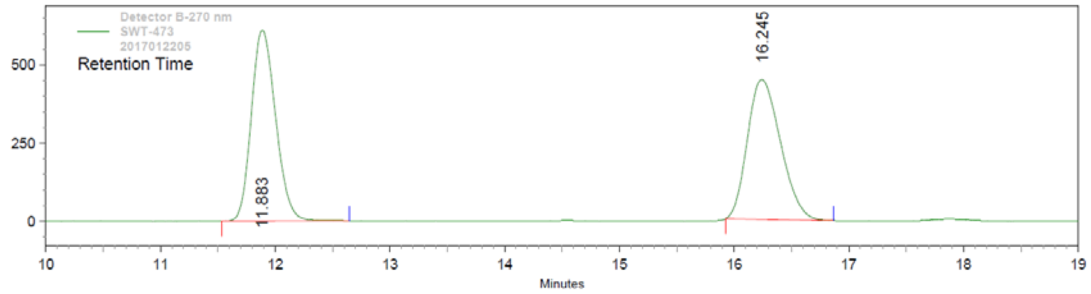


Detector B-270 nm

| Pk # | Retention Time | Area | Area Percent | Height |
|---|---|---|---|---|
| 1 | 11.883 | 8989956 | 49.99 | 610083 |
| 2 | 16.245 | 8994740 | 50.01 | 447376 |

***Supplementary Figure 130.*** Racemate of compound **2k-3**.

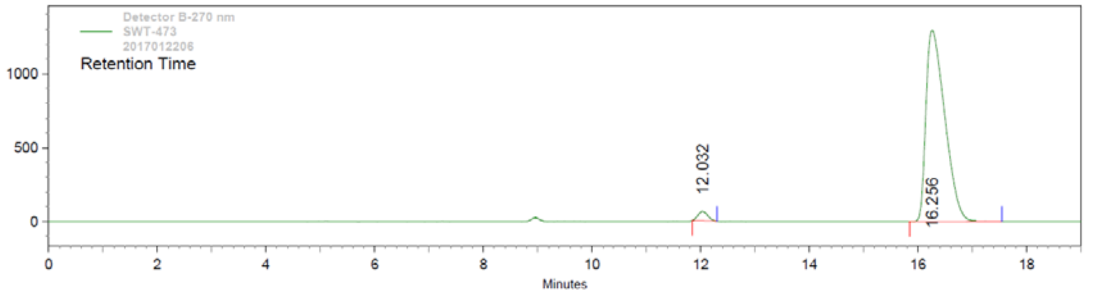


Detector B-270 nm

| Pk # | Retention Time | Area | Area Percent | Height |
|---|---|---|---|---|
| 1 | 12.032 | 802192 | 2.51 | 61774 |
| 2 | 16.256 | 31219066 | 97.49 | 1294447 |

***Supplementary Figure 131.*** Enantioenriched mixture of compound **2k-3**.

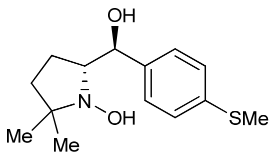

diastereomer of **2k-3** (minor)

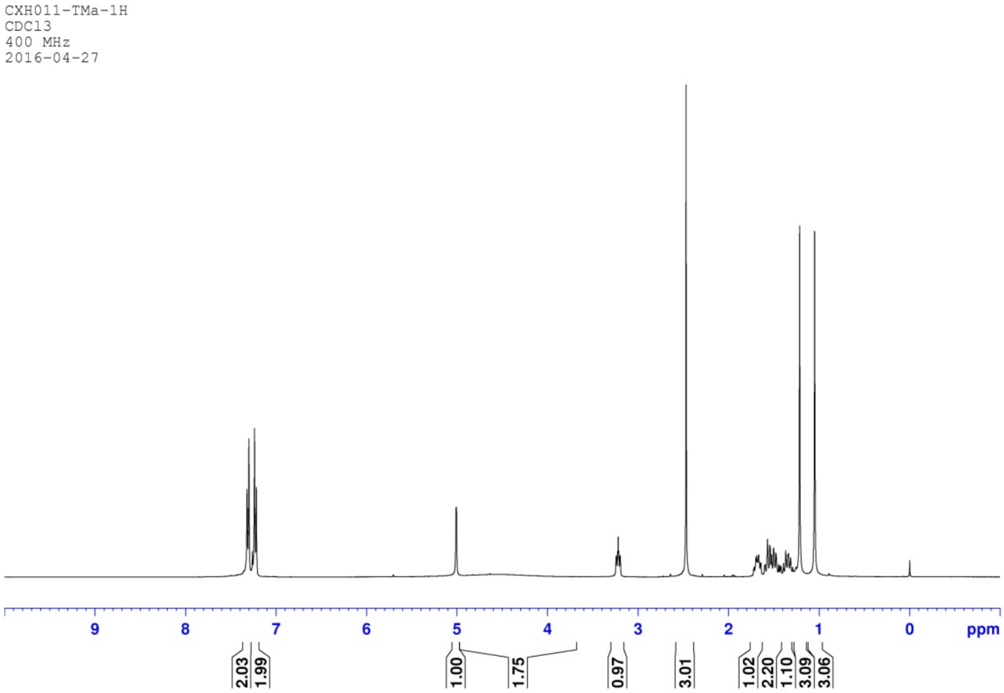


***Supplementary Figure 132.*** $^{1}$H NMR ($CDCl_3$) of the diastereomer of **2k-3**.

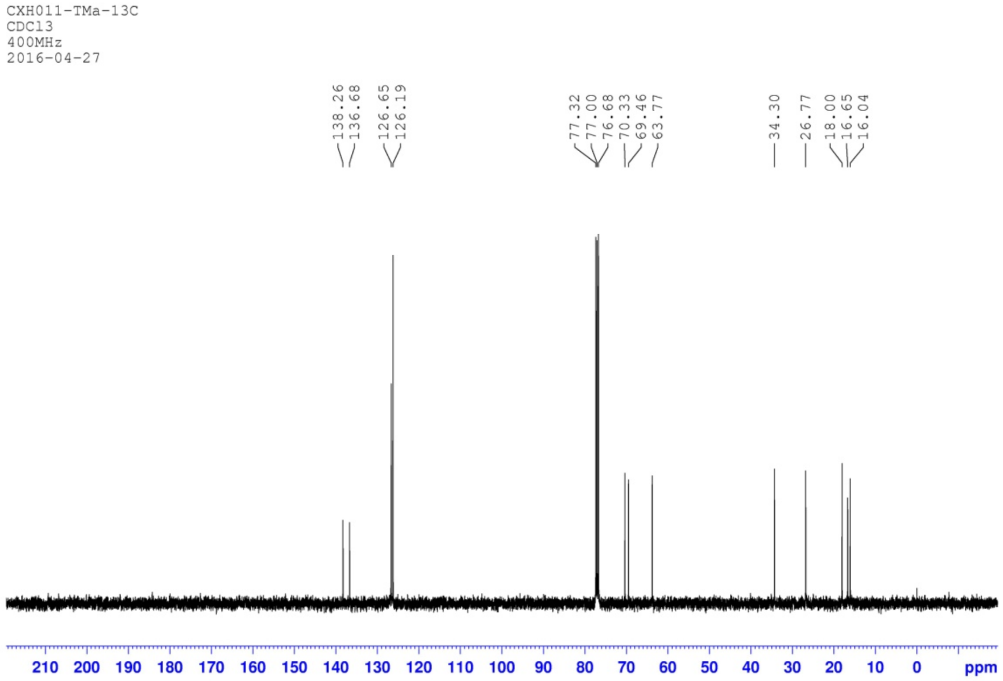


***Supplementary Figure 133.*** $^{13}$C NMR ($CDCl_3$) of the diastereomer of **2k-3**.

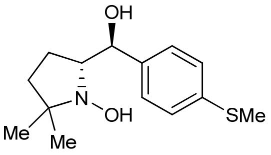

diastereomer of **2k-3** (minor)

[Lux® Amylose-1 column, 30 ºC, hexane/EtOH = 85/15 (v/v), 1.0 mL/min, 270 nm]

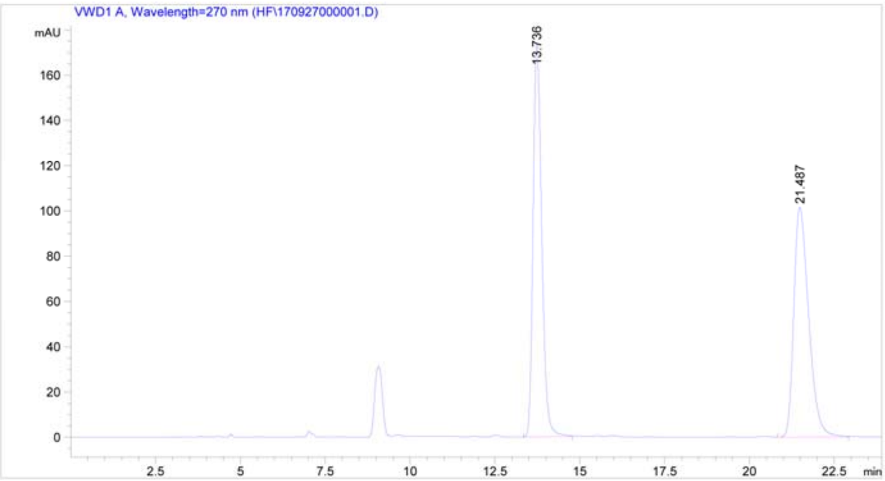


| Pk # | Retention Time [min] | Area [mAU*s] | Height [mAU] | Area Percent % |
|---|---|---|---|---|
| 1 | 13.736 | 3080.38794 | 172.95279 | 50.0224 |
| 2 | 21.487 | 3077.63428 | 101.38963 | 49.9776 |
| Totals: | | 6158.02222 | 274.34241 | 100.0000 |

***Supplementary Figure 134.*** Racemate of the diastereomer of **2k-3**.

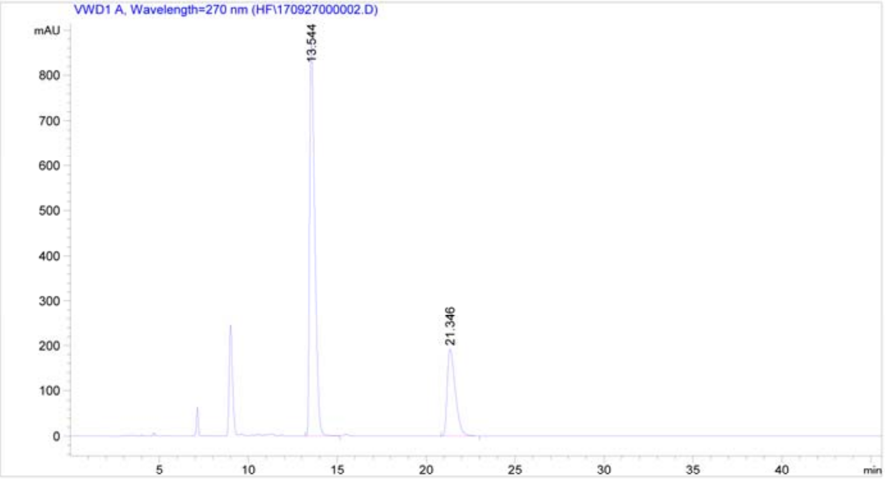


| Pk # | Retention Time [min] | Area [mAU*s] | Height [mAU] | Area Percent % |
|---|---|---|---|---|
| 1 | 13.544 | 1.76278e4 | 871.05206 | 74.4174 |
| 2 | 21.346 | 6059.94482 | 191.72087 | 25.5826 |
| Totals: | | 2.36877e4 | 1062.77293 | 100.0000 |

***Supplementary Figure 135.*** Enantioenriched mixture of the diastereomer of **2k-3**.

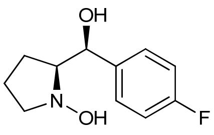


**2l** (major)

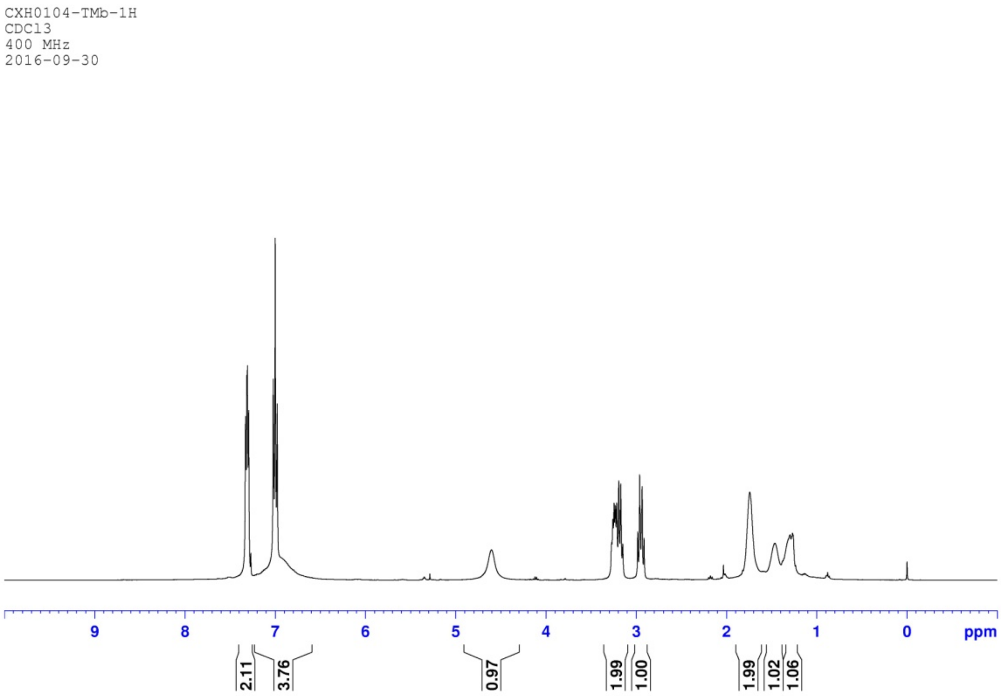


***Supplementary Figure 136.*** $^{1}$H NMR ($CDCl_3$) of compound **2l**.

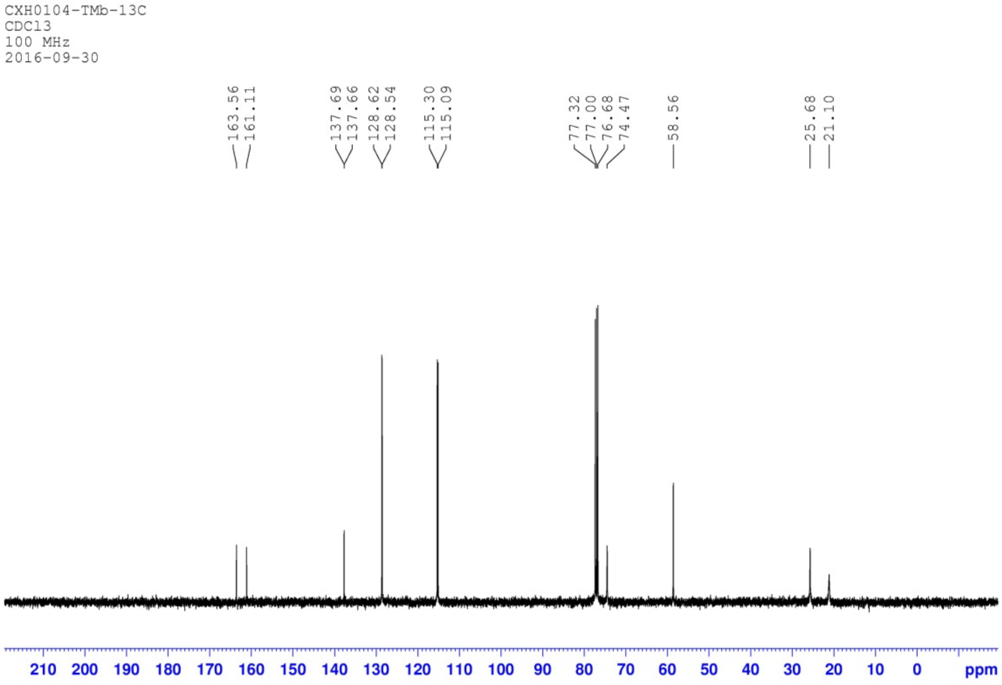


***Supplementary Figure 137.*** $^{13}$C NMR ($CDCl_3$) of compound **2l**.

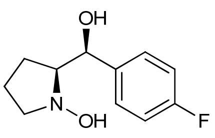


**2l** (major)

[Lux® Amylose-1 column, 30 ºC, hexane/EtOH = 70/30 (v/v), 1.0 mL/min, 270 nm]

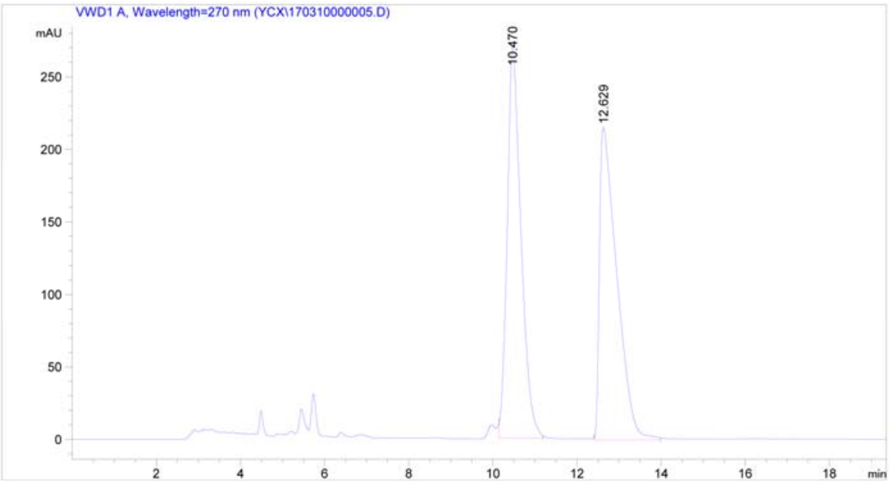


| Pk # | Retention Time [min] | Area [mAU*s] | Height [mAU] | Area Percent % |
|---|---|---|---|---|
| 1 | 10.470 | 6165.55664 | 270.67529 | 50.0106 |
| 2 | 12.629 | 6162.93604 | 215.40651 | 49.9894 |
| Totals: | | 1.23285e4 | 486.08180 | 100.0000 |

***Supplementary Figure 138.*** Racemate of compound **2l**.

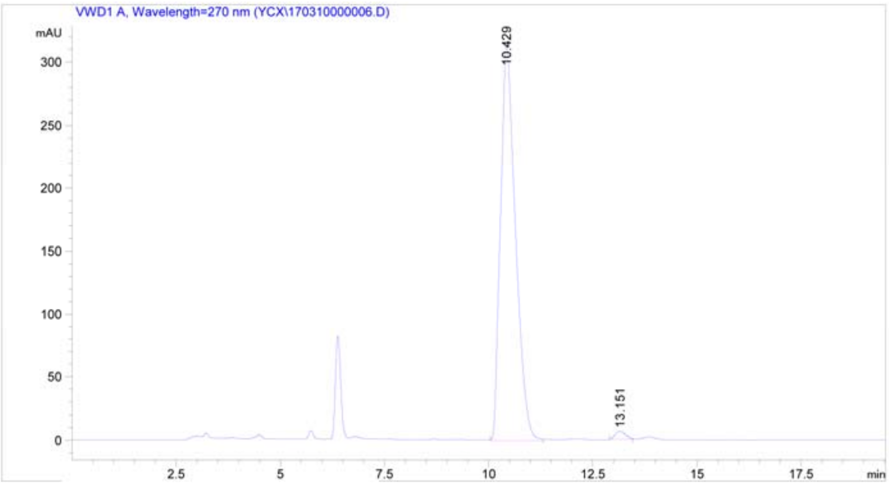


| Pk # | Retention Time [min] | Area [mAU*s] | Height [mAU] | Area Percent % |
|---|---|---|---|---|
| 1 | 10.429 | 7830.97705 | 313.14923 | 98.5951 |
| 2 | 13.151 | 111.58207 | 6.43195 | 1.4049 |
| Totals: | | 7942.55912 | 319.58118 | 100.0000 |

***Supplementary Figure 139.*** Enantioenriched mixture of compound **2l**.

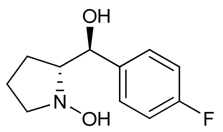


diastereomer of **2l** (minor)

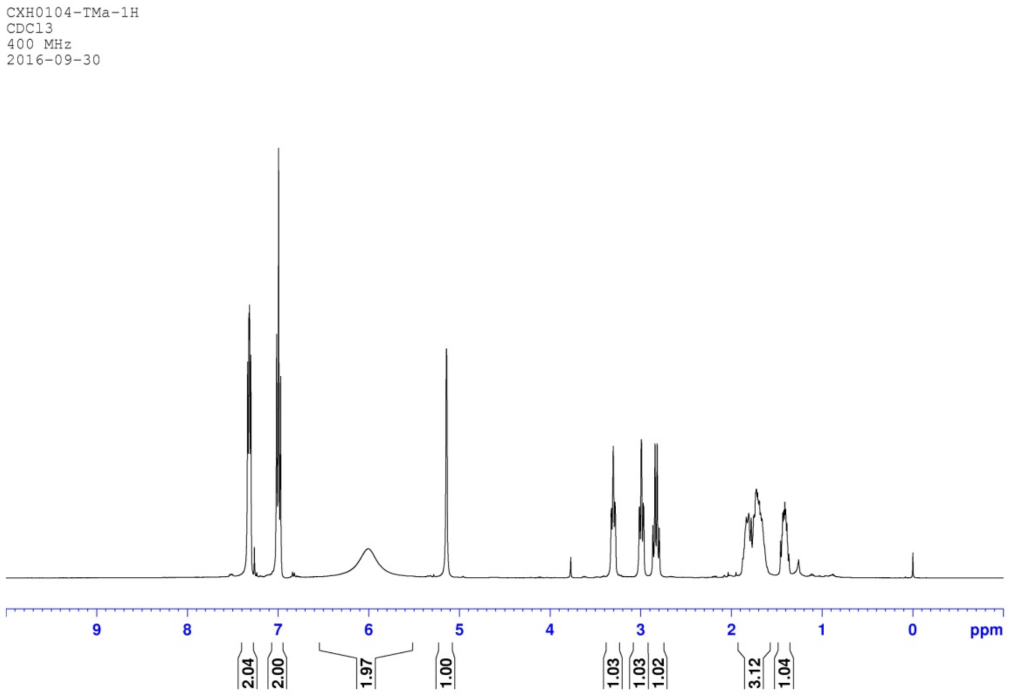


***Supplementary Figure 140.*** $^{1}$H NMR ($CDCl_3$) of the diastereomer of **2l**.

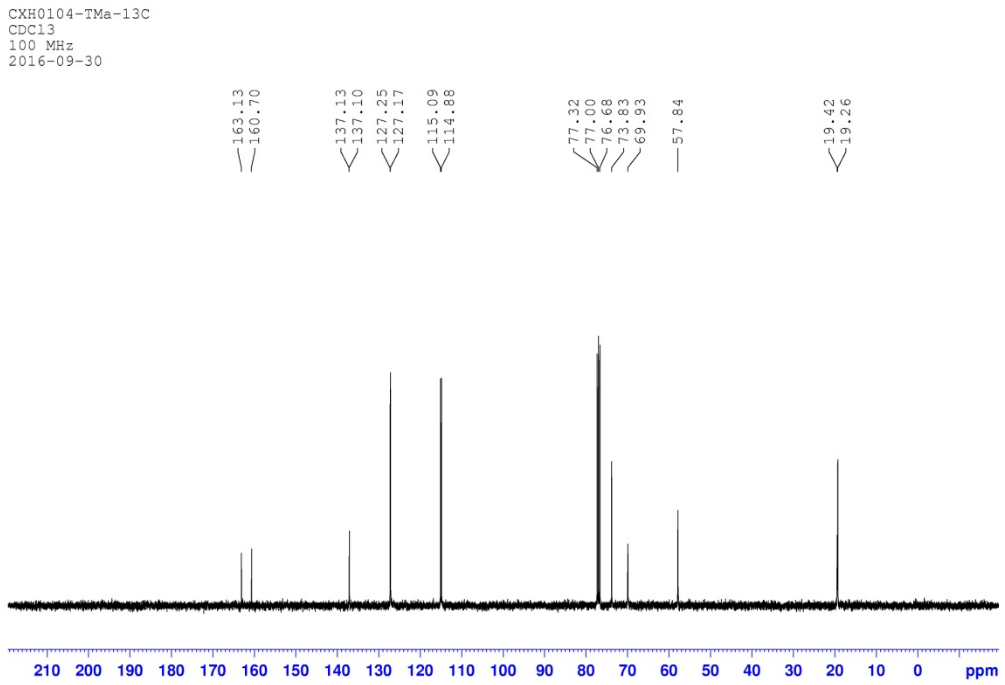


***Supplementary Figure 141.*** $^{13}$C NMR ($CDCl_3$) of the diastereomer of **2l**.

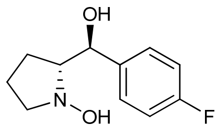


diastereomer of **2l** (minor)

[Lux® Amylose-1 column, 30 ºC, hexane/EtOH = 85/15 (v/v), 1.0 mL/min, 270 nm]

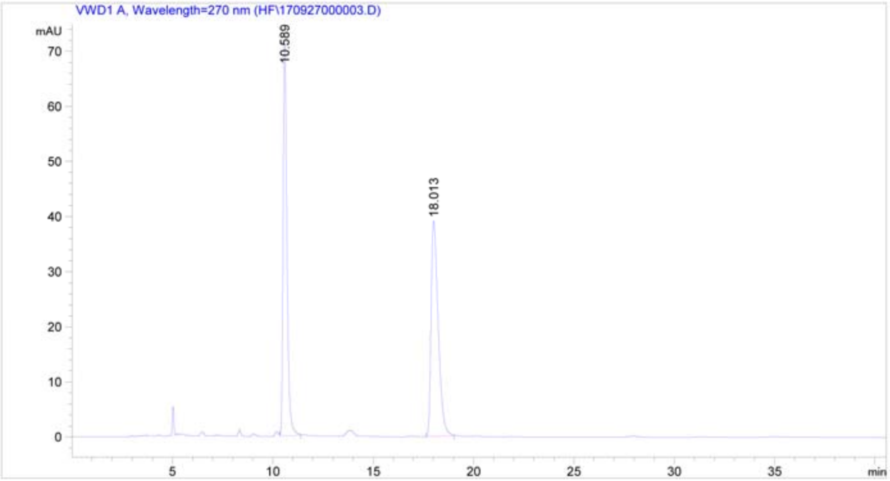


| Pk # | Retention Time [min] | Area [mAU*s] | Height [mAU] | Area Percent % |
|---|---|---|---|---|
| 1 | 10.589 | 955.67194 | 71.14536 | 49.9710 |
| 2 | 18.013 | 956.78064 | 39.13541 | 50.0290 |
| Totals: | | 1912.45258 | 110.28077 | 100.0000 |

***Supplementary Figure 142.*** Racemate of the diastereomer of **2l**.

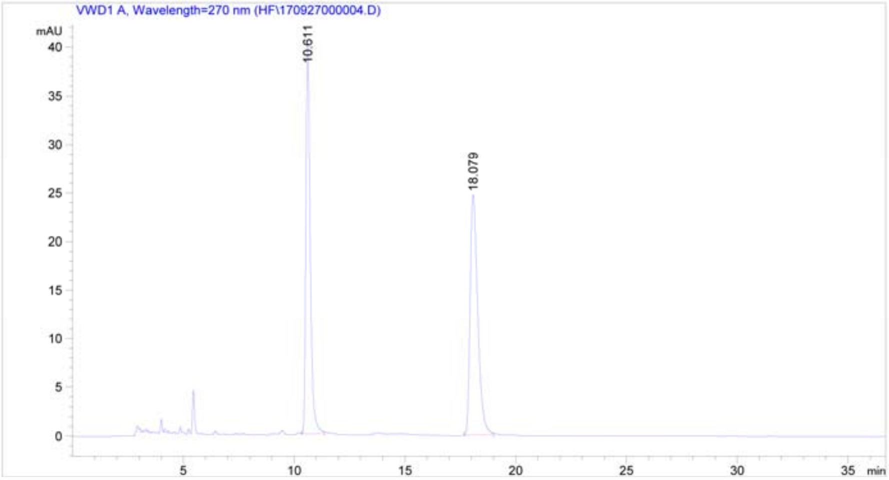


| Pk # | Retention Time [min] | Area [mAU*s] | Height [mAU] | Area Percent % |
|---|---|---|---|---|
| 1 | 10.611 | 547.21069 | 40.09608 | 48.2250 |
| 2 | 18.079 | 587.49280 | 24.67539 | 51.7750 |
| Totals: | | 1134.70349 | 64.77147 | 100.0000 |

***Supplementary Figure 143.*** Enantioenriched mixture of the diastereomer of **2l**.

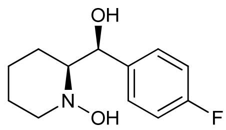


**2m** (DMSO-d6, 25 ºC)

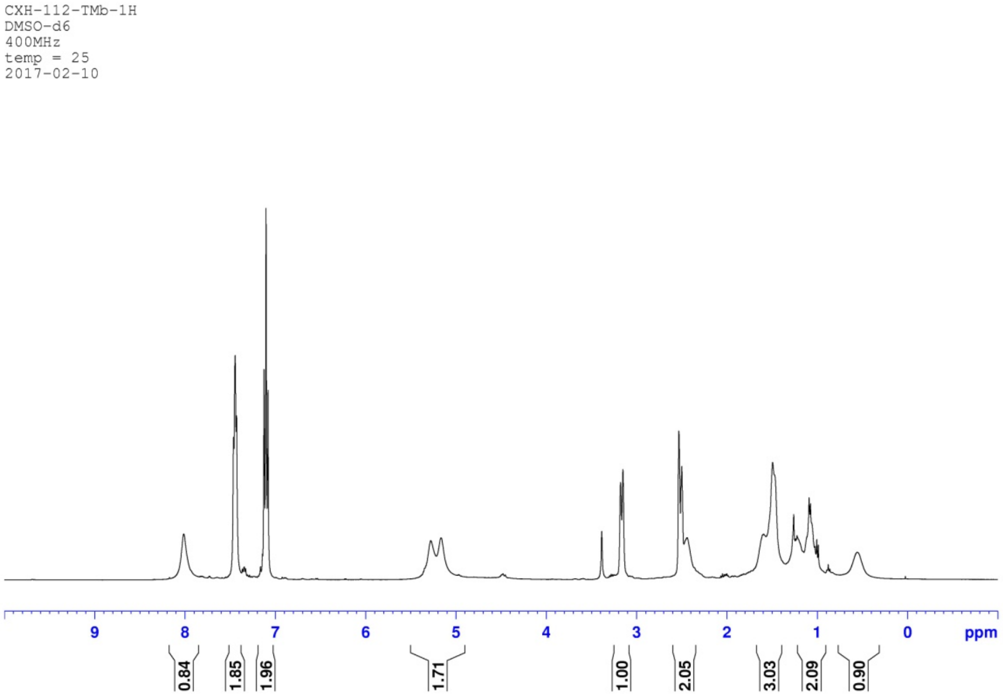


***Supplementary Figure 144.*** $^{1}$H NMR (DMSO-d6, 25 ºC) of compound **2m**.

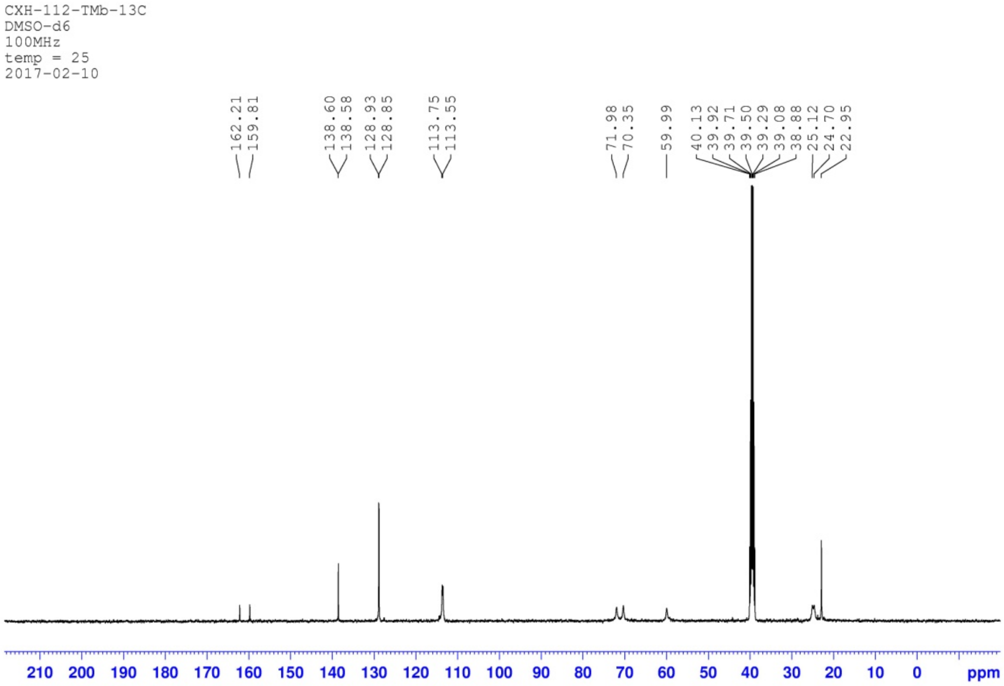


***Supplementary Figure 145.*** $^{13}$C NMR (DMSO-d6, 25 ºC) of compound **2m**.

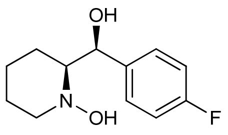


**2m** (DMSO-d6, 75 ºC)

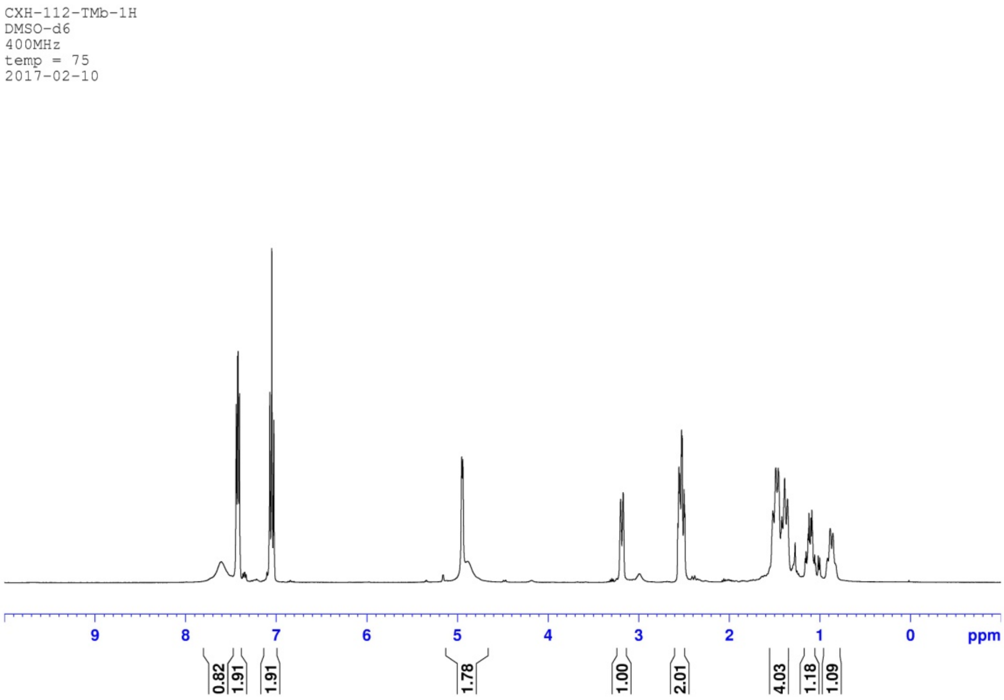


***Supplementary Figure 146.*** $^{1}$H NMR (DMSO-d6, 75 ºC) of compound **2m**.

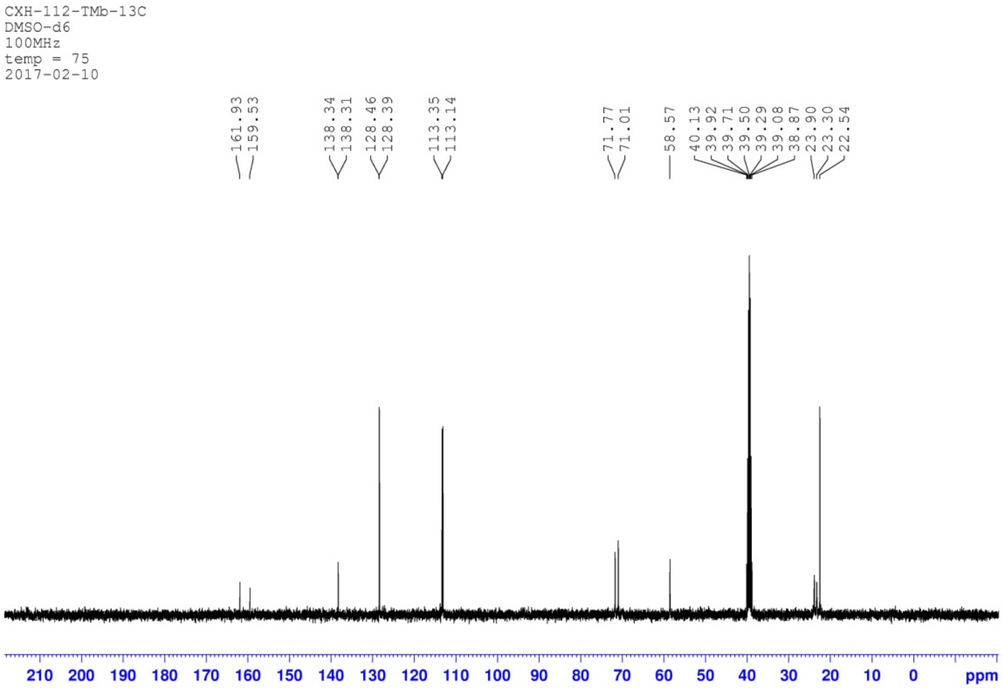


***Supplementary Figure 147.*** $^{13}$C NMR (DMSO-d6, 75 ºC) of compound **2m**.

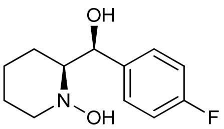


**2m**

[Lux® Amylose-1 column, 30 ºC, hexane/EtOH = 80/20 (v/v), 1.0 mL/min, 270 nm]

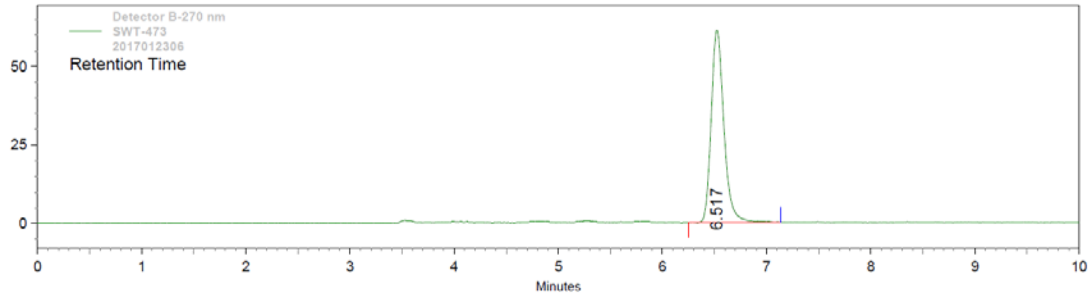


***Supplementary Figure 148.*** Racemate of the diastereomer of **2m**.

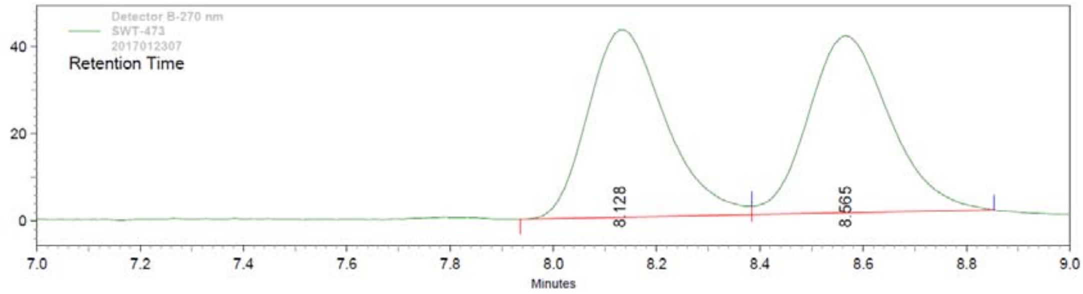


Detector B-270 nm

| Pk # | Retention Time | Area | Area Percent | Height |
|---|---|---|---|---|
| 1 | 8.128 | 446470 | 50.04 | 43060 |
| 2 | 8.565 | 445785 | 49.96 | 40691 |

***Supplementary Figure 149.*** Racemate of compound **2m**.

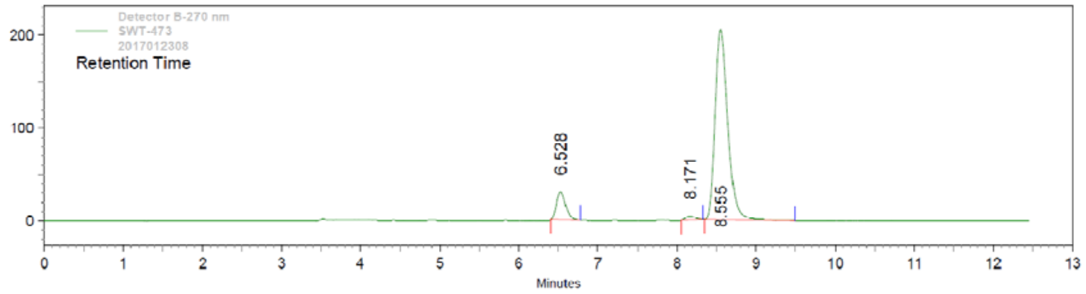


Detector B-270 nm

| Pk # | Retention Time | Area | Area Percent | Height |
|---|---|---|---|---|
| 1 | 6.528 | 234961 | 9.17 | 29771 |
| 2 | 8.171 | 30985 | 1.21 | 3631 |
| 3 | 8.555 | 2296744 | 89.62 | 204445 |

***Supplementary Figure 150.*** Enantioenriched diastereomeric mixture of **2m**.

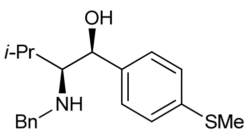


**6a-9**

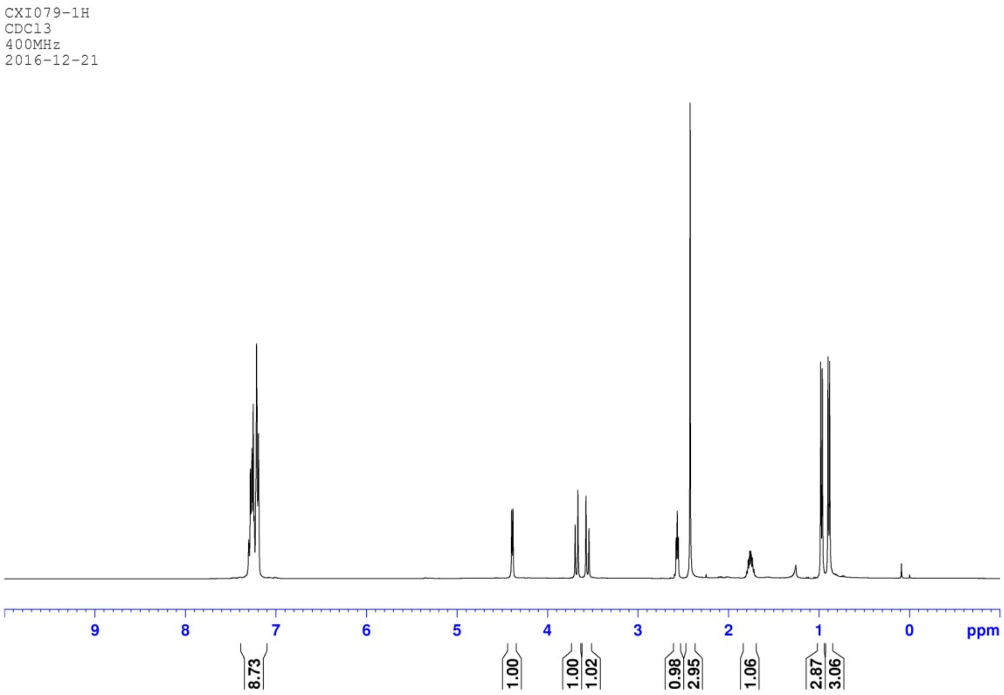


***Supplementary Figure 151.*** $^{1}$H NMR ($CDCl_3$) of compound **6a-9**.

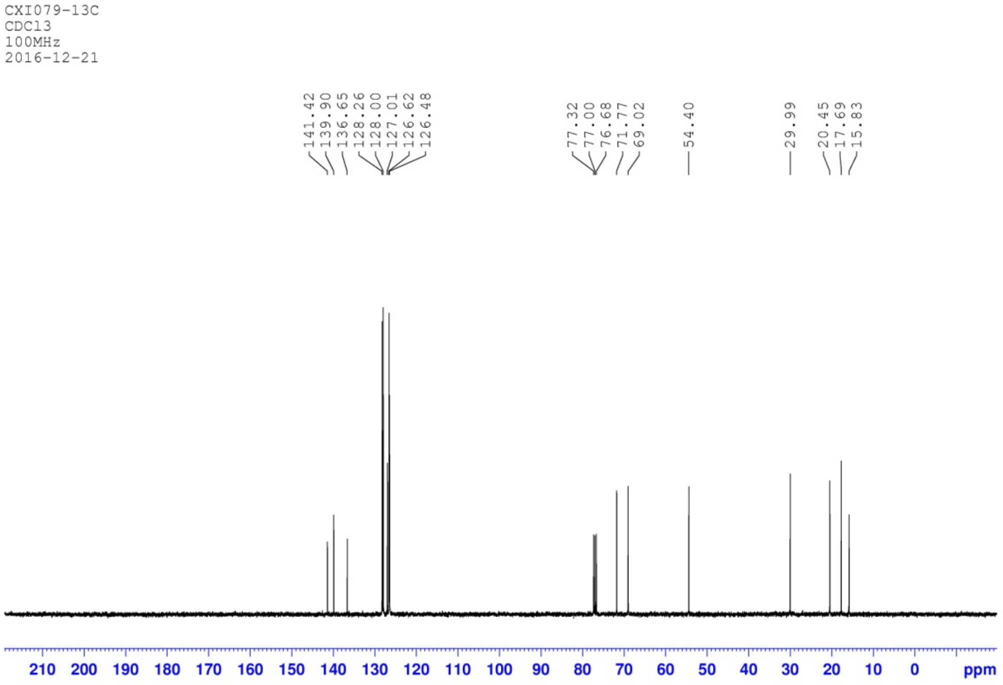


***Supplementary Figure 152.*** $^{13}$C NMR ($CDCl_3$) of compound **6a-9**.

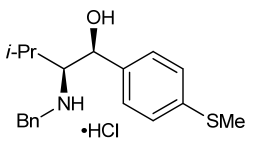


**6a-9•HCl**

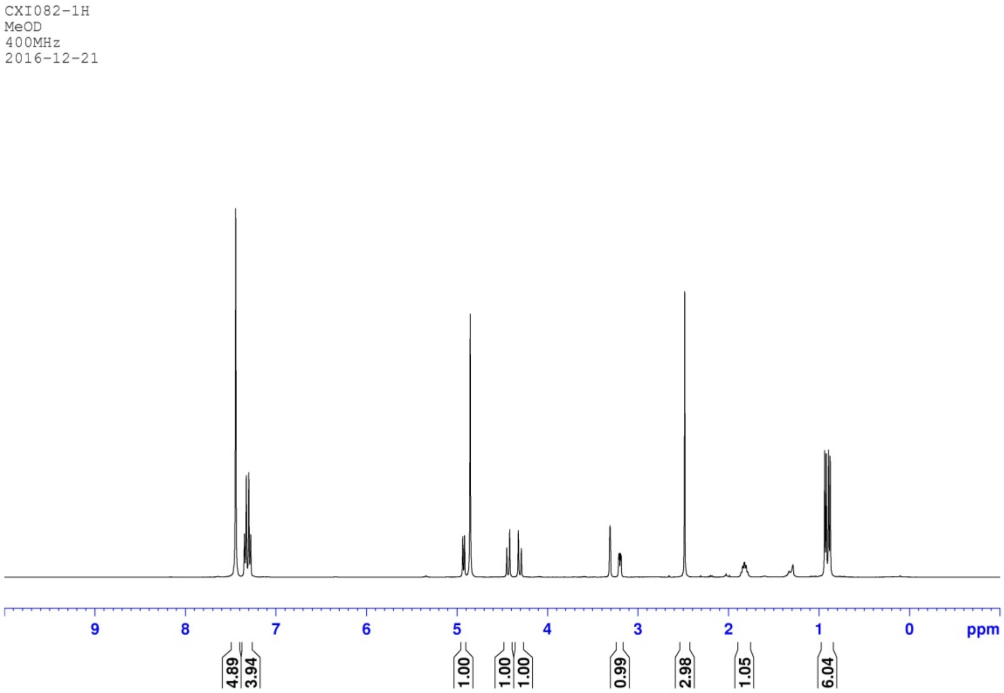


***Supplementary Figure 153.*** $^{1}$H NMR (MeOD) of compound **6a-9•HCl**.

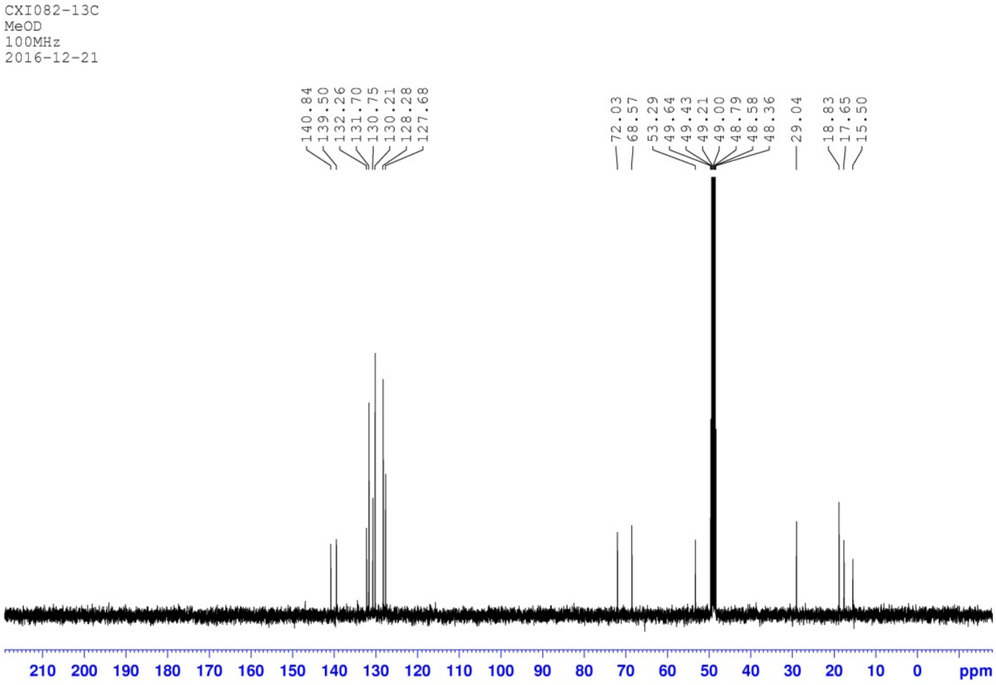


***Supplementary Figure 154.*** $^{13}$C NMR (MeOD) of compound **6a-9•HCl**.

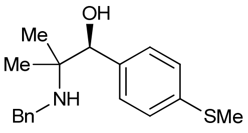


**6b-3**

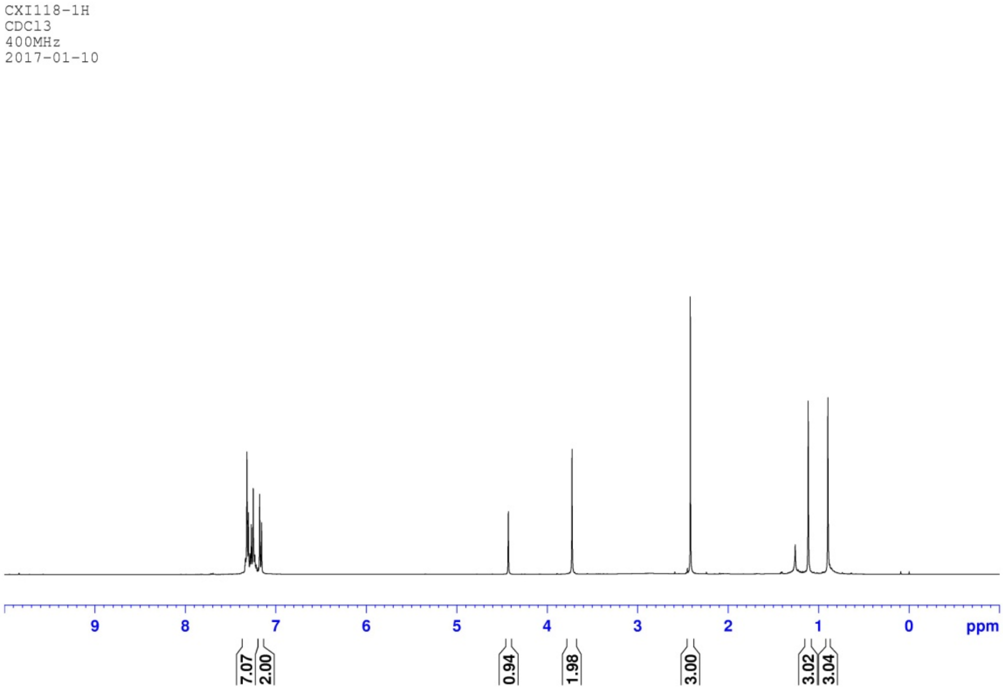


***Supplementary Figure 155.*** $^{1}$H NMR ($CDCl_3$) of compound **6b-3**.

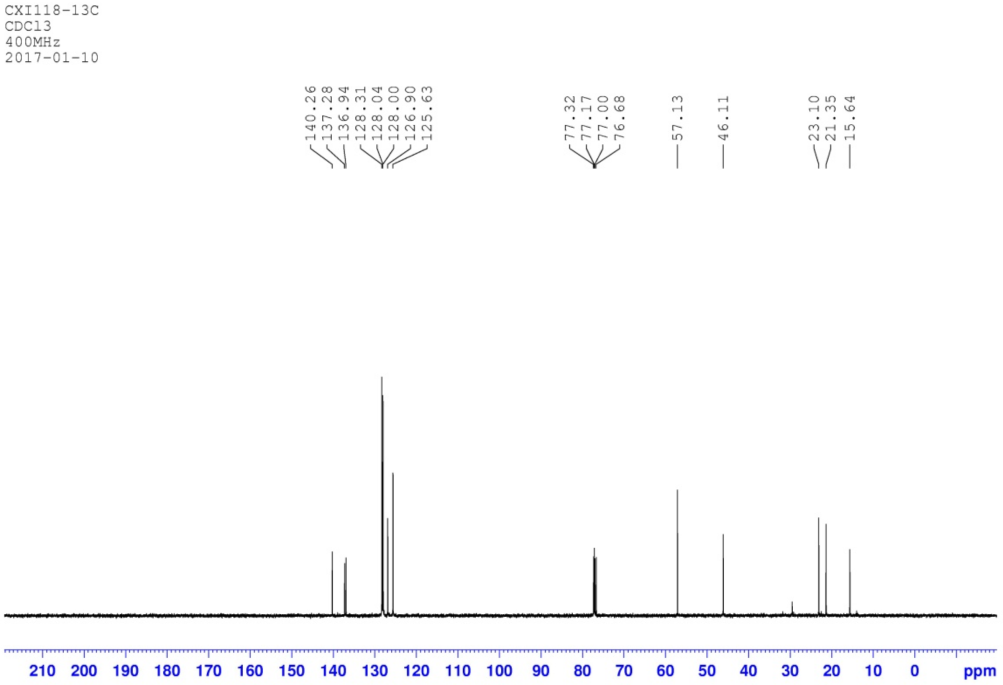


***Supplementary Figure 156.*** $^{13}$C NMR ($CDCl_3$) of compound **6b-3**.

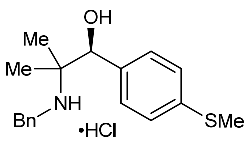


**6b-3•HCl**

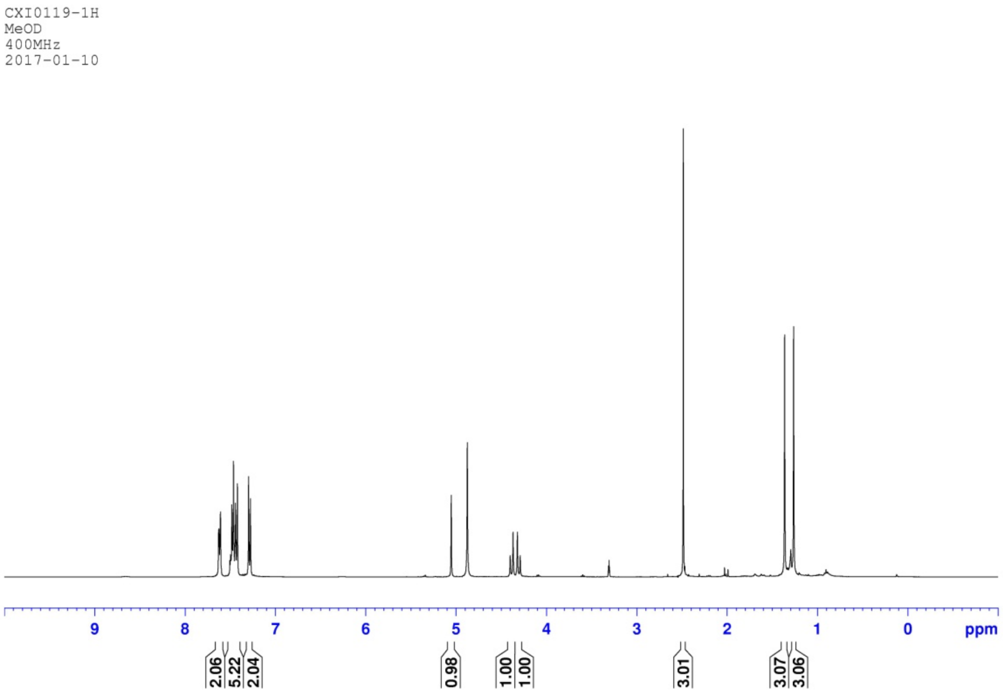


***Supplementary Figure 157.*** $^{1}$H NMR (MeOD) of compound **6b-3•HCl**.

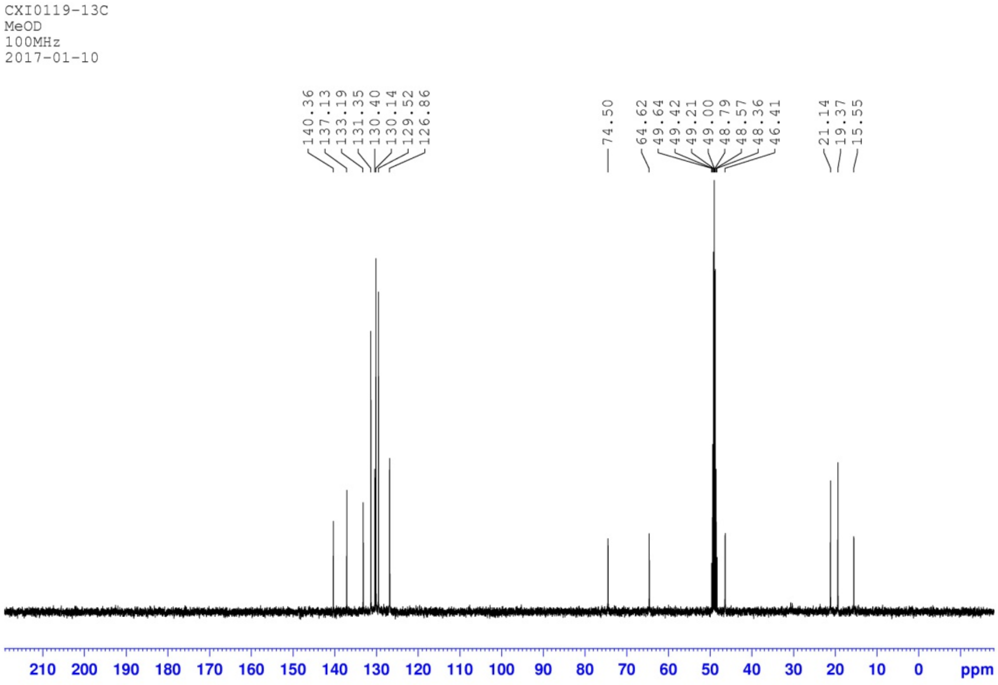


***Supplementary Figure 158.*** $^{13}$C NMR (MeOD) of compound **6b-3•HCl**.

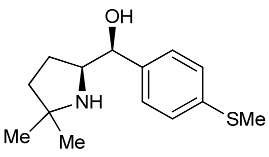


**6k-3**

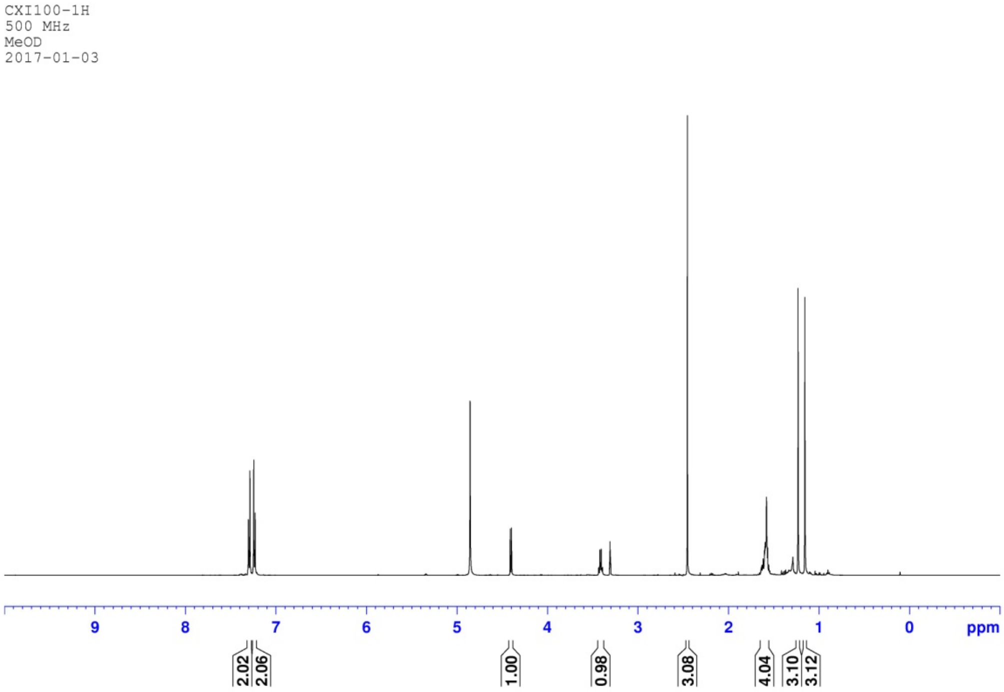


***Supplementary Figure 159.*** $^{1}$H NMR (MeOD) of compound **6k-3**.

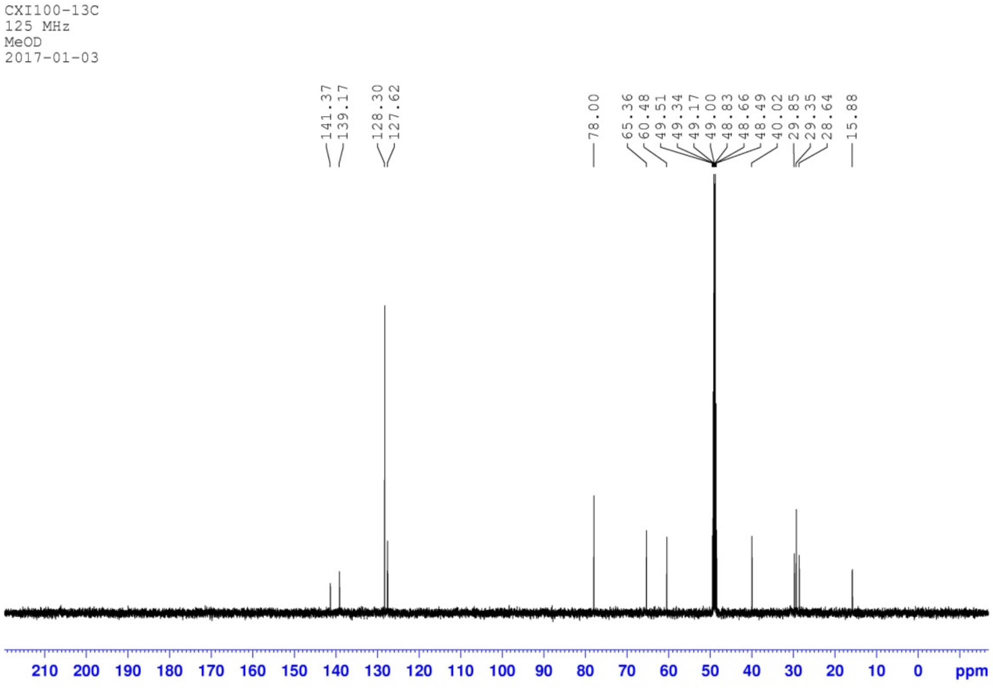


***Supplementary Figure 160.*** $^{13}$C NMR (MeOD) of compound **6k-3**.

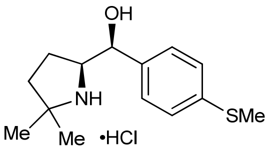


**6k-3•HCl**

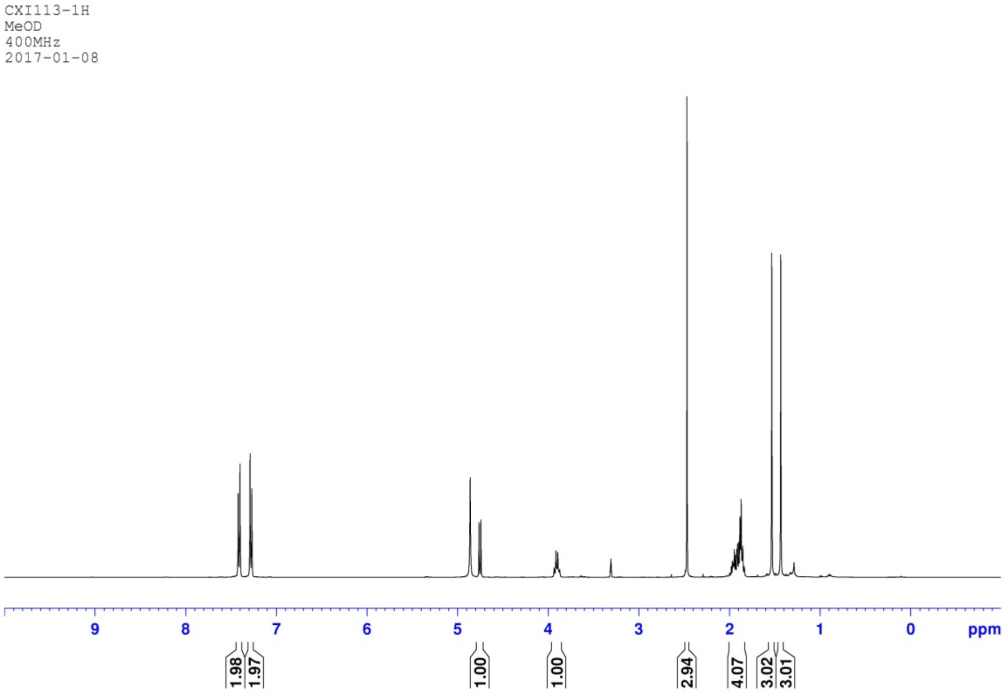


***Supplementary Figure 161.*** $^{1}$H NMR (MeOD) of compound **6k-3•HCl**.

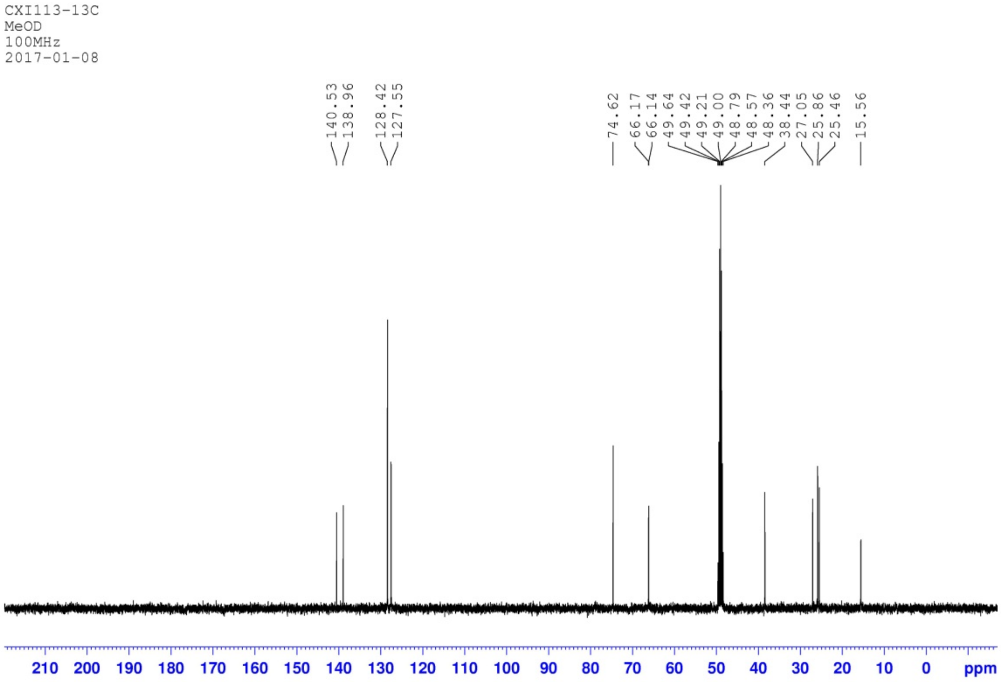


***Supplementary Figure 162.*** $^{13}$C NMR (MeOD) of compound **6k-3•HCl**.

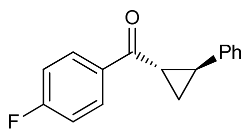

4

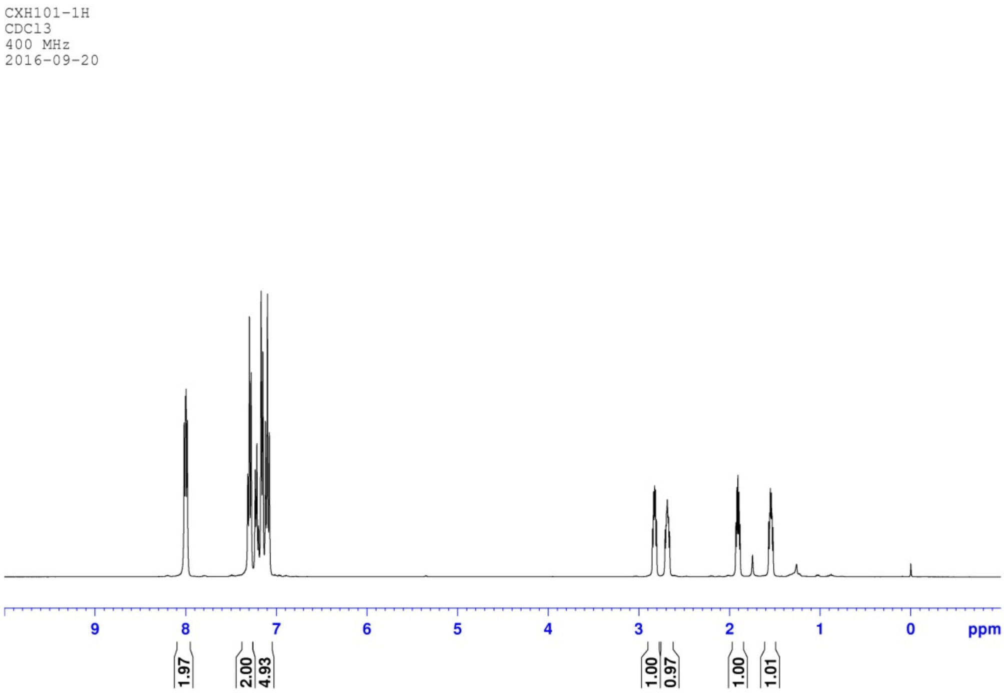


***Supplementary Figure 163.*** $^{1}$H NMR ($CDCl_3$) of compound **4**.

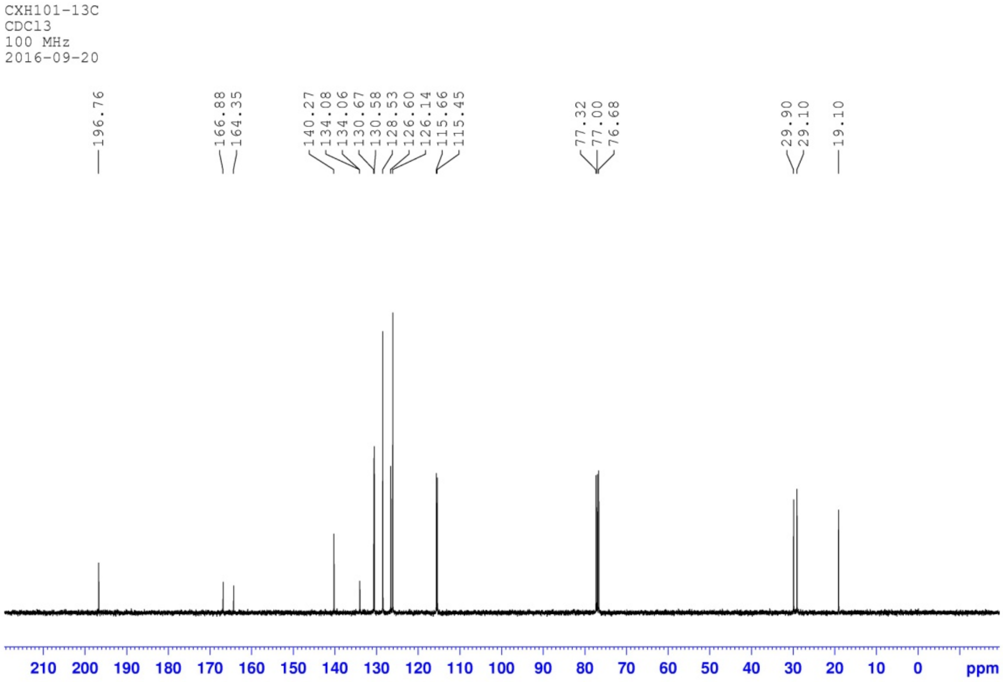


***Supplementary Figure 164.*** $^{13}$C NMR ($CDCl_3$) of compound **4**.

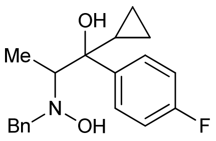


**2f-2** (mixture of diastereomers)

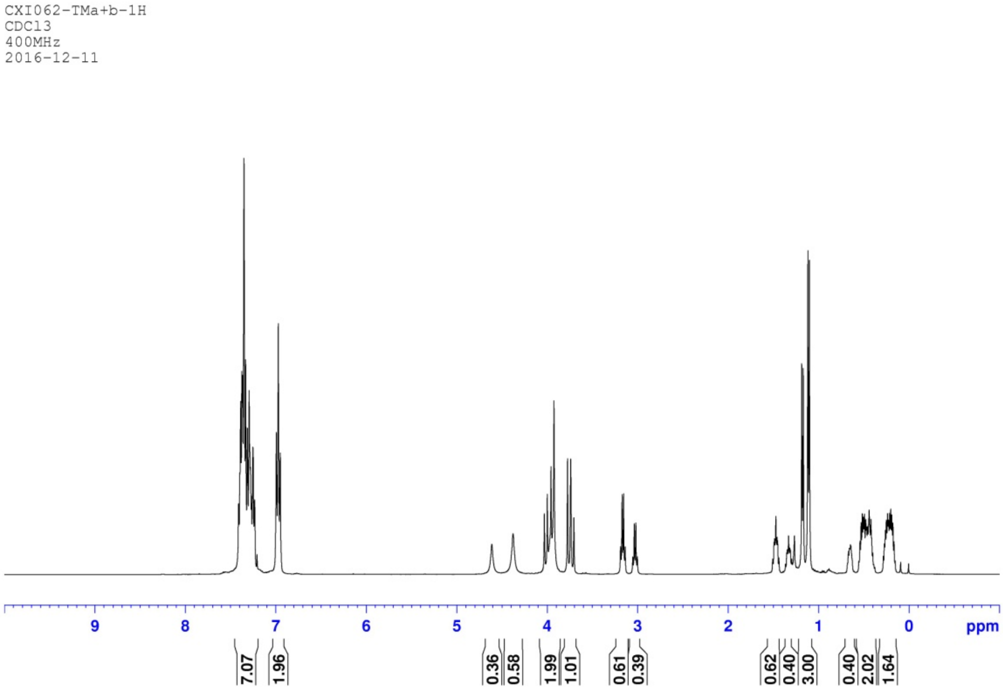


***Supplementary Figure 165.*** $^{1}$H NMR ($CDCl_3$) of the diastereomeric mixture of **2f-2**.

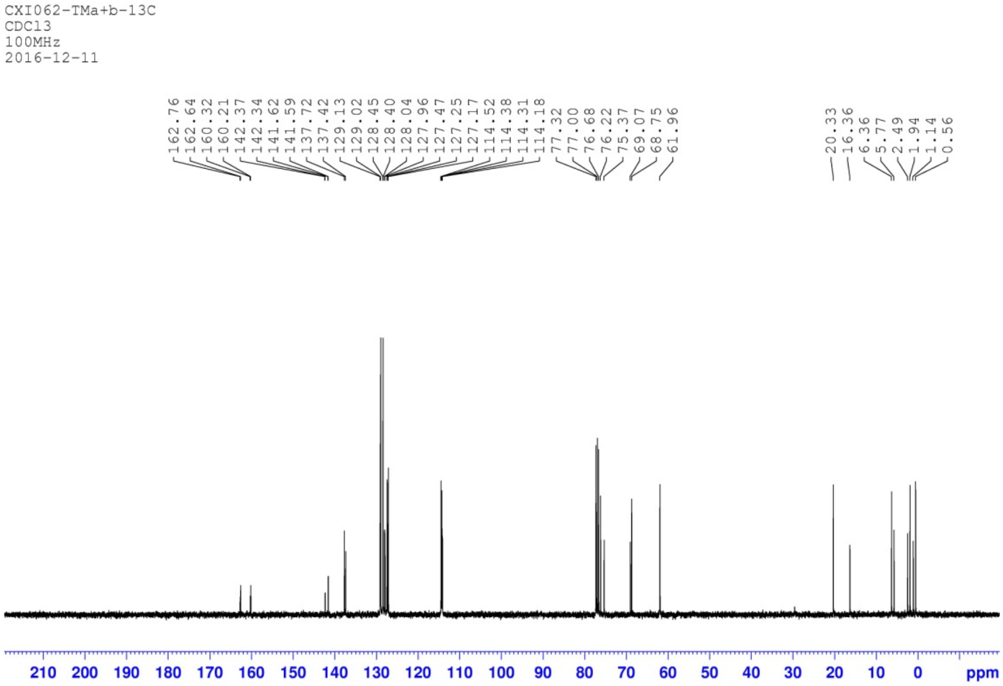


***Supplementary Figure 166.*** $^{13}$C NMR ($CDCl_3$) of the diastereomeric mixture of **2f-2**.

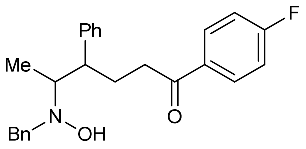


(major diastereomer of **5**)

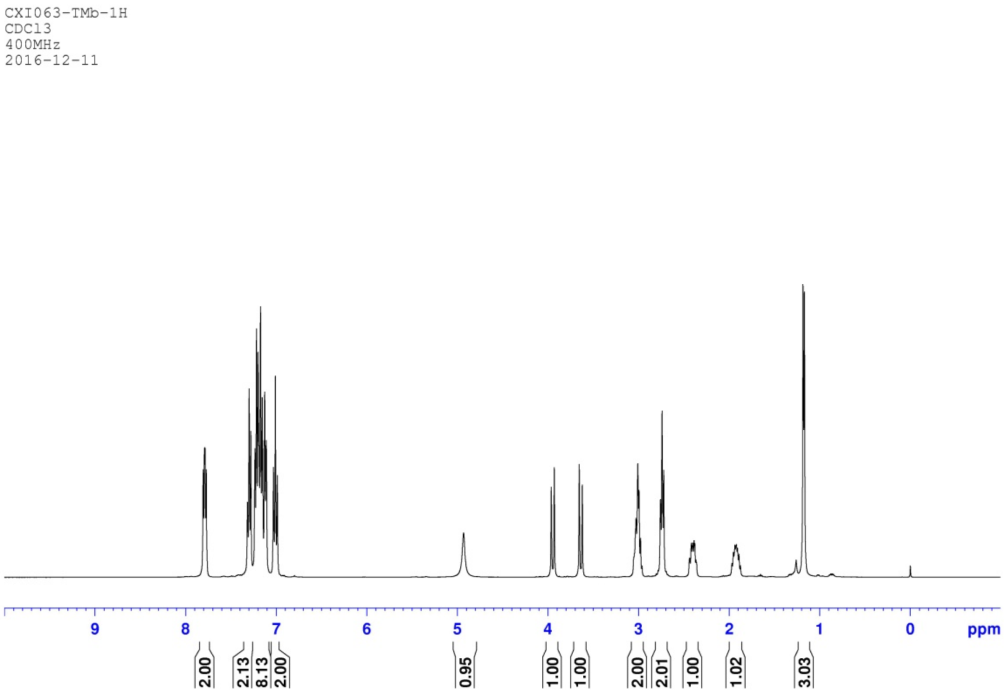


***Supplementary Figure 167.*** $^{1}$H NMR ($CDCl_3$) of the major diastereomer of **5**.

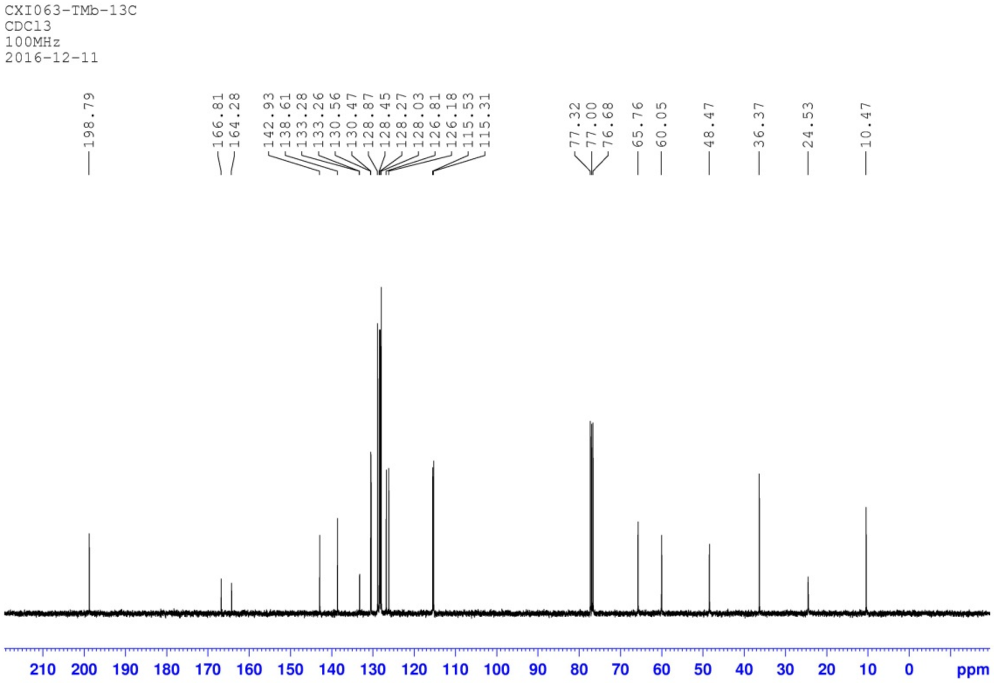


***Supplementary Figure 168.*** $^{13}$C NMR ($CDCl_3$) of the major diastereomer of **5**.

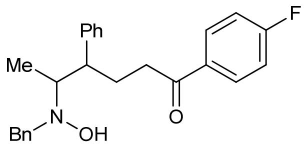


(minor diastereomer of **5**)

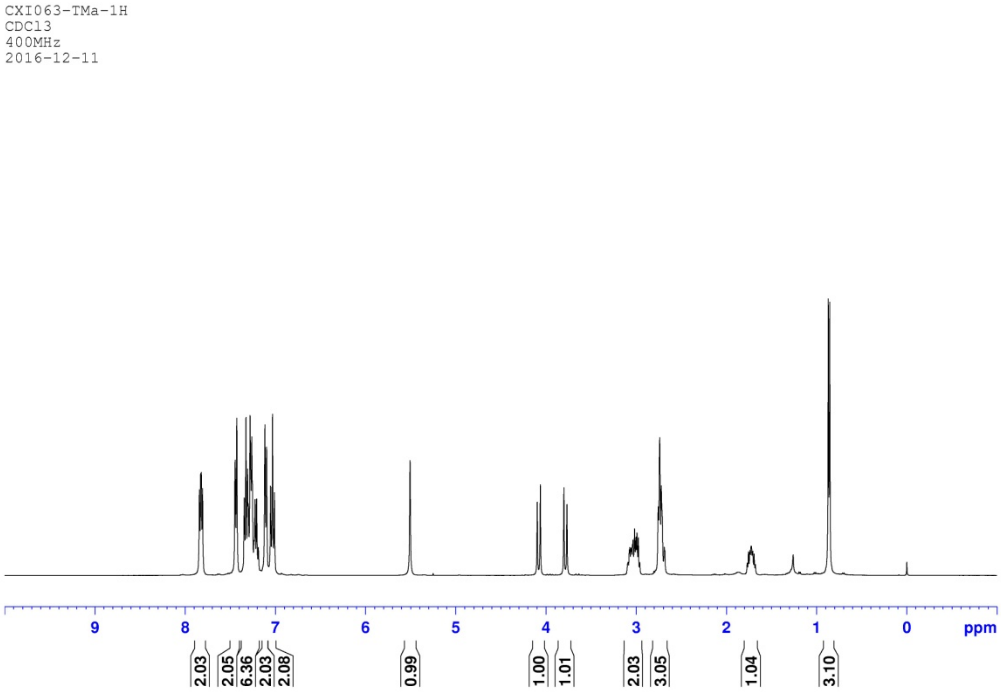


***Supplementary Figure 169.*** $^{1}H$ NMR ($CDCl_3$) of the minor diastereomer of **5**.

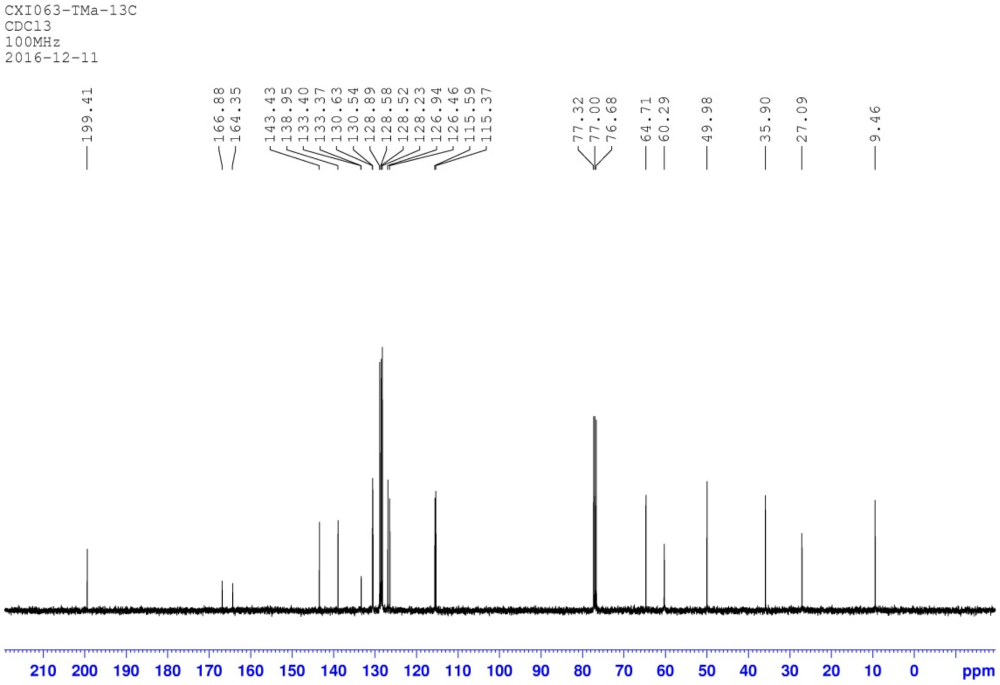


***Supplementary Figure 170.*** $^{13}C$ NMR ($CDCl_3$) of the minor diastereomer of **5**.

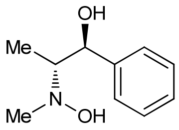


**2n**

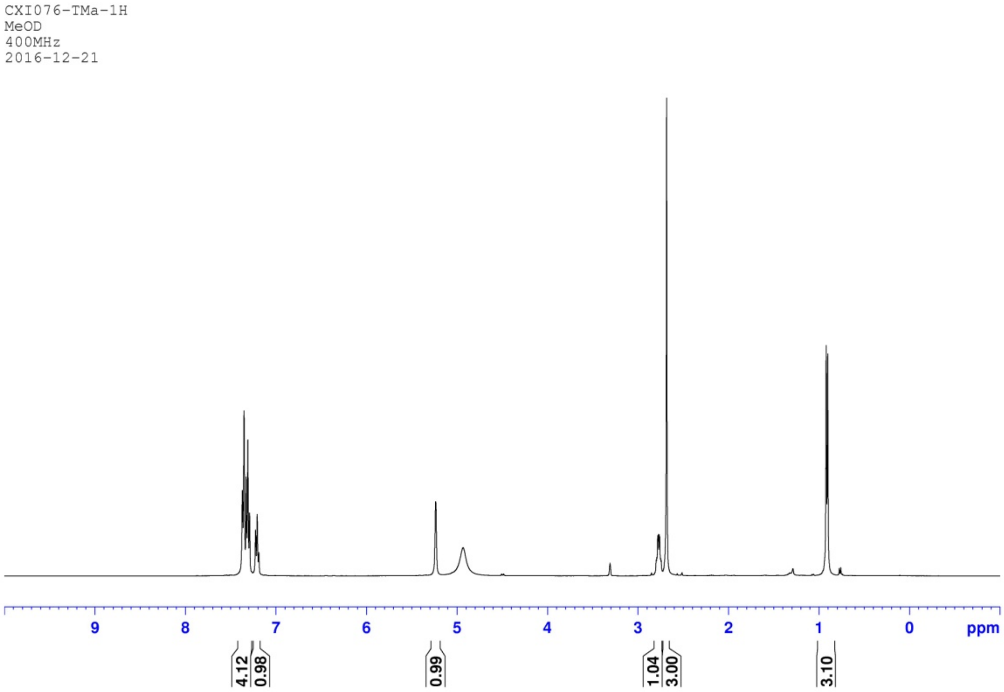


***Supplementary Figure 171.*** $^{1}$H NMR (MeOD) of compound **2n**.

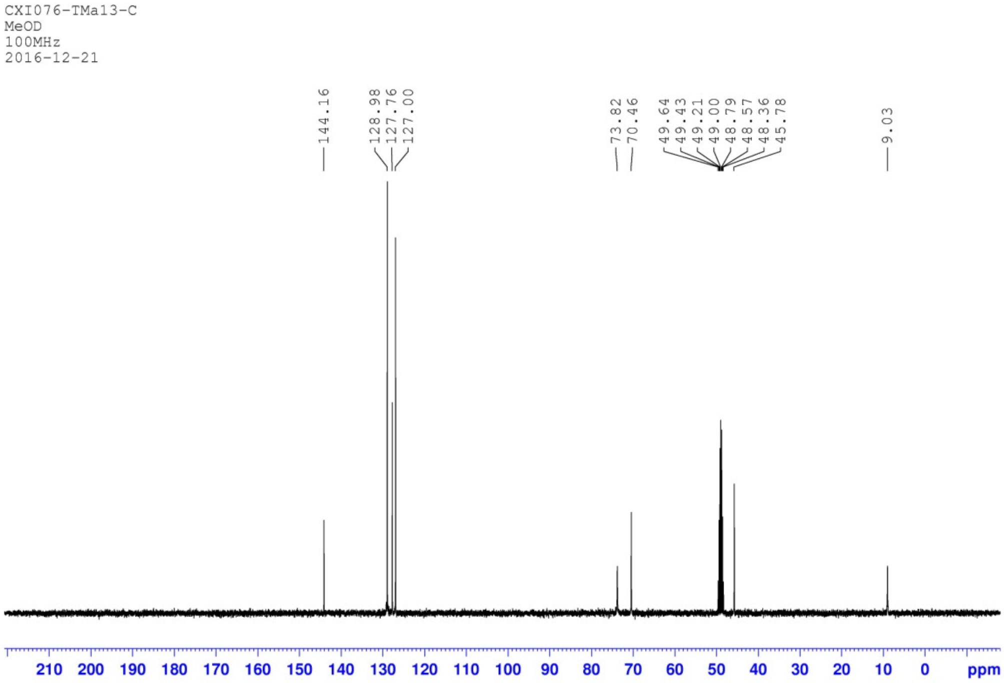


***Supplementary Figure 172.*** $^{13}$C NMR (MeOD) of compound **2n**.

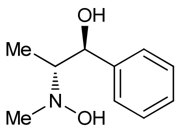


**2n**

[Lux® Amylose-1 column, 30 ºC, hexane/EtOH = 80/20 (v/v), 1.0 mL/min, 225 nm]

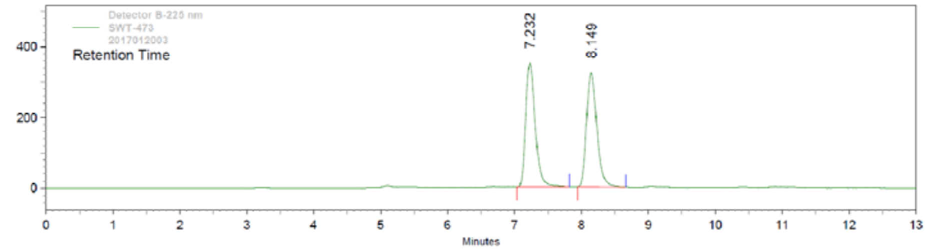


Detector B-225 nm

| Pk # | Retention Time | Area | Area Percent | Height |
|---|---|---|---|---|
| 1 | 7.232 | 3482321 | 49.91 | 351013 |
| 2 | 8.149 | 3495101 | 50.09 | 322859 |

***Supplementary Figure 173.*** Racemate of compound **2n**.

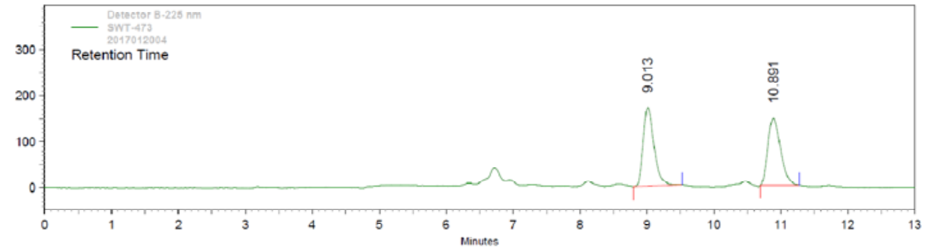


Detector B-225 nm

| Pk # | Retention Time | Area | Area Percent | Height |
|---|---|---|---|---|
| 1 | 9.013 | 1941403 | 50.03 | 171220 |
| 2 | 10.891 | 1938703 | 49.97 | 146651 |

***Supplementary Figure 174.*** Racemate of the diastereomer of **2n**.

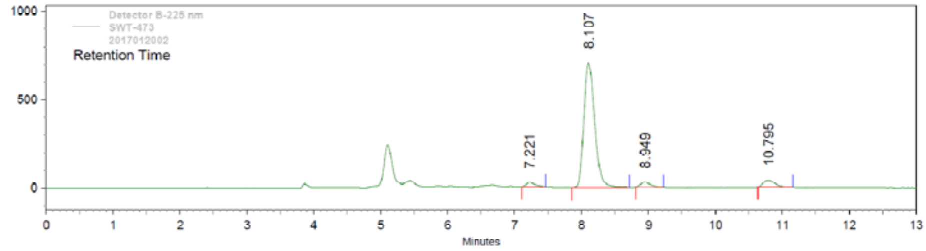


Detector B-225 nm

| Pk # | Retention Time | Area | Area Percent | Height |
|---|---|---|---|---|
| 1 | 7.221 | 225302 | 2.53 | 22680 |
| 2 | 8.107 | 7928720 | 89.01 | 705142 |
| 3 | 8.949 | 267169 | 3.00 | 25805 |
| 4 | 10.795 | 486012 | 5.46 | 39213 |

***Supplementary Figure 175.*** Enantioenriched diastereomeric mixture of **2n**.

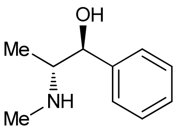


**6n**

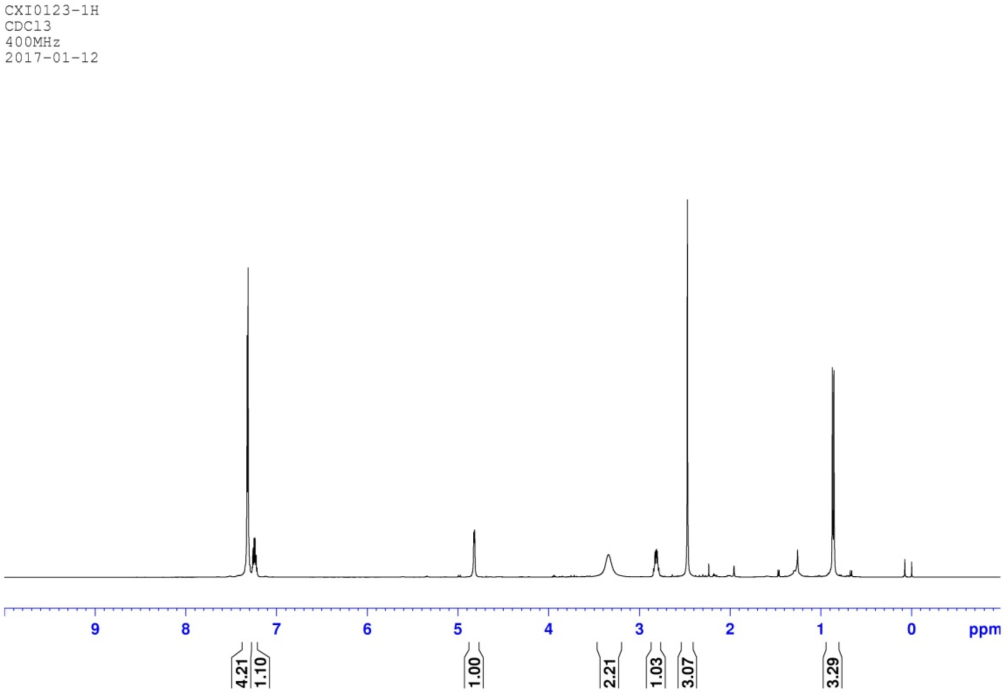


***Supplementary Figure 176.*** $^{1}$H NMR ($CDCl_3$) of compound **6n**.

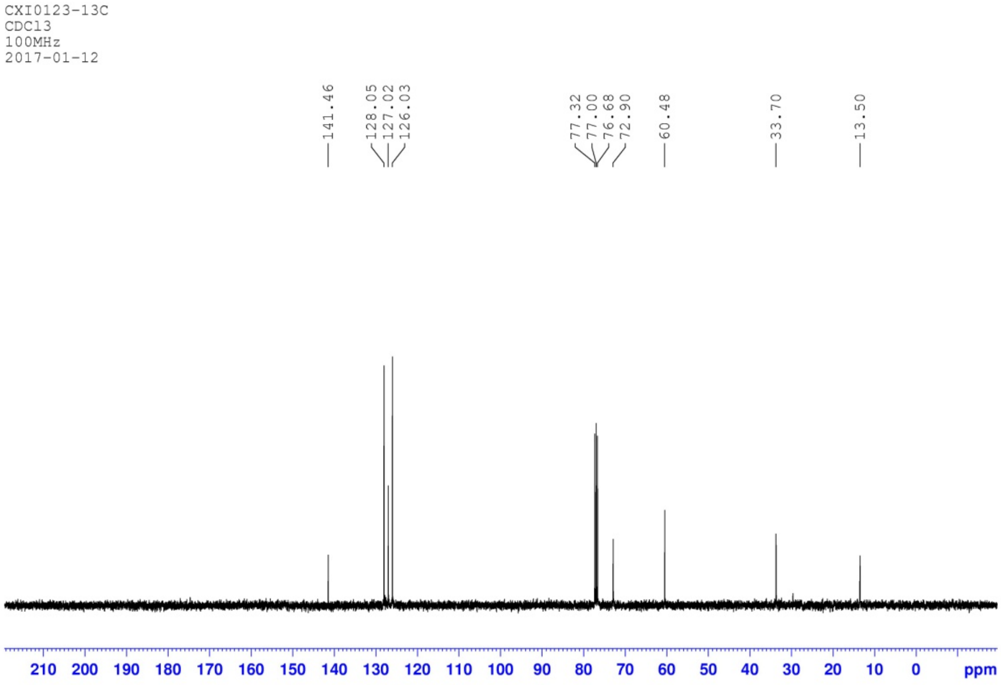


***Supplementary Figure 177.*** $^{13}$C NMR ($CDCl_3$) of compound **6n**.

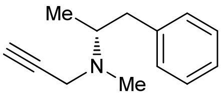


**8**

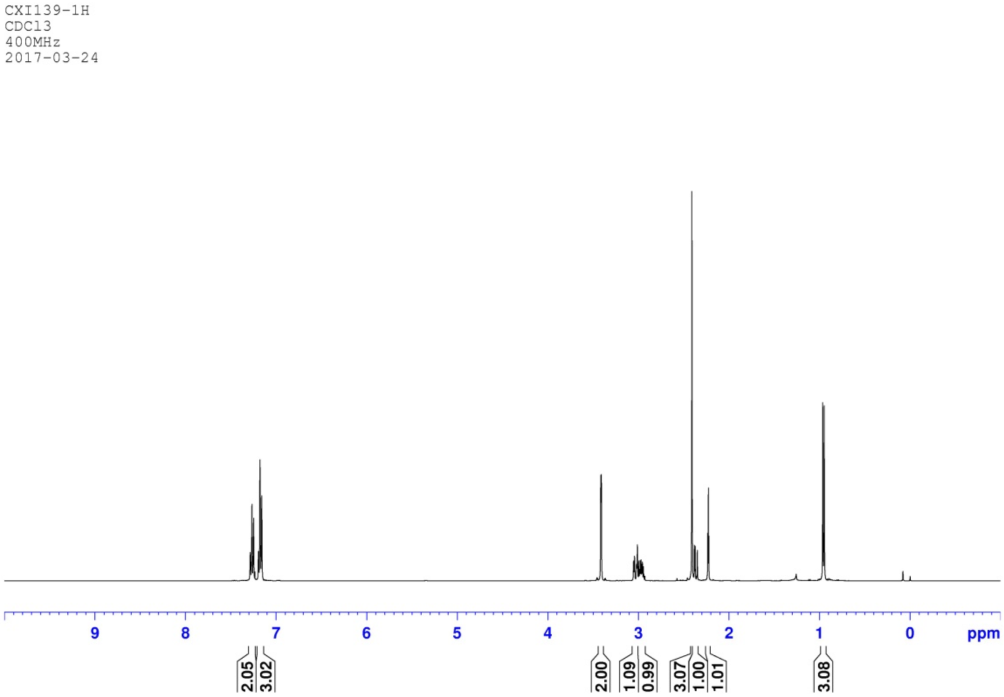


***Supplementary Figure 178.*** $^1$H NMR ($CDCl_3$) of compound **8**.

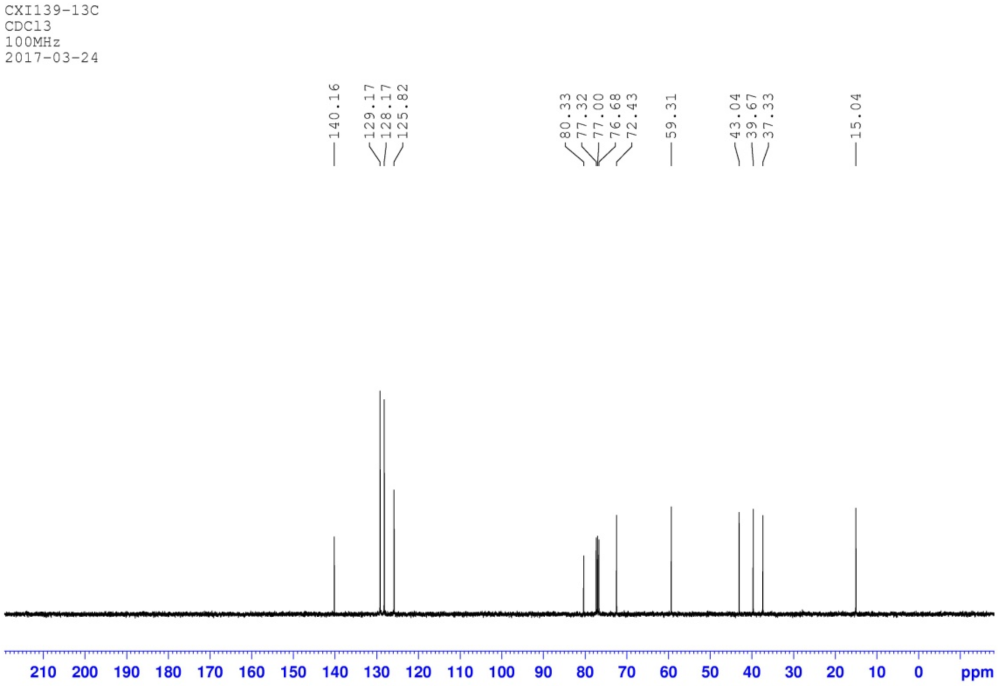


***Supplementary Figure 179.*** $^{13}$C NMR ($CDCl_3$) of compound **8**.

## Supplementary References:


1. Aschwanden, P., Kværnø, L., Geisser, R. W., Kleinbeck, F. & Carreira, E. M. Reduction of 2,3-dihydroisoxazoles to β-amino ketones and β-amino alcohols. *Org. Lett.* **7**, 5741−5742 (2005).
2. Dondoni, A. *et al*. Synthesis of *N*-benzyl nitrones. *Synth. Commun.* **24**, 2537−2550 (1994).
3. Aurich, H. G., Franzke, M., Kesselheim, H. P. & Rohr, M. Steric effects on regioselectivity in 1.3-dipolar cycloaddition of C,N-dialyl nitrones with acceptor-substituted alkynes. *Tetrahedron* **48**, 669−682 (1992).
4. Bagley, M. C. & Tovey, J. Diastereoselective synthesis of *cis*-2,5-disubstituted pyrrolidine *N*-oxides by the retro-Cope elimination. *Tetrahedron Lett.* **42**, 351−353 (2001).
5. Murahashi, S.-I., Imada, Y. & Ohtake, H. Tungstate-catalyzed decarboxylative oxidation of *N*-alkyl-α-amino acids: an efficient method for regioselective synthesis of nitrones. *J. Org. Chem.* **59**, 6170−6172 (1994).
6. Malinowski, J. T., Malow, E. J. & Johnson, J. S. α-Amination of keto-nitrones via Multihetero-Cope rearrangement employing an imidoyl chloride reagent. *Chem. Commun.* **48**, 7568−7570 (2012).
7. Pfeiffer, J. Y. & Beauchemin, A. M. Simple reaction conditions for the formation of ketonitrones from ketones and hydroxylamines. *J. Org. Chem.* **74**, 8381−8383 (2009).
8. Cicchi, S., Marradi, M., Goti, A. & Brandi, A. Manganese dioxide oxidation of hydroxylamines to nitrones. *Tetrahedron Lett.* **42**, 6503−6505 (2001).
9. Li, W. *et al.* A catalytic asymmetric ring-expansion reaction of isatins and α-alkyl-α-diazoesters: highly efficient synthesis of functionalized 2-quinolone derivatives. *Angew. Chem. Int. Ed.* **51**, 8644−8647 (2012).
10. O'Neil, I. A. *et al*. The synthesis and structure of chiral enamine *N*-oxides. *Chem. Commun.* **50**, 7336−7339 (2014).
11. Cicchi, S., Bonanni, M., Cardona, F., Revuelta, J. & Goti, A. Indium-mediated reduction of hydroxylamines to amines. *Org. Lett.* **5**, 1773−1776 (2003).
12. Masson, G., Py, S. & Vallée, Y. Samarium diiodide-induced reductive cross-coupling of nitrones with aldehydes and ketones. *Angew. Chem. Int. Ed.* **41**, 1772−1775 (2002).
13. Xu, J., Samsuri, N. B. & Duong, H. A. Nickel-catalysed cyclopropanation of electron-deficient alkenes with diiodomethane and diethylzinc. *Chem. Commun.* **52**, 3372−3375 (2016).
14. Raffa, R. J., Stern, M. J. & Malspeis, L. Thermometric titration determination of ΔH°, ΔG°, and ΔS° of dissociation of ephedrinium and pseudoephedrinium ions. *Anal. Chem.* **40**, 70−77 (1968).
15. Bornholdt, J., Felding, J., Clausen, R. P. & Kristensen, J. L. Ring opening of pymisyl-protected aziridines with organocuprates. *Chem. Eur. J.* **16**, 12474−12480 (2010).
16. Frisch, M. J. *et al*. *Gaussian 09, Revision A.02* (Gaussian, Inc., Pittsburgh, PA, 2009).
17. Becke, A. D. Density-functional thermochemistry. III. The role of exact exchange. *J. Chem. Phys.* **98**, 5648−5652 (1993).
18. Lee, C., Yang, W. & Parr, R. G. Development of the Colle-Salvetti correlation-energy formula into a functional

of the electron density. *Phys. Rev. B* **37**, 785−789 (1988).

19. Cave, R. J., Burke, K. & Castner, E. W., Jr. Theoretical investigation of the ground and excited states of coumarin 151 and coumarin 120. *J. Phys. Chem. A* **106**, 9294−9305 (2002).

20. Trendafilova, N., Bauer, G. & Mihaylov, T. DFT and AIM studies of intramolecular hydrogen bonds in dicoumarols. *Chem. Phys.* **302**, 95−104 (2004).

21. Hehre, W. J., Ditchfield, R. & Pople, J. A. Self-consistent molecular orbital methods. XII. Further extensions of Gaussian-type basis sets for use in molecular orbital studies of organic molecules. *J. Chem. Phys.* **56**, 2257−2261 (1972).

22. Maron, L. & Eisenstein, O. Do f electrons play a role in the lanthanide-ligand bonds? A DFT study of $Ln(NR_2)_3$; R = H, $SiH_3$. *J. Phys. Chem. A* **104**, 7140−7143 (2000).

23. Petersson, G. A. *et al.* A complete basis set model chemistry. I. The total energies of closed-shell atoms and hydrides of the first-row elements. *J. Chem. Phys.* **89**, 2193−2218 (1988).

24. Dolg, M., Stoll, H., Savin, A. & Preuss, H. Energy-adjusted pseudopotentials for the rare earth elements. *Theor. Chim. Acta* **75**, 173−194 (1989).

25. Cao, X. & Dolg, M. Segmented contraction scheme for small-core lanthanide pseudopotential basis sets. *J. Molec. Struct. (Theochem)* **581**, 139−147 (2002).

26. Dolg, M., Stoll, H. & Preuss, H. A combination of quasirelativistic pseudopotential and ligand field calculations for lanthanoid compounds. *Theor. Chim. Acta* **85**, 441−450 (1993).

27. Gonzalez, C. & Schlegel, H. B. An improved algorithm for reaction path following. *J. Chem. Phys.* **90**, 2154−2161 (1989).

28. Marenich, A. V., Cramer, C. J. & Truhlar, D. G. Universal solvation model based on solute electron density and on a continuum model of the solvent defined by the bulk dielectric constant and atomic surface tensions. *J. Phys. Chem. B* **113**, 6378−6396 (2009).

29. Liu, X., Lin, L. & Feng, X. Chiral *N,N'*-dioxides: new ligands and organocatalysts for catalytic asymmetric reactions. *Acc. Chem. Res.* **44**, 574−587 (2011).
